# Synthesis of Diaryl‐ and Dialkynylphosphinates From Ubiquitous P^V^ Sources via a Redox‐Neutral Approach

**DOI:** 10.1002/advs.202509922

**Published:** 2025-07-02

**Authors:** Tobias Schneider, Kai Schwedtmann, Jannis Fidelius, Rosa M. Gomila, Antonio Frontera, Jan J. Weigand

**Affiliations:** ^1^ Faculty of Chemistry and Food Chemistry Technische Universität Dresden 01062 Dresden Germany; ^2^ Department of Chemistry Universitat de les Illes Balears Palma de Mallorca 07122 Spain

**Keywords:** phosphinates, phosphorylation reagent, redox‐neutral activation, sustainable chemistry, white phosphorus free

## Abstract

Organophosphinic acids, R_2_P(O)(OH), and their derivatives are versatile compounds with applications in catalysis, material chemistry, biomolecular bridging, metal extraction, and flame retardancy. Current synthetic methods primarily rely on white phosphorus (P_4_) as a precursor, which is converted into nucleophilic or electrophilic P‐synthons through acid‐/base‐induced disproportionation (e.g., PH_3_, [H_2_PO_2_]^–^) or chlorination (PCl_3_). However, P_4_ poses significant drawbacks due to its highly pyrophoric nature, environmental hazards, and the energy‐intensive production process from phosphate ores. Previously, we introduced a method to bypass P_4_ in the synthesis of P^V^ compounds through the redox‐neutral activation of phosphates using trifluoromethanesulfonic anhydride (Tf_2_O) and pyridine, yielding the electrophilic PO_2_^+^ phosphorylation reagent [(pyridine)_2_PO_2_][OTf] (**1a**[OTf]). While this reagent exhibits high selectivity for alcohols, amides, and (pseudo)halides, it failed to react efficiently with organometallic compounds to form organophosphinates. Here, we present mechanistic studies that rationalize this limitation and report an optimized strategy, which successfully facilitates reactions with Grignard reagents. By replacing the pyridine leaving group in **1a**[OTf] with 4‐dimethylaminopyridine (DMAP), the reagent [(DMAP)_2_PO_2_][OTf] (**1b**[OTf]) is obtained, which enables the selective synthesis of a broad range of diaryl‐ and dialkynylphosphinates, thereby expanding the scope of redox‐neutral phosphate activation.

## Graphical abstract

Classical strategies for P─C bond formation rely on precursors (e.g., PH_3_, H_2_PO_2_, PCl_3_) obtained from P_4_, which is produced via the energy‐intensive thermal process. Here, an alternative, redox‐neutral approach is explored. Primary phosphate sources are activated with triflic anhydride (Tf_2_O) and 4‐dimethylaminopyridine (DMAP) to a versatile P^V^ precursor, which can be functionalized with Grignard reagents to afford various organophosphinates.

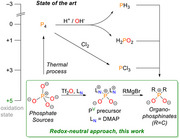

## 1 Introduction

**Scheme 1:**
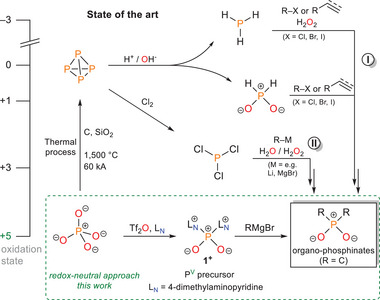
Existing, common strategies for the synthesis of organophosphinates via intermittent reduction of phosphate to P_4_ and alternative, redox‐neutral synthesis (this work).

Organophosphinic acids R_2_P(O)(OH) (R  =  hydrocarbyl group) and their corresponding esters and metal salts (hereafter referred to as “organophosphinates”) represent a class of compounds with diverse applications. Their distinct ability to coordinate metal cations^[^ ^1^ ^]^ enables their broad utilization as ligands in catalysis,^[^ ^2^ ^]^ solar cells,^[^ ^3^ ^]^ material chemistry,^[^ ^4^ ^]^ and metal extraction,^[^ ^5^ ^]^ while they are also widely applied as flame retardants,^[^ ^6^, ^7^ ^]^ and more recently, as bridging agents for biomolecules.^[^ ^8^ ^]^ Additionally, organophosphinates serve as valuable synthetic intermediates, for example, in the reduction to phosphines^[^ ^9^ ^]^ or functionalization to P‐stereogenic compounds^[^ ^10^ ^]^ and light‐emitting materials.^[^ ^11^ ^]^ For their synthesis, there is a well‐established toolbox of transformations in organophosphorus chemistry^[^ ^12^, ^13^ ^]^ which can be combined in various ways to yield the desired organophosphinate. These synthetic schemes follow the general pattern of first forming P─C bonds with the hydrocarbyl substituents, followed by hydrolysis or oxidation of any remaining reactive site on the phosphorus, if necessary. Common approaches to P─C bond formation include palladium‐catalyzed cross‐coupling with aryl halides^[^ ^14^ ^]^ and hydrophosphination of unsaturated hydrocarbons,^[^ ^15^ ^]^ employing nucleophilic phosphorus precursors such as phosphine gas (PH_3_) and hypophosphite ([H_2_PO_2_]^–^), which are produced from acid‐ or base‐catalyzed disproportionation^[^ ^16^ ^]^ of white phosphorus (P_4_) (**Scheme** **1**‐I). Alternatively, electrophilic precursors like PCl_3_, obtained from the chlorination of P_4_, allow for nucleophilic substitution with carbon nucleophiles such as organolithium and Grignard reagents (Scheme 1‐II).^[^ ^12^ ^]^ Among these approaches, hypophosphites particularly stand out as attractive precursors for organophosphinates because, after functionalization of the two P─H bonds, no further oxidation or hydrolysis is needed. This results in greater atom efficiency and reduces the risk of side reactions.^[^ ^13^ ^]^ Consequently, several synthetic approaches based on hypophosphite precursors have been explored, including arylation reactions with palladium‐^[^ ^17^ ^]^ and copper‐based^[^ ^18^ ^]^ catalysts, as well as radical^[^ ^19^ ^]^ or palladium‐^[^ ^20^ ^]^/nickel‐catalyzed^[^ ^21^ ^]^ hydrophosphinylation. The Michaelis‐Arbuzov reaction^[^ ^22^ ^]^ provides another versatile route for P─C bond formation, particularly useful for the synthesis of asymmetrically substituted products. This reaction has been adapted for pharmaceutical applications, for example, in the silicon‐modified synthesis of the phosphinate drug fosinopril.^[^ ^23^ ^]^

A common feature of all these synthetic strategies is their dependency on intermediates derived from white phosphorus (P_4_). While this currently remains unavoidable on an industrial scale, P_4_ poses several significant drawbacks: it is highly pyrophoric and environmentally hazardous,^[^ ^24^, ^25^ ^]^ and its production from phosphate ores via the thermal process is highly energy‐intensive.^[^ ^26^ ^]^ Consequently, chemists have sought to develop strategies that circumvent P_4_ by activating phosphoric acid, which is obtained from the energetically favorable wet process or other secondary phosphate sources.^[^ ^27^ ^]^ However, due to the high stability of the P─O bonds, this has proven challenging. Although a few examples of direct esterification from phosphoric acid have been reported,^[^ ^28^ ^]^ examples of direct P─C bond formation remain rare. Notably, Cummins and co‐workers recently succeeded in activating trimetaphosphate to the bis(trichlorosilyl)phosphide anion,^[^ ^29^ ^]^ which essentially functions as a synthetic surrogate for PH_3_. In a separate contribution, they demonstrated the functionalization of condensed phosphates with acetylides via ball‐milling,^[^ ^30^ ^]^ representing the first redox‐neutral synthesis of organophosphonates RP(O)(OH)_2_ directly from phosphate sources. More recent efforts by Quan and co‐workers could successfully use tetrabutylammonium phosphate to obtain the activated reagent [TBA][PO_2_Cl_2_] (TBA  =  tetrabutylammonium) by chlorination with cyanuric chloride or oxalyl chloride. This reagent could then be employed for direct synthesis of various P^V^ compounds including organophosphinates,^[^ ^31^ ^]^ while in situ reduction with HSiCl_3_ enabled Pd‐catalyzed arylation to triarylphosphines.^[^ ^32^ ^]^

**Scheme 2:**
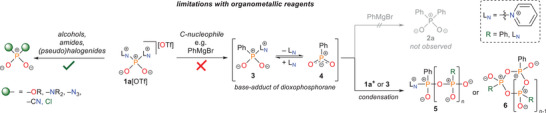
Previous work on redox‐neutral activation and functionalization of various phosphate sources (left)^[^ ^33^ ^]^ and impaired applicability with organometallic nucelophiles like phenylmagnesium bromide due to formation of linear (**5**) or cyclic (**6**) oligomers induced by intermediate **3** as a dominant side reaction (right).

Previously, we described a method for activating phosphate sources using triflic anhydride and pyridine, forming the electrophilic PO_2_^+^ synthon [(pyridine)_2_PO_2_][OTf] (**1a**[OTf]).^[^ ^33^ ^]^ This reagent, recently also used for pyrophosphate synthesis,^[^ ^34^ ^]^ exhibits high selectivity for alcohols, amides, and pseudohalides (**Scheme** **2**, left). In addition, the halogenated transfer reagent [Cl_2_PO_2_]^–^ is also conveniently accessible by reaction with two equivalents of [TBA][Cl] (see S1, Supporting Information, Section 2.2.1). However, it failed to react effectively with organometallic compounds, which are typically regarded as universal carbon nucleophiles, to form organophosphinates (Scheme 2, right). Motivated by these findings, we conducted detailed studies on the reactivity of our pyridinio‐stabilized PO_2_^+^ phosphorylation reagent with carbon nucleophiles. Here, we present a viable strategy that enables the redox‐neutral synthesis of diaryl‐ and dialkynylphosphinates through replacement of the leaving group pyridine in **1a**[OTf] with 4‐dimethylaminopyridine (DMAP), significantly expanding the scope of redox‐neutral phosphate activation.

## 2 Results and Discussion

### 2.1 Mechanistic study of the phosphorylation reagent's reactivity with nucleophiles

Owing to its versatile reactivity with oxygen‐ and nitrogen‐based nucleophiles, we began our investigations with model reactions of **1a**[OTf] and phenylmagnesium bromide (PhMgBr). Upon addition of two equivalents of the Grignard reagent to a suspension of **1a**[OTf] in diethyl ether, the reaction onset was immediately evident by the complete dissolution of the triflate salt. However, to our surprise, the ^31^P nuclear magnetic resonance (NMR) spectrum of the organic phase, recorded after quenching with 1 M aqueous HCl, showed no observable phosphorus‐containing resonances. This indicates that neither the anticipated diphenylphosphinic acid (**2a**) nor any other organically soluble phosphorus‐containing product was formed in appreciable amounts (Scheme 2, right). Attempts to optimize the reaction by varying parameters such as solvent, temperature, or reaction time, as well as employing alternative carbon nucleophiles including organolithium or organozinc reagents, all failed to yield satisfactory results. Instead, the major phosphorus‐containing product consistently appeared as an orange‐colored solid that precipitated upon aqueous quenching. This material is insoluble in both water and most common organic solvents, but displayed acceptable solubility in dimethylformamide (DMF). In DMF, the ^31^P NMR spectrum exhibits very broad resonances ranging from −30 to +15 ppm, indicative of a polymeric structure (see Supporting Information, Section 2.2.2). However, further structural characterization, such as matrix‐assisted laser desorption/ionization ‐ time of flight (MALDI‐TOF) mass spectrometry, was inconclusive.

To rationalize the unexpectedly divergent reactivity of **1a**[OTf] with carbon nucleophiles, in contrast to its previously observed behavior toward oxygen‐ or nitrogen‐centered nucleophiles,^[^ ^33^ ^]^ we turned our attention to the mono‐substituted intermediate **3** (Scheme 2, right). Due to the relatively weak donor ability of pyridine, intermediate **3** may be described as a labile base‐adduct of dioxophosphorane **4**. Such dioxophosphoranes are well‐known for their high electrophilicity, and their pronounced tendency to undergo spontaneous oligomerization or polymerization via P─O─P chain formation, yielding linear or cyclic oligomers, has been well documented in the literature.^[^ ^35^ ^]^ Thus, the successful double substitution of **1a**[OTf] with a carbon nucleophile is critically dependent on the kinetic and thermodynamic stability of mono‐substituted intermediate **3**. If intermediate **3** lacks sufficient stability, as observed with PhMgBr, it appears to initiate oligomerization reactions, incorporating itself and unreacted **1a^+^** as monomeric units. This process outcompetes the desired formation of diphenylphosphinic acid (**2a**), instead favoring the formation of linear (**5**) or cyclic (**6**) phosphorus‐containing oligomers. To validate this mechanistic hypothesis, we continued stirring the reaction mixture in aqueous media for several days following the acidic quench and observed the gradual dissolution of the precipitated material. After prolonged stirring, the ^31^P NMR spectrum of the aqueous phase displayed two new resonances at δ(^31^P) = – 0.3 ppm (s) and δ(^31^P) = 17.0 ppm (t, ^3^ *J*_PH_ = 13.2 Hz). These signals were identified as phosphoric acid and phenylphosphonic acid,^[^ ^36^ ^]^ respectively, which is consistent with slow hydrolytic degradation of the phosphorus‐based polymer backbone (see Supporting Information, Section 2.2.2).

**Scheme 3:**
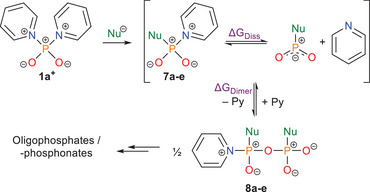
Reaction equation for theoretical studies of dissociation energies ΔG_Diss_ and dimerization energies ΔG_Dimer_ for type **7** intermediates as a function of different nucleophiles.

**Figure 1:**
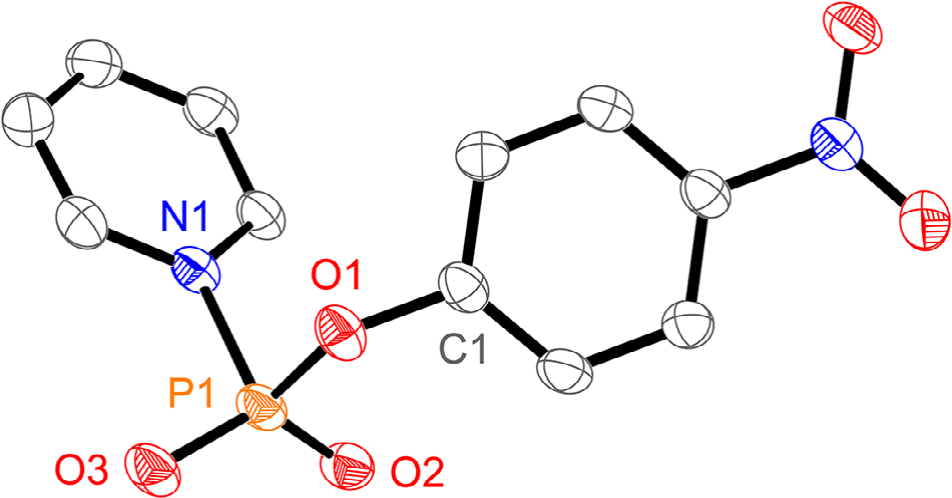
Molecular structure of **7b**. Hydrogen atoms are omitted for clarity. Thermal ellipsoids are displayed at 50 % probability level. Selected bond lengths in Å and angles in (°): P1–N1 1.8048(15), P1–O1 1.6160(13), P1–O2 1.4691(14), P1–O3 1.4637(13), N1–P1–O1 98.33(7), O2–P1–O3 125.58(8).

To gain a deeper understanding of why oligomerization becomes particularly dominant in the presence of carbon nucleophiles, we investigated the system using theoretical methods. To quantify the stability of generalized intermediates of type **7** against spontaneous oligomerization, we calculated the Gibbs free energy for its initial dimerization to yield half an equivalent of the corresponding condensate **8** (**Scheme** **3**, ΔG_Dimer_), using this as a benchmark. In addition, the dissociation energy ΔG_Diss_ toward the corresponding dioxophosphorane and pyridine was determined complementarily. Calculations were performed with an implicit solvent model (Conductor‐like screening model (COSMO) = pyridine) for a set of five different nucleophiles for which experimental observations regarding the empirical stability of **7** were available. The resulting data summarized in **Table** **1** reveals a clear correlation between the nucleophile's basicity and the stability of the related intermediate. As the donor strength of the nucleophile increases, quantified by the pK_A_ of its corresponding acid, the Gibbs free energy of dimerization decreases significantly, indicating reduced thermodynamic stability. With the exception of anionic intermediate **7d^−^**, this is accompanied by a concurrent decline of the dissociation energy ΔG_Diss_, suggesting that a weakening of the P─N bond contributes to the reduced stability. This trend aligns well with experimental findings. While the starting material **1a^+^** (equivalent to **7a^+^** in Scheme 3) forms a stable and isolable triflate salt, the aryloxyphosphinates **7b** and **7c** were only observed in situ by ^31^P NMR following reaction of **1a**[OTf] with the corresponding phenols in pyridine solution (δ(^31^P, **7b**) = – 11.5 ppm (s); δ(^31^P, **7c**)  =  –9.7 ppm (s); see Supporting Information Sections 2.2.3 and 2.2.4). Attempts to isolate these compounds led to partial decomposition into higher oligomers. The slightly more stable derivative **7b** could be characterized by single‐crystal X‐ray diffraction, further supporting the spectral assignment (**Figure** **1**).

**Table 1:** Dissociation energies for intermediates of type **7** (ΔG_Diss_) and corresponding Gibbs free energies of dimerization (ΔG_Dimer_) as a benchmark for stability toward spontaneous oligomerization. Calculations were conducted using pyridine as solvent (RI‐PB86‐D4(COSMO = py)/def2‐TZVP level of theory).

Nu	pK_A_(Nu─H)^a)^	ΔG_Diss_ in kcal/mol	ΔG_Dimer_ in kcal/mol
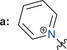	5.23	+ 38.0	+ 27.4
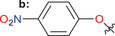	7.15	+ 27.8	+ 13.0
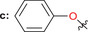	9.99	+ 25.8	+ 10.6
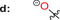	29	+ 0.4	+ 5.4
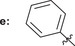	43	+ 15.6	+ 2.6

^a)^ pK_A_ values from reference.^[^ ^37^ ^]^

The tendency to oligomerize upon concentration becomes even more pronounced for pyridiniophosphonate **7d^−^**, which is formed as an intermediate during the synthesis of **1a**[OTf] and has previously been shown to reversibly form various oligomers in pyridine suspensions.^[^ ^33^ ^]^ In contrast, oligomerization of arylphosphinate **7e** occurs rapidly and irreversibly, as demonstrated by the failure to obtain diphenylphosphinic acid (**2a**) from **1a**[OTf] even in the presence of stoichiometric PhMgBr. In general, the difficulty of functionalizing precursor **1a**[OTf] with carbon nucleophiles arises from their higher basicity compared to oxygen‐ or nitrogen‐based nucleophiles, which promotes oligomerization of type **7** intermediates and prevents the second substitution reaction. This may be interpreted as a shift from a predominantly electrophilic character in **7a^+^** to a more amphiphilic character in **7e**, driven by increasing electron density at the phosphoryl oxygen introduced by more electron‐rich substituents.

**Scheme 4:**
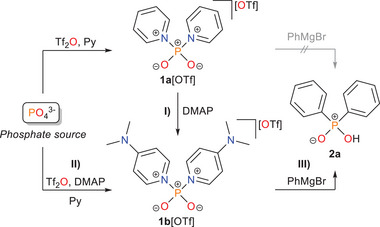
Synthesis of the phosphinylation reagent **1b**[OTf] from **1a**[OTf] (**I**), direct one‐pot synthesis from primary phosphate sources (**II**), and subsequent functionalization to diphenylphosphinic acid **2a** (**III**). *Reagents and conditions*: **I)** + 2 DMAP, – 2 pyridine, CH_2_Cl_2_, 20 h (92 %, 4.13 g); **II)** from H_3_PO_4_: + 2.2 Tf_2_O, + 5.5 DMAP, – 3 [HDMAP][OTf], pyridine, 45 °C, 3 days (98 %, 4.47 g); from P_2_O_5_: + 1.1 Tf_2_O, + 4.4 DMAP, pyridine, 45 °C, 4 days (97 %, 443.75 g); **III)** + 2.5 PhMgBr, + HCl, – 2 DMAP, – MgBr_2_, – MgCl[OTf], toluene, 4 h (67 %, 146 mg).

Based on this interpretation supported by theoretical results, we hypothesized that increasing the basicity of the leaving group could reduce the overall electrophilicity of the PO_2_^+^ reagent, thereby stabilizing the intermediate and enabling successful functionalization with organometallic reagents. Seeking a simple and synthetically accessible solution, we selected 4‐dimethylaminopyridine (DMAP, pK_A_([H–DMAP]^+^)  =  9.2)^[^ ^37^ ^]^ as an attractive alternative leaving group. The corresponding PO_2_^+^ synthon **1b**[OTf] had previously been obtained from **1a**[OTf] by simple exchange with DMAP in 92 % yield (**Scheme** **4**‐I).^[^ ^33^ ^]^

**Figure 2:**
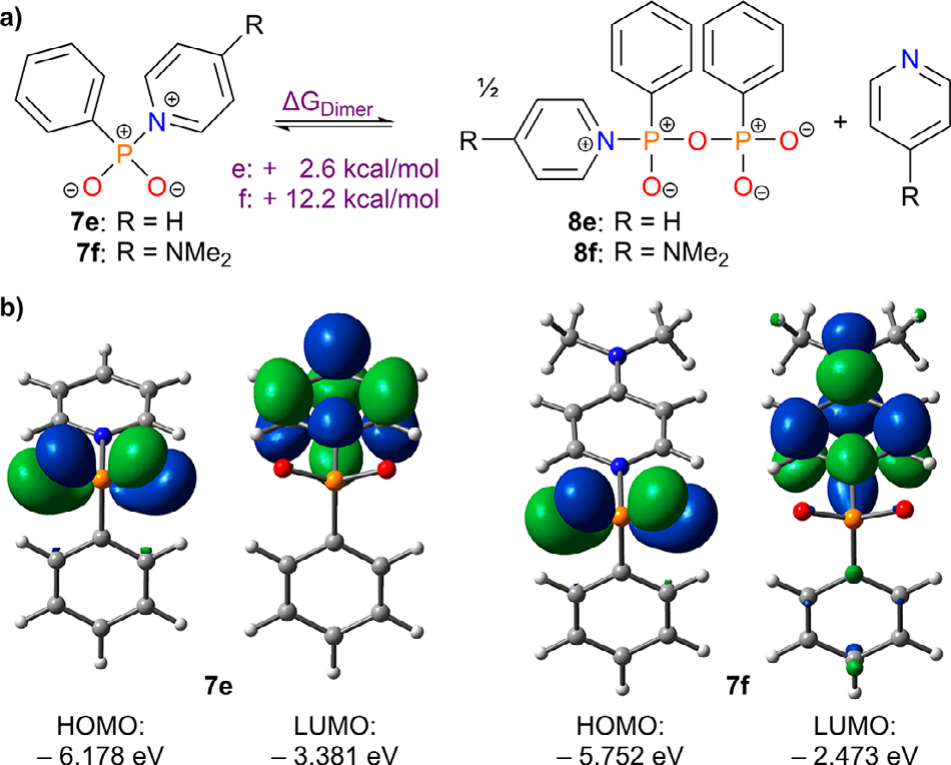
Comparison of intermediates **7e** and **7f** (from reactions of **1a,b**[OTf] with PhMgBr) based on theoretical analysis: a) Dimerization energy (ΔG_Dimer_) as a measure of stability; b) HOMO and LUMO energies and orbital distributions.

Following the same theoretical protocol as for Table 1, we also examined intermediate **7f** (**Figure** **2**). As anticipated, compound **7f** is significantly less electrophilic than **7e**, as evidenced by a 0.908 eV increase in the lowest unoccupied molecular orbital (LUMO) energy. In contrast, the highest occupied molecular orbital (HOMO) energy increases by only 0.426 eV, resulting in a larger HOMO‐LUMO gap, which correlates with an enhanced thermodynamic stability. This finding is supported by the significantly increased calculated dimerization energy for **8f** of 12.2 kcal mol^−1^, indicating that **1b**[OTf] should be able to form **2a** via reaction with PhMgBr. To our satisfaction, this prediction was confirmed experimentally. Upon reaction with 2.5 equivalents of PhMgBr in toluene, followed by aqueous work‐up, diphenylphosphinic acid (**2a**) was obtained in an isolated yield of 67 % (Scheme 4‐III). Notably, salt **1b**[OTf] can also be synthesized directly from primary phosphate sources via a convenient one‐pot protocol. Following the generation of **1a**[OTf] in pyridine suspension as previously described,^[^ ^33^ ^]^ the addition of a slight excess (10%) of DMAP leads to the clean precipitation of **1b**[OTf] without intermediate isolation. Filtration of the reaction mixture then affords **1b**[OTf] in yields exceeding 97 % from either H_3_PO_4_ or P_2_O_5_ (Scheme 4‐II). Gratifyingly, this procedure is readily scalable and enabled us to prepare precursor **1b**[OTf] on a molar scale with batch sizes exceeding 400 g – highlighting the potential for future large‐scale applications. It should also be noted that derivative **1b**[OTf] is significantly more moisture‐resistant than **1a**[OTf]. While both compounds should be stored in a water‐free inert atmosphere to avoid hydrolysis of the reactive P─N bonds, and do not show any appreciable decomposition under such conditions over months, short exposure of **1b**[OTf] to atmospheric moisture is generally unproblematic. For example, a sample of **1b**[OTf] that was stored under air for 4 days still retained 85 % of the starting material as evidenced by ^31^P NMR spectroscopy. In contrast, exposure of **1a**[OTf] to air for the same time led to complete hydrolysis of the compound, with pyridinium tri‐ and tetrametaphosphate as the main hydrolysis products (see Supporting Information, Section 2.2.8).

### 2.2 Application of **1b**[OTf] as a precursor for phosphinates

**Scheme 5:**
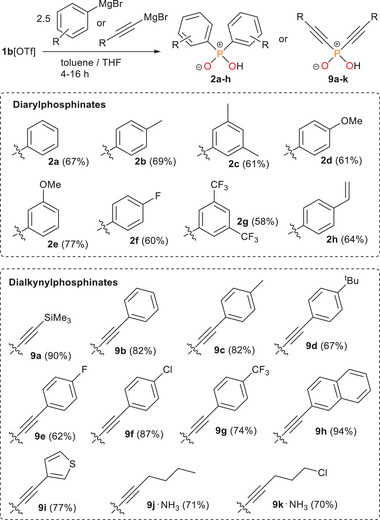
Synthetic scope of the reaction of **1b**[OTf] with Grignard reagents. *Reagents and conditions*: + 2.5 RMgBr, + HCl, – 2 DMAP, – MgBr_2_, – MgCl[OTf], toluene/THF, 4 – 16 h at room temperature. Grignard reagents for diarylphosphinates were received commercially (**2a‐b**, **2d‐f**) or prepared in situ (**2c**, **2g‐h**) from aryl bromides (2.5 eq.) and Mg turnings (2.5 eq.). Grignard reagents for dialkynylphosphinates were prepared in situ by deprotonation of terminal alkynes (3 eq.) with ethylmagnesium bromide solution (3 M in Et_2_O, 2.5 eq.). For synthetic details and characterization data (see Supporting Information, Sections 2.3 and 2.4).

Having addressed the previous reagent's limitation with carbon nucleophiles, we next explored the scope of organophosphinates accessible from **1b**[OTf]. Based on the initially optimized synthesis of diphenylphosphinic acid (**2a**), a general procedure was developed: treatment of **1b**[OTf] with a slight excess of the corresponding Grignard reagent (2.5 eq.) in either tetrahydrofuran (THF) or toluene for 4–16 h, followed by quenching with 1 M aqueous HCl, allowed for the extraction of the phosphinic acid into the organic phase. Reaction cooling was found to be unnecessary and did not significantly improve yields, likely due to the low solubility of **1b**[OTf], which already slows the reaction relative to typical Grignard reactions. The crude products were then purified by re‐extraction into an alkaline aqueous phase and subsequent precipitation by acidification, affording the free acids as bench‐stable, colorless powders. This protocol was first tested for a variety of diarylphosphinic acids, which are relevant, for instance, in the preparation of P‐stereogenic compounds,^[^ ^38^ ^]^ and gave phosphinic acids **2a‐h** in good isolated yields of 58–77 % on 1–2 mmol scale (**Scheme** **5**). Notably, the procedure does not require chromatographic purification, making it readily scalable. Characterization was performed by heteronuclear NMR, infrared (IR) and Raman spectroscopy, and elemental analysis to confirm purity of the bulk sample. Additionally, all products were structurally verified by single crystal X‐ray diffraction (see Supporting Information, Section 2.3). The scope demonstrated tolerance for common functional groups in Grignard syntheses like alkyl‐ (**2b**, **2c**), alkoxy‐ (**2d**, **2e**) fluoroaryl‐ (**2f**), fluoroalkyl‐ (**2** **g**) and vinyl‐substituents (**2** **h**), in both *para*‐ and *meta*‐positions. However, *ortho*‐substitution was found to be a limiting factor, as demonstrated by the reaction with 2‐mesitylmagnesium bromide. In this case, the expected phosphinic acid was not formed, likely due to the increased steric hindrance of the reagent (see Supporting Information, Section 2.2.5). Aiming to assess the influence of sterics on the reaction more comprehensively, we additionally conducted systematic studies with alkyl magnesium halides of varying bulkiness (MeMgBr, EtMgBr, *^i^* PrMgCl, *^t^* BuMgCl). These investigations demonstrated that both *^i^* PrMgCl and *^t^* BuMgCl did not result in appreciable formation of the related phosphinate as evidenced by ^31^P NMR spectroscopy, while EtMgBr already displayed a considerably decreased selectivity (< 10 % from integration of all signals in the ^31^P NMR spectrum) compared to MeMgBr (>90 %, experimental details see Supporting Information, Section 2.2.6).

**Figure 3:**
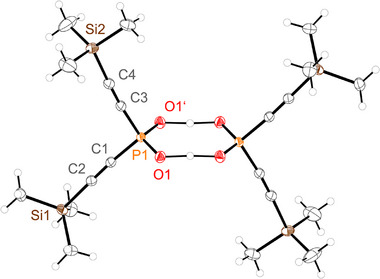
Dimeric molecular structure of **9a** Thermal ellipsoids are displayed at 50 % probability level. Selected bond lengths in Å and angles in (°): **9a**: P1–O1 1.5129(9), O1–H1 1.232(3), P1–C1 1.7465(17), P1–C3 1.7455(17), C1–C2 1.205(3), C3–C4 1.206(3), C2–Si1 1.8655(17), C4–Si2 1.8663(19), C1–P1–C3 106.38(8).

**Scheme 6:**
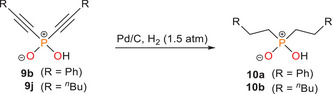
Pd‐catalyzed hydrogenation of dialkynylphosphinates **9b** and **9j** to dialkylphosphinates **10a** and **10b**. *Reagents and conditions*: + H_2_ (1.5 atm), 10 wt.% Pd/C, EtOH, 16 h (**10a**: 92 %, **10b**: 94 %).

Given the importance of the Grignard reagent's steric demand in these reactions, we shifted our attention to terminal alkynes for expanding the substrate scope. The incorporation of two C≡C triple bonds on a P^V^ center is synthetically attractive due to its potential in cycloaddition chemistry.^[^ ^39^ ^]^ However, while dialkynylphosphine oxides have been widely reported, their phosphinate counterparts remain largely underrepresented. Owing to the high C─H acidity of terminal alkynes, they can be conveniently deprotonated with alkyl Grignard reagents to form the related alkynyl Grignard in situ. Using this strategy, we developed a synthetic protocol for dialkynylphosphinates: the terminal alkynes (3.0 eq.) were deprotonated with ethylmagensium bromide (2.5 eq.) in THF, followed by the addition of **1b**[OTf] and stirring for 4–16 h. The work‐up procedure was slightly modified for this class of compounds and they were obtained in good to very good isolated yields of 62%–94% (**Scheme** **5**). Some derivatives (**9c‐h**) were found to be poorly soluble as sodium salts in alkaline solution and were therefore precipitated directly before conversion to the fee acid. Derivatives **9j** and **9k**, isolated as oils, were converted to their ammonium salts for characterization. Compound **9a** crystallized readily from pentane and was therefore isolated directly by recrystallization from the crude extract (structure shown in **Figure** **3**). All compounds **9a‐k** were fully characterized by heteronuclear NMR spectroscopy, IR and Raman spectroscopy, and elemental analysis. Additionally, compounds **9a‐j** could be structurally verified by single crystal X‐ray diffraction (see Supporting Information, Section 2.4). The connectivity of derivative **9k** ⋅ NH_3_ was also confirmed by X‐ray diffraction experiments, however, full crystallographic characterization was not possible due to reproducible sample decay upon prolonged X‐ray irradiation. In comparison to the diarylphosphinic acids (**2a‐h**, δ(^31^P) ranges from + 14 to + 35 ppm), the dialkynyl derivatives display significantly upfield‐shifted ^31^P resonances, ranging from – 33.6 to – 17.8 ppm. Additionally, the isolated yields were generally higher (62% to 94%), likely due to the lower steric demand of the alkynyl Grignards. To further broaden the accessible structural motifs, we also explored the hydrogenation to the corresponding dialkylphosphinates exemplarily for an aryl‐ (**9a**) and alkyl‐derivative (**9j**, **Scheme** **6**). This reaction proceeds cleanly via Pd/C catalysis in ethanol under mild H_2_ pressure (1.5 atm), affording bis(phenylethyl)phosphinic acid (**10a**) and dihexylphosphinic acid (**10b**) in excellent yields of 92 and 94%, respectively. These results demonstrate that **1b**[OTf] is not only a robust precursor for diaryl‐ and dialkynylphosphinates, but also a versatile entry point into the broader class of dialkylphosphinates, which are widely used as flame retardants (e.g., Al[Et_2_PO_2_], “*Exolit OP*”)^[^ ^40^ ^]^ or metal extracting agents (e.g., bis(2,2,4‐trimethylpentyl)phosphinic acid, “*Cyanex 272*”).^[^ ^41^ ^]^

## 3 Conclusion

In summary, we have developed a redox‐neutral synthetic strategy for direct P─C functionalization of various phosphate sources, thereby circumventing the need for white phosphorus (P_4_) and addressing the key limitations of our previously reported method.^[^ ^33^ ^]^ While the earlier PO_2_^+^ synthon, [(pyridine)_2_PO_2_][OTf] (**1a**[OTf]), proved unsuitable for organophosphinate synthesis, due to a mechanistically understood oligomerization side reaction, a strategic replacement of the pyridyl leaving group sufficiently suppressed this undesired pathway. The resulting alternative PO_2_^+^ synthon, [(DMAP)_2_PO_2_][OTf] (**1b**[OTf]), enabled the direct synthesis of a broad range of organophosphinates, including diarylphosphinates (**2a‐h**) and dialkynylphosphinates (**9a‐k**), with isolated yields of up to 94 %. Furthermore, two dialkylphosphinates (**10a, 10b**) were successfully accessed via Pd‐catalyzed hydrogenation of the corresponding alkynyl moiety.

Thanks to its operational simplicity, compatibility with commercially available reagents, and the promising synthetic potential of the dialkynylphosphinate scaffolds, this methodology offers a versatile and scalable platform for further functionalization and application. Beyond the immediate scope of this study, our findings highlight the untapped potential of *N*‐onio‐substituted phosphorylation reagents, such as [(L_N_)_2_PO_2_]^+^‐type species, for fine‐tuning reactivity in contrast to the classical halogenated phosphorylation reagents. These systems open the door to new reactivity patterns and warrant further investigation in the broader context of organophosphorus chemistry.

## Conflict of Interest

The authors declare no conflict of interest.

## Supporting information



Supporting Information

Supporting Information

## Data Availability

The data that support the findings of this study are available in the supplementary material of this article.
