# Supplementary material for: Synthesis of Diaryl‐ and Dialkynylphosphinates From Ubiquitous PV Sources via a Redox‐Neutral Approach
Source: Adv Sci (Weinh). 2025 Jul 2;12(36):e09922. doi: 10.1002/advs.202509922 (PMC12463002; doi:10.1002/advs.202509922)
Supplement: Supplementary file 1 — Supporting Information [file ADVS-12-e09922-s002.pdf]

ADVANCED SCIENCE
Open Access

Supporting Information

for *Adv. Sci.*, DOI 10.1002/advs.202509922

Synthesis of Diaryl- and Dialkynylphosphinates From Ubiquitous P^V Sources via a Redox-Neutral Approach

*Tobias Schneider, Kai Schwedtmann, Jannis Fidelius, Rosa M. Gomila, Antonio Frontera and Jan J. Weigand**

TABLE OF CONTENTS

1. General remarks, materials and methods 4

2. Synthetic details and characterization data 6

2.1. Preparation of $(DMAP)_2PO_2$[OTf] (**1b**[OTf]) 6

*2.1.1. Small-scale preparation from [(pyridine)$_2PO_2$][OTf] (**1a**[OTf]) 6*
2.1.2. Small-scale preparation from H_3PO_4 9
2.1.3. Large-scale preparation from P_2O_5 11

2.2. Reactivity studies 14

*2.2.1. Reaction of [(pyridine)$_2PO_2$][OTf] (**1a**[OTf]) with tetrabutylammonium chloride ([TBA][Cl]) 14*
*2.2.2. Reaction of [(pyridine)$_2PO_2$][OTf] (**1a**[OTf]) with phenylmagnesium bromide 15*
*2.2.3. Reaction of [(pyridine)$_2PO_2$][OTf] (**1a**[OTf]) with p-nitrophenol 17*
*2.2.4. Reaction of [(pyridine)$_2PO_2$][OTf] (**1a**[OTf]) with phenol 18*
*2.2.5. Reaction of [(DMAP)$_2PO_2$][OTf] (**1b**[OTf]) with 2-mesitylmagnesium bromide 19*
*2.2.6. Reaction of [(DMAP)$_2PO_2$][OTf] (**1b**[OTf]) with alkyl Grignard reagents $H_n(Me)_{3-n}C$–MgX 20*
*2.2.7. Reaction of [(DMAP)$_2PO_2$][OTf] (**1b**[OTf]) with other incompatible Grignard reagents 22*
*2.2.8. Stability tests of **1a**[OTf] and **1b**[OTf] 26*

2.3. Preparation of Diarylphosphinates 28

2.3.1. General procedure 28
*2.3.2. Preparation of Diphenylphosphinic acid (**2a**) 29*
*2.3.3. Preparation of Di-p-tolylphosphinic acid (**2b**) 32*
*2.3.4. Preparation of Bis(3,5-dimethylphenyl)phosphinic acid (**2c**) 35*
*2.3.5. Preparation of Bis(4-methoxyphenyl)phosphinic acid (**2d**) 38*
*2.3.6. Preparation of Bis(3-methoxyphenyl)phosphinic acid (**2e**) 41*
*2.3.7. Preparation of Bis(4-fluorophenyl)phosphinic acid (**2f**) 44*
*2.3.8. Preparation of Bis(3,5-bis(trifluoromethyl)phenyl)phosphinic acid (**2g**) 47*
*2.3.9. Preparation of Bis(4-vinylphenyl)phosphinic acid (**2h**) 50*

2.4. Preparation of Dialkynylphosphinates 53

*2.4.1. Preparation of Bis(trimethylsilylethynyl)phosphinic acid (**9a**) 53*
*2.4.2. General procedure for compounds **9b-k** 56*
*2.4.3. Preparation of Bis(phenylethynyl)phosphinic acid (**9b**) 58*
*2.4.4. Preparation of Bis(p-tolylethynyl)phosphinic acid (**9c**) 61*
*2.4.5. Preparation of Bis((4-tert-butylphenyl)ethynyl)phosphinic acid (**9d**) 64*
*2.4.6. Preparation of Bis((4-fluorophenyl)ethynyl)phosphinic acid (**9e**) 67*
*2.4.7. Preparation of Bis((4-chlorophenyl)ethynyl)phosphinic acid (**9f**) 70*
*2.4.8. Preparation of Bis((4-(trifluoromethyl)phenyl)ethynyl)phosphinic acid (**9g**) 73*
*2.4.9. Preparation of Bis(2-naphtylethynyl)phosphinic acid (**9h**) 77*
*2.4.10. Preparation of Bis(thiophen-3-ylethynyl)phosphinic acid (**9i**) 80*
*2.4.11. Preparation of Ammonium bis(1-hexynyl)phosphinate (**9j** · NH_3) 83*
*2.4.12. Preparation of Ammonium bis(5-chloro-1-pentynyl)phosphinate (**9k** · NH_3) 86*

2.5. Reduction of Dialkynylphosphinates 89
*2.5.1. Preparation of Bis(phenylethyl)phosphinic acid (**10a**) 89*
*2.5.2. Preparation of Dihexylphosphinic acid (**10b**) 92*

3. X-ray Diffraction Refinements 95

3.1. General remarks........ 95

3.2. Crystallographic data 96

4. Computational Investigations........ 102

4.1. Theoretical methods........ 102

4.2. Cartesian Coordinates........ 102

5. References 112

1. General remarks, materials and methods

All **manipulations** with the exception of aqueous work-up procedures were performed in a Glovebox or using Schlenk techniques under an atmosphere of purified argon or nitrogen. Dry, oxygen-free **solvents** were distilled from either CaH_2 or potassium. Deuterated solvents were purchased from Merck, Deutero or Eurisotop. Solvents for use under inert conditions were stored over molecular sieves (4 Å: CH_2Cl_2, $CHCl_3$, *n*-pentane, *n*-hexane, benzene, toluene, Et_2O, THF, pyridine, DMF, CD_2Cl_2, $CDCl_3$, DMSO-d_6; 3 Å: CH_3CN, CD_3CN, CD_3NO_2). All glassware was oven-dried at 150 °C prior to use.

The **chemicals** crystalline phosphoric acid (H_3PO_4), phosphorous pentoxide (P_4O_{10}), 4-dimethylaminopyridine (DMAP), magnesium turnings, tetrabutylammonium chloride ([TBA][Cl]), and Palladium on carbon (10 wt.% loading) were purchased from either Sigma Aldrich, Fischer Scientific, TCI chemicals or Carl Roth, stored in an inert atmosphere and used without further purification. Phenol and *p*-nitrophenol were purchased from Sigma Aldrich and sublimed prior to use. Triflic anhydride (Tf_2O) [Tf = CF_3SO_2] was received from Solvay and distilled over P_4O_{10} prior to use. [(pyridine)$_2$$PO_2$][OTf] (**1a**[OTf]) was synthesized according to the previously published procedure.[1] The **Grignard reagents** phenylmagnesium bromide, 2-mesityl-magnesium bromide, methylmagnesium bromide, ethylmagnesium bromide, *iso*-propyl-magnesium chloride, *tert*-butylmagnesium chloride, vinylmagnesium bromide, cyclohexyl-magnesium chloride, *p*-tolylmagnesium bromide, *p*-methoxyphenylmagnesium bromide, *m*-methoxyphenylmagnesium bromide, and *p*-fluorophenylmagnesium bromide were purchased from Sigma Aldrich as either THF or Et_2O solutions and used as received. All other Grignard reagents were prepared *in situ* as described in sections 2.3 and 2.4 either from the aryl halides benzyl chloride, 1-bromo-3,5-dimethylbenzene, 1,3-bis(trifluoromethyl)-5-bromobenzene, and 1-bromo-4-vinylbenzene or from the terminals alkynes 1-bromo-3-ethynylbenzene, 2-ethynylpyridine, trimethylsilylacetylene, phenylacetylene, *p*-tolylacetylene, *p*-*tert*-butylacetylene, *p*-fluoro-phenylacetylene, *p*-chlorophenylacetylene, *p*-(trifluoromethyl)-phenylacetylene, 2-ethynyl-naphtalene, 3-ethynylthiophene, 1-hexyne, and 5-chloro-1-pentyne, which were purchased from BLDpharm and used without further purification.

NMR spectra were measured using a Bruker AVANCE III HD Nanobay 400 MHz UltraShield equipped with a BBO probe (^{1}H: 400.13 MHz, ^{13}C: 100.61 MHz, ^{19}F: 376.50 MHz, ^{23}Na: ^{31}P: 161.98 MHz), or on a Bruker AVANCE III HDX, 500 MHz Ascend equipped with a BBO(F) ProdigyCryo probe (^{1}H: 500.13 MHz, ^{13}C: 125.75 MHz, ^{19}F: 470.59 MHz, ^{29}Si: 99.36 MHz, ^{31}P: 202.45 MHz). Reported numbers assigned to atoms in the ^{13}C spectra were indirectly deduced from the cross-peaks in 2D correlation experiments (HMBC, HSQC). Chemical shifts are referenced to δ(Me_4Si) = 0.00 ppm (^{1}H, ^{13}C, ^{29}Si, external standard), δ($CFCl_3$) = 0.00 ppm (^{19}F,

external standard), and δ(H_3PO_4, 85%) = 0.00 ppm (^{31}P, external standard). Unless otherwise stated, all NMR spectra were measured at 300 K. Chemical shifts (δ) are reported in ppm. Coupling constants (*J*) are reported in Hz. Splitting patterns are named by convention (s = singlet, d = doublet, t = triplet, q = quartet, quin = quintet, sxt = sextet, non = nonet, m = multiplet). Splitting patterns arising from similar coupling constants to chemically inequivalent nuclei are denoted as *pseudo*-multiplets.

Melting points were recorded with an electrothermal melting point device (Büchi Switzerland, Melting point M-560) in sealed capillaries under nitrogen atmosphere and are uncorrected.

Infrared (IR) spectra were recorded at room temperature using a Bruker Vertex 70 instrument with an ATR unit (diamond). **Raman** spectra were recorded at room temperature on a Bruker RAM II module or Bruker RFS 100 spectrometer (Nd:YAG laser, 1064 nm). Raman intensities are reported in percent relative to the most intense peak and are given in parenthesis. IR intensities are reported relative to the most intense peak and are given in parenthesis using the following abbreviations: vw = very weak, w = weak, m = medium, s = strong, vs = very strong.

Elemental analyses (EA) were performed on a Vario MICRO cube Elemental Analyzer by Elementar Analysatorsysteme GmbH in CHNS mode.

Simultaneous thermal analysis (STA) was conducted with a STA 8000 apparatus (Perkin Elmer) under helium gas flow (20 mL/min) and a heating rate of 20 K/min.

Details on **Single crystal X-ray diffraction (SCXRD)** measurements are provided separately in section 3 along with the crystallographic data of the obtained structures.

2. Synthetic details and characterization data

2.1. Preparation of (DMAP)$_2$PO$_2$[OTf] (1b[OTf])

2.1.1. Small-scale preparation from [(pyridine)$_2$PO$_2$][OTf] (**1a**[OTf])

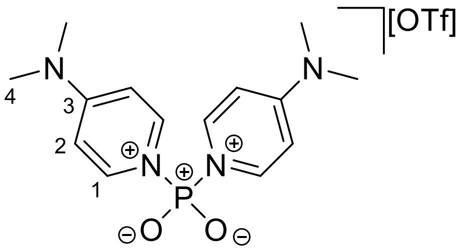

To a suspension of [(pyridine)$_2$PO$_2$][OTf] (**1a**[OTf], 3.60 g, 9.7 mmol, 1 eq.) in CH_2Cl_2 was added a solution of 4-dimethylaminopyridine (DMAP, 2.38 g, 19.4 mmol, 2 eq.) in CH_2Cl_2 and the mixture was stirred for 20 h. The colorless solid was then filtered off from the mixture, washed with CH_2Cl_2 and dried *in vacuo* to give **1b**[OTf] as a colorless powder.

The analytical data of this compound obtained via the described synthesis was previously published[1] and is here reiterated for the reader's convenience.

Yield: 4.13 g (93 %); **Raman** ($\tilde{\nu}$ in cm^{-1}): 3111 (10), 2940 (9), 1638 (23), 1585 (34), 1429 (6), 1364 (5), 1233 (8), 1113 (10), 1081 (29), 1055 (7), 1034 (36), 974 (5), 945 (20), 771 (67), 753 (11), 657 (9), 616 (7), 348 (7), 313 (9), 247 (17), 80 (100); **IR** (ATR, $\tilde{\nu}$ in cm^{-1}): 1633 (s), 1577 (w), 1514 (w), 1404 (w), 1359 (w), 1330 (w), 1264 (s), 1222 (m), 1139 (m), 1116 (s), 1084 (m), 1052 (vs), 1031 (vs), 943 (vw), 823 (m), 771 (w), 635 (s), 611 (s), 602 (vs), 572 (w), 518 (vs), 486 (vs), 446 (m); **m.p.**: 315–320 °C (decomp.); **^{1}H NMR** (CD_3NO_2, δ in ppm): 3.33 (s, 12H, H4), 6.93–6.97 (m, 4H, H2), 8.42–8.48 (m, 4H, H1); **^{13}C{^{1}H} NMR** (CD_3NO_2, δ in ppm): 39.5 (s, C4), 107.3 (d, $^{3}J_{CP}$ = 5.9 Hz, C2), 121.1 (q, $^{1}J_{CF}$ = 320.5 Hz, OTf$^-$), 141.0 (d, $^{2}J_{CP}$ = 3.8 Hz, C1), 157.6 (d, $^{4}J_{CP}$ = 1.2 Hz, C3); **^{19}F NMR** (CD_3NO_2, δ in ppm): − 79.7 (s, OTf$^-$); **^{31}P NMR** (CD_3NO_2, δ in ppm): − 15.4 (s); **elemental analysis:** calc. for $C_{15}H_{20}F_3N_4O_5PS$: C 39.5, H 4.4, N 12.3, S 7.0; found: C 39.8, H 4.6, N 12.1, S 7.0.

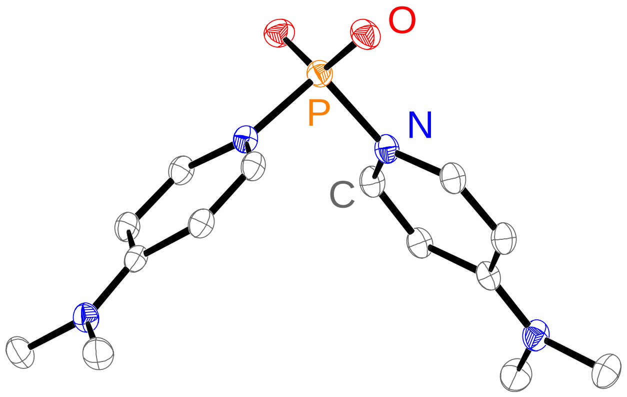

Fig. S 1. Molecular structure of **1b$^+$** in **1b**[OTf]; thermal ellipsoids are displayed at 50 % probability level; hydrogen atoms and the triflate anion are omitted for clarity. This structure was previously published[1] and is available under CCDC number 2232181.

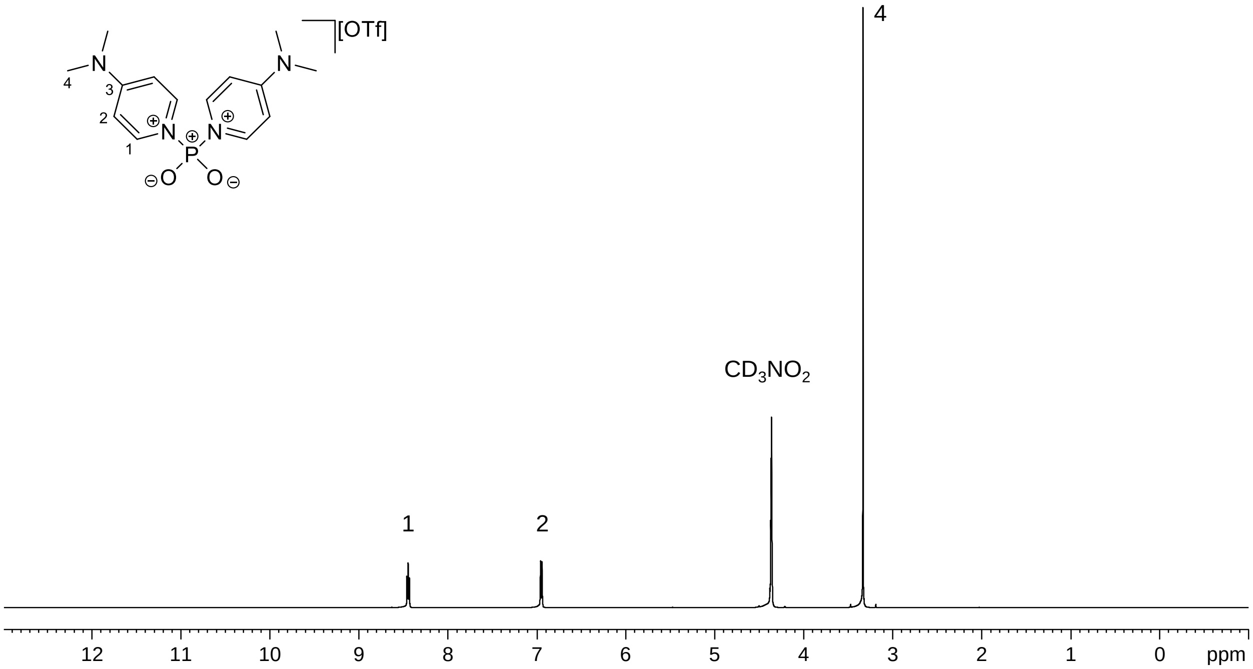

Fig. S 2. ^{1}H NMR spectrum (CD_3NO_2, 300 K) of [(DMAP)$_2$PO$_2$][OTf] (**1b**[OTf]) synthesized from **1a**[OTf].

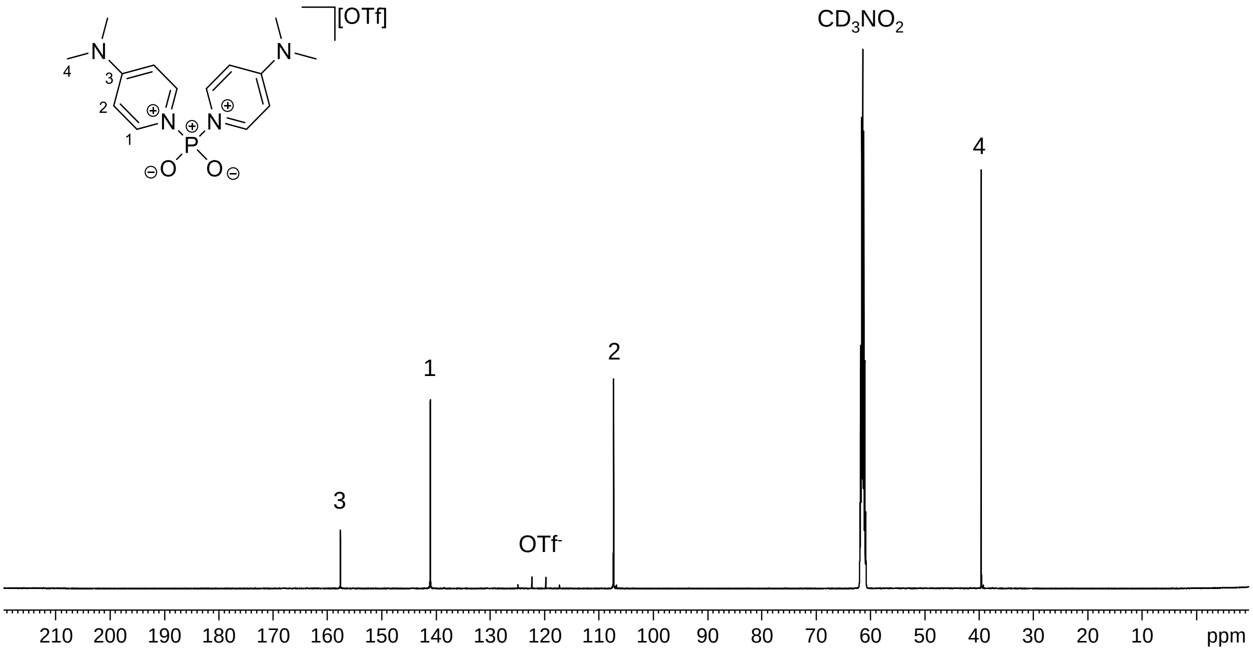

Fig. S 3. $^{13}C\{^{1}H\}$ NMR spectrum (CD_3NO_2, 300 K) of [(DMAP)$_2$PO$_2$][OTf] (**1b**[OTf]) synthesized from **1a**[OTf].

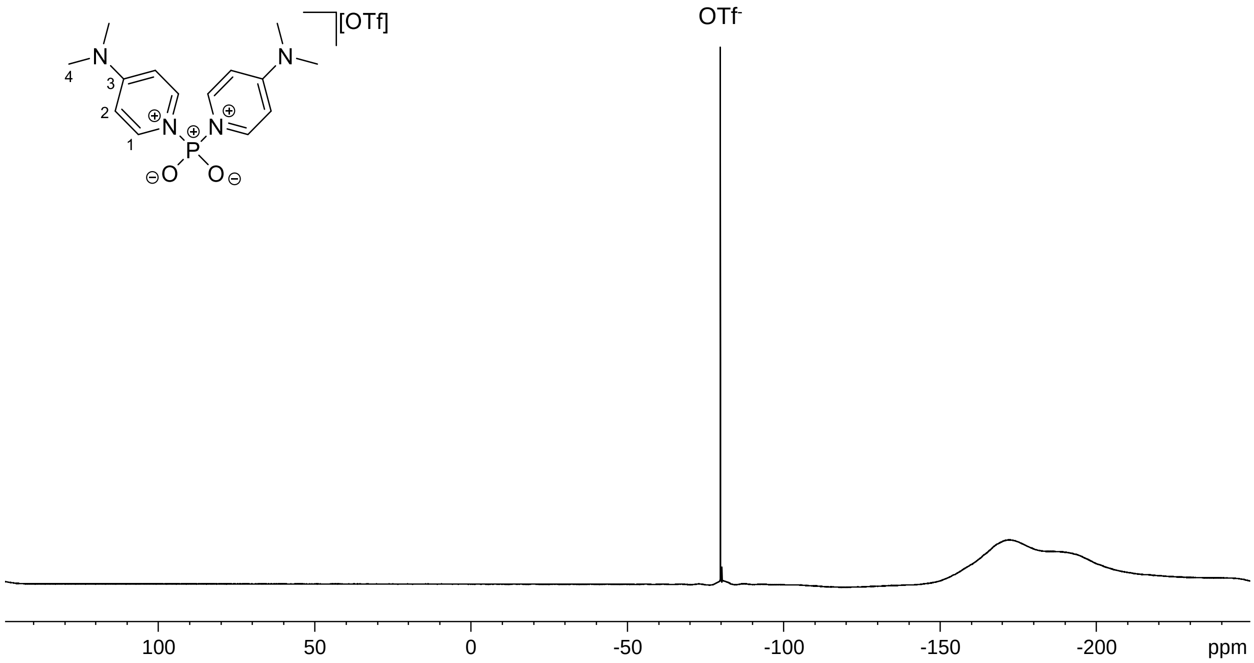

Fig. S 4. ^{19}F NMR spectrum (CD_3NO_2, 300 K) of [(DMAP)$_2$PO$_2$][OTf] (**1b**[OTf]) synthesized from **1a**[OTf].

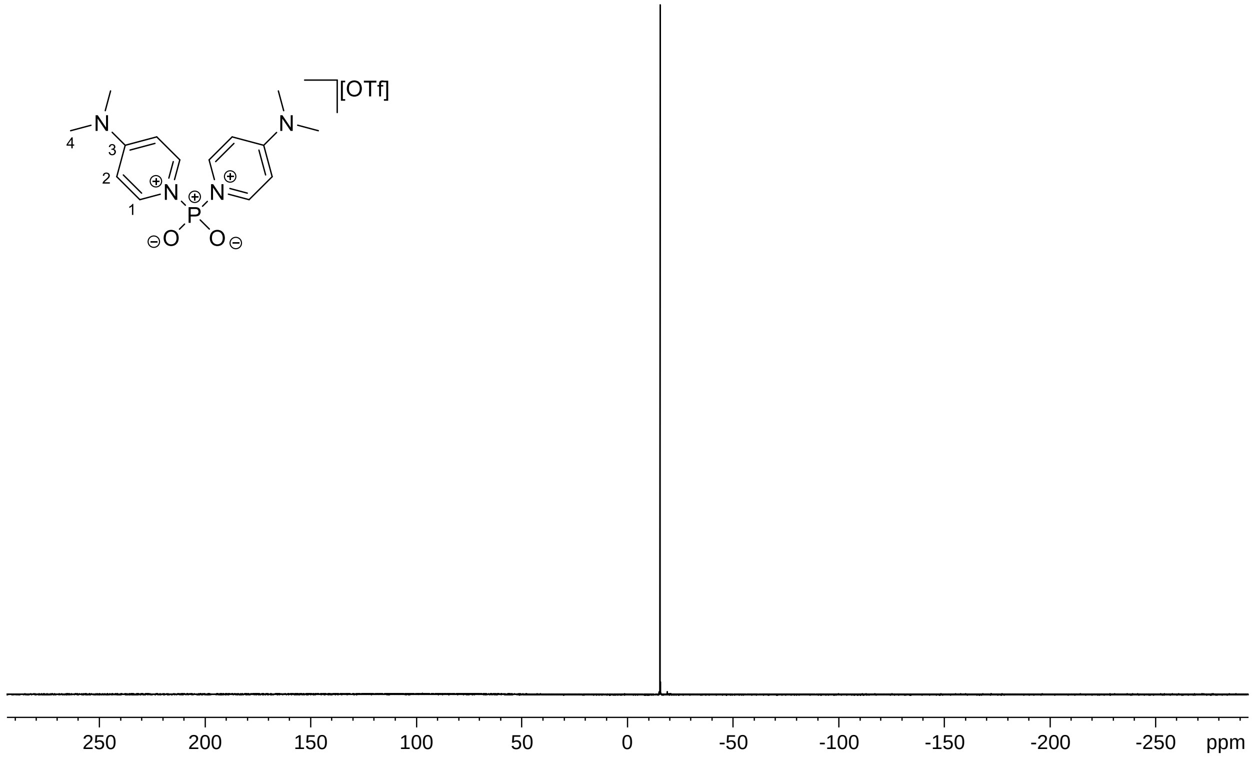

Fig. S 5. ^{31}P NMR spectrum (CD_3NO_2, 300 K) of [(DMAP)$_2$PO$_2$][OTf] (**1b**[OTf]) synthesized from **1a**[OTf].

2.1.2. Small-scale preparation from H_3PO_4

To a suspension of H_3PO_4 (980 mg, 10 mmol, 1 eq.) in 40 mL of pyridine maintained at room temperature with a water bath was slowly added triflic anhydride (6.21 g, 22 mmol, 2.2 eq.) over the course of 10 min, during which strong foaming and brown discoloration of the mixture could be observed. After further stirring the mixture for 1 h, it was heated to 45 °C for 3 days, resulting in a deep black suspension. The mixture was then cooled back to room temperature and 4-dimethylaminopyridine (DMAP, 6.72 g, 55 mmol, 5.5 eq.) was added. After further stirring the resulting mixture for 5 h, the precipitate was filtered off from the suspension, washed thoroughly with pyridine until the filtrate became colorless, and dried *in vacuo* to give **1b**[OTf] as a colorless powder.

To confirm the purity of the obtained product, ^{1}H, ^{19}F and ^{31}P NMR spectra were measured and found to be identical to the spectroscopic data described in section 2.1.1 (Fig. S 6 – Fig. S 8).

Yield: 4.47 g (98 %).

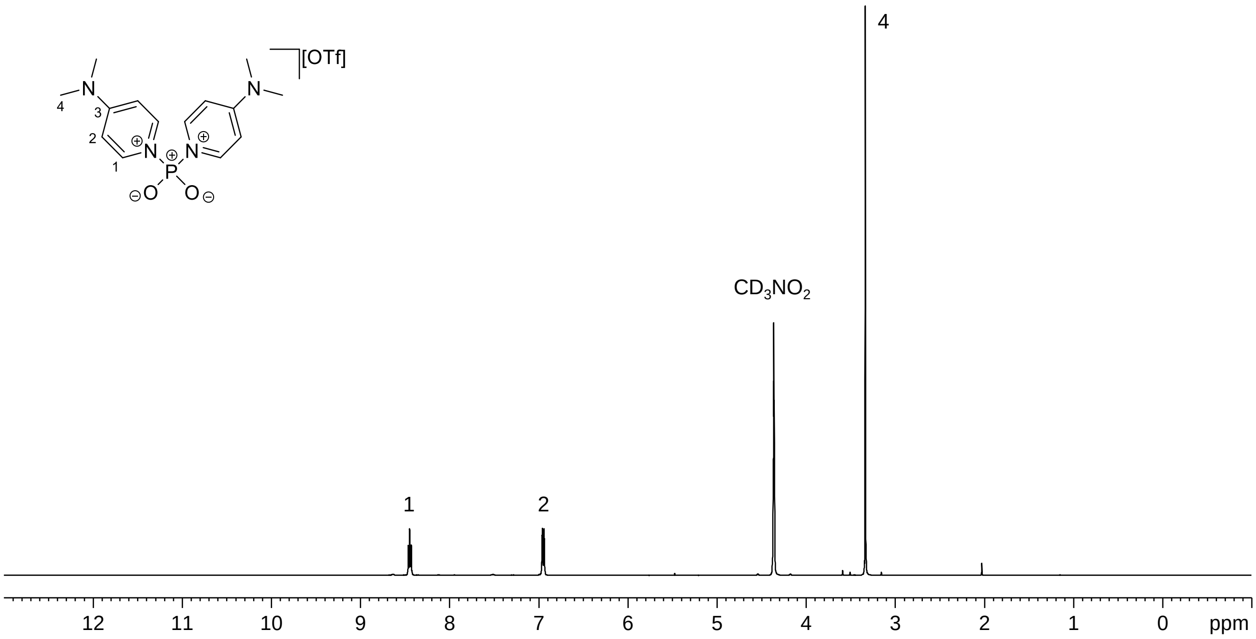

Fig. S 6. ^{1}H NMR spectrum (CD_3NO_2, 300 K) of [(DMAP)$_2$PO$_2$][OTf] (**1b**[OTf]) synthesized from H_3PO_4.

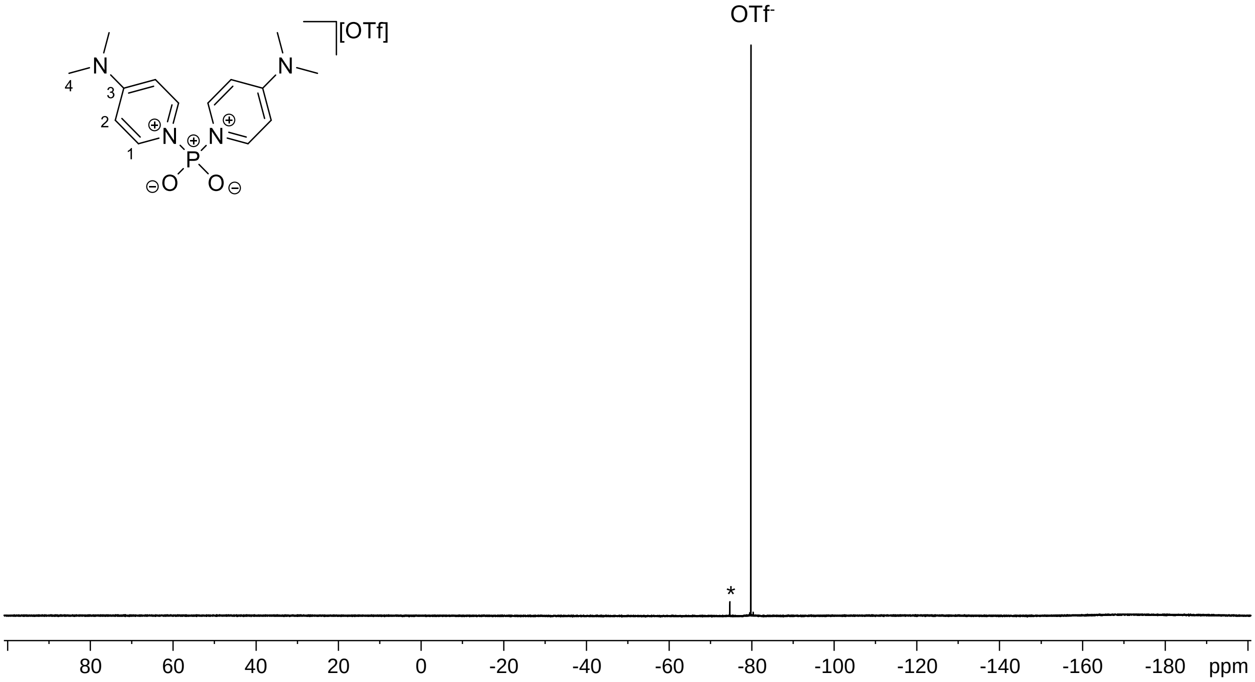

Fig. S 7. ^{19}F NMR spectrum (CD_3NO_2, 300 K) of [(DMAP)$_2$PO$_2$][OTf] (**1b**[OTf]) synthesized from H_3PO_4; Residual amounts of [Tf–DMAP][OTf] are marked with asterisks (*).

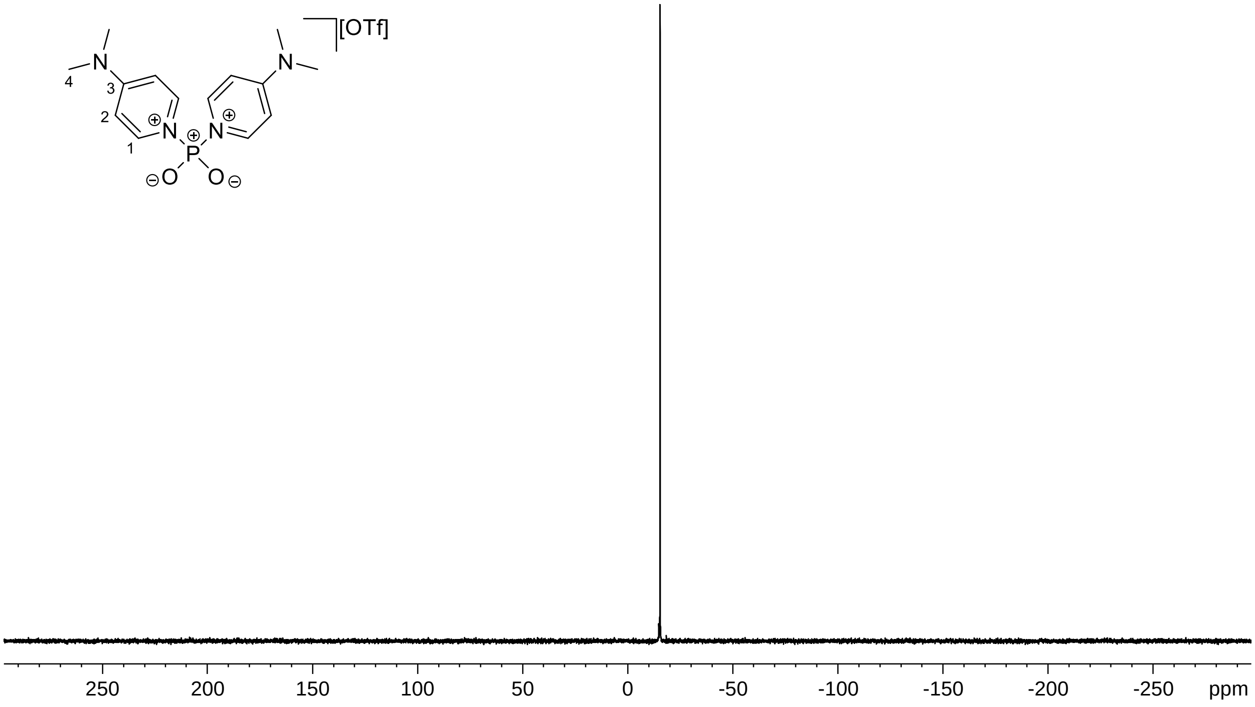

Fig. S 8. ^{31}P NMR spectrum (CD_3NO_2, 300 K) of [(DMAP)$_2$PO$_2$][OTf] (**1b**[OTf]) synthesized from H_3PO_4.

2.1.3. Large-scale preparation from P_2O_5

To a 4L three-necked round-bottom flask equipped with an overhead stirrer and a reflux condenser, 3 L of pyridine dried over 4 Å molecular sieve were first added followed by P_2O_5 (70.97 g, 0.5 mol, 1 eq.) via a solid addition funnel under vigorous stirring (300 rpm). The resulting suspension was then slowly brought to a boil, increasing the temperature by about 10 K every 30 minutes, which leads to a pronounced increase in turbidity and yellow discoloration of the mixture *(Note: It was repeatedly observed that rapid heating of the suspension temporarily resulted in a clear solution before formation of very firm, spherical chunks of precipitate. Not only did this inhibit the desired reaction due to a passivation effect of the outer surface, their size of up to 5 cm of diameter also causes a serious safety hazard as they could potentially break the glass walls of the flask. Therefore, slow heating is of paramount importance)*. After 16 h of reflux and cooling back to room temperature, the flask was fitted with a dropping funnel and triflic anhydride (155.17 g, 0.55 mol, 1.1 eq.) was added slowly over the course of 30 minutes under cooling in an ice bath. The ice bath was removed and the mixture further stirred for 1 h at room temperature and subsequently at 45 °C for 3 days, resulting in a black-colored suspension with large amounts of precipitate. To the mixture was then added 4-dimethylaminopyridine (268.78 g, 2.2 mol, 4.4 eq.), leading to a light brown suspension. After further stirring the mixture at 45 °C for 16 h, the precipitate was filtered off from the suspension, washed thoroughly with pyridine until the filtrate became colorless, and dried *in vacuo* to give **1b**[OTf] as a colorless powder.
To confirm the purity of the obtained product, ^{1}H, ^{19}F and ^{31}P NMR spectra were measured and found to be identical to the spectroscopic data described in section 2.1.1 (Fig. S 10 – Fig. S 12).
Yield: 456.38 g (97 %).

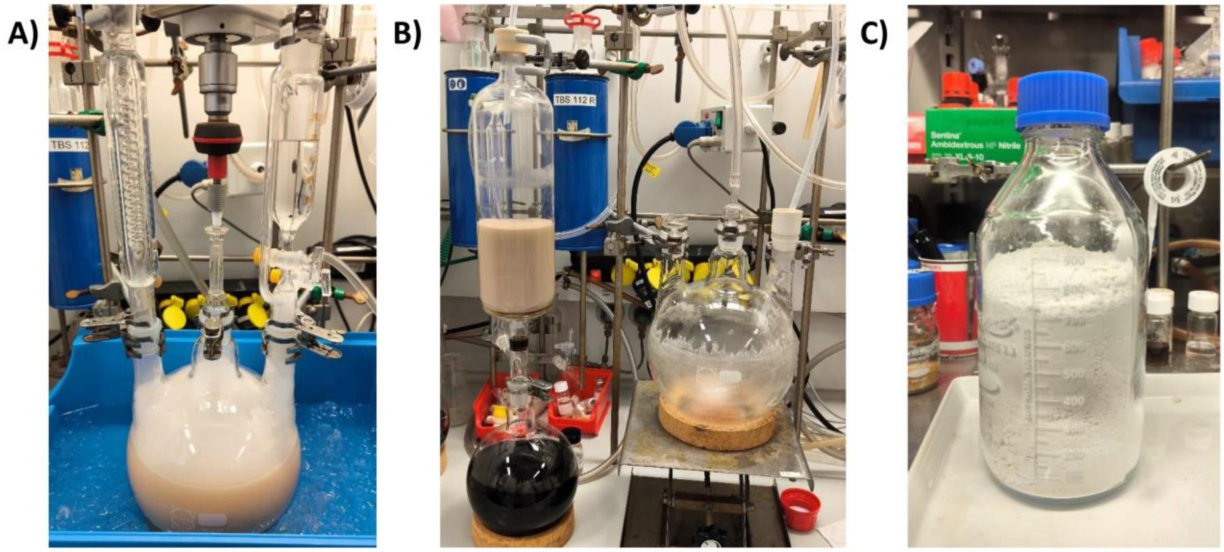

Fig. S 9. Image documentation of the synthesis of **1b**[OTf] from P_2O_5 on a molar scale; **A)** suspension of P_2O_5 during slow addition of Tf_2O; **B)** work-up via filtration after reaction is completed; **C)** bottled product after drying.

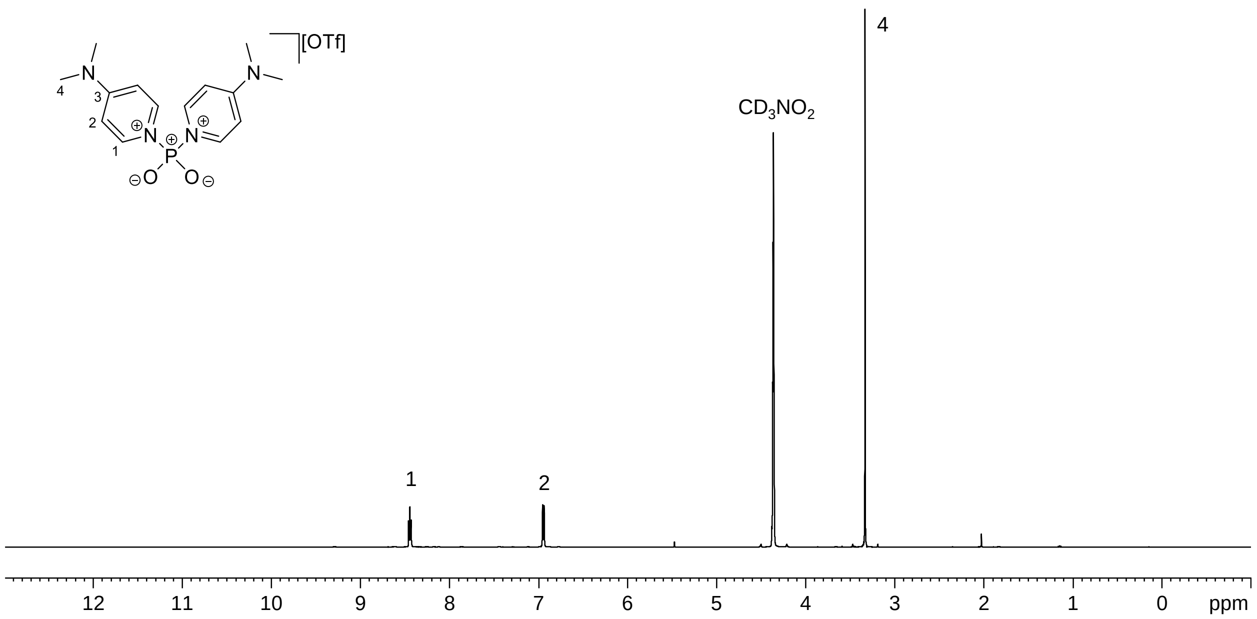

Fig. S 10. ^{1}H NMR spectrum (CD_3NO_2, 300 K) of [$(DMAP)_2PO_2$][OTf] (**1b**[OTf]) synthesized from P_4O_{10}.

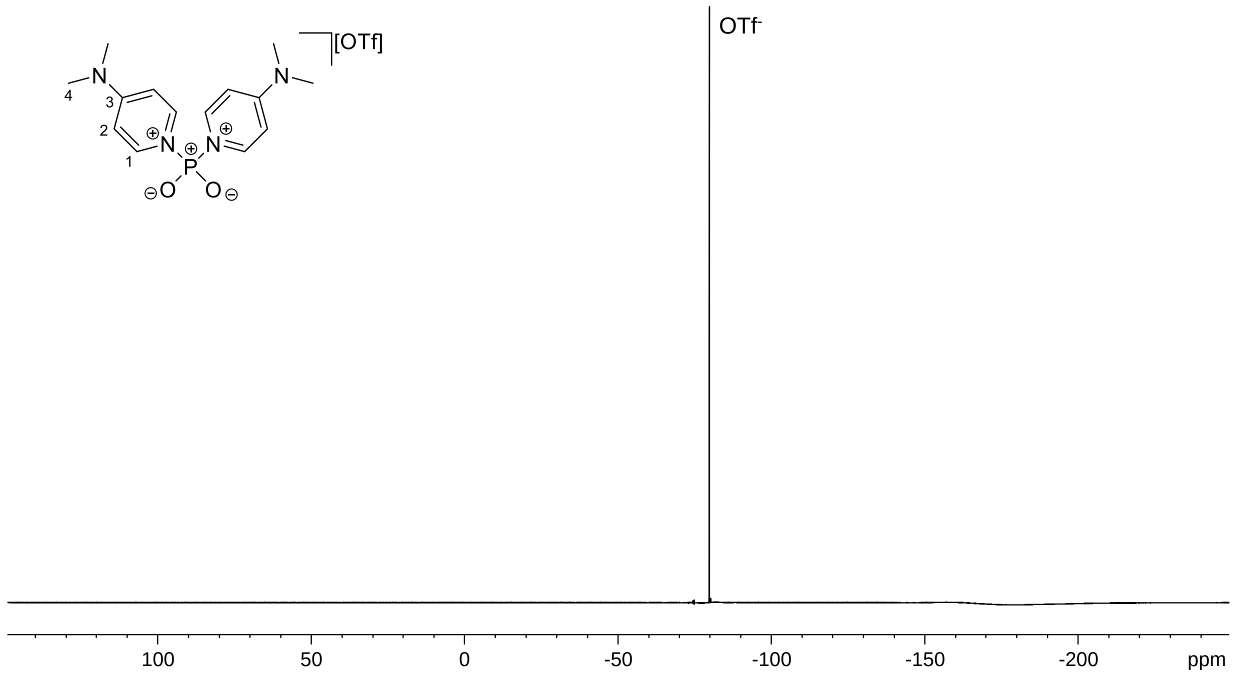

Fig. S 11. ^{19}F NMR spectrum (CD_3NO_2, 300 K) of [$(DMAP)_2PO_2$][OTf] (**1b**[OTf]) synthesized from P_4O_{10}.

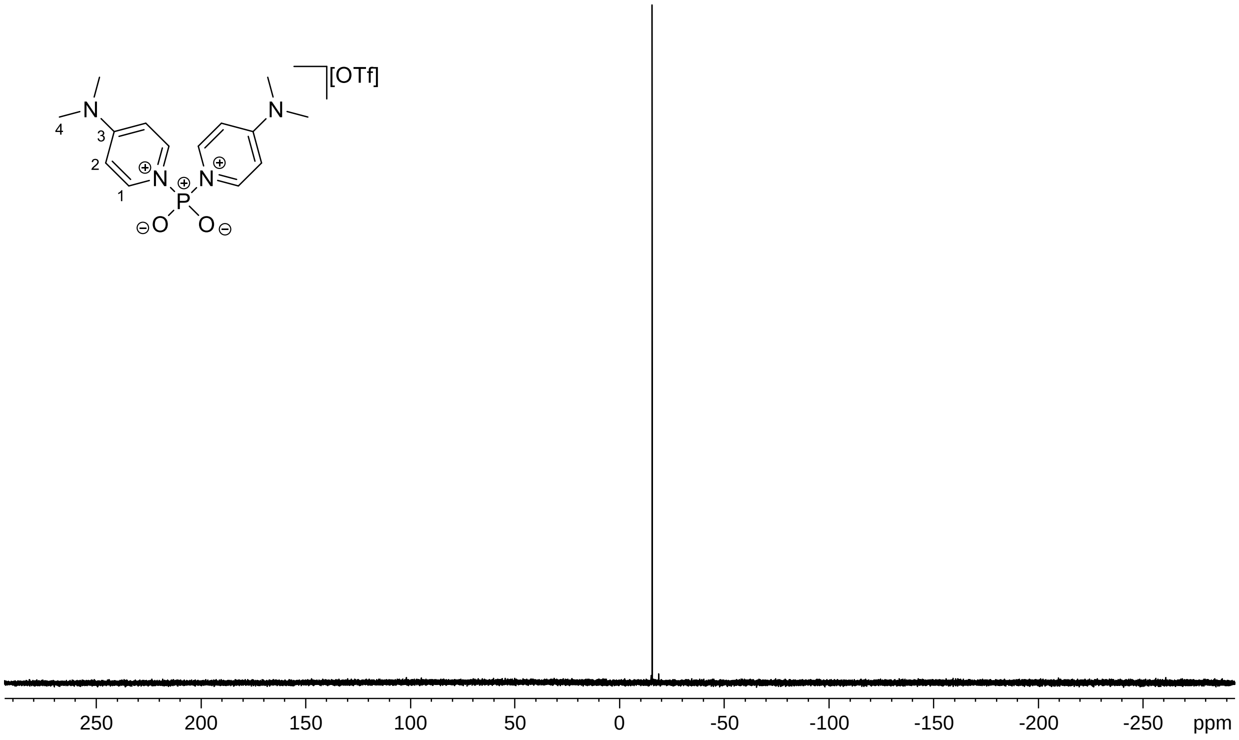

Fig. S 12. ^{31}P NMR spectrum (CD_3NO_2, 300 K) of [(DMAP)$_2$PO$_2$][OTf] (**1b**[OTf]) synthesized from P_4O_{10}.

2.2. Reactivity studies

2.2.1. Reaction of [(pyridine)$_2$PO$_2$][OTf] (**1a**[OTf]) with tetrabutylammonium chloride ([TBA][Cl])

To a stirred solution of tetrabutylammonium chloride ([TBA][Cl], 80 mg, 0.28 mmol, 2 eq.) in CH_3CN (1.5 mL) was slowly added [(pyridine)$_2$PO$_2$][OTf] (**1a**[OTf], 53 mg, 0.14 mmol, 1 eq.) in CH_3CN (0.5 mL). After stirring the mixture for 2 hours, an aliquot was taken for NMR investigation. The ^{31}P NMR spectrum reveals clean formation of the "PO_2^+" transfer reagent $[Cl_2PO_2]^-$ as indicated by a singlet resonance at $\delta(^{31}P) = -8.2$ ppm (Fig. S 13).

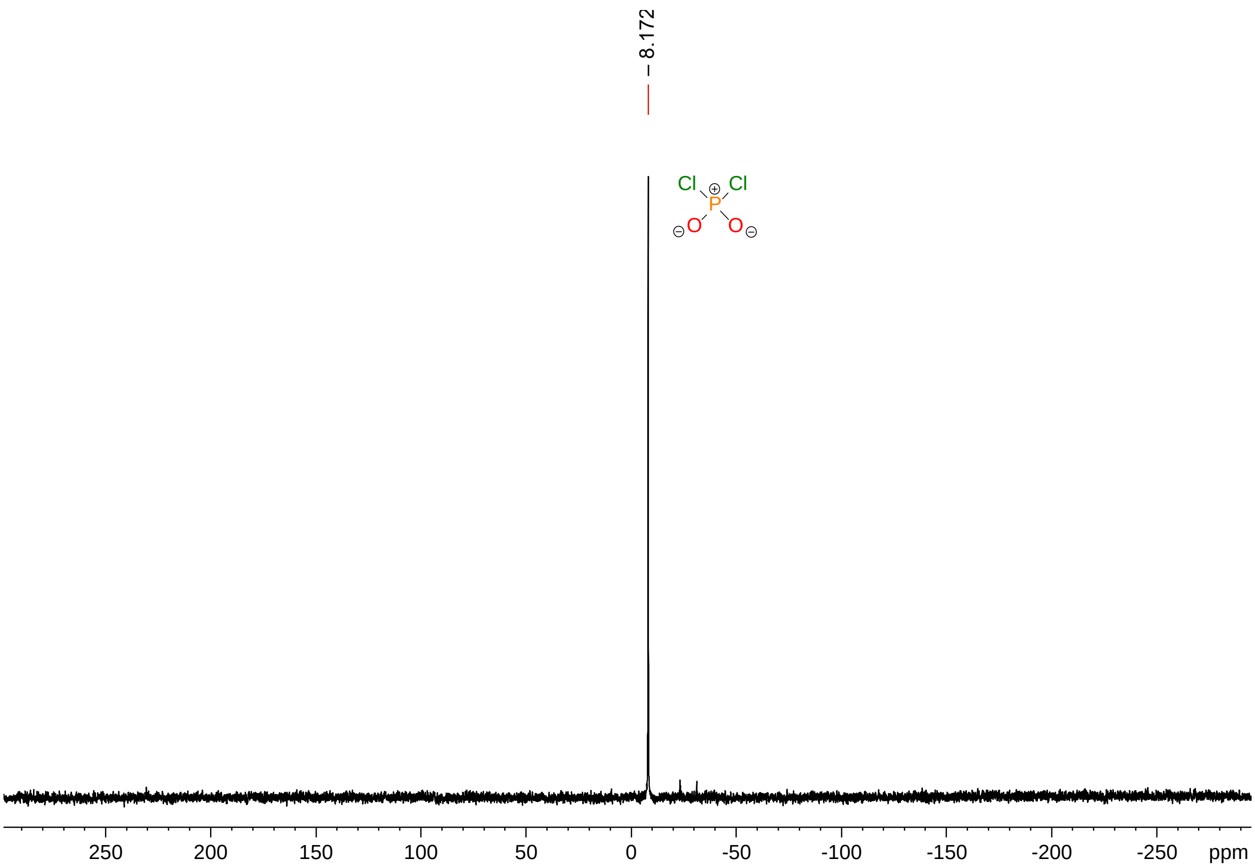

Fig. S 13. ^{31}P NMR spectrum (CH_3CN, C_6D_6-capillary, 300 K) of the reaction mixture of **1a**[OTf] with 2 eq. [TBA][Cl].

2.2.2. Reaction of [(pyridine)$_2$PO$_2$][OTf] (**1a**[OTf]) with phenylmagnesium bromide

To a stirred solution of phenylmagnesium bromide (1 M in THF, 2 mL, 2 mmol, 2 eq.) in Et_2O was slowly added [(pyridine)$_2$PO$_2$][OTf] (**1a**[OTf], 370 mg, 1 mmol, 1 eq.), resulting in slow dissolution of **1a**[OTf]. After stirring the mixture for 2 hours, it was quenched with 1 M HCl which caused large amounts of an orange-colored precipitate to separate from the two-phase mixture. The precipitate was immediately filtered off and dried vigorously *in vacuo* over P_2O_5 for 16 hours to obtain 413 mg of an orange-colored powder.

The obtained sample was found to be poorly soluble in common organic solvents (CH_2Cl_2, CH_3CN, CH_3NO_2) and water. Full dissolution was achieved in DMF and DMSO, which afforded the ^{31}P NMR spectra depicted in Fig. S 14. In DMF, very broad resonances in the range of – 30 to + 15 ppm are the only signals observable, which comprises literature-reported values of condensed oligophosphates[2] and poly(arylphosphonates)[3]. In DMSO, a significant decrease of the polymeric resonance is accompanied by two new resonances identified as phosphoric acid (**13**, δ(^{31}P) = – 1.2 ppm, s) and phenylphosphonic acid (**14**, δ(^{31}P) = 12.6 ppm, t, $^3J_{PH}$ = 13.2 Hz)[4], indicating solvent-induced depolymerization.

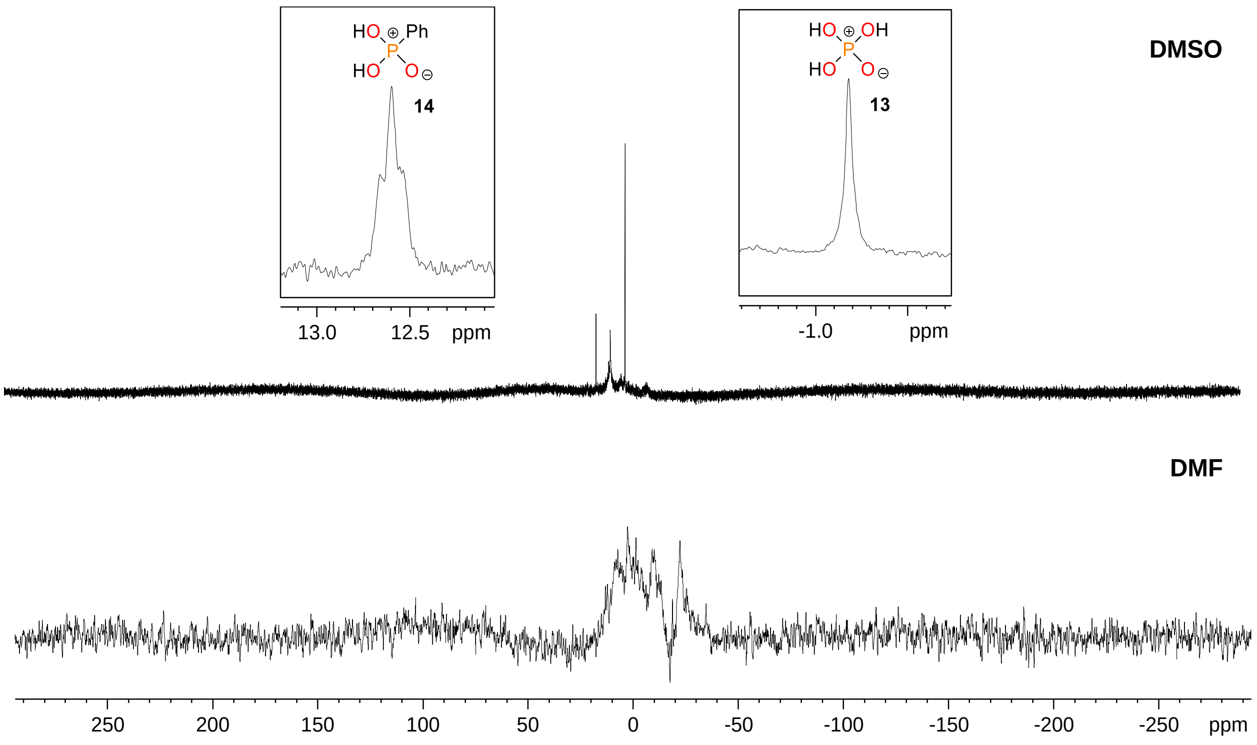

Fig. S 14. ^{31}P NMR spectra (DMF / DMSO, C_6D_6-capillary, 300 K) of the polymeric sample obtained from reaction of **1a**[OTf] with PhMgBr after acidic aqueous quench and drying.

Based on this, it is assumed that the primarily formed intermediate **3** acts as an initiator to copolymerization of itself and remaining **1a**$^+$ as monomeric units, yielding linear or cyclic products of types **5** and **6**, respectively (Fig. S 15). Upon acidic aqueous quench, remaining pyridyl-groups are hydrolyzed to give copolymers of types **11** or **12**, which is supported by the ^{31}P NMR spectra shown in Fig. S 14. Further hydrolysis of the sample could be observed when the polymeric precipitate was not separated after acidic aqueous quench. After continuing to stir the mixture for 2 days, most of the solid had dissolved and the ^{31}P NMR spectrum of the aqueous phase (Fig. S 16) shows formation of phosphoric acid (**13**, δ(^{31}P) = – 0.3 ppm, s) and phenylphosphonic acid (**14**, δ(^{31}P) = 17.0 ppm, t, $^3J_{PH}$ = 13.2 Hz)[4] arising from hydrolytic chain degradation of **11** or **12** as depicted in Fig. S 15.

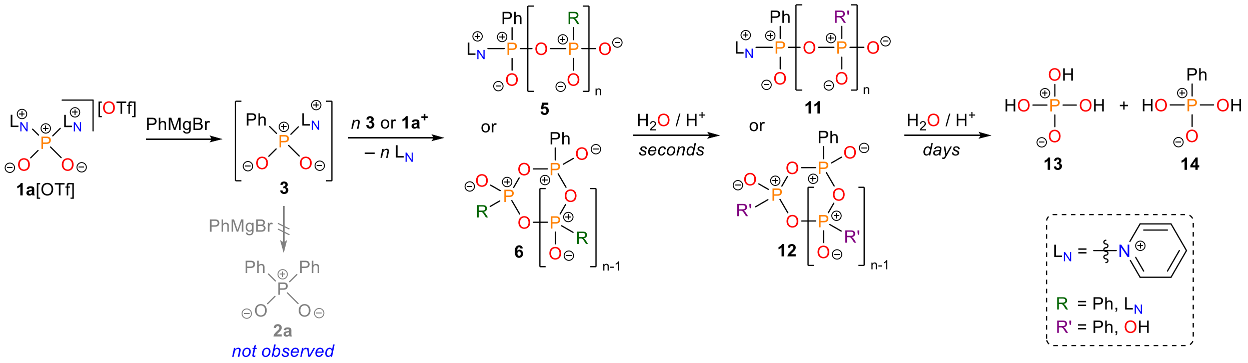

Fig. S 15. Proposed mechanism for oligomerization side reaction during attempted reaction of **1a**[OTf] with phenylmagnesium bromide and subsequent hydrolytic degradation, competitively suppressing formation of **2a**.

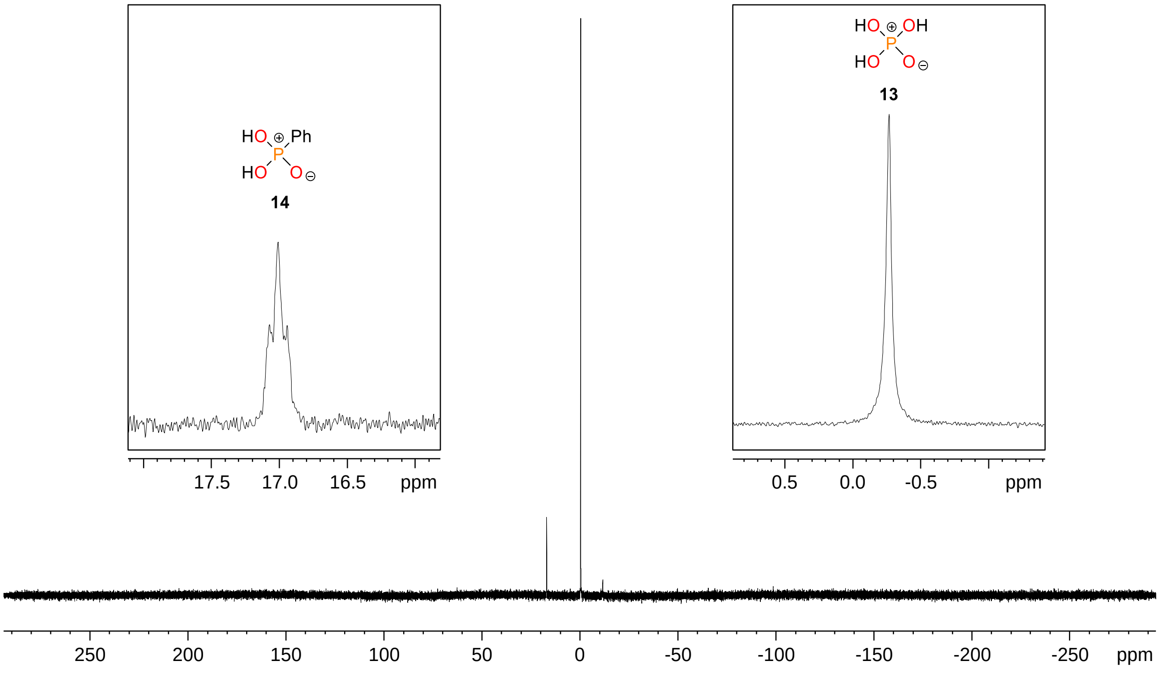

Fig. S 16. ^{31}P NMR spectra (H_2O, C_6D_6-capillary, 300 K) of the polymeric sample obtained from reaction of **1a**[OTf] with PhMgBr after acidic aqueous quench and hydrolytic digestion for 2 days.

2.2.3. Reaction of [(pyridine)$_2$PO$_2$][OTf] (**1a**[OTf]) with *p*-nitrophenol

To a stirred suspension of [(pyridine)$_2$PO$_2$][OTf] (**1a**[OTf], 93 mg, 0.25 mmol, 1 eq.) in pyridine was added *p*-nitrophenol (35 mg, 0.25 mmol, 1 eq.), resulting in formation of a clear solution within 5 minutes of stirring. After stirring the mixture for 30 minutes, an aliquot of the solution was taken and investigated by means of ^{31}P{^{1}H} NMR spectroscopy with a C_6D_6-capillary (Fig. S 19). The spectrum shows a singlet resonance at δ(^{31}P) = – 11.5 ppm as the main product, indicating the formation and stability in solution of betain **7b**. As a minor product, an AX-spinsystem (δ(A) = – 23.7 ppm (d), δ(X) = – 17.7 ppm (d), $^2J_{PP}$ = 18.5 Hz) was identified and tentatively assigned to the diphosphate **8b** arising from condensation of **7b** under liberation of pyridine. From this mixture, single crystals of **7b** suitable for single crystal structure determination were obtained by vapor diffusion of Et_2O into the reaction mixture at – 30 °C (Fig. S 18). All attempts to isolate **7b** from co-crystallized [HPy][OTf] failed and resulted in partial decomposition of the compound.

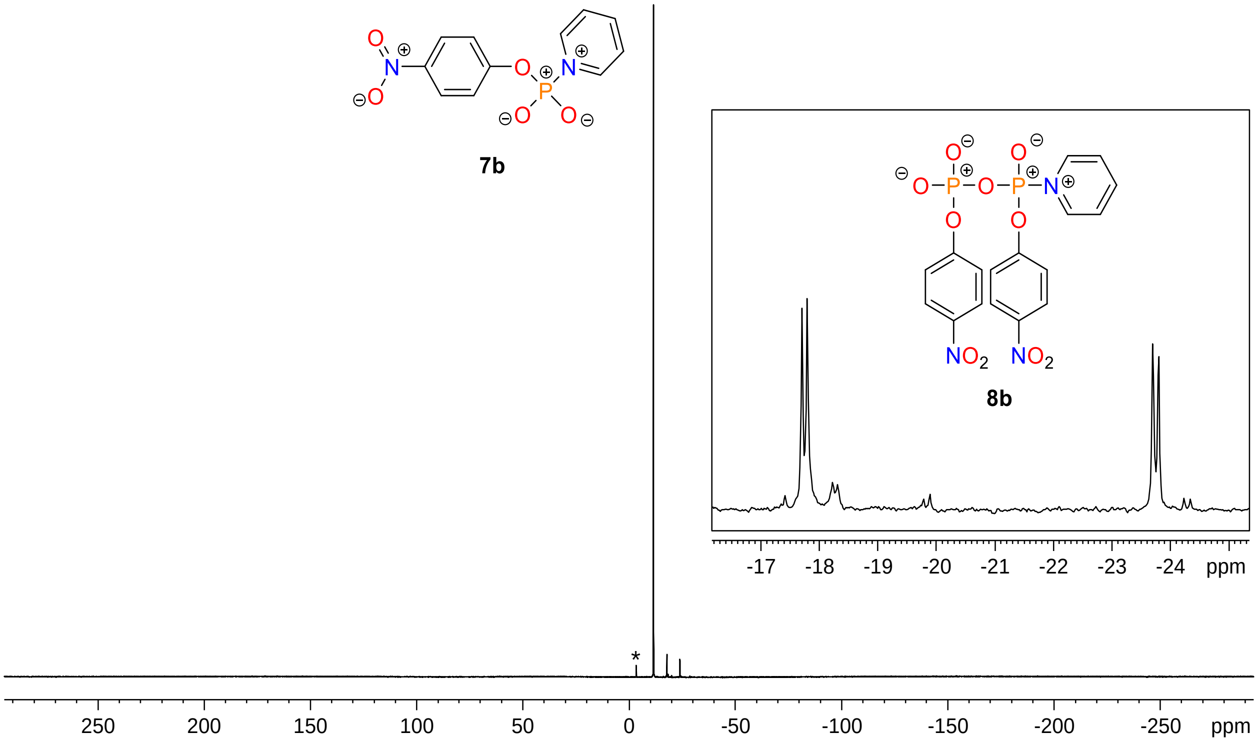

Fig. S 17. ^{31}P{^{1}H} NMR spectrum (pyridine, C_6D_6-capillary, 300 K) of the reaction of **1a**[OTf] with *p*-nitrophenol. The main product (δ(^{31}P) = – 11.5 ppm) was tentatively identified as betain **7b**. An AX-spinsystem (δ(A) = – 23.7 ppm (d), δ(X) = – 17.7 ppm (d), $^2J_{PP}$ = 18.5 Hz) indicates the minor formation of condensation product **8b**. Other, unidentified minor products are marked with asterisks (*).

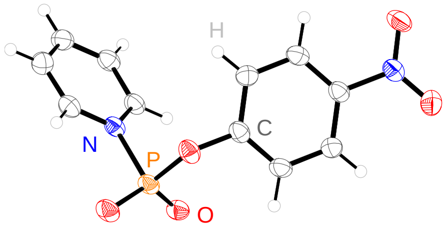

Fig. S 18. Molecular structure of betain **7b**; thermal ellipsoids are displayed at 50 % probability level; CCDC 2442189.

2.2.4. Reaction of [(pyridine)$_2$PO$_2$][OTf] (**1a**[OTf]) with phenol

To a stirred suspension of [(pyridine)$_2$PO$_2$][OTf] (**1a**[OTf], 116 mg, 0.31 mmol, 1 eq.) in pyridine was added phenol (29 mg, 0.31 mmol, 1 eq.), resulting in formation of a clear solution within 5 minutes of stirring. After stirring the mixture for 30 minutes, an aliquot of the solution was taken and investigated by means of $^{31}P\{^{1}H\}$ NMR spectroscopy with a C_6D_6-capillary (Fig. S 19). The spectrum shows a singlet resonance at $\delta(^{31}P) = -9.7$ ppm as the main product, indicating the formation and stability in solution of betain **7c**. As a minor product, an AX-spinsystem (δ(A) = – 21.8 ppm (d), δ(X) = – 16.6 ppm (d), $^{2}J_{PP}$ = 17.1 Hz) was identified and tentatively assigned to the diphosphate **8c** arising from condensation of **7c** under liberation of pyridine. All attempts to isolate or crystallize **7c** from the mixture failed.

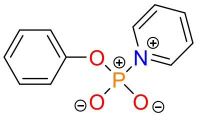

7c

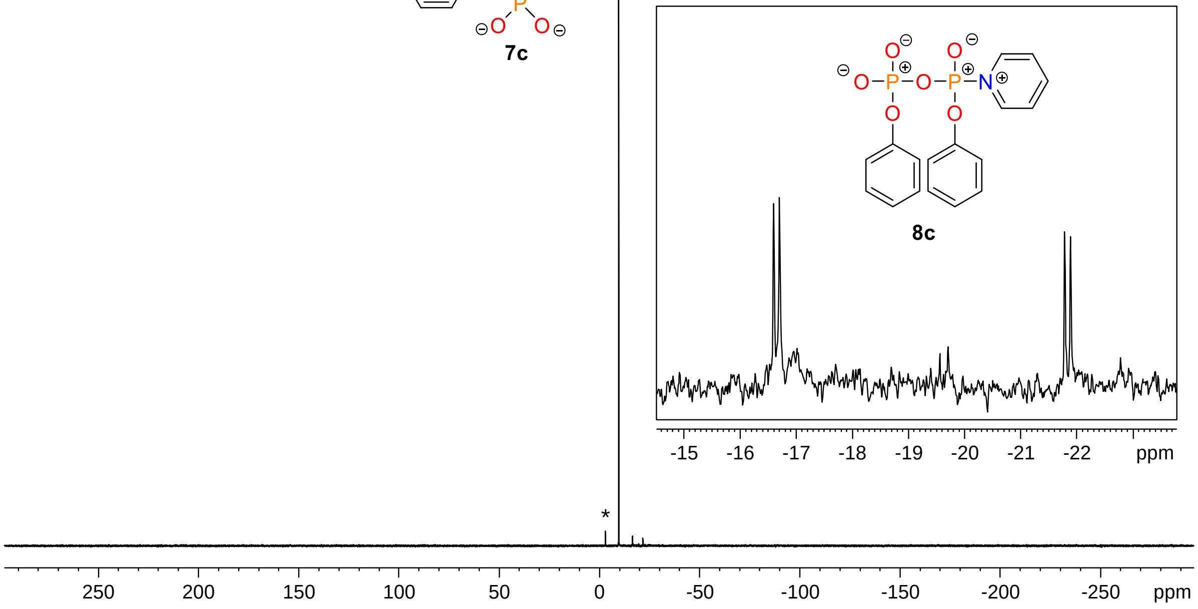

Fig. S 19. $^{31}P\{^{1}H\}$ NMR spectrum (pyridine, C_6D_6-capillary, 300 K) of the reaction of **1a**[OTf] with phenol. The main product ($\delta(^{31}P)$ = – 9.7 ppm) was tentatively identified as betain **7c**. An AX-spinsystem (δ(A) = – 21.8 ppm (d), δ(X) = – 16.6 ppm (d), $^{2}J_{PP}$ = 17.1 Hz) indicates the minor formation of condensation product **8c**. Other, unidentified minor products are marked with asterisks (*).

2.2.5. Reaction of [(DMAP)$_2$PO$_2$][OTf] (**1b**[OTf]) with 2-mesitylmagnesium bromide

To a stirred solution of 2-mesitylmagnesium bromide (1 M in in THF, 0.55 mL, 0.55 mmol, 2.2 eq.) in Et_2O was slowly added [(DMAP)$_2$PO$_2$][OTf] (**1b**[OTf], 114 mg, 0.25 mmol, 1 eq.), resulting in deep brown discoloration and formation of a wax-like solid. After stirring the mixture for 2 hours, it was quenched with 1 M HCl to obtain a clear two-phase mixture. Both phases were then investigated by means of ^{31}P NMR spectroscopy. In the organic phase, no signals were observed. In the aqueous phase, two singlet resonances at δ(^{31}P) = – 0.5 ppm and δ(^{31}P) = – 5.9 ppm were observed as the main products and identified as phosphoric acid and diphosphoric acid, respectively, arising from hydrolysis of the starting material (Fig. S 20). The spectrum did not show any indication for formation of arylphosphonic or diarylphosphinic acids.

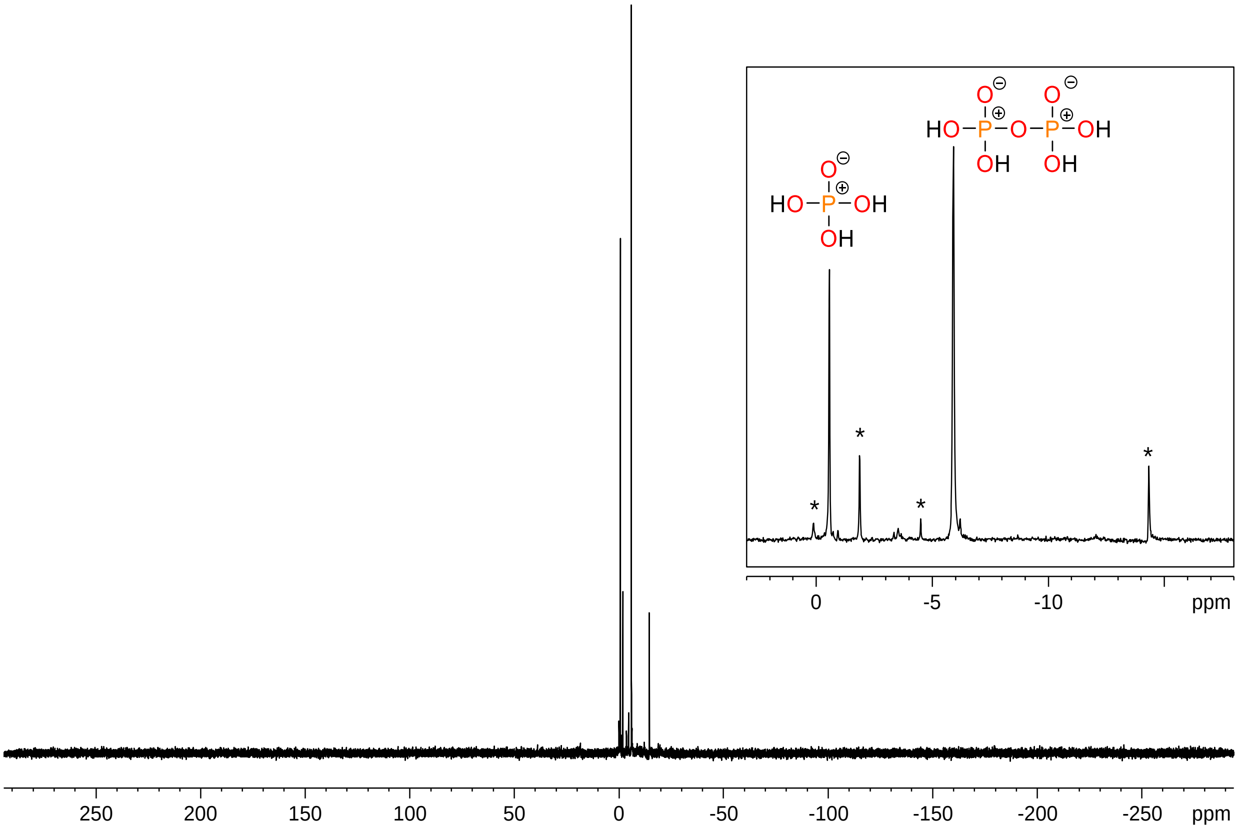

Fig. S 20. ^{31}P NMR spectrum (H_2O / D_2O, 300 K) of the reaction of **1a**[OTf] with 2-mesitylmagnesium bromide after quenching with 1 M HCl (aqueous phase). Two singlet resonances at δ(^{31}P) = – 0.5 ppm and δ(^{31}P) = – 5.9 ppm were identified as the main products phosphoric acid and diphosphoric acid, respectively. Other, unidentified minor products are marked with asterisks (*).

2.2.6. Reaction of [(DMAP)$_2$PO$_2$][OTf] (**1b**[OTf]) with alkyl Grignard reagents H$_n$(Me)$_{3-n}$C–MgX

To a series of stirred solutions of alkylgrignard reagents (0.5 mmol, 2 eq.) according to Table S 1 in Et$_2$O was given [(DMAP)$_2$PO$_2$][OTf] (**1b**[OTf], 114 mg, 0.25 mmol, 1 eq.) and the obtained suspensions were continued to stir for 16 h. The reaction mixtures were quenched with 1 M HCl, resulting in formation of clear two-phase mixtures. Both phases were then investigated by means of ^{31}P NMR spectroscopy. In the organic phase, no signals were observed in the ^{31}P NMR spectrum for all of the tested alkylation reactions. The ^{31}P NMR spectra of the aqueous phases are depicted in Fig. S 21.

Table S 1. Details on used Grignard reagents for rection study of **1b**[OTf] with alkyl-Grignard reagents.

Grignard reagent	solvent	concentration	volume
MeMgBr	Bu$_2$O	1 M	0.5 mL
EtMgBr	Et$_2$O	3 M	0.17 mL
^{i}PrMgCl	THF	2 M	0.25 mL
^{t}BuMgCl	THF	2 M	0.25 mL

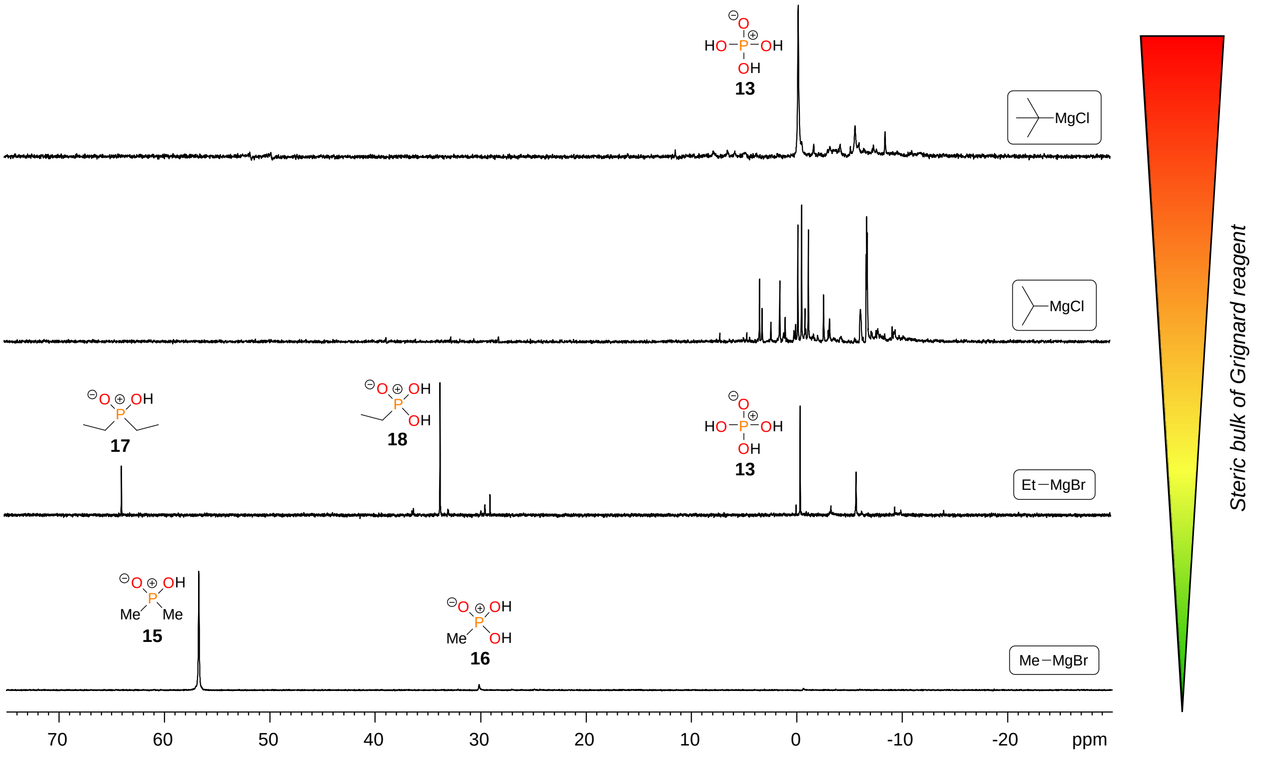

Fig. S 21. ^{31}P{^{1}H} NMR spectra (H$_2$O, C$_6$D$_6$-cappilary, 300 K) from reaction studies of **1b**[OTf] with 2 equivalents of Grignard reagents (methyl-, ethyl-, *iso*-propylmagnesium chloride and *tert*-butylmagnesium chloride) imposing variable steric bulk.

The obtained NMR spectra demonstrate that only methylmagnesium bromide is able to form the corresponding dimethylphosphinic acid (**15**, δ(^{31}P) = 56.7 ppm, sept, $^{2}J_{PH}$ = 14.1 Hz) in high selectivity of 93 % as determined by integration of all signals in the ^{31}P NMR spectrum, with methylphosphonic acid (**16**, δ(^{31}P) = 30.1 ppm, q, $^{2}J_{PH}$ = 14.7 Hz) being the only detectable side product.[5] For ethylmagnesium bromide, the reaction's selectivity already decreases significantly, forming diethylphosphinic acid (**17**, δ(^{31}P) = 63.8 ppm, m)[6] with less than 10 % selectivity alongside ethylphosphonic acid (**18**, δ(^{31}P) = 33.6 ppm, *pseudo*-sxt, $^{2}J_{PH} \approx {}^{3}J_{PH} \approx$ 19.4 Hz)[7] and phosphoric acid (**13**, δ(^{31}P) = – 0.5 ppm, s) as the main products. Following this trend, both ^{i}PrMgCl and ^{t}BuMgCl did not form the corresponding phosphinic acid after reaction with **1b**[OTf]. While the former displays multiple unidentified products in the ^{31}P{^{1}H} NMR spectrum ranging from – 10 to + 7 ppm, indicating oligomerization after single substitution similar to the reactivity of **1a**[OTf], the latter mainly displays a singlet resonance at δ(^{31}P) = – 0.4 ppm identified as phosphoric acid (**13**). This suggests that for ^{t}BuMgBr, even the first substitution step with **1b^{+}** is strongly sterically hindered, resulting in most of the starting material hydrolyzing under liberation of DMAP during the quenching step.

2.2.7. Reaction of [(DMAP)$_2$PO$_2$][OTf] (**1b**[OTf]) with other incompatible Grignard reagents

In a typical experiment, [(DMAP)$_2$PO$_2$][OTf] (**1b**[OTf], 114 mg, 0.25 mmol, 1 eq.) was added to a solution of the Grignard reagent (0.50 – 0.55 mmol, 2.0 – 2.5 eq.) in either Et_2O or THF. After stirring the mixture for 4 – 16 h, it was quenched with 1 M HCl and the resulting phases were investigated by means of ^{31}P NMR spectroscopy. Table S 2 gives a summary of the results for all substrates not explicitly discussed in the main text, where no further isolation of the products was attempted.

Table S 2. Reactivity of [(DMAP)$_2$PO$_2$][OTf] (**1b**[OTf]) with Grignard reagents resulting in unsatisfactory reaction outcomes.

Grignard reagent	solvent	eq.	notes
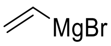	Et_2O	2.2	Formation of target phopshinic acid observed, but no isolation attempted due to low selectivity (12 % by integration of all signals in ^{31}P NMR spectrum, Fig. S 22).
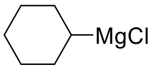	Et_2O	2.0	No formation of the target phosphinic acid observed (Fig. S 23).
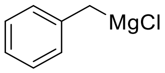	THF	2.5	No formation of the target phosphinic acid observed (Fig. S 24).
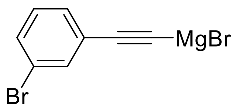	THF	2.5	Formation of target phosphinic acid observed, but no isolation attempted due to dominant Grignard metathesis with bromoaryl-group (Fig. S 25).
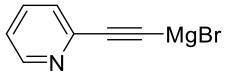	THF	2.5	No formation of the target phosphinic acid observed (Fig. S 26).

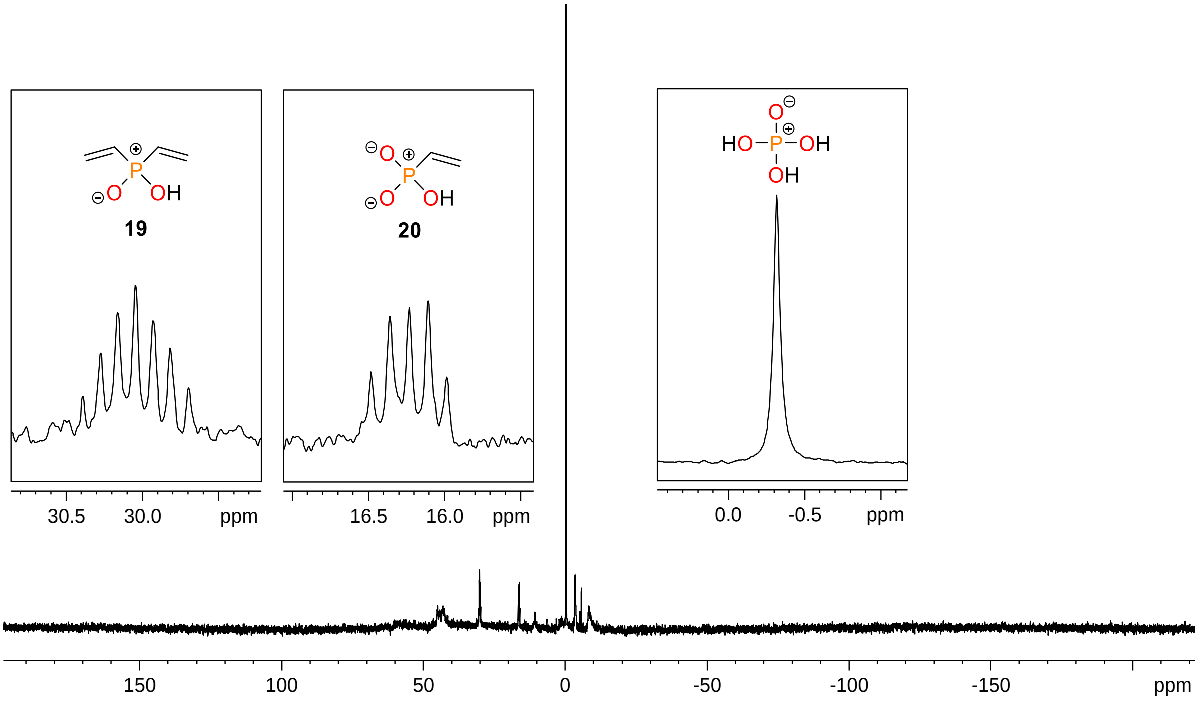

Fig. S 22. ^{31}P NMR spectrum (H_2O, C_6D_6-capillary., 300 K) of reaction of **1b**[OTf] with vinylmagnesium bromide after quenching with 1 M HCl (aqueous, no signals in organic phase); The three main signals were tentatively assigned as divinylphosphinic acid (**19**, $\delta(^{31}P)$ = 33.6 ppm, *pseudo*-non, $^3J_{PH, trans}$ = 46.4 Hz, $^2J_{PH} \approx {}^3J_{PH, cis} \approx$ 23.2 Hz), vinylphosphonic acid (**20**, $\delta(^{31}P)$ = 16.2 ppm, dt, $^3J_{PH, trans}$ = 50.0 Hz, $^2J_{PH} \approx {}^3J_{PH, cis} \approx$ 25.0 Hz) and phosphoric acid ($\delta(^{31}P)$ = – 0.3 ppm).

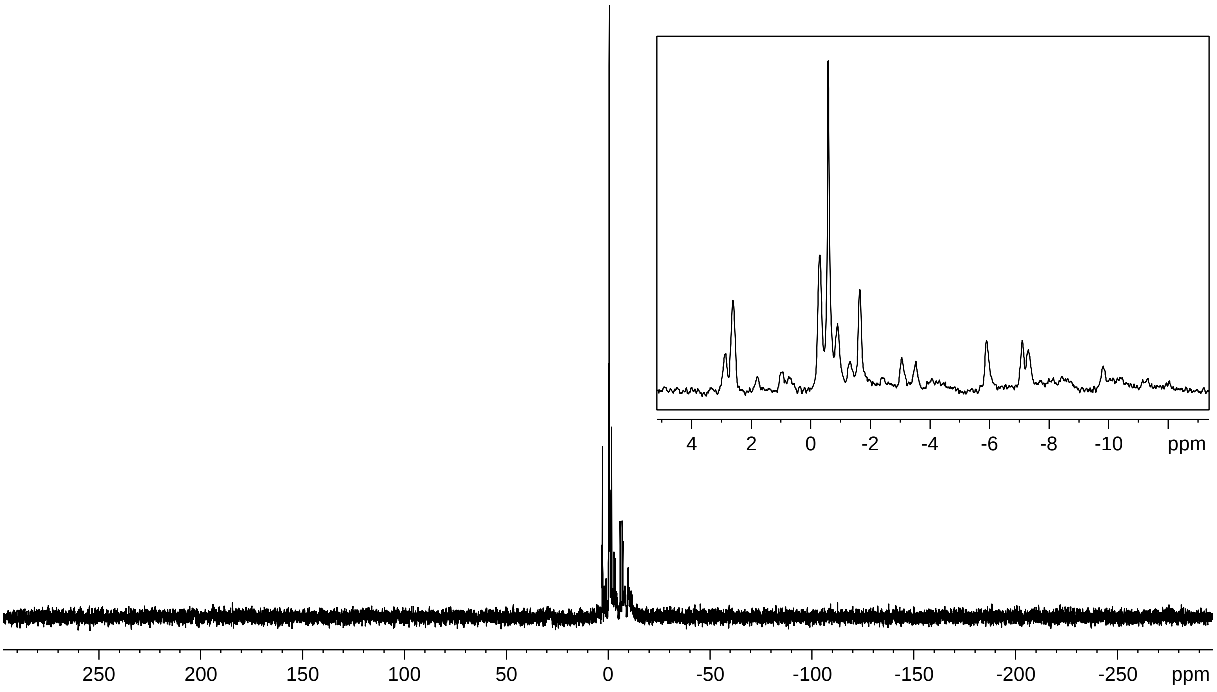

Fig. S 23. ^{31}P NMR spectrum (H_2O, C_6D_6-capillary, 300 K) of reaction of **1b**[OTf] with CyMgCl after quenching with 1 M HCl (aqueous, no signals in organic phase); Several signals in a shift range from – 12 to + 3 ppm indicate formation of various oligophosphates; no formation of dicyclohexylphosphinic acid can be observed.

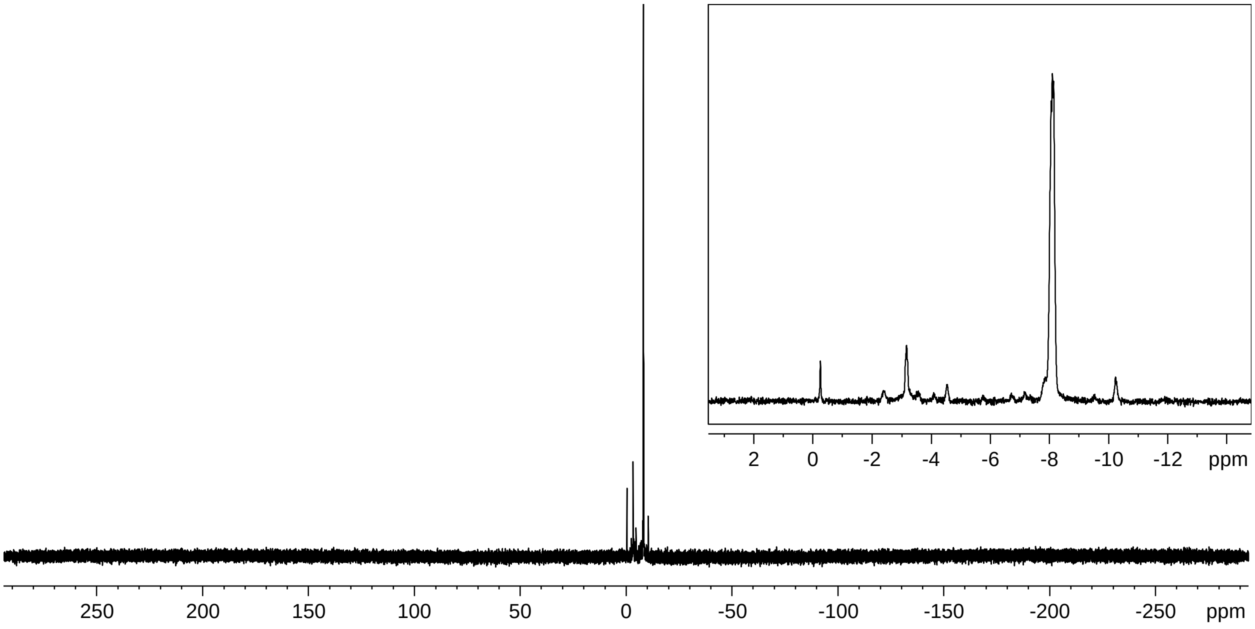

Fig. S 24. ^{31}P NMR spectrum (H_2O, C_6D_6-capillary, 300 K) of reaction of **1b**[OTf] with BnMgCl after quenching with 1 M HCl (aqueous, no signals in organic phase); Several signals in a shift range from – 10 to + 0 ppm indicate formation of various oligophosphates; no formation of dibenzylphosphinic acid can be observed.

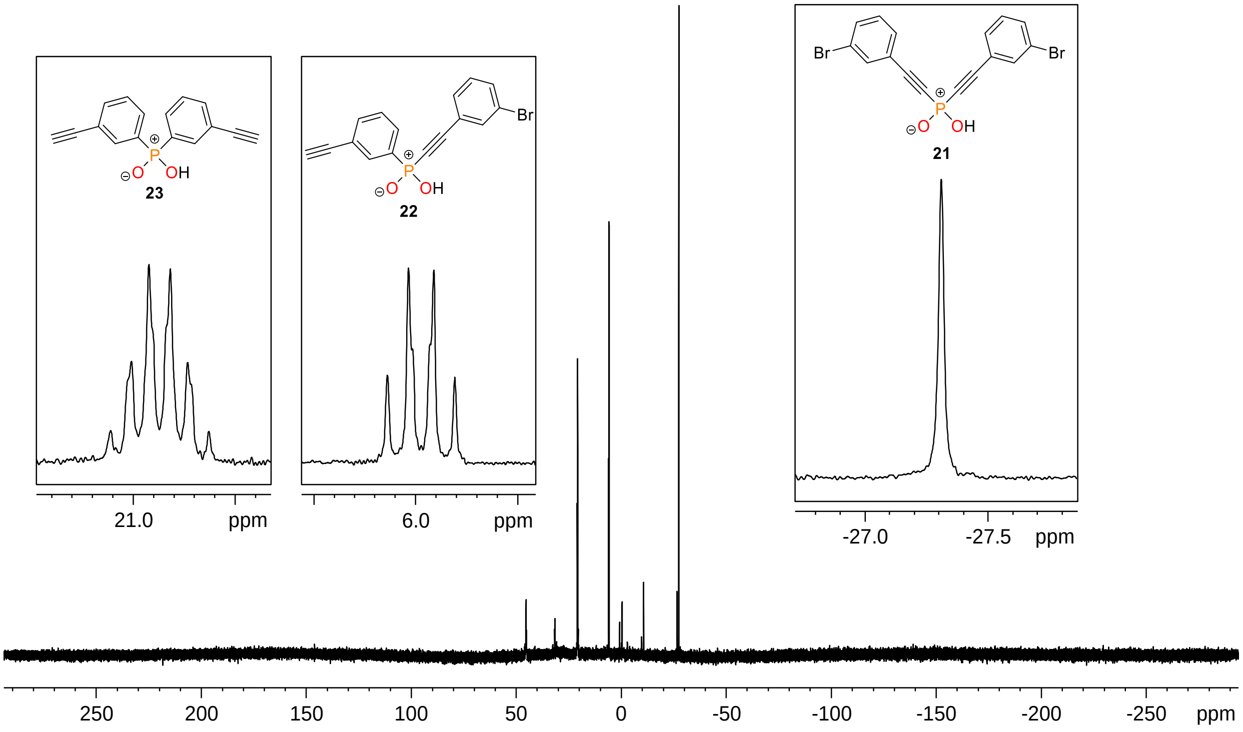

Fig. S 25. ^{31}P NMR spectrum (THF, C_6D_6-capillary, 300 K) of reaction of **1b**[OTf] with ((3-bromophenyl)-ethynyl)magnesium bromide after quenching with 1 M HCl (organic phase); The three main signals were tentatively assigned as the dialkynyl- (**21**, $\delta(^{31}P) = -27.3$ ppm, s), aryl-alkynyl- (**22**, $\delta(^{31}P) = 6.0$ ppm, *pseudo*-q, $^{2}J_{PH} \approx {}^{3}J_{PH} = 22.2$ Hz) and diarylphosphinic acid (**23**, $\delta(^{31}P) = 20.8$ ppm, *pseudo*-sxt, $^{2}J_{PH} \approx {}^{3}J_{PH} = 19.3$ Hz).

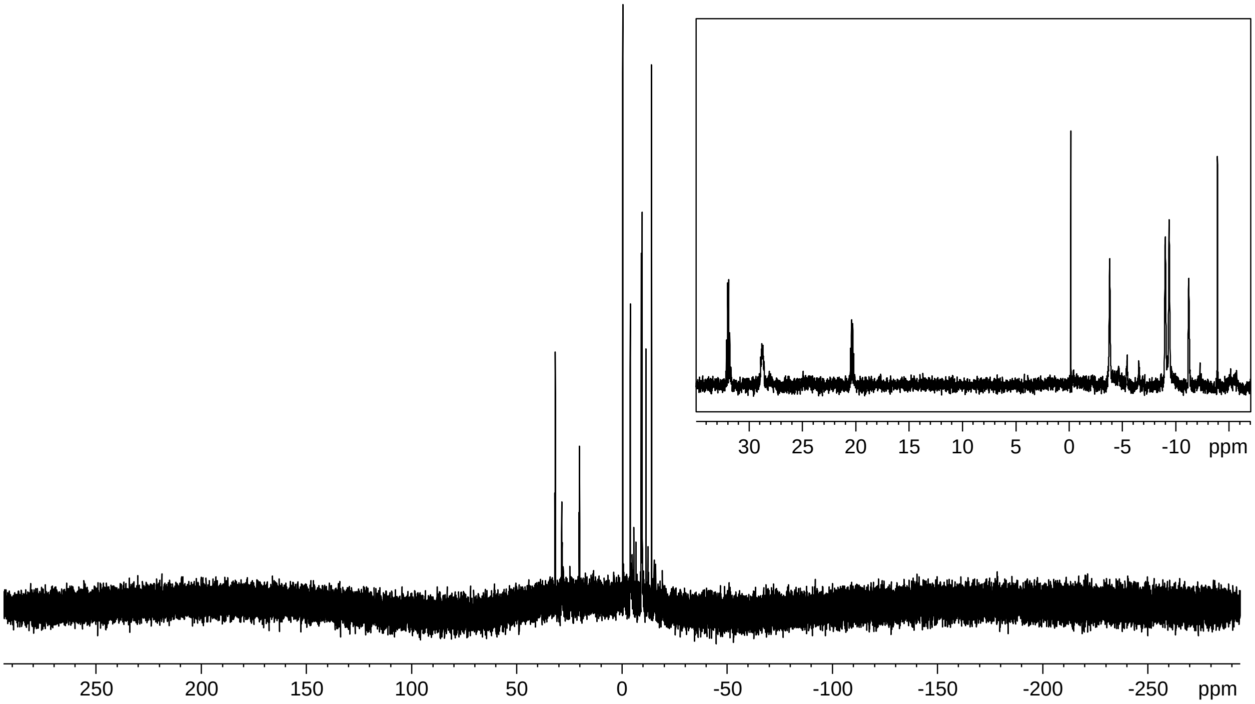

Fig. S 26. ^{31}P NMR spectrum (THF / H_2O, C_6D_6-capillary, 300 K) of reaction of **1b**[OTf] with (pyridin-2-ylethynyl)-magnesium bromide after quenching with 1 M HCl (no phase separation); A mixture of several unidentified signals indicates multiple unselective reaction pathways; No signals are observed in the characteristic chemical shift range of dialkynylphosphinic acids (– 15 to – 35 ppm).

2.2.8. Stability tests of **1a**[OTf] and **1b**[OTf]

Samples of [(pyridine)$_2$PO$_2$][OTf] (**1a**[OTf]) and [(DMAP)$_2$PO$_2$][OTf] (**1b**[OTf]) were prepared under an inert atmosphere and then exposed openly to air for 96 hours. After every 24 hours, the samples were stirred with a spatula to avoid formation of a passivation layer. During exposure, the sample of **1a**[OTf] changed from a fine, free-flowing powder to a firmer and coarser structure. The sample of **1b**[OTf] remained a fine, free-flowing powder throughout the full exposure time. After completed exposure, the samples were taken back into an inert atmosphere and investigated by means of ^{31}P NMR spectroscopy with dry CD$_3$CN and CD$_3$NO$_2$, respectively (Fig. S 27 and Fig. S 28).

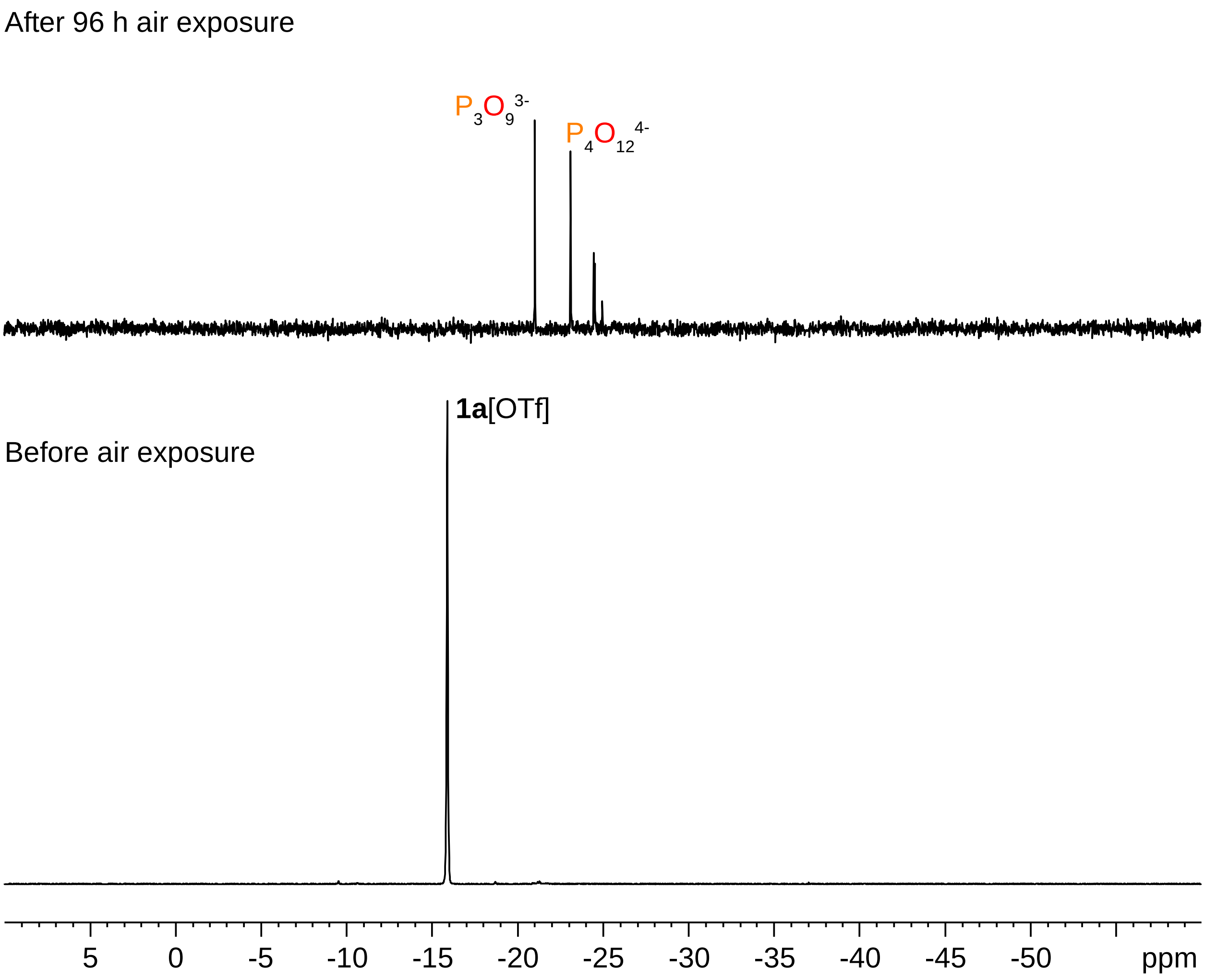

Fig. S 27. ^{31}P NMR spectra (CD$_3$CN, 300 K) of **1a**[OTf] before and after exposure to air for 96 h; After exposure, the compound's characteristic signal (δ(^{31}P) = – 15.9 ppm, s) fully disappeared. Two new main signals at δ(^{31}P) = – 21.0 ppm and δ(^{31}P) = – 23.1 ppm were tentatively identified as tri- ($P_3O_9^{3-}$) and tetrametaphosphate ($P_4O_{12}^{4-}$), respectively, indicating typical cyclo-oligomerization after hydrolysis of the first P–N bond.[1]

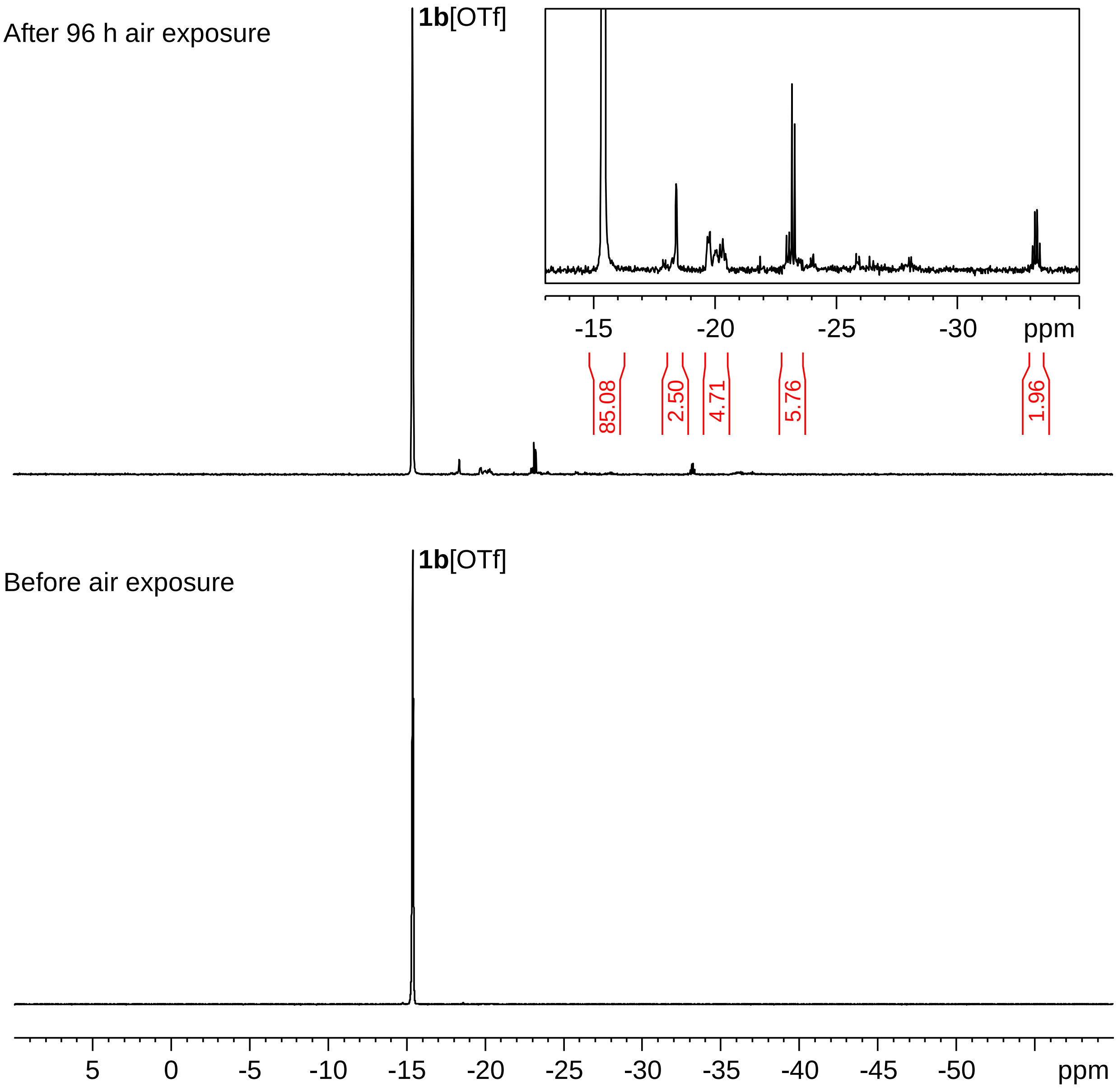

Fig. S 28. ^{31}P NMR spectra (CD_3NO_2, 300 K) of **1b**[OTf] before and after exposure to air for 96 h; After exposure, the majority of the sample remains structurally intact as evidenced by the main signal at $\delta(^{31}P) = -15.4$ ppm characteristic for **1b**[OTf]. Partial hydrolysis to several unidentified oligophosphates has decreased the sample purity to approximately 85 % as derived from integration of all signals.

2.3. Preparation of Diarylphosphinates

2.3.1. General procedure

For preparation of the diarylphosphinic acids, $(DMAP)_2PO_2[OTf]$ (**1b**[OTf], 1 eq.) was slowly added to a stirred solution of the Grignard reagent (2.5 eq.) diluted with toluene to approx. 0.25 mol/L. Grignards which were not commercially available were prepared *in situ* from the corresponding arylbromide by reaction with equimolar amounts of magnesium turnings (see Table S 3). After stirring the mixture for 4 to 16 h, a clear solution was obtained and quenched with 1 M HCl. The organic phase was then separated from the mixture and the remaining aqueous phase extracted with $CHCl_3$ three more times. The combined organic phases were washed twice with acidified brine (0.1 M HCl) followed by re-extraction with 1 M NaOH for three times. Subsequent acidification of the combined aqueous phases (pH = 1) with conc. HCl led to precipitation of the diarylphosphinic acid, which was obtained as a colorless to off-white powder after filtration, washing with 1 M HCl and drying *in vacuo*. Analytical details and individual information about the synthetic procedure are provided in sections 2.3.2 to 2.3.9.

Table S 3. Details on used Grignard reagents for the synthesis of diarylphosphinic acids.

Grignard reagent	source	conc. / solvent	reaction scale (amount of 1b[OTf])
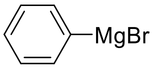	commercial	1 M in THF	1 mmol
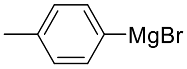	commercial	1 M in THF	1 mmol
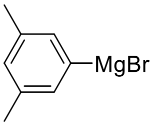	prepared *in situ*	0.5 M in THF	2 mmol
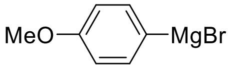	commercial	0.5 M in THF	1 mmol
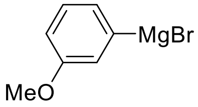	commercial	1 M in THF	1 mmol
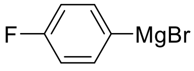	commercial	1 M in THF	1 mmol
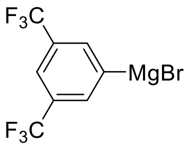	prepared *in situ*	0.5 M in Et_2O	2 mmol
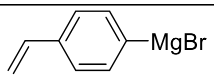	prepared *in situ*	0.5 M in THF	2 mmol

2.3.2. Preparation of Diphenylphosphinic acid (**2a**)

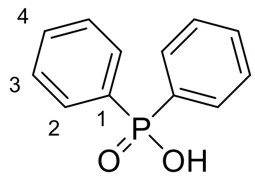

Diphenylphosphinic acid (**2a**) was synthesized according to the general procedure from 1 mmol of $(DMAP)_2PO_2[OTf]$ (**1b**[OTf], 456 mg, 1 eq.) with 2.5 mmol of phenylmagnesium bromide (1 M in THF, 2.5 mL, 2.5 eq.) and obtained as a colorless powder.

Single crystals of **2a** suitable for single crystal structure determination were obtained by slow evaporation of Et_2O from a concentrated solution (Fig. S 29).

The NMR spectra of the obtained sample were in accordance with previously published analytical data.[8]

Yield: 146 mg (67 %); **Raman** ($\tilde{\nu}$ in cm^{-1}): 3061 (100), 1592 (43), 1574 (9), 1186 (13), 1160 (13), 1139 (11), 1127 (9), 1032 (10), 1008 (30), 999 (36), 697 (29), 617 (16), 299 (7), 244 (25); **IR** (ATR, $\tilde{\nu}$ in cm^{-1}): 3077 (vw), 3056 (vw), 2614 (vw), 2358 (vw), 2140 (vw), 1900 (vw), 1645 (w), 1588 (w), 1484 (w), 1438 (m), 1338 (vw), 1315 (vw), 1177 (m), 1153 (w), 1128 (m), 1120 (s), 1069 (m), 1027 (w), 1002 (m), 976 (m), 955 (vs), 932 (s), 863 (m), 755 (m), 727 (vs), 691 (vs), 549 (vs), 523 (vs), 495 (s), 433 (vs); **m.p.**: 187 – 190 °C (decomp.); **^{1}H NMR** ($CDCl_3$, δ in ppm): 7.34 (td, 4H, $^3J_{HH}$ = 11.4 Hz, $^4J_{HH}$ = 3.6 Hz, H3), 7.43–7.47 (m, 2H, H4), 7.69–7.75 (m, 4H, H2), 8.50 (s(br), 1H, P–OH); **^{13}C{^{1}H} NMR** ($CDCl_3$, δ in ppm): 128.5 (d, $^3J_{CP}$ = 13.4 Hz, C3), 131.4 (d, $^2J_{CP}$ = 10.6 Hz, C2), 132.0 (d, $^4J_{CP}$ = 2.9 Hz, C4), 132.8 (d, $^1J_{CP}$ = 140.1 Hz, C1); **^{31}P NMR** ($CDCl_3$, δ in ppm): 33.3 (m); **elemental analysis:** calc. for $C_{12}H_{11}O_2P$: C 66.1, H 5.1, N 0.0, S 0.0; found: C 65.7, H 5.1, N 0.0, S 0.1.

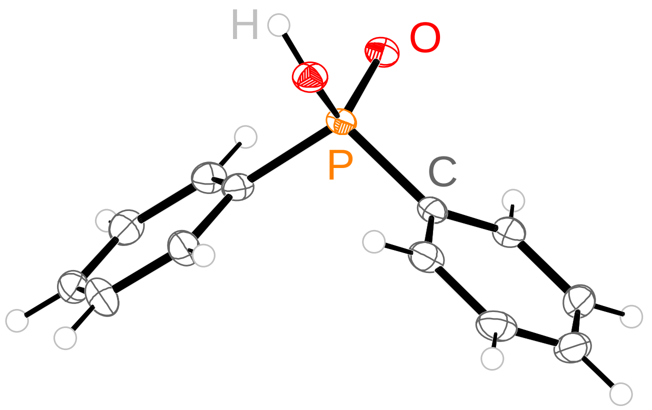

Fig. S 29. Molecular structure of diphenylphosphinic acid (**2a**); thermal ellipsoids are displayed at 50 % probability level; CCDC 2442184.

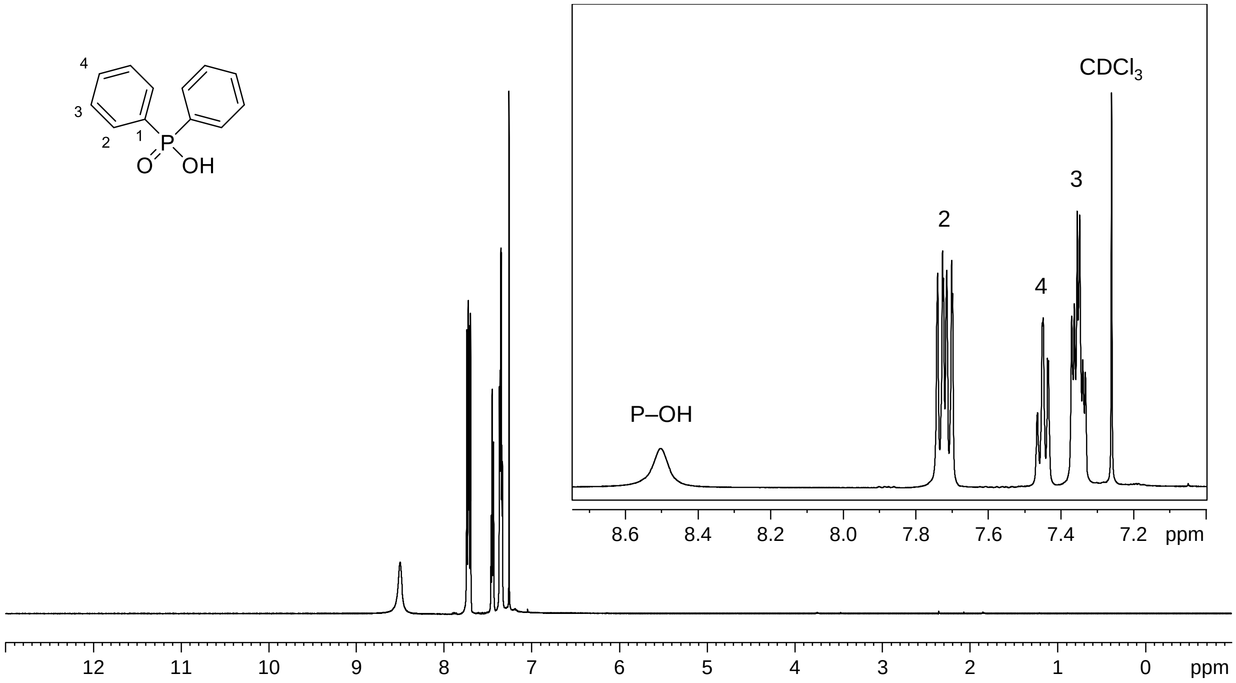

Fig. S 30. ^{1}H NMR spectrum ($CDCl_3$, 300 K) of diphenylphosphinic acid (**2a**) synthesized from **1b**[OTf].

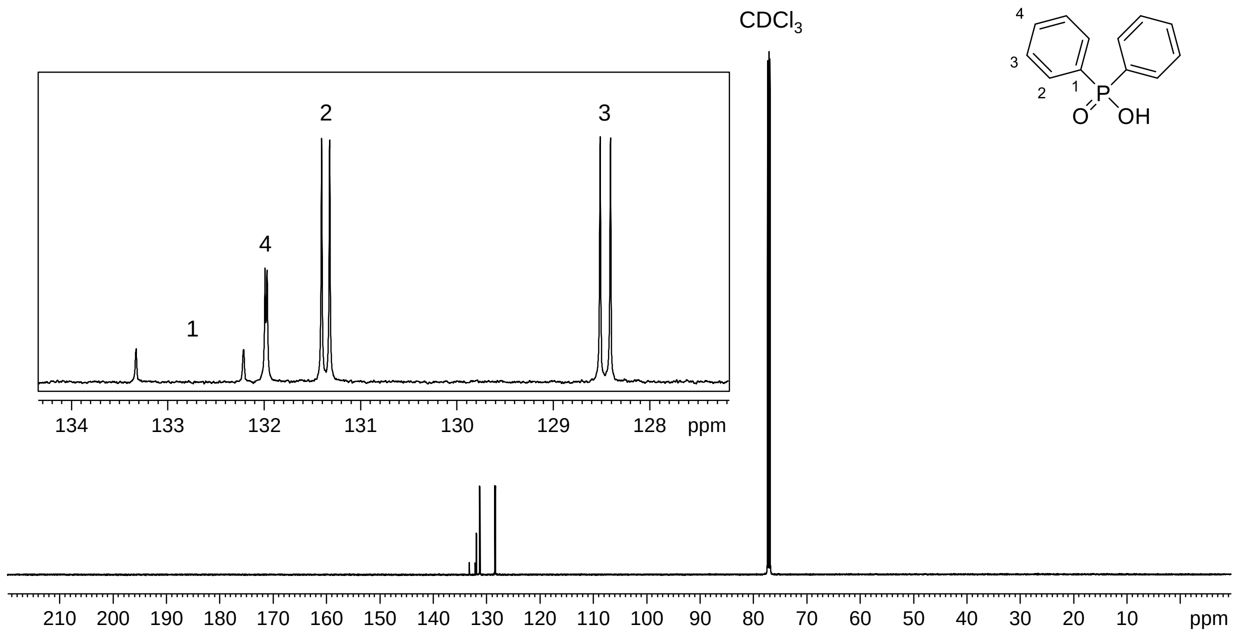

Fig. S 31. $^{13}C\{^{1}H\}$ NMR spectrum ($CDCl_3$, 300 K) of diphenylphosphinic acid (**2a**) synthesized from **1b**[OTf].

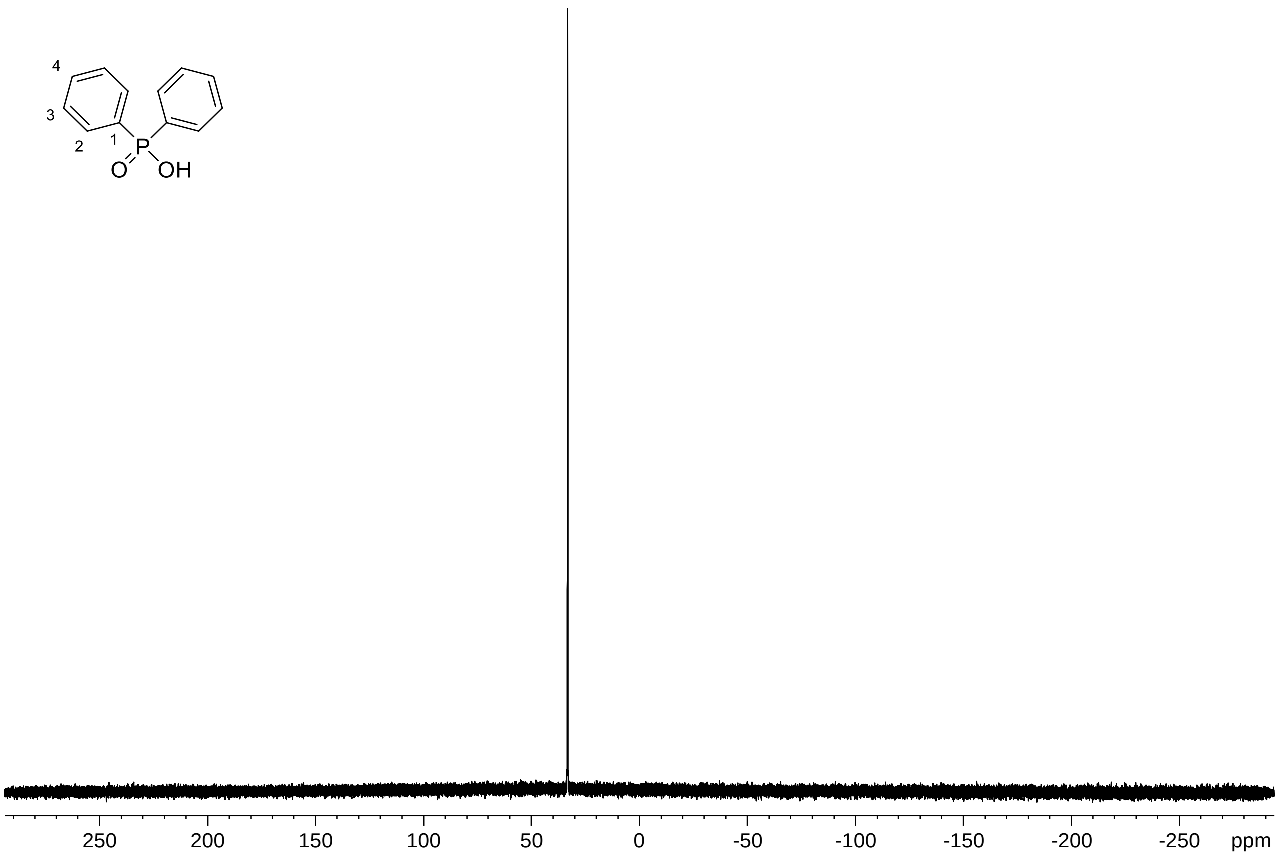

Fig. S 32. ^{31}P NMR spectrum ($CDCl_3$, 300 K) of diphenylphosphinic acid (**2a**) synthesized from **1b**[OTf].

2.3.3. Preparation of Di-*p*-tolylphosphinic acid (**2b**)

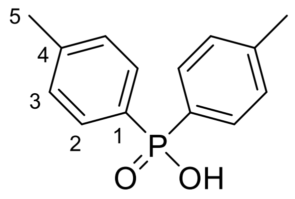

Di-*p*-tolylphosphinic acid (**2b**) was synthesized according to the general procedure from 1 mmol of $(DMAP)_2PO_2[OTf]$ (**1b**[OTf], 456 mg, 1 eq.) with 2.5 mmol of *p*-tolylmagnesium bromide (1 M in THF, 2.5 mL, 2.5 eq.) and obtained as a colorless powder.

Single crystals of **2b** suitable for single crystal structure determination were obtained by slow evaporation of Et_2O from a concentrated solution (Fig. S 33).

The NMR spectra of the obtained sample were in accordance with previously published analytical data.[8]

Yield: 171 mg (69 %); **Raman** ($\tilde{\nu}$ in cm^{-1}): 3055 (100), 3038 (28), 3022 (16), 2993 (11), 2955 (10), 2920 (56), 2867 (11), 1604 (79), 1566 (5), 1453 (5), 1382 (16), 1375 (14), 1309 (6), 1215 (14), 1189 (22), 1138 (45), 811 (47), 795 (42), 670 (5), 637 (28), 625 (6), 344 (6), 278 (11), 246 (15); **IR** (ATR, $\tilde{\nu}$ in cm^{-1}): 3037 (vw), 3022 (vw), 2985 (vw), 2956 (vw), 2918 (vw), 2864 (vw), 2585 (vw), 2248 (vw), 2117 (vw), 1923 (vw), 1694 (w), 1603 (w), 1562 (w), 1501 (w), 1447 (w), 1399 (w), 1374 (vw), 1311 (vw), 1214 (vw), 1187 (vw), 1158 (m), 1129 (s), 1036 (m), 1003 (m), 959 (s), 920 (m), 859 (w), 804 (s), 711 (m), 667 (s), 637 (w), 620 (m), 519 (vs), 493 (m), 476 (s), 459 (s), 448 (s); **m.p.**: 134 – 136 °C (decomp.); **^{1}H NMR** ($CDCl_3$, δ in ppm): 2.34 (s, 6H, H5), 7.14 (dd, 4H, $^3J_{HH}$ = 8.0 Hz, $^4J_{HP}$ = 3.0 Hz, H3), 7.59 (dd, 4H, $^3J_{HP}$ = 12.4 Hz, $^3J_{HH}$ = 8.0 Hz, H2), 10.39 (s(br), 1H, P–OH); **^{13}C{^{1}H} NMR** ($CDCl_3$, δ in ppm): 21.7 (d, $^5J_{CP}$ = 1.2 Hz, C5), 129.1 (d, $^3J_{CP}$ = 13.8 Hz, C3), 129.9 (d, $^1J_{CP}$ = 142.7 Hz, C1), 131.4 (d, $^2J_{CP}$ = 11.0 Hz, C2), 142.2 (d, $^4J_{CP}$ = 2.9 Hz, C4); **^{31}P NMR** ($CDCl_3$, δ in ppm): 34.1 (m); **elemental analysis:** calc. for $C_{14}H_{15}O_2P$: C 68.3, H 6.1, N 0.0, S 0.0; found: C 67.9, H 6.2, N 0.0, S 0.0.

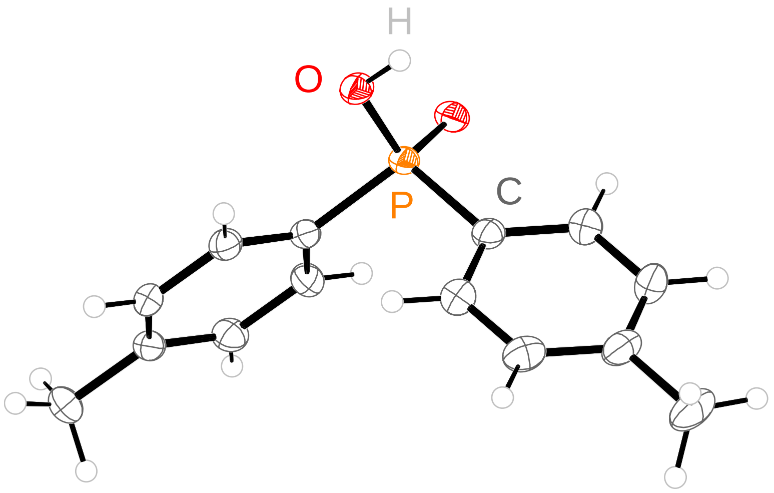

Fig. S 33. Molecular structure of di-*p*-tolylphosphinic acid (**2b**); thermal ellipsoids are displayed at 50 % probability level; CCDC 2442190.

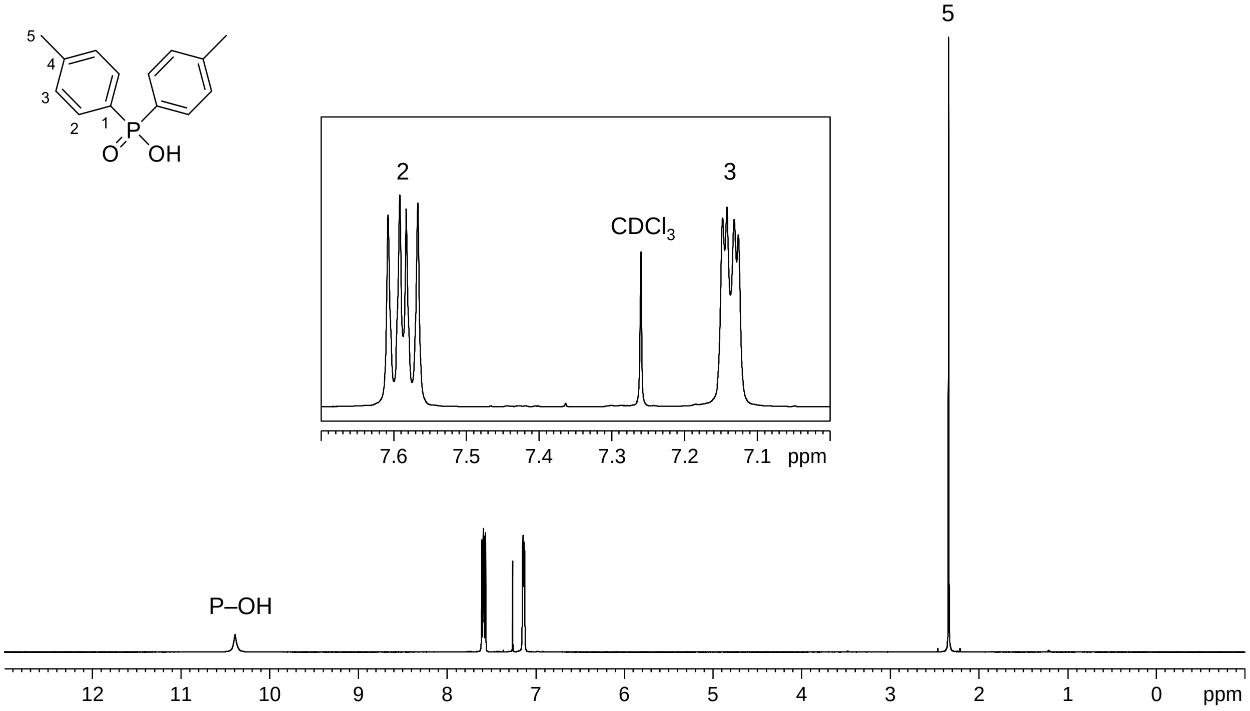

Fig. S 34. ^{1}H NMR spectrum ($CDCl_3$, 300 K) of di-*p*-tolylphosphinic acid (**2b**) synthesized from **1b**[OTf].

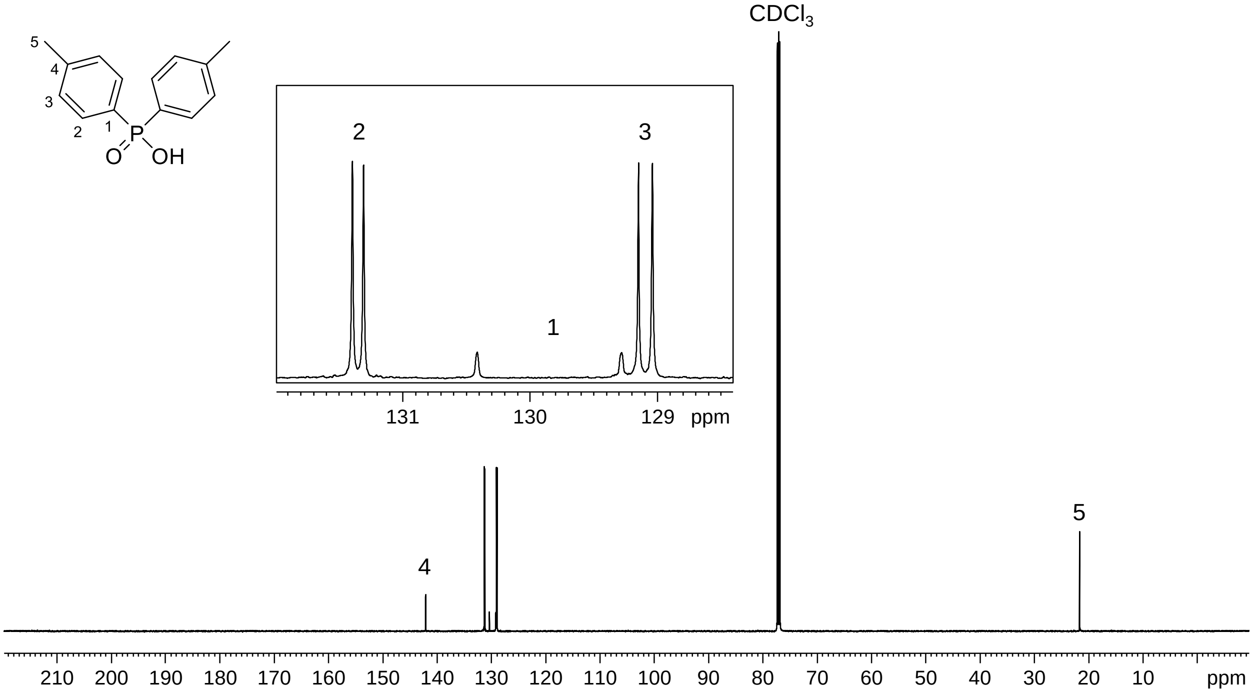

Fig. S 35. $^{13}C\{^{1}H\}$ NMR spectrum ($CDCl_3$, 300 K) of di-*p*-tolylphosphinic acid (**2b**) synthesized from **1b**[OTf].

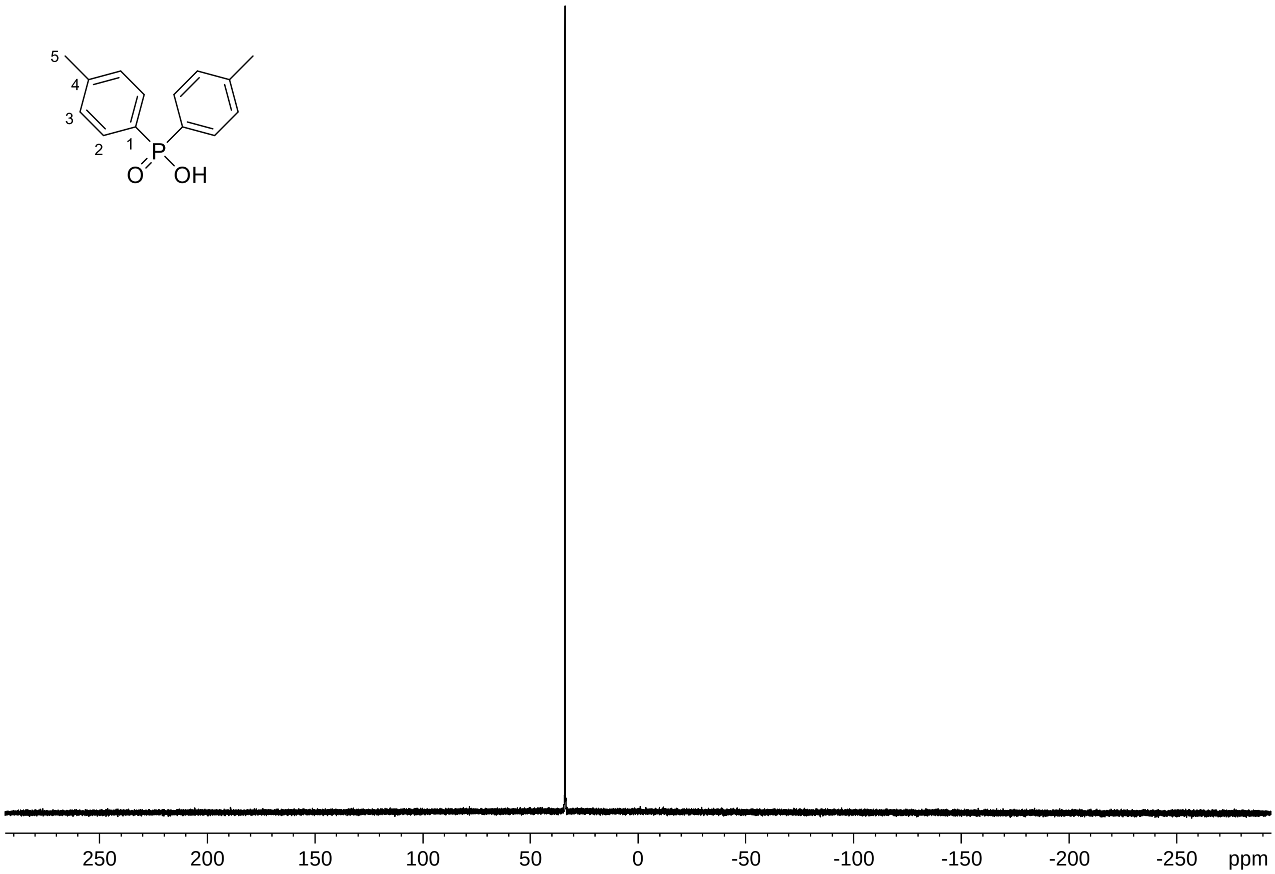

Fig. S 36. ^{31}P NMR spectrum ($CDCl_3$, 300 K) of di-*p*-tolylphosphinic acid (**2b**) synthesized from **1b**[OTf].

2.3.4. Preparation of Bis(3,5-dimethylphenyl)phosphinic acid (**2c**)

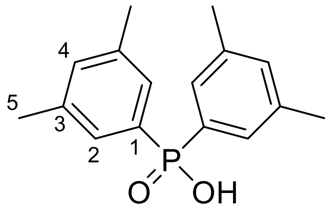

Bis(3,5-dimethylphenyl)phosphinic acid (**2c**) was synthesized according to the general procedure from 2 mmol of $(DMAP)_2PO_2[OTf]$ (**1b**[OTf], 456 mg, 1 eq.) with 5 mmol of the Grignard reagent and obtained as a colorless powder. The Grignard reagent was prepared priorly by slow addition of 1-bromo-3,5-dimethylbenzene (0.68 mL, 5 mmol, 2.5 eq.) to a stirred suspension of dry Mg turnings (122 mg, 5 mmol, 2.5 eq.) in 10 mL of THF and subsequent reflux for 1 h to obtain a clear solution, which was used after cooling back to room temperature.

Single crystals of **2c** suitable for single crystal structure determination were obtained by slow diffusion of *n*-pentane into a concentrated $CHCl_3$ solution (Fig. S 37).

The NMR spectra of the obtained sample were in accordance with previously published analytical data.[8]

Yield: 335 mg (61 %); **Raman** ($\tilde{\nu}$ in cm^{-1}): 3036 (34), 3006 (25), 2976 (9), 2915 (100), 2862 (25), 2732 (9), 2221 (5), 1591 (43), 1380 (25), 1277 (34), 1171 (5), 1150 (7), 1026 (9), 994 (50), 574 (25), 548 (61), 530 (5), 522 (13), 512 (9), 314 (16), 266 (20), 252 (14), 226 (41); **IR** (ATR, $\tilde{\nu}$ in cm^{-1}): 2914 (vw), 2858 (vw), 2476 (vw), 2231 (vw), 1688 (w), 1602 (w), 1423 (w), 1381 (vw), 1310 (vw), 1276 (w), 1209 (m), 1169 (m), 1149 (m), 1130 (m), 1107 (m), 1042 (w), 1003 (m), 981 (m), 958 (s), 918 (w), 901 (w), 881 (s), 852 (s), 692 (s), 689 (s), 590 (vs), 573 (s), 538 (w), 527 (w), 511 (vw), 457 (vs); **m.p.**: 258 – 260 °C; **^{1}H NMR** ($CDCl_3$, δ in ppm): 2.27 (s, 12H, H5), 7.06 (s, 2H, H4), 7.34 (d, 4H, $^3J_{HP}$ = 12.9 Hz, H2), 10.20 (s(br), 1H, P–OH); **^{13}C{^{1}H} NMR** ($CDCl_3$, δ in ppm): 21.3 (d, $^4J_{CP}$ = 1.0 Hz, C5), 128.9 (d, $^2J_{CP}$ = 10.6 Hz, C2), 132.6 (d, $^1J_{CP}$ = 138.2 Hz, C1), 133.7 (d, $^4J_{CP}$ = 3.0 Hz, C4), 138.0 (d, $^3J_{CP}$ = 14.1 Hz, C3); **^{31}P NMR** ($CDCl_3$, δ in ppm): 35.3 (quin, $^3J_{PH}$ = 12.9 Hz); **elemental analysis:** calc. for $C_{16}H_{19}O_2P$: C 70.1, H 7.0, N 0.0, S 0.0; found: C 69.7, H 6.7, N 0.1, S 0.1.

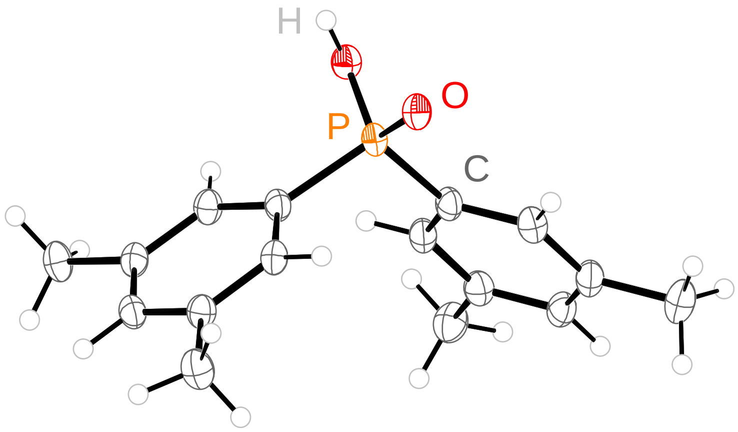

Fig. S 37. Molecular structure of bis(3,5-dimethylphenyl)phosphinic acid (**2c**); thermal ellipsoids are displayed at 50 % probability level; CCDC 2442203.

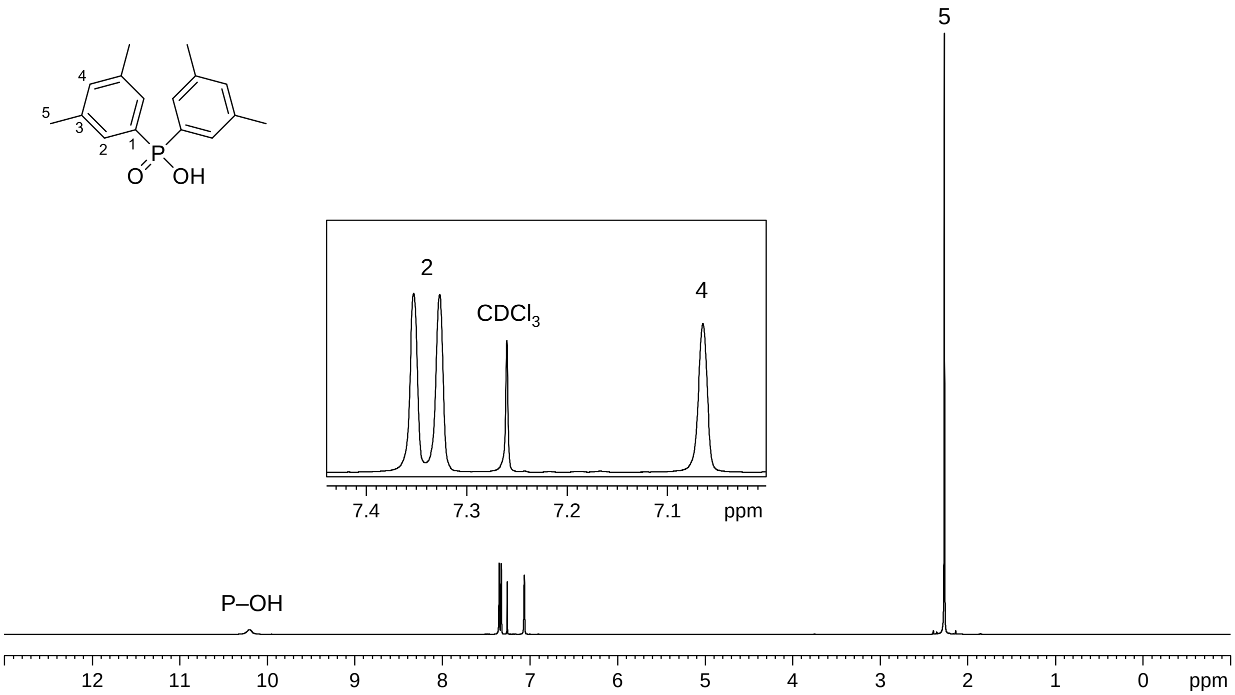

Fig. S 38. ^{1}H NMR spectrum ($CDCl_3$, 300 K) of bis(3,5-dimethylphenyl)phosphinic acid (**2c**) synthesized from **1b**[OTf].

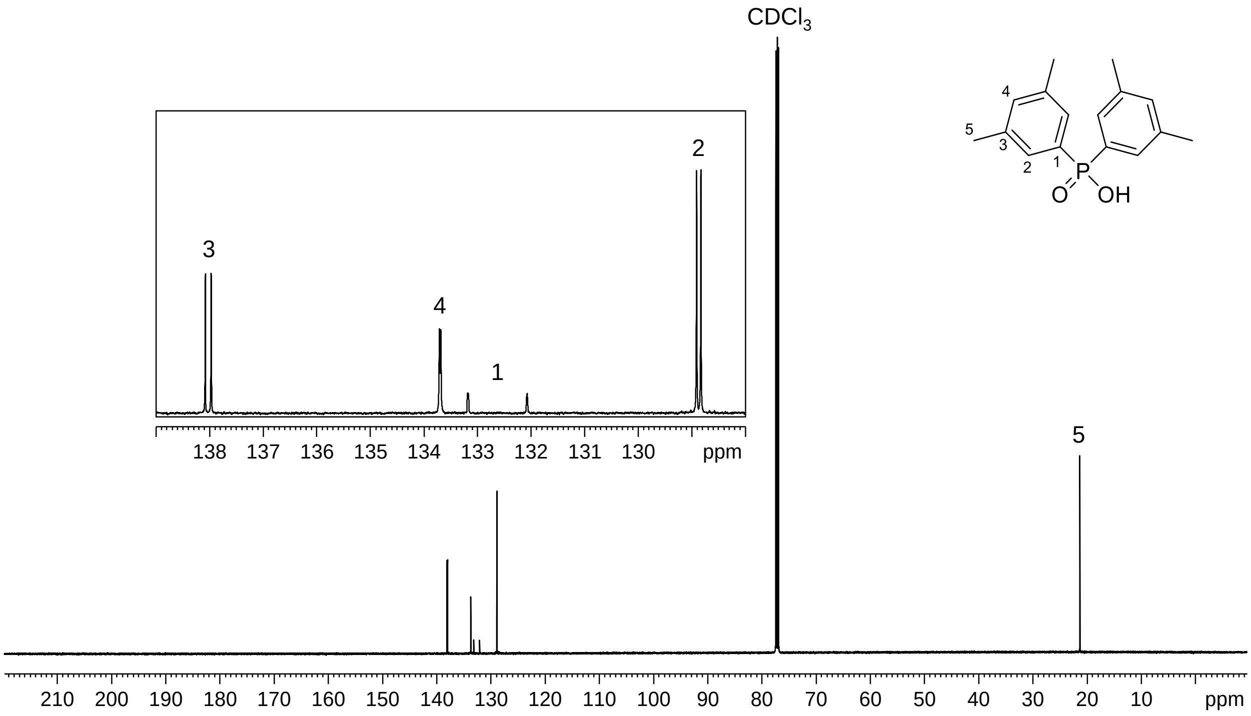

Fig. S 39. $^{13}C\{^{1}H\}$ NMR spectrum ($CDCl_3$, 300 K) of bis(3,5-dimethylphenyl)phosphinic acid (**2c**) synthesized from **1b**[OTf].

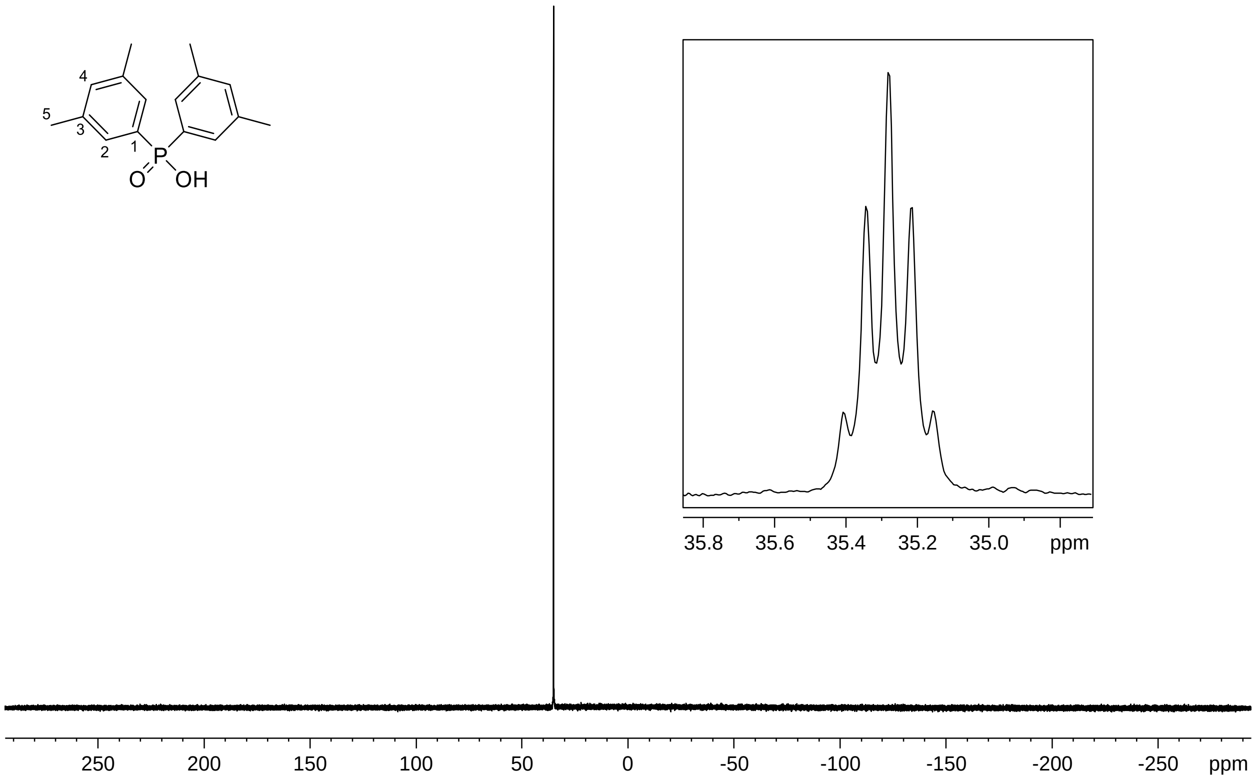

Fig. S 40. ^{31}P NMR spectrum ($CDCl_3$, 300 K) of bis(3,5-dimethylphenyl)phosphinic acid (**2c**) synthesized from **1b**[OTf].

2.3.5. Preparation of Bis(4-methoxyphenyl)phosphinic acid (**2d**)

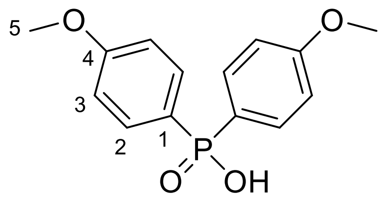

Bis(4-methoxyphenyl)phosphinic acid (**2d**) was synthesized according to the general procedure from 1 mmol of $(DMAP)_2PO_2$[OTf] (**1b**[OTf], 456 mg, 1 eq.) with 2.5 mmol of *p*-methoxyphenylmagnesium bromide (0.5 M in THF, 5 mL, 2.5 eq.) and obtained as a colorless powder. The compound first separated as a wax-like substance in the final precipitation step, which solidified upon further stirring the mixture for 10 min.

Single crystals of **2d** suitable for single crystal structure determination were obtained by slow vapor diffusion of *n*-pentane into a concentrated $CHCl_3$ solution (Fig. S 41).

The NMR spectra of the obtained sample were in accordance with previously published analytical data.[8]

Yield: 167 mg (60 %); **Raman** ($\tilde{\nu}$ in cm^{-1}): 3069 (80), 3012 (27), 2947 (20), 2930 (13), 2839 (47), 1598 (93), 1572 (13), 1460 (13), 1441 (13), 1306 (7), 1296 (7), 1257 (7), 1177 (20), 1158 (73), 1129 (20), 1108 (20), 1030 (7), 1011 (7), 818 (13), 801 (100), 673 (7), 634 (13), 548 (7), 458 (7), 361 (13), 272 (13), 254 (13); **IR** (ATR, $\tilde{\nu}$ in cm^{-1}): 3093 (vw), 3009 (vw), 2933 (vw), 2895 (vw), 2838 (vw), 2604 (vw), 2323 (vw), 2149 (vw), 2051 (vw), 1596 (s), 1572 (w), 1503 (m), 1461 (w), 1440 (vw), 1409 (vw), 1297 (w), 1251 (s), 1200 (s), 1182 (s), 1176 (s), 1127 (s), 1105 (s), 1027 (s), 951 (s), 934 (m), 836 (m), 803 (s), 721 (w), 669 (s), 626 (w), 542 (vs), 505 (m), 468 (m), 457 (m), 430 (m); **m.p.**: 180 – 182 °C (decomp.); **^{1}H NMR** ($CDCl_3$, δ in ppm): 3.80 (s, 6H, H5), 6.83 (dd, 4H, $^3J_{HH}$ = 8.8 Hz, $^4J_{HP}$ = 2.3 Hz, H3), 7.62 (dd, 4H, $^3J_{HP}$ = 12.1 Hz, $^3J_{HH}$ = 8.8 Hz, H2), 9.63 (s(br), 1H, P–OH); **^{13}C{^{1}H} NMR** ($CDCl_3$, δ in ppm): 55.4 (s, C5), 113.9 (d, $^3J_{CP}$ = 14.4 Hz, C3), 124.7 (d, $^1J_{CP}$ = 146.9 Hz, C1), 133.2 (d, $^2J_{CP}$ = 12.0 Hz, C2), 162.4 (d, $^4J_{CP}$ = 2.9 Hz, C4); **^{31}P NMR** ($CDCl_3$, δ in ppm): 33.9 (m); **elemental analysis:** calc. for $C_{14}H_{15}O_4P$: C 60.4, H 5.4, N 0.0, S 0.0; found: C 60.0, H 5.5, N 0.0, S 0.1.

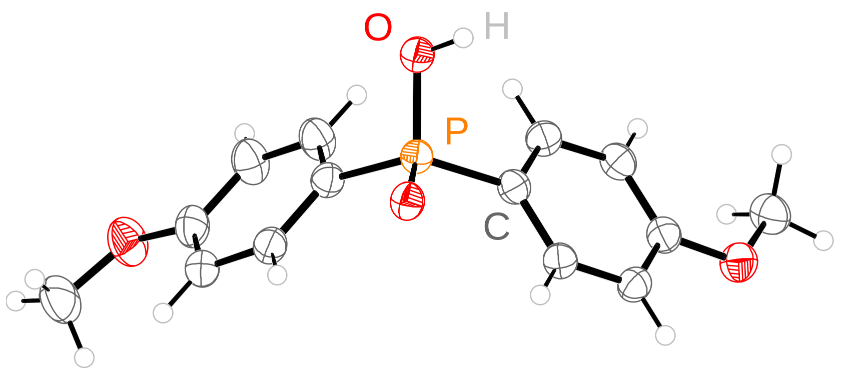

Fig. S 41. Molecular structure of bis(4-methoxyphenyl)phosphinic acid (**2d**); thermal ellipsoids are displayed at 50 % probability level; CCDC 2442191.

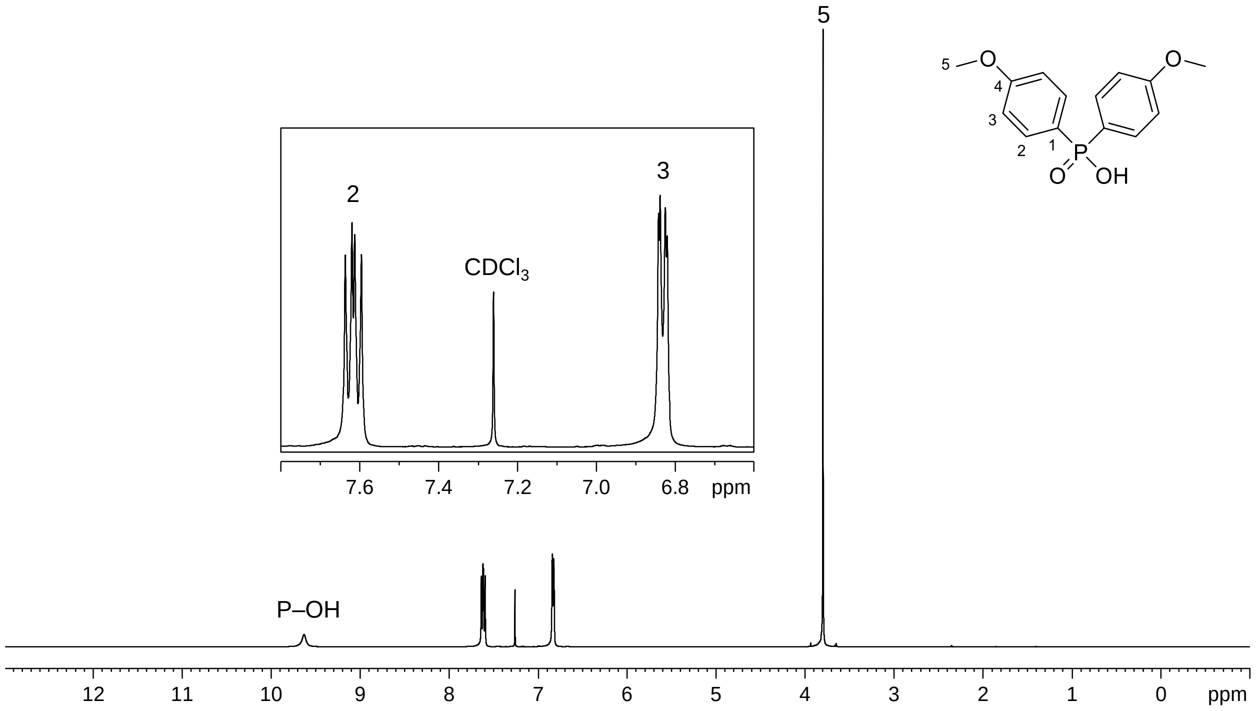

Fig. S 42. ^{1}H NMR spectrum ($CDCl_3$, 300 K) of bis(4-methoxyphenyl)phosphinic acid (**2d**) synthesized from **1b**[OTf].

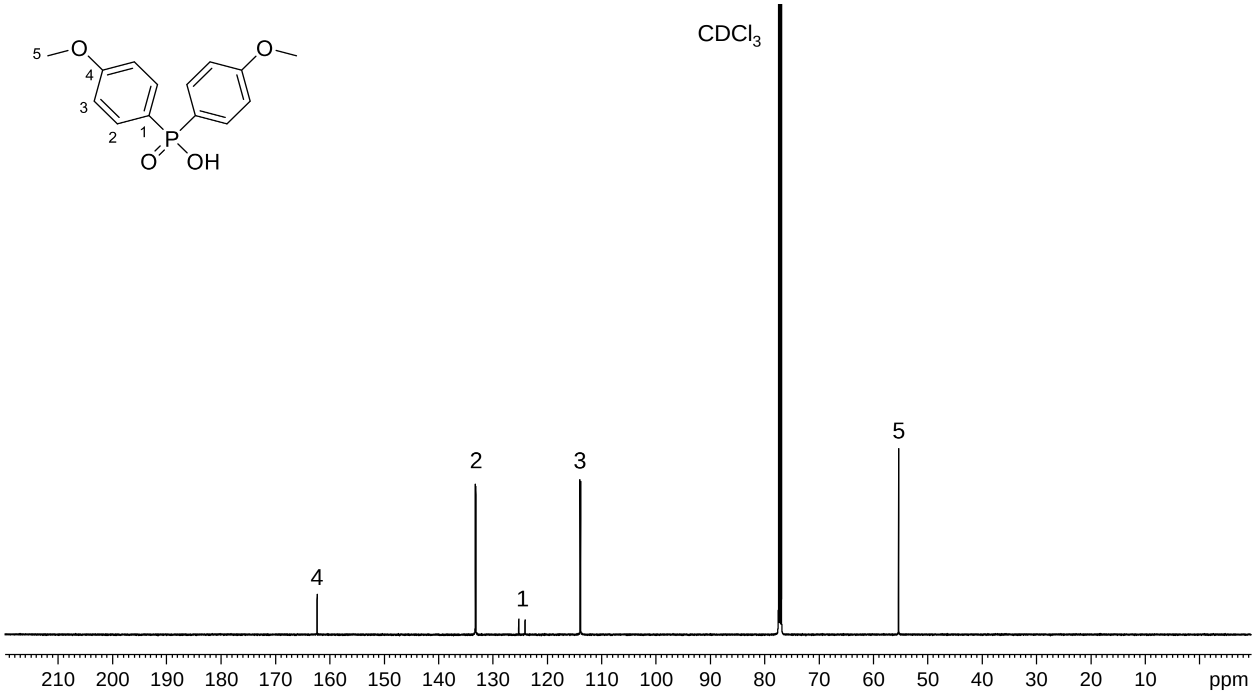

Fig. S 43. $^{13}C\{^1H\}$ NMR spectrum ($CDCl_3$, 300 K) of bis(4-methoxyphenyl)phosphinic acid (**2d**) synthesized from **1b**[OTf].

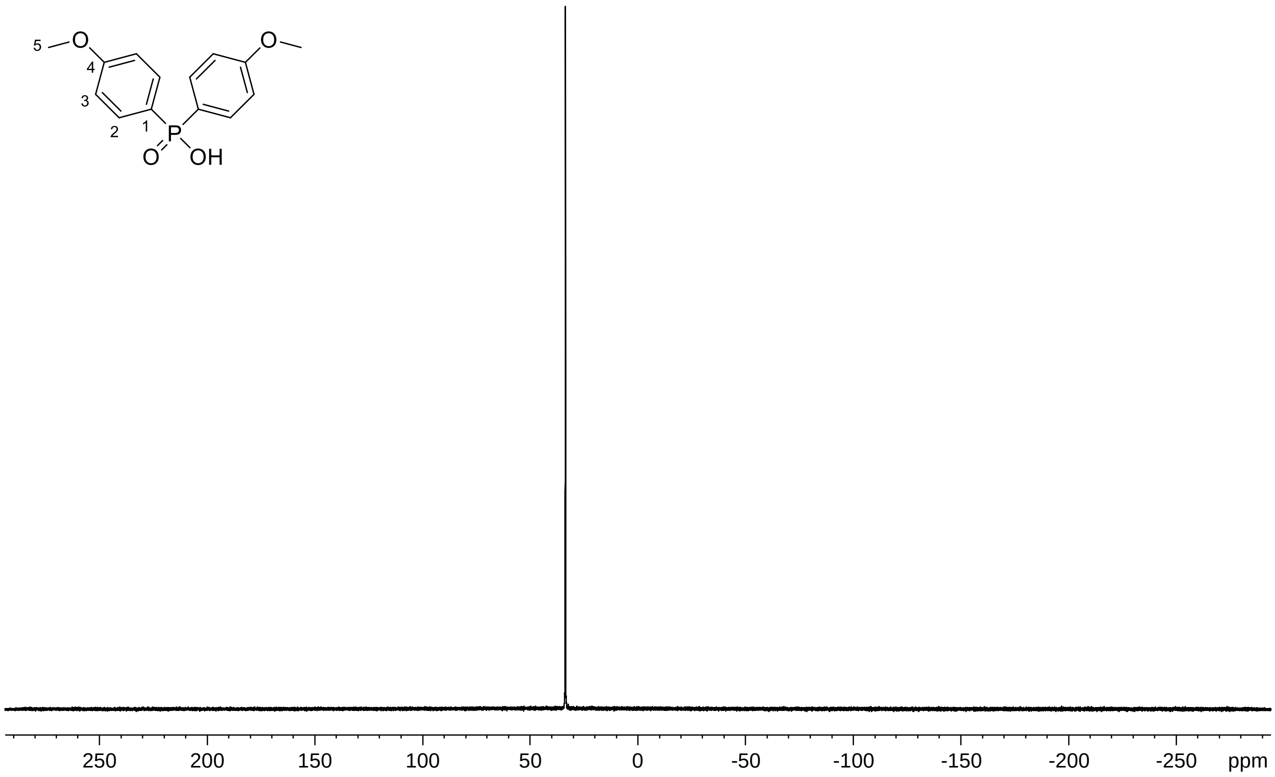

Fig. S 44. ^{31}P NMR spectrum ($CDCl_3$, 300 K) of bis(4-methoxyphenyl)phosphinic acid (**2d**) synthesized from **1b**[OTf].

2.3.6. Preparation of Bis(3-methoxyphenyl)phosphinic acid (**2e**)

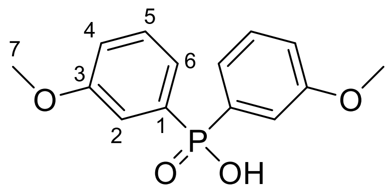

Bis(3-methoxyphenyl)phosphinic acid (**2e**) was synthesized according to the general procedure from 1 mmol of $(DMAP)_2PO_2[OTf]$ (**1b**[OTf], 456 mg, 1 eq.) with 2.5 mmol of *m*-methoxyphenylmagnesium bromide (1 M in THF, 2.5 mL, 2.5 eq.) and obtained as a colorless powder.

Single crystals of **2e** suitable for single crystal structure determination were obtained by slow vapor diffusion of Et_2O into a concentrated DMF solution (Fig. S 45).

The NMR spectra of the obtained sample were in accordance with previously published analytical data.[8]

Yield: 213 mg (77 %); **Raman** ($\tilde{\nu}$ in cm^{-1}): 3088 (19), 3061 (79), 3045 (10), 3027 (32), 2975 (15), 2944 (27), 2841 (23), 1598 (27), 1575 (22), 1458 (9), 1443 (7), 1288 (13), 1232 (24), 1189 (17), 1172 (6), 1093 (10), 1040 (6), 990 (100), 980 (5), 786 (5), 705 (5), 684 (29), 603 (5), 501 (6), 294 (13), 234 (19); **IR** (ATR, $\tilde{\nu}$ in cm^{-1}): 3088 (vw), 3060 (vw), 2974 (vw), 2943 (vw), 2840 (vw), 2674 (vw), 2516 (vw), 2072 (vw), 1934 (vw), 1850 (vw), 1808 (vw), 1596 (w), 1576 (m), 1473 (m), 1458 (m), 1425 (s), 1327 (w), 1307 (w), 1290 (s), 1233 (s), 1187 (m), 1163 (m), 1102 (m), 1090 (m), 1040 (s), 992 (s), 959 (vs), 907 (s), 881 (s), 865 (vs), 782 (s), 692 (vs), 590 (vs), 569 (vs), 511 (s), 475 (vs), 426 (s); **m.p.**: 185 – 187 °C; **^{1}H NMR** (DMSO-d_6, δ in ppm): 3.77 (s, 6H, H7), 7.09 (dd, 2H, $^{3}J_{HH}$ = 8.3 Hz, $^{4}J_{HH}$ = 2.7 Hz, H4), 7.23 (ddd, 2H, $^{3}J_{HP}$ = 13.3 Hz, $^{4}J_{HH}$ = 2.6 / 1.1 Hz, H2), 7.27 (dd, 2H, $^{3}J_{HP}$ = 11.4 Hz, $^{3}J_{HH}$ = 7.7 Hz, H6), 7.38 (td, $^{3}J_{HH}$ = 7.7 Hz, $^{4}J_{HP}$ = 4.3 Hz, H5), 12.1 (s(br), 1H, P–OH); **^{13}C{^{1}H} NMR** (DMSO-d_6, δ in ppm): 55.2 (s, C7), 115.9 (d, $^{2}J_{CP}$ = 11.0 Hz, C6), 117.0 (d, $^{4}J_{CP}$ = 2.6 Hz, C4), 123.1 (d, $^{2}J_{CP}$ = 9.6 Hz, C2), 129.9 (d, $^{3}J_{CP}$ = 14.7 Hz, C5), 136.3 (d, $^{1}J_{CP}$ = 133.7 Hz, C1), 158.9 (d, $^{3}J_{CP}$ = 15.7 Hz, C3); **^{31}P NMR** (DMSO-d_6, δ in ppm): 23.0 (m); **elemental analysis:** calc. for $C_{14}H_{15}O_4P$: C 60.4, H 5.4, N 0.0, S 0.0; found: C 60.3, H 5.3, N 0.0, S 0.2.

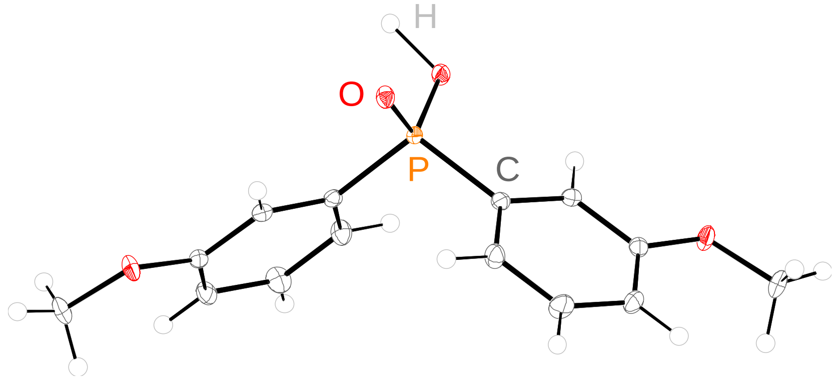

Fig. S 45. Molecular structure of bis(3-methoxyphenyl)phosphinic acid (**2e**); thermal ellipsoids are displayed at 50 % probability level; CCDC 2442185.

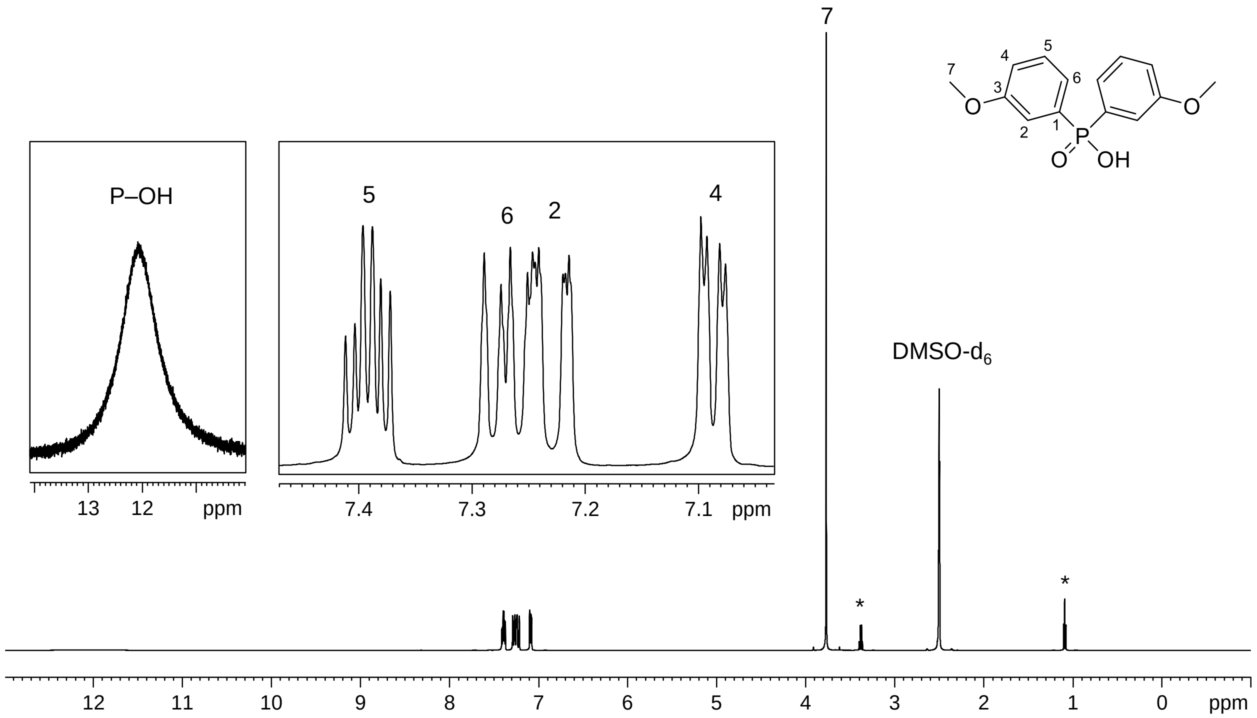

Fig. S 46. ^{1}H NMR spectrum (DMSO-d6, 300 K) of bis(3-methoxyphenyl)phosphinic acid (**2e**) synthesized from **1b**[OTf]; residual amounts of Et_2O from the NMR solvent are marked with asterisks (*).

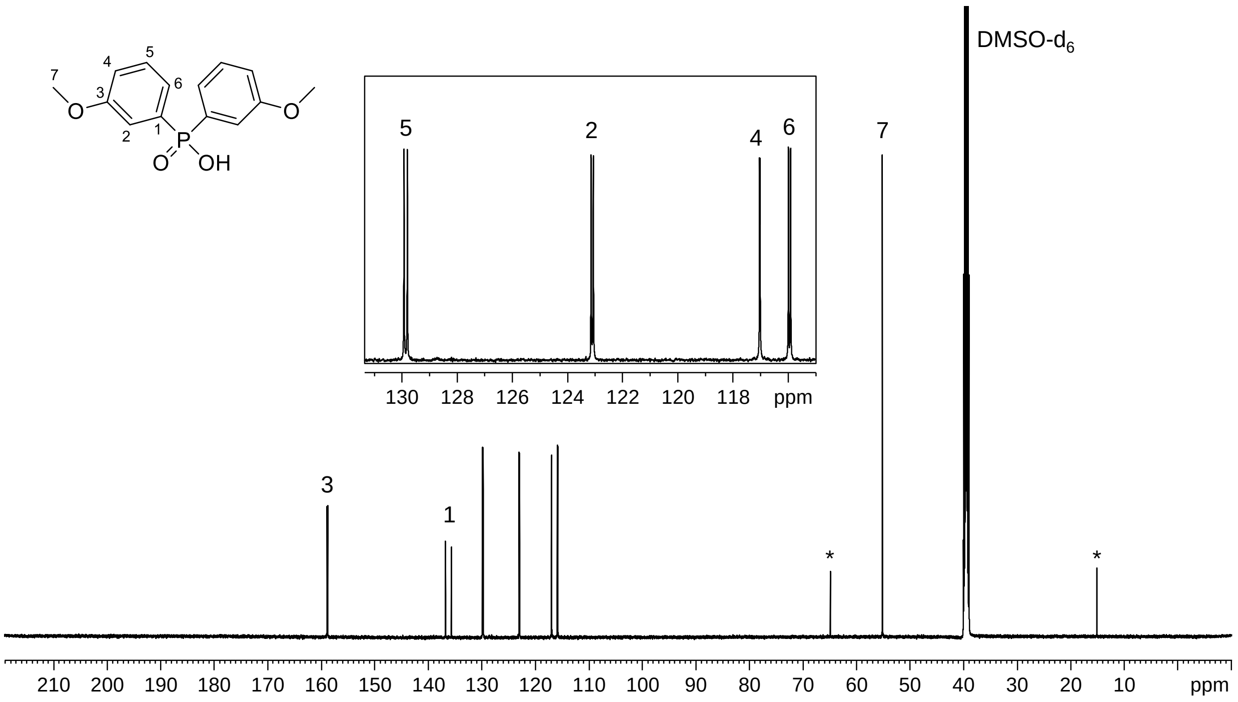

Fig. S 47. ^{13}C{^{1}H} NMR spectrum (DMSO-d6, 300 K) of bis(3-methoxyphenyl)phosphinic acid (**2e**) synthesized from **1b**[OTf]; residual amounts of Et_2O from the NMR solvent are marked with asterisks (*).

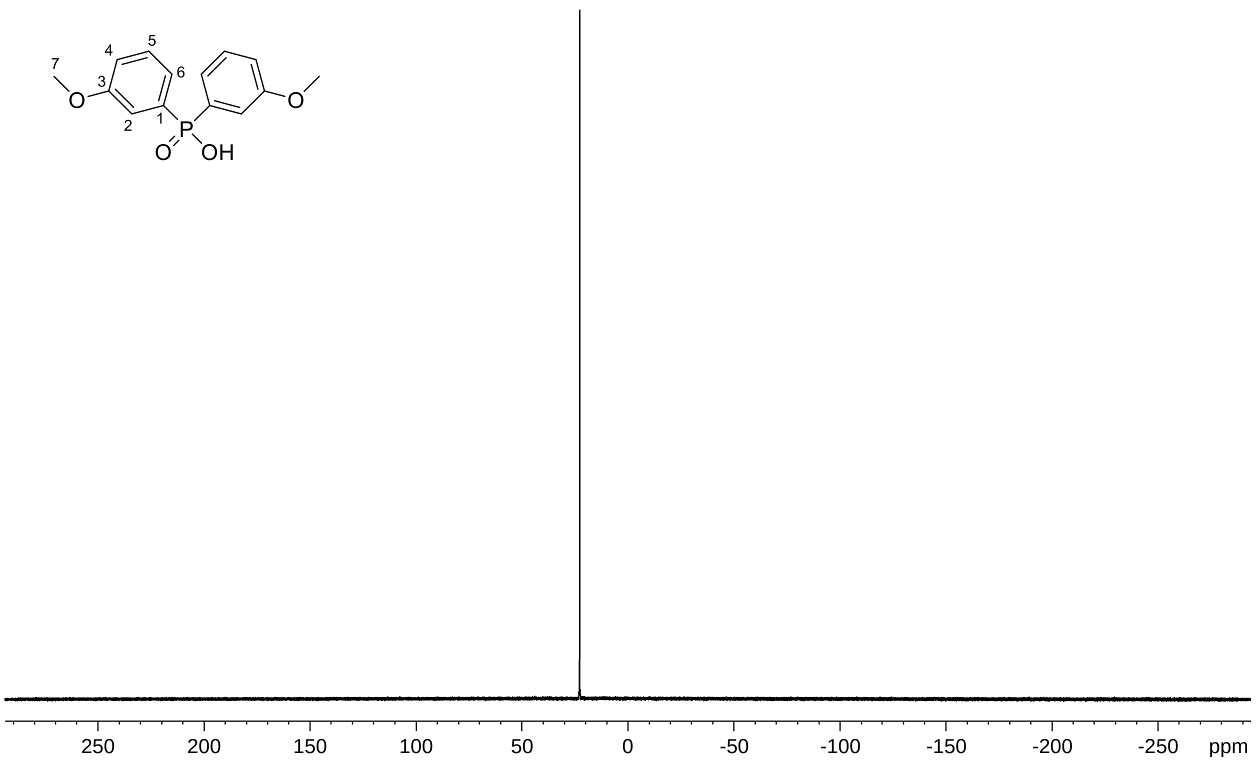

Fig. S 48. ^{31}P NMR spectrum (DMSO-d6, 300 K) of bis(3-methoxyphenyl)phosphinic acid (**2e**) synthesized from **1b**[OTf].

2.3.7. Preparation of Bis(4-fluorophenyl)phosphinic acid (**2f**)

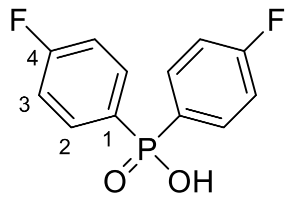

Bis(4-fluorophenyl)phosphinic acid (**2f**) was synthesized according to the general procedure from 1 mmol of $(DMAP)_2PO_2[OTf]$ (**1b**[OTf], 456 mg, 1 eq.) with 2.5 mmol of *p*-fluorophenylmagnesium bromide (1 M in THF, 2.5 mL, 2.5 eq.) and obtained as a colorless powder. powder.

Single crystals of **2f** suitable for single crystal structure determination were obtained by slow vapor diffusion of *n*-pentane into a concentrated $CHCl_3$ solution (Fig. S 49).

The NMR spectra of the obtained sample were in accordance with previously published analytical data.[8]

Yield: 153 mg (60 %); **Raman** ($\tilde{\nu}$ in cm^{-1}): 3073 (100), 1594 (51), 1232 (7), 1165 (27), 1134 (45), 1096 (7), 828 (75), 812 (34), 631 (38), 474 (5), 364 (7), 279 (8), 266 (7); **IR** (ATR, $\tilde{\nu}$ in cm^{-1}): 3104 (vw), 3072 (vw), 2052 (vw), 1912 (vw), 1641 (w), 1591 (m), 1498 (m), 1397 (w), 1302 (w), 1228 (m), 1197 (m), 1157 (m), 1129 (s), 1119 (s), 1097 (m), 1016 (w), 961 (s), 827 (s), 713 (m), 669 (s), 624 (w), 553 (w), 526 (vs), 485 (s), 452 (vs), 421 (m); **m.p.**: 132 – 134 °C (decomp.); **^{1}H NMR** ($CDCl_3$, δ in ppm): 7.03 (td, 4H, $^3J_{HH}$ = $^3J_{HF}$ = 13.0 Hz, H3), 7.64–7.71 (m, 4H, H2), 11.27 (s(br), 1H, P–OH); **^{13}C{^{1}H} NMR** ($CDCl_3$, δ in ppm): 115.9 (dd, $^2J_{CF}$ = 21.4 Hz, $^3J_{CP}$ = 14.7 Hz, C3), 128.5 (dd, $^1J_{CP}$ = 145.5 Hz, $^4J_{CF}$ = 3.5 Hz, C1), 133.9 (dd, $^2J_{CP}$ = 12.1 Hz, $^3J_{CF}$ = 8.9 Hz, C2), 165.2 (dd, $^1J_{CF}$ = 253.4 Hz, $^4J_{CP}$ = 3.5 Hz, C4); **^{19}F NMR** ($CDCl_3$, δ in ppm): – 106.4 (m); **^{31}P NMR** ($CDCl_3$, δ in ppm): 29.8 (m); **elemental analysis:** calc. for $C_{12}H_9F_2O_2P$: C 56.7, H 3.6, N 0.0, S 0.0; found: C 56.5, H 3.6, N 0.0, S 0.1.

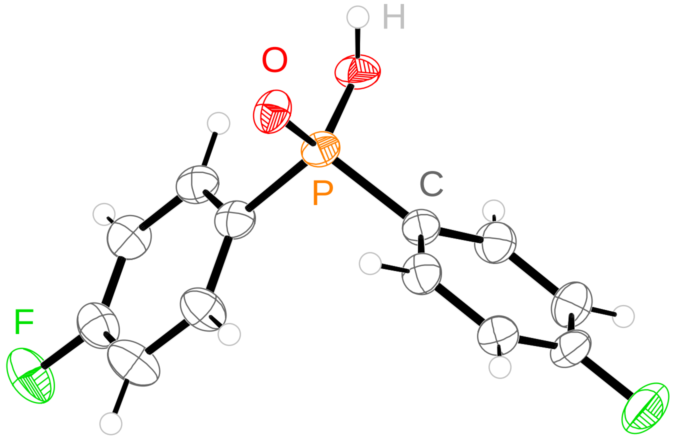

Fig. S 49. Molecular structure of bis(4-fluorophenyl)phosphinic acid (**2f**); thermal ellipsoids are displayed at 50 % probability level; CCDC 2442192.

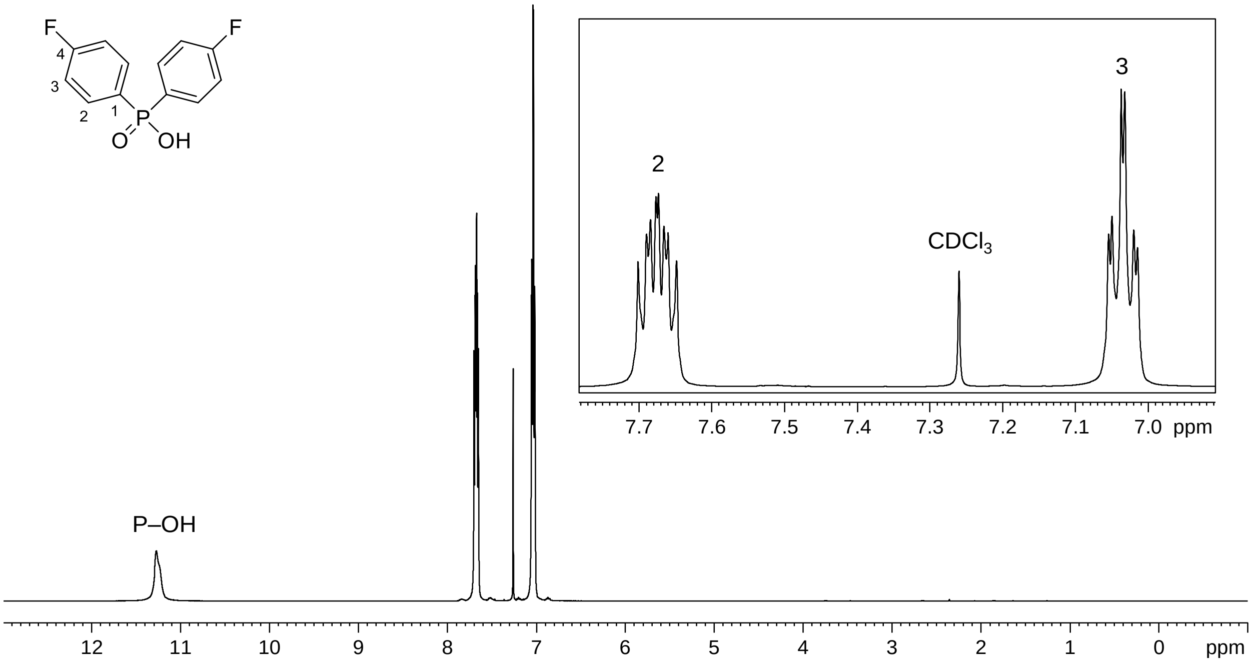

Fig. S 50. ^{1}H NMR spectrum ($CDCl_3$, 300 K) of bis(4-fluorophenyl)phosphinic acid (**2f**) synthesized from **1b**[OTf].

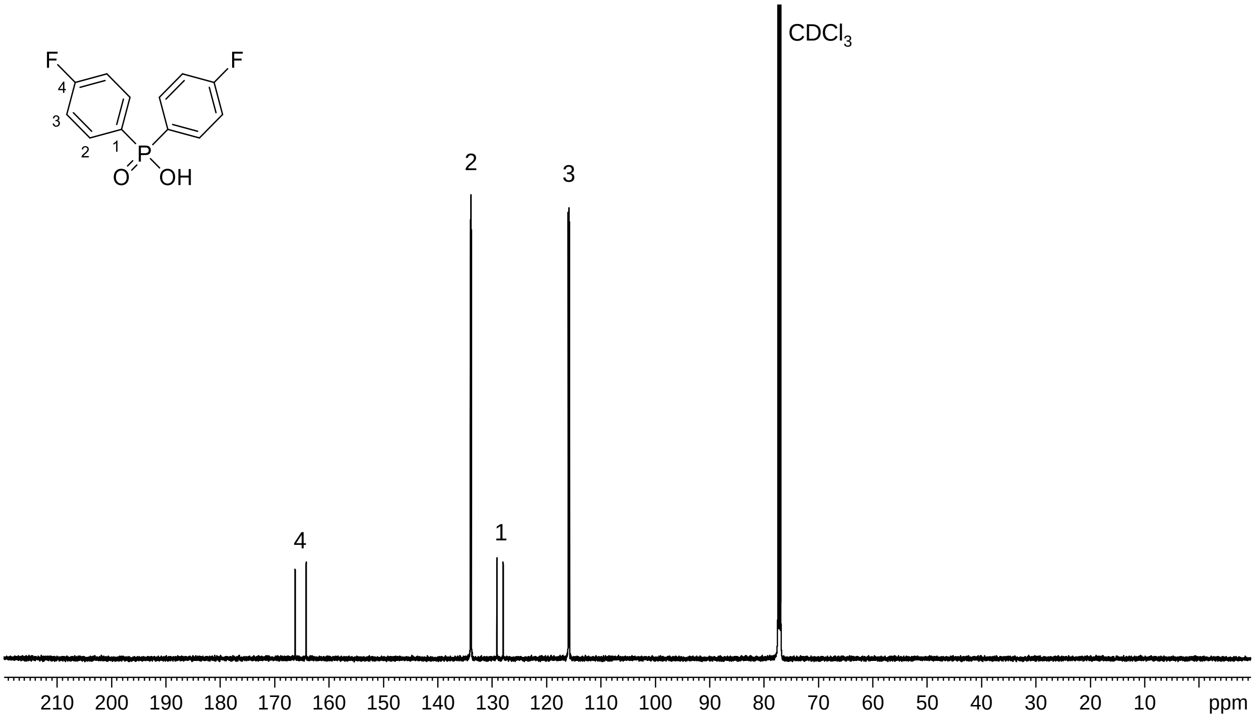

Fig. S 51. ^{13}C{^{1}H} NMR spectrum ($CDCl_3$, 300 K) of bis(4-fluorophenyl)phosphinic acid (**2f**) synthesized from **1b**[OTf].

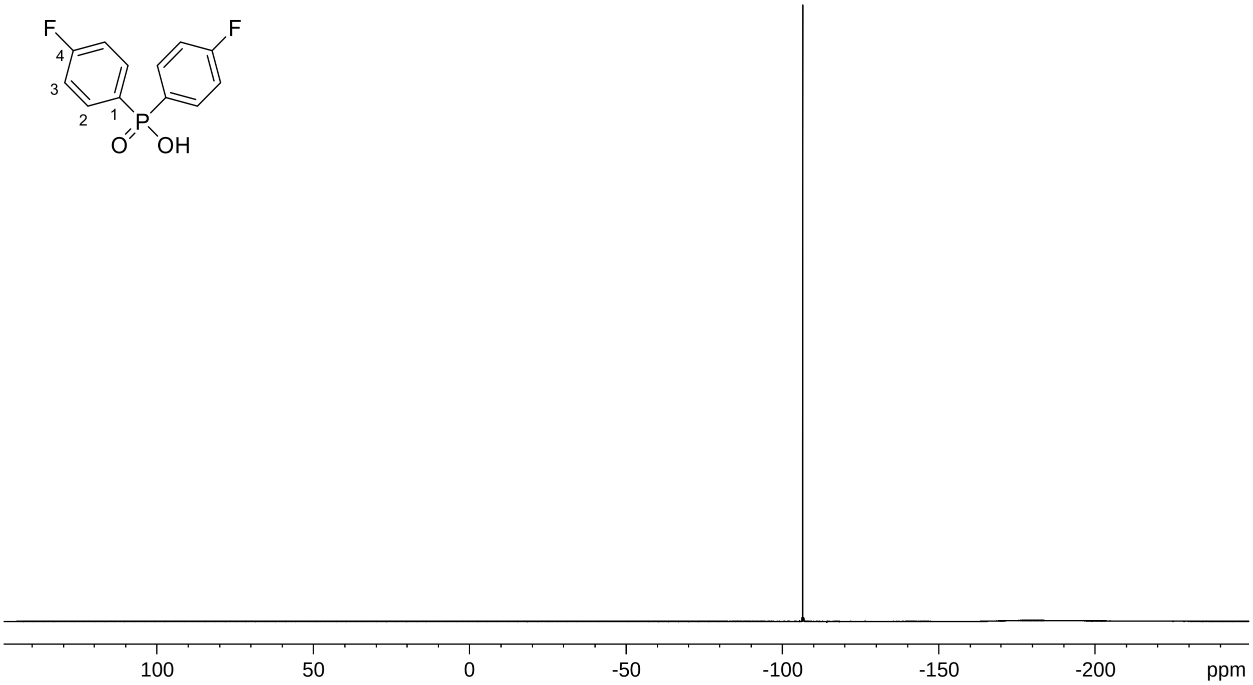

Fig. S 52. ^{19}F NMR spectrum ($CDCl_3$, 300 K) of bis(4-fluorophenyl)phosphinic acid (**2f**) synthesized from **1b**[OTf].

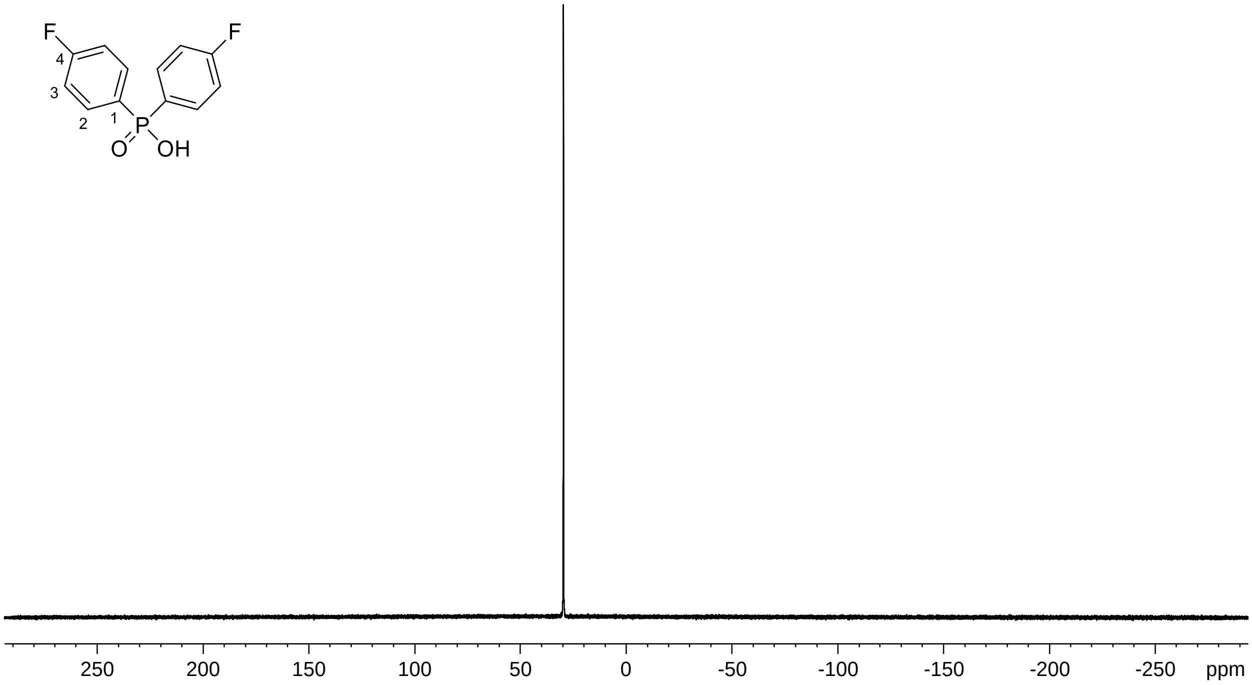

Fig. S 53. ^{31}P NMR spectrum ($CDCl_3$, 300 K) of bis(4-fluorophenyl)phosphinic acid (**2f**) synthesized from **1b**[OTf].

2.3.8. Preparation of Bis(3,5-bis(trifluoromethyl)phenyl)phosphinic acid (**2g**)

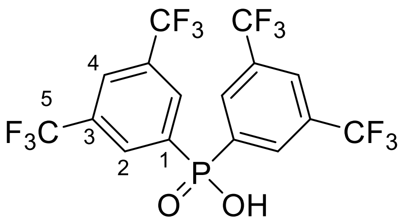

Bis(3,5-bis(trifluoromethyl)phenyl)phosphinic acid (**2g**) was synthesized according to the general procedure from 2 mmol of $(DMAP)_2PO_2[OTf]$ (**1b**[OTf], 456 mg, 1 eq.) with 5 mmol of the Grignard reagent and obtained as a colorless powder. The Grignard reagent was prepared priorly by slow addition of a solution of 1,3-bis(trifluoromethyl)-5-bromobenzene (0.86 mL, 5 mmol, 2.5 eq.) in 5 mL of Et_2O to a stirred suspension of dry Mg turnings (122 mg, 5 mmol, 2.5 eq.) in 5 mL of Et_2O, maintaining a gentle self-sustained reflux. Refluxing the mixture for an additional hour then gave a clear solution, which was used after cooling back to room temperature.

Single crystals of **2g** suitable for single crystal structure determination were obtained by slow vapor diffusion of cyclohexane into a concentrated solution in a CH_2Cl_2 / CH_3CN / ^{i}PrOH mixture over several weeks (Fig. S 54).

The NMR spectra of the obtained sample were in accordance with previously published analytical data.[9]

Yield: 569 mg (58 %); **Raman** ($\tilde{\nu}$ in cm^{-1}): 3093 (13), 1612 (14), 1367 (9), 1003 (23), 985 (12), 715 (19), 273 (73), 242 (78), 216 (100); **IR** (ATR, $\tilde{\nu}$ in cm^{-1}): 3093 (vw), 2640 (vw), 2246 (vw), 2099 (vw), 1837 (vw), 1621 (vw), 1612 (vw), 1461 (vw), 1440 (vw), 1364 (w), 1278 (s), 1227 (m), 1189 (m), 1171 (m), 1126 (vs), 1099 (s), 1005 (w), 985 (m), 966 (w), 930 (vw), 908 (m), 844 (w), 821 (vw), 755 (vw), 700 (m), 683 (s), 623 (w), 612 (m), 574 (vw), 544 (s), 532 (m), 506 (vw), 468 (m), 427 (w); **m.p.**: 223 – 225 °C (decomp.); **^{1}H NMR** (DMSO-d$_6$, δ in ppm): 8.27 (s, 2H, H4), 8.42 (d, 4H, $^3J_{HP}$ = 11.6 Hz, H2), 14.6 (s(br), 1H, P–OH); **^{13}C{^{1}H} NMR** (DMSO-d$_6$, δ in ppm): 123.0 (qd, $^1J_{CF}$ = 273.1 Hz, $^4J_{CP}$ = 1.5 Hz, C5), 125.5–125.7 (m, C4), 130.5 (qd, $^2J_{CF}$ = 33.1 Hz, $^3J_{CP}$ = 12.9 Hz, C3), 131.8–132.0 (m, C2), 138.2 (d, $^1J_{CP}$ = 135.5 Hz, C1); **^{19}F NMR** (DMSO-d$_6$, δ in ppm): – 61.5 (s) ; **^{31}P NMR** (DMSO-d$_6$, δ in ppm): 14.0 (quin, $^3J_{PH}$ = 11.6 Hz); **elemental analysis:** calc. for $C_{16}H_7F_{12}O_2P$: C 39.2, H 1.4, N 0.0, S 0.0; found: C 39.3, H 1.3, N 0.0, S 0.2.

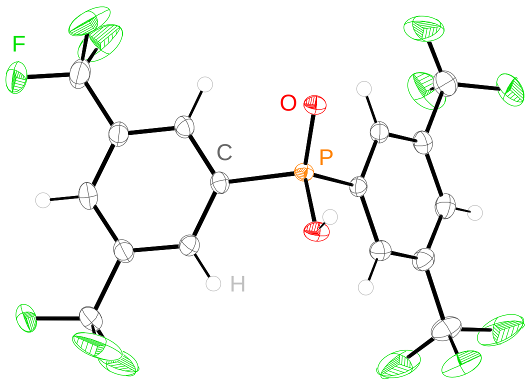

Fig. S 54. Molecular structure of bis(3,5-bis(trifluoromethyl)phenyl)phosphinic acid (**2g**); thermal ellipsoids are displayed at 50 % probability level; CCDC 2442202.

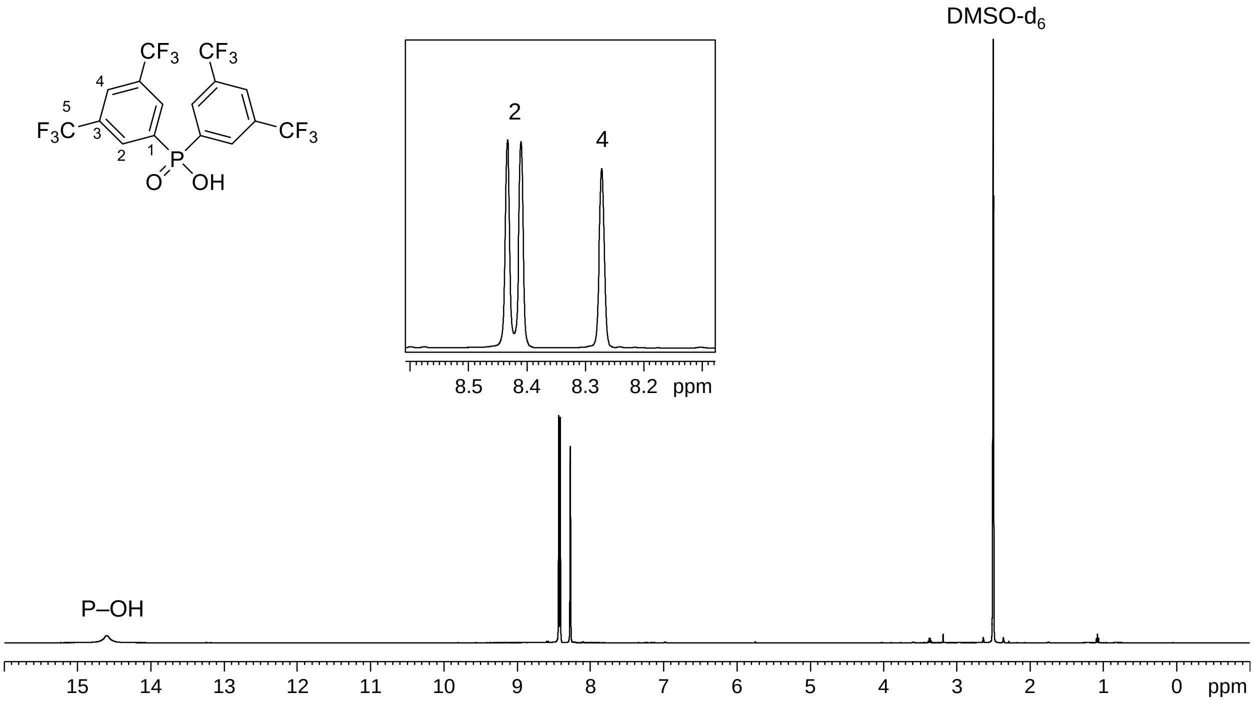

Fig. S 55. ^{1}H NMR spectrum (DMSO-d6, 300 K) of bis(3,5-bis(trifluoromethyl)phenyl)phosphinic acid (**2g**) synthesized from **1b**[OTf].

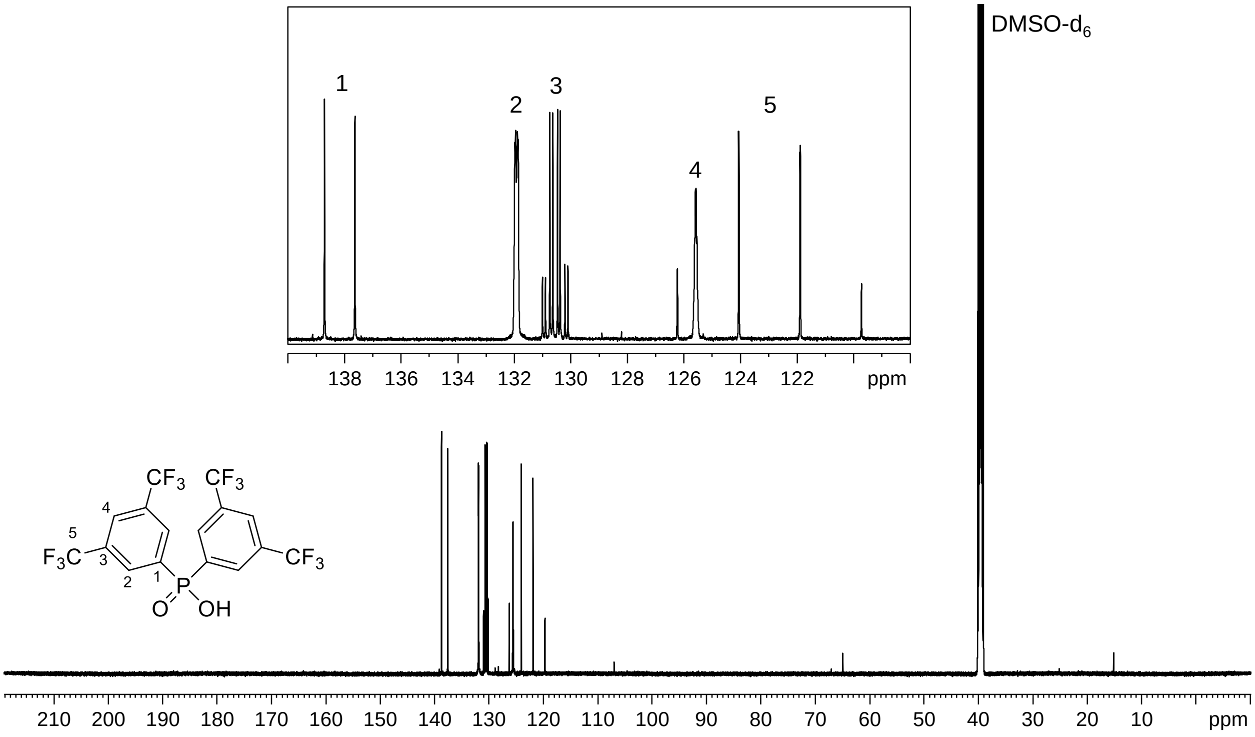

Fig. S 56. $^{13}C\{^{1}H\}$ NMR spectrum (DMSO-d6, 300 K) of bis(3,5-bis(trifluoromethyl)phenyl)phosphinic acid (**2g**) synthesized from **1b**[OTf].

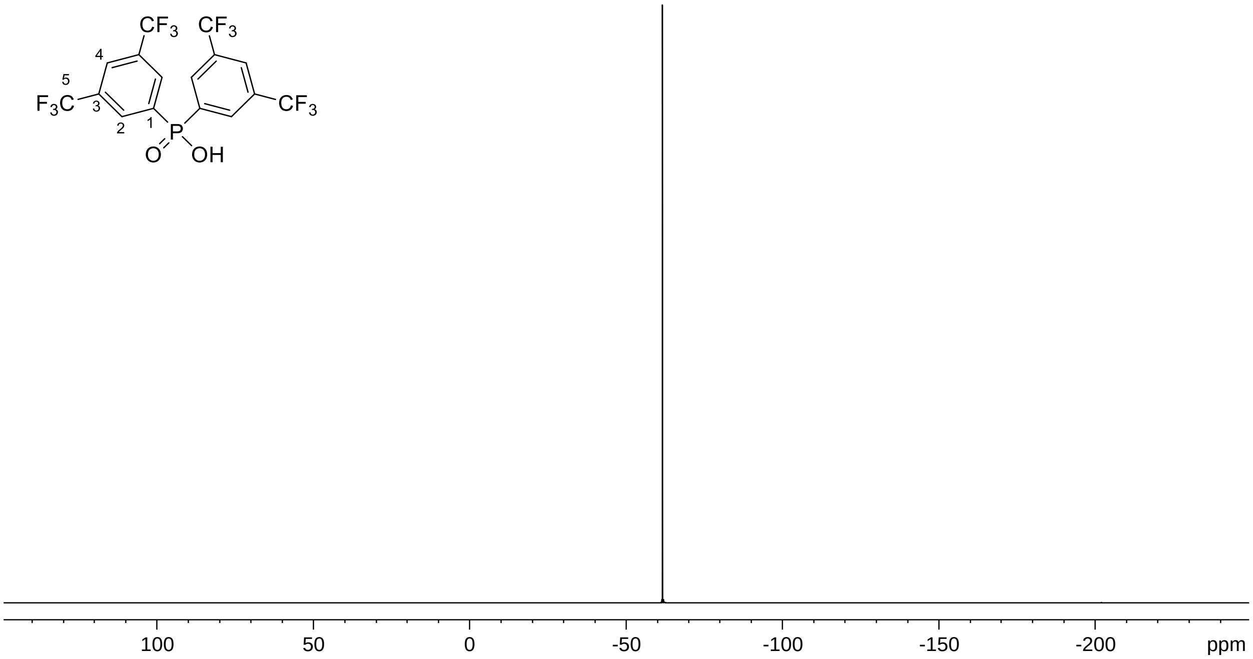

Fig. S 57. ^{19}F NMR spectrum (DMSO-d$_6$, 300 K) of bis(3,5-bis(trifluoromethyl)phenyl)phosphinic acid (**2g**) synthesized from **1b**[OTf].

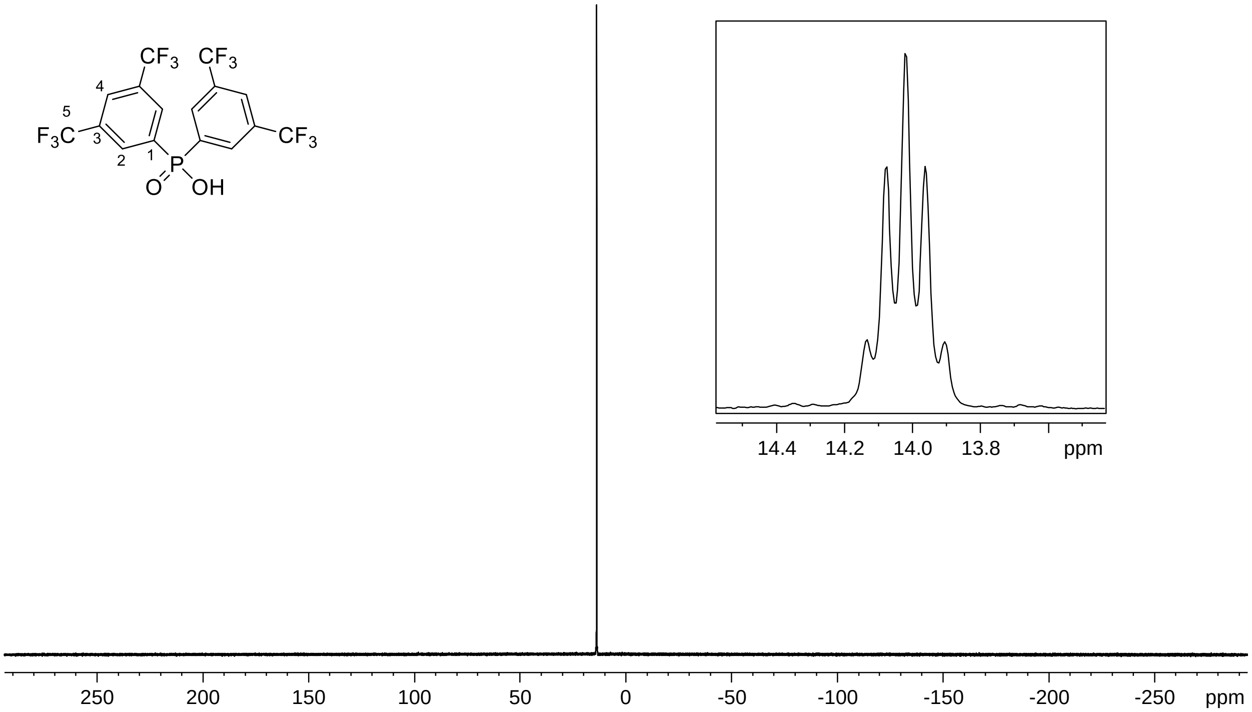

Fig. S 58. ^{31}P NMR spectrum (DMSO-d$_6$, 300 K) of bis(3,5-bis(trifluoromethyl)phenyl)phosphinic acid (**2g**) synthesized from **1b**[OTf].

2.3.9. Preparation of Bis(4-vinylphenyl)phosphinic acid (**2h**)

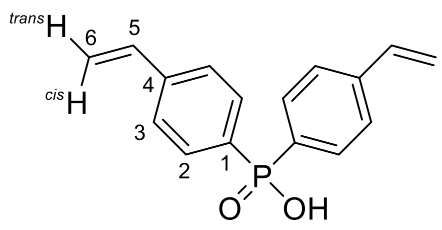

Bis(4-vinylphenyl)phosphinic acid (**2h**) was synthesized according to the general procedure from 2 mmol of $(DMAP)_2PO_2[OTf]$ (**1b**[OTf], 456 mg, 1 eq.) with 5 mmol of the Grignard reagent and obtained as an off-white powder. The Grignard reagent was prepared priorly by slow addition of 1-bromo-4-vinylbenzene (0.66 mL, 5 mmol, 2.5 eq.) to a stirred suspension of dry Mg turnings (122 mg, 5 mmol, 2.5 eq.) in 10 mL of THF and subsequent reflux for 1 h to obtain a clear solution, which was used after cooling back to room temperature. Single crystals of **2h** suitable for single crystal structure determination were obtained by slow vapor diffusion of *n*-pentane into a concentrated $CHCl_3$ solution (Fig. S 59).
Although compound **2h** has previously been mentioned in the literature,[10] no set of published analytical data was found.
During melting point determination with the standard melting point device, no melting was observed until 400 °C. For further verification, the compound was subjected to STA measurement (Fig. S 60), which showed an exothermic process at 180 °C and decomposition above 362 °C.
Yield: 347 mg (64 %); **Raman** ($\tilde{\nu}$ in cm^{-1}): 3063 (11), 3006 (9), 1630 (91), 1600 (100), 1422 (16), 1398 (6), 1317 (6), 1296 (5), 1205 (11), 1186 (11), 1161 (18), 1108 (9), 794 (6), 635 (5), 81 (51); **IR** (ATR, $\tilde{\nu}$ in cm^{-1}): 3004 (vw), 2615 (w), 2313 (w), 2151 (vw), 1853 (vw), 1629 (vw), 1598 (w), 1555 (w), 1499 (vw), 1398 (w), 1211 (s), 1200 (s), 1191 (s), 1184 (s), 1128 (m), 1118 (m), 1105 (s), 1028 (w), 1016 (w), 989 (w), 957 (s), 941 (s), 924 (s), 857 (m), 843 (s), 833 (m), 805 (m), 761 (w), 745 (w), 660 (vs), 635 (m), 621 (s), 542 (s), 519 (s), 497 (s), 480 (m), 440 (m), 417 (w); **m.p.**: n.a.; **^{1}H NMR** ($CDCl_3$, δ in ppm): 5.33 (d, 2H, $^3J_{HH}$ = 10.9 Hz, ^{trans}H6), 5.79 (d, 2H, $^3J_{HH}$ = 17.6 Hz, ^{cis}H6), 6.67 (dd, 2H, $^3J_{HH}$ = 17.6 / 10.9 Hz, H5), 7.35 (dd, 4H, $^3J_{HH}$ = 8.1 Hz, $^4J_{HP}$ = 3.2 Hz, H3), 7.64 (dd, 4H, $^3J_{HP}$ = 12.3 Hz, $^3J_{HH}$ = 8.1 Hz, H2), 7.96 (s(br), 1H, P–OH); **^{13}C{^{1}H} NMR** ($CDCl_3$, δ in ppm): 116.4 (s, C6), 126.2 (d, $^3J_{CP}$ = 13.8 Hz, C3), 131.6 (d, $^2J_{CP}$ = 10.9 Hz, C2), 131.8 (d, $^1J_{CP}$ = 142.6 Hz, C1), 136.2 (d, $^5J_{CP}$ = 1.4 Hz, C5), 141.0 (d, $^4J_{CP}$ = 3.0 Hz, C4); **^{31}P NMR** ($CDCl_3$, δ in ppm): 32.7 (m); **elemental analysis:** calc. for $C_{16}H_{15}O_2P \cdot 0.2\ H_2O$: C 70.2, H 5.7, N 0.0, S 0.0; found: C 70.0, H 5.7, N 0.4, S 0.0.

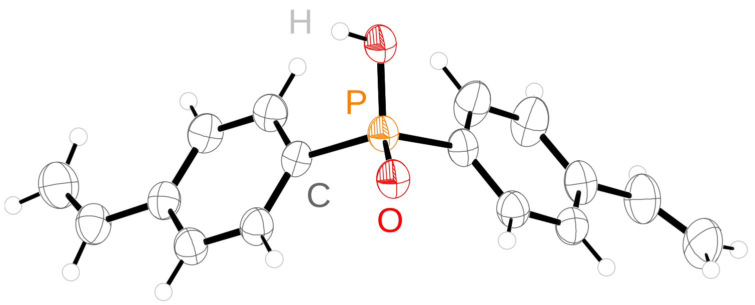

Fig. S 59. Molecular structure of bis(4-vinylphenyl)phosphinic acid (**2h**); thermal ellipsoids are displayed at 50 % probability level; CCDC 2442201.

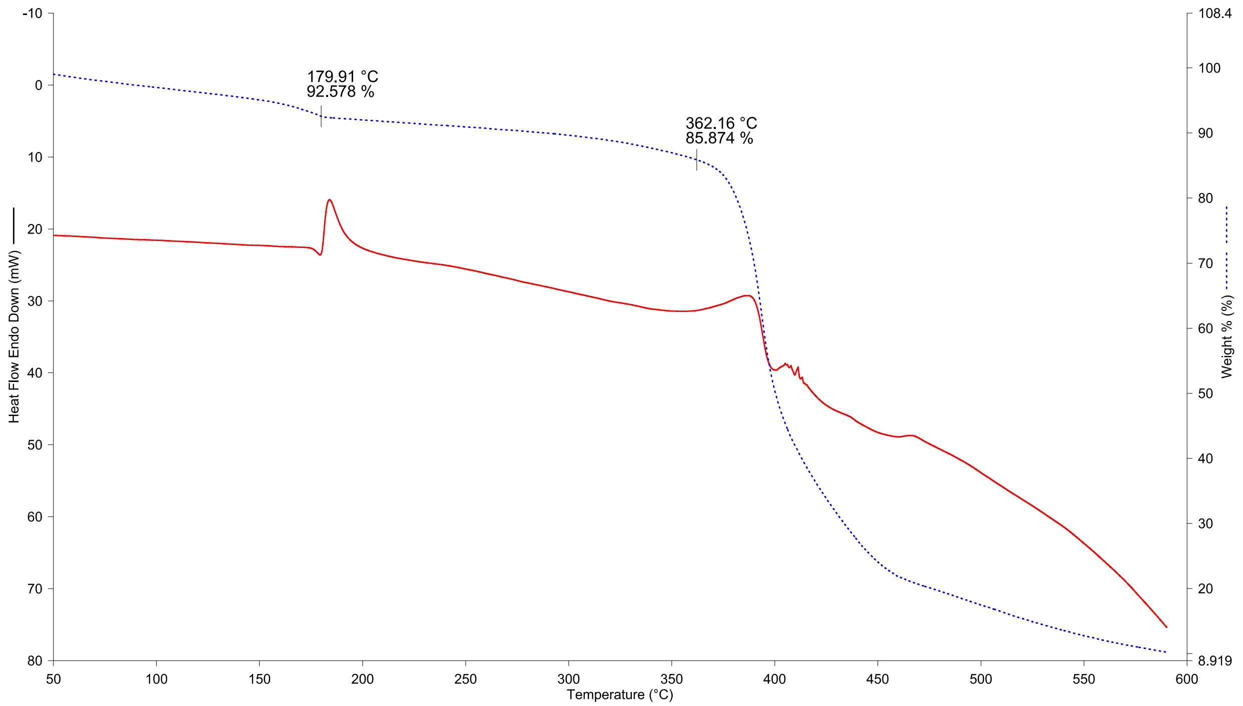

Fig. S 60. Simultaneous thermogravimetric analysis (STA) of bis(4-vinylphenyl)phosphinic acid (**2h**); Thermogravimetric analysis (TGA) is depicted in blue and differential scanning calorimetry (DSC) in red; heating rate was 10.00 °C/min from 35.00 °C to 600.00 °C.

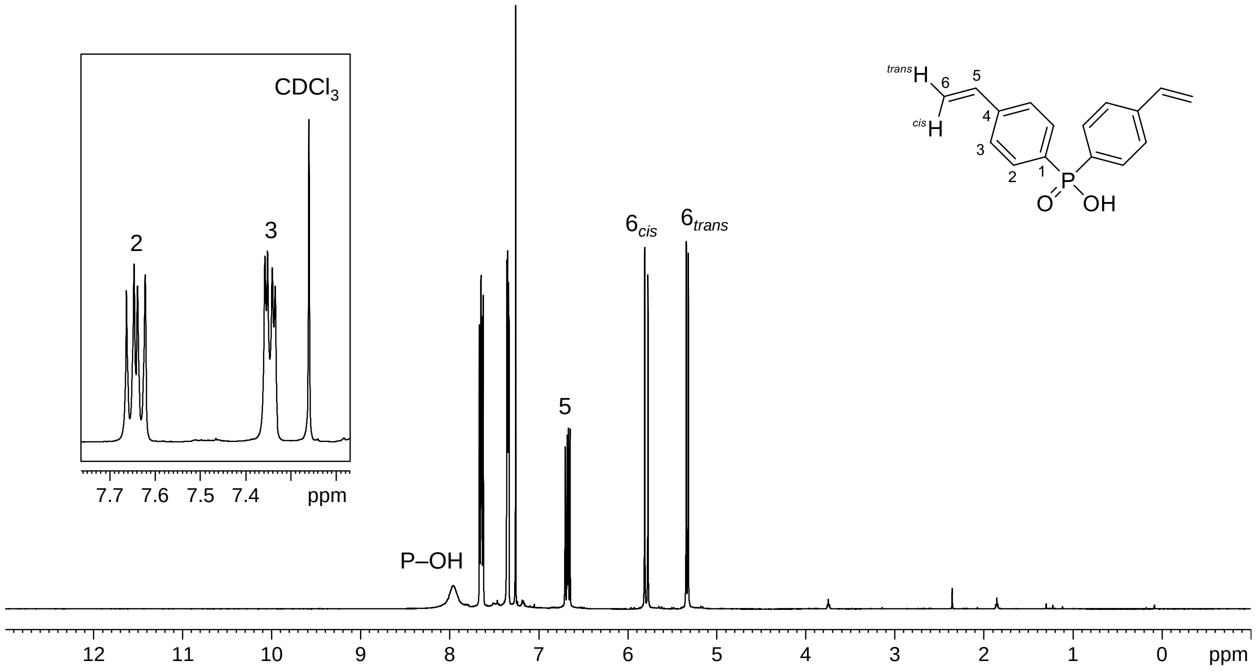

Fig. S 61. ^{1}H NMR spectrum ($CDCl_3$, 300 K) of bis(4-vinylphenyl)phosphinic acid (**2h**) synthesized from **1b**[OTf].

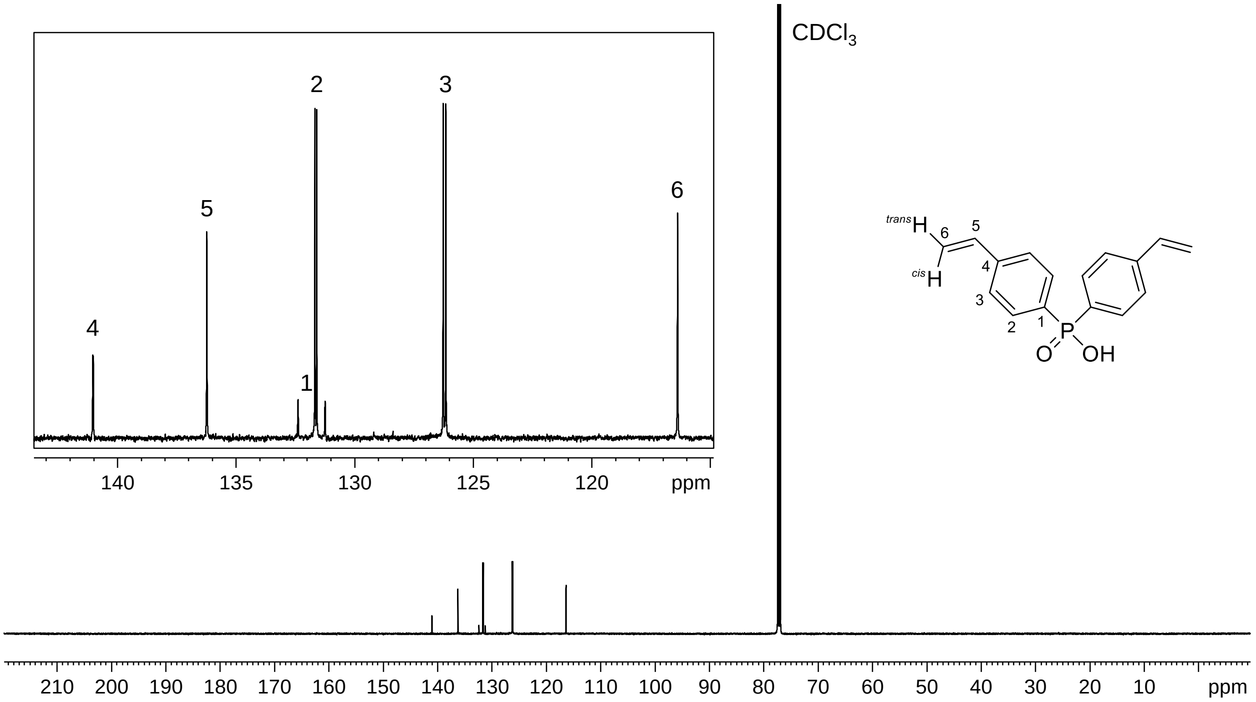

Fig. S 62. $^{13}C\{^{1}H\}$ NMR spectrum ($CDCl_3$, 300 K) of bis(4-vinylphenyl)phosphinic acid (**2h**) synthesized from **1b**[OTf].

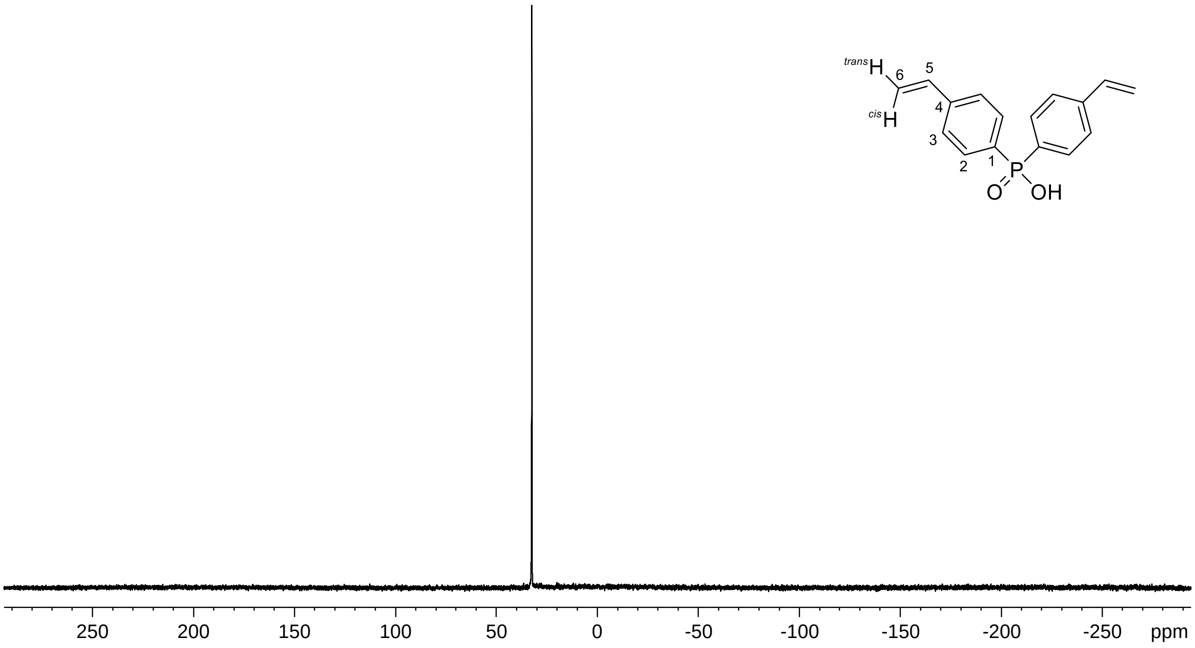

Fig. S 63. ^{31}P NMR spectrum ($CDCl_3$, 300 K) of bis(4-vinylphenyl)phosphinic acid (**2h**) synthesized from **1b**[OTf].

2.4. Preparation of Dialkynylphosphinates

2.4.1. Preparation of Bis(trimethylsilylethynyl)phosphinic acid (**9a**)

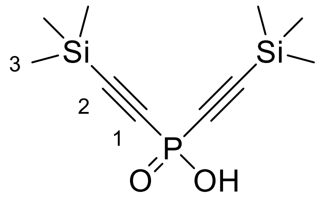

To a stirred solution of trimethylsilylacetylene (1.964 g, 20 mmol, 4 eq.) in 20 mL of THF was slowly added ethylmagnesium bromide (3 M in Et_2O, 4.2 mL, 12.5 mmol, 2.5 eq.) over the course of 10 min, resulting in visible gas formation. The mixture was continued to stir for 1 h before slow addition of $(DMAP)_2PO_2$[OTf] (**1b**[OTf], 2.282 g, 5 mmol, 1 eq.) over the course of 30 min, during which warming of the suspension can be observed. After further stirring for 2 h, the suspension was diluted with 10 mL of toluene and subsequently quenched with 20 mL of 1 M HCl. The organic phase was then separated from the mixture and the remaining aqueous phase extracted with toluene two more times. The combined organic phases were evaporated to dryness and the obtained residue extracted with Et_2O three times. The combined and filtered Et_2O extracts were again evaporated to dryness, yielding a yellow-colored oil. Upon addition of 8 mL of *n*-pentane to this oil, the product readily crystallized as colorless rods from the mixture, which were suitable for single crystal structure determination (Fig. S 64). After leaving the mixture to crystallize for another 30 min, the solvent was decanted off after centrifugation and the remaining solid dried *in vacuo* to obtain **9a** as a colorless crystalline powder.

Yield: 1.161 g (90 %); **Raman** ($\tilde{\nu}$ in cm^{-1}): 2968 (25), 2903 (38), 2131 (100), 761 (5), 630 (30), 218 (10), 153 (9), 122 (10), 75 (8); **IR** (ATR, $\tilde{\nu}$ in cm^{-1}): 2964 (vw), 2902 (vw), 2323 (vw), 2131 (vw), 1563 (vw), 1411 (vw), 1252 (w), 1210 (m), 1132 (w), 1103 (vw), 985 (m), 842 (vs), 803 (vs), 772 (s), 758 (vs), 705 (m), 580 (w), 527 (s), 435 (w); **m.p.**: 133 – 136 °C (decomp.); **^{1}H NMR** (CD_2Cl_2, δ in ppm): 0.27 (s, 18H, H3), 13.42 (s(br), 1H, P–OH); **^{13}C{^{1}H} NMR** (CD_2Cl_2, δ in ppm): – 0.9 (s, C3), 98.9 (d, $^{1}J_{CP}$ = 247.4 Hz, C1), 110.9 (d, $^{2}J_{CP}$ = 37.9 Hz, C2); **^{29}Si NMR** (CD_2Cl_2, δ in ppm): – 13.9 (d, $^{3}J_{SiP}$ = 4.2 Hz); **^{31}P NMR** (CD_2Cl_2, δ in ppm): – 22.7 (s); **elemental analysis:** calc. for $C_{10}H_{19}O_2PSi_2$: C 46.5, H 7.4, N 0.0, S 0.0; found: C 46.2, H 7.1, N 0.0, S 0.0.

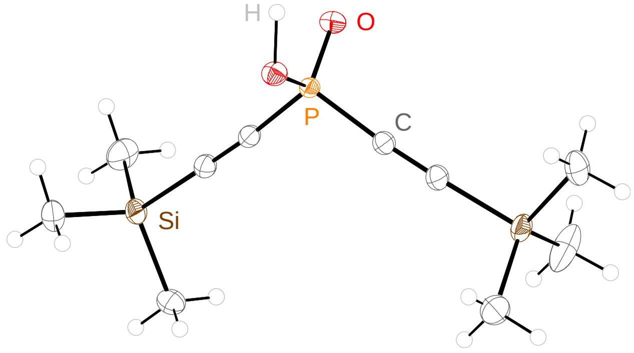

Fig. S 64. Molecular structure of bis(trimethylsilylethynyl)phosphinic acid (**9a**); thermal ellipsoids are displayed at 50 % probability level; CCDC 2442198.

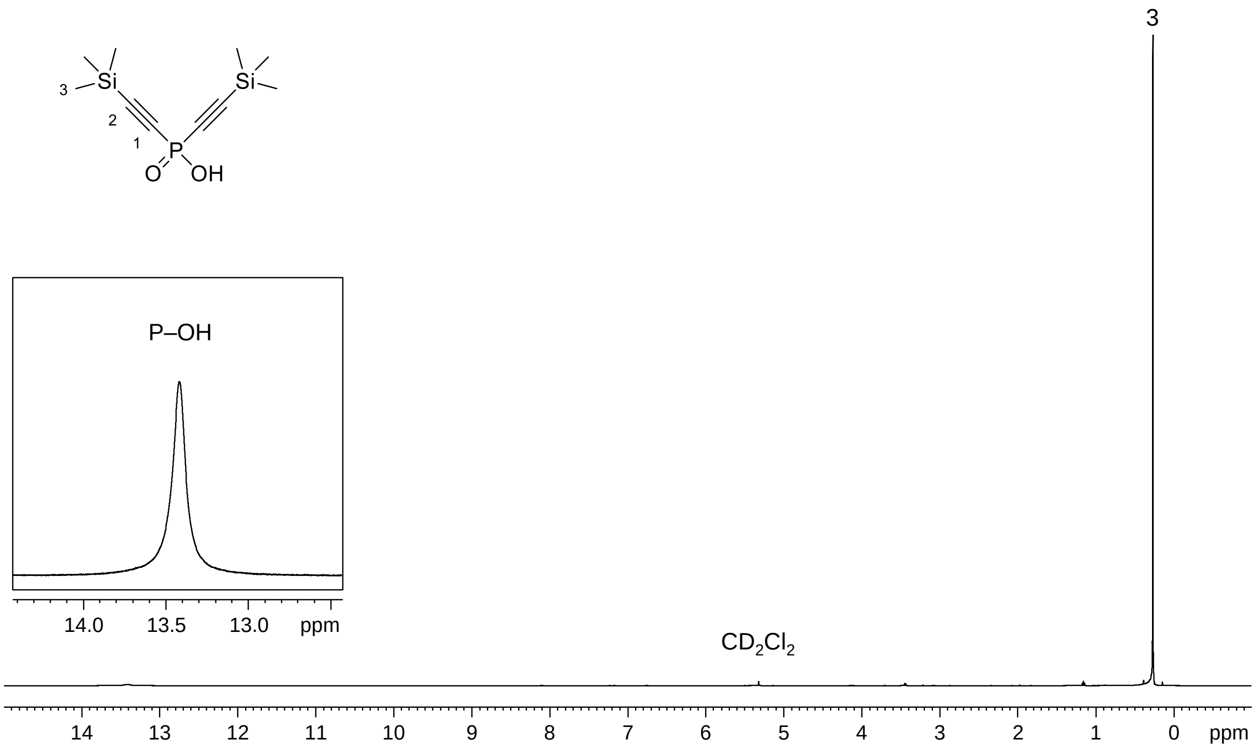

Fig. S 65. ^{1}H NMR spectrum (CD_2Cl_2, 300 K) of bis(trimethylsilylethynyl)phosphinic acid (**9a**) synthesized from **1b**[OTf].

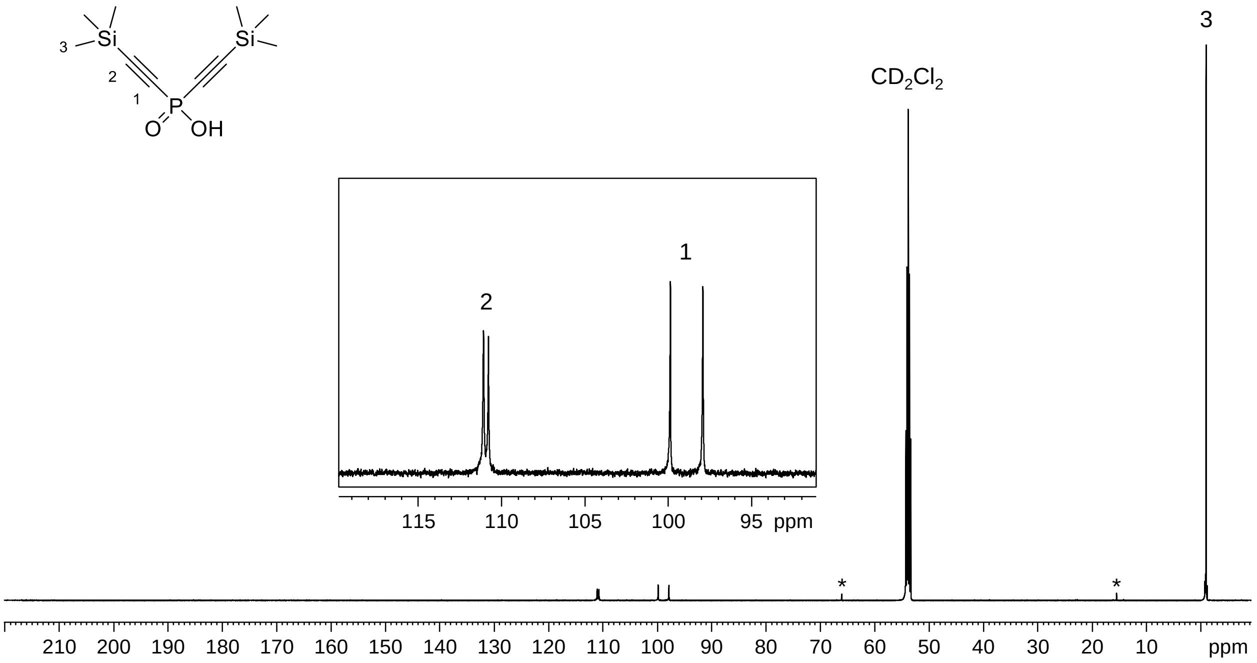

Fig. S 66. ^{13}C{^{1}H} NMR spectrum (CD_2Cl_2, 300 K) of bis(trimethylsilylethynyl)phosphinic acid (**9a**) synthesized from **1b**[OTf]; Residual amounts of Et_2O are marked with asterisks (*).

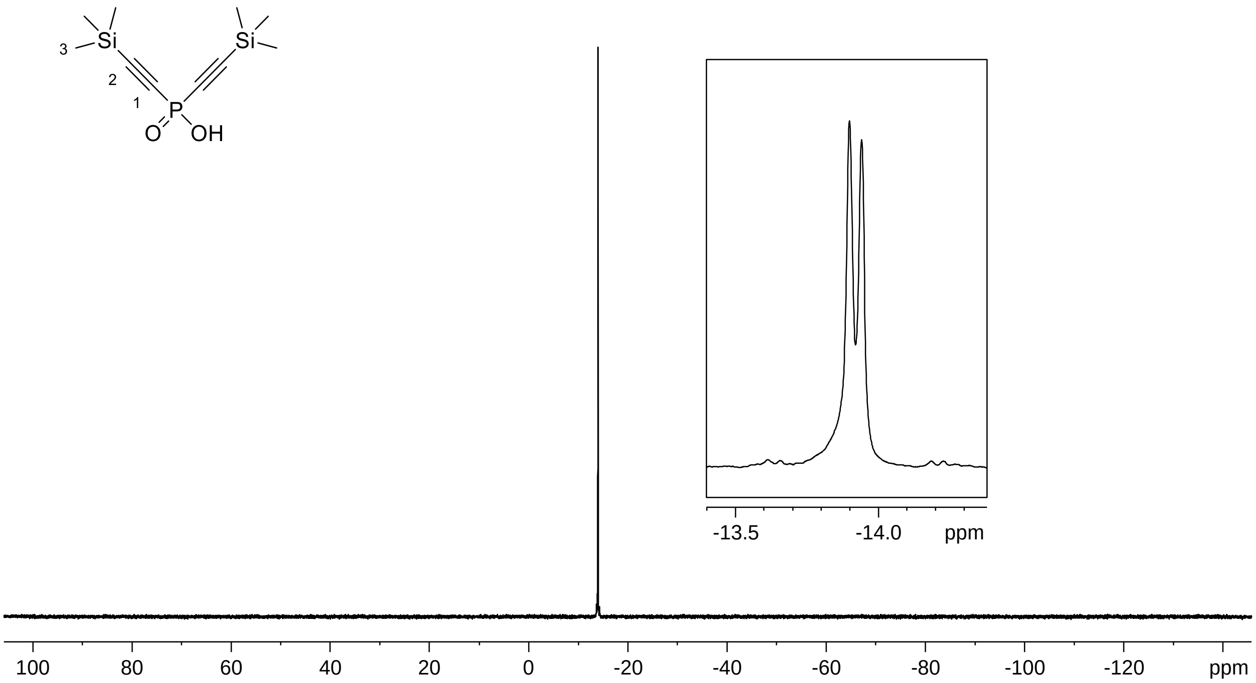

Fig. S 67. $^{29}Si\{^{1}H\}$ DEPT-19.5 NMR spectrum (CD_2Cl_2, 300 K) of bis(trimethylsilylethynyl)phosphinic acid (**9a**) synthesized from **1b**[OTf].

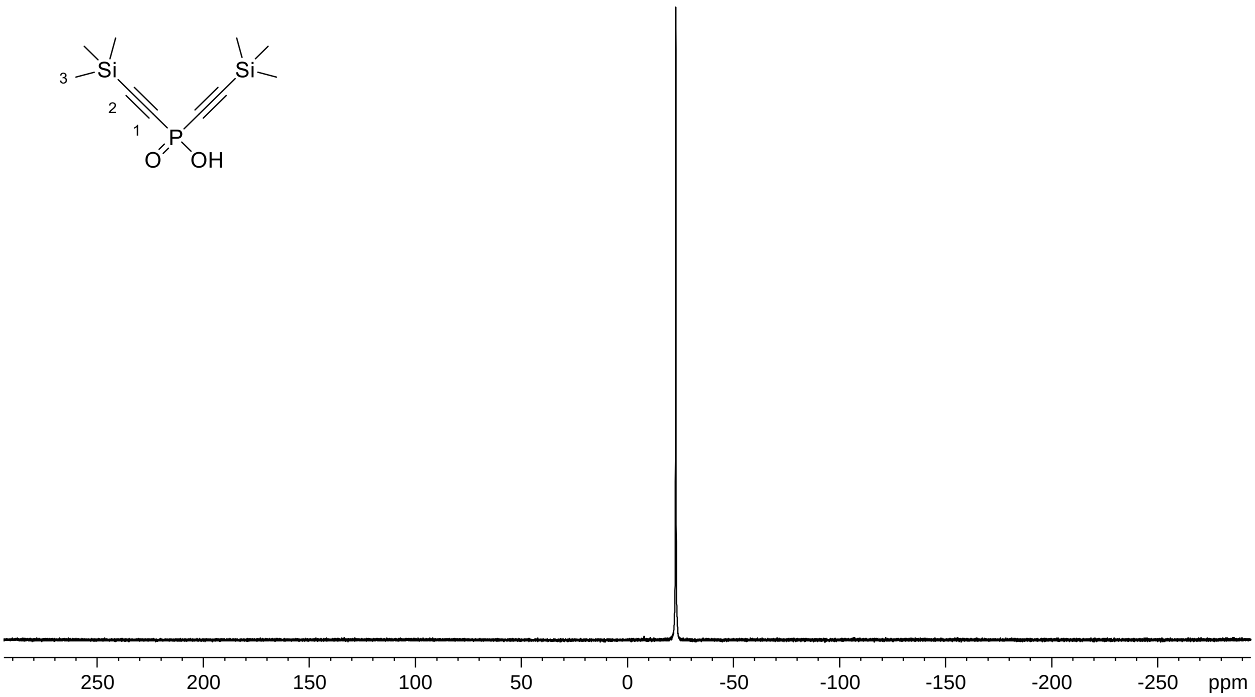

Fig. S 68. ^{31}P NMR spectrum (CD_2Cl_2, 300 K) of bis(trimethylsilylethynyl)phosphinic acid (**9a**) synthesized from **1b**[OTf].

2.4.2. General procedure for compounds **9b-k**

For synthesis of the dialkynylphosphinic acids, the Grignard reagent was first prepared *in situ* by slow addition of ethylmagnesium bromide (3 M in Et_2O, 0.83 mL, 2.5 mmol, 2,5 eq.) to a stirred solution of the corresponding terminal alkyne (3 mmol, 3 eq.) in 10 mL of THF, resulting in visible gas formation. After stirring the mixture for an additional hour, $(DMAP)_2PO_2[OTf]$ (**1b**[OTf], 456 mg, 1 mmol, 1 eq.) was slowly added. The obtained suspension cleared up visibly within 10 to 30 minutes and was further stirred for 4 to 16 hours before quenching with 1 M HCl. The organic phase was then separated from the mixture and the remaining aqueous phase extracted with $CHCl_3$ three more times. The combined organic phases were evaporated to dryness, re-dissolved in $CHCl_3$ and washed with acidified brine (0.1 M HCl) twice. To assess the target compound's solubility in alkaline aqueous solution, an aliquot of 1 mL was taken, shaken with the same amount of 1 M NaOH and centrifuged. If two clear layers were obtained, the steps listed for "Case A" were performed, for samples with formation of precipitate between the layers, the steps listed for "Case B" were performed (see Table S 4).

<u>Case A:</u>
The crude product solution in $CHCl_3$ was re-extracted with 1 M NaOH for three times. Subsequent acidification of the combined aqueous phases (pH = 1) with conc. HCl led to precipitation of the dialkynylphosphinic acid, which was obtained as a colorless powder after filtration, washing with 1 M HCl and drying *in vacuo*. Phosphinic acids with alkylalkynyl substituents (compounds **9j** and **9k**) instead separated as colorless oils from the aqueous solution and were isolated by careful phase separation followed by extracting the aqueous phase twice with small amounts of $CHCl_3$. The $CHCl_3$ was then evaporated *in vacuo* and the obtained oil dissolved in 1 M ammonia solution. Subsequent evaporation of the solvent *in vacuo* gave the corresponding ammonium phosphinate as a colorless solid.

<u>Case B:</u>
To the crude product solution in $CHCl_3$ was slowly added 15 mL of 1 M NaOH while stirring vigorously, resulting in formation of a turbid emulsion with colorless precipitate. The precipitate was filtered off, washed with 1 M NaOH and then suspended in 1 HCl. The obtained mixture was placed in an ultrasonic bath for about 10 min and subsequently stirred for another hour before filtering off the precipitate again. After washing with 1 M HCl and drying *in vacuo*, the dialkynylphosphinic acid was obtained as a colorless powder.

Analytical details and individual information about the synthetic procedure are provided in sections 2.4.3 to 2.4.12.

Table S 4. Details on used Grignard reagents for the synthesis of dialkynylphosphinic acids.

Grignard reagent	Workup procedure
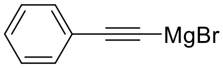	A
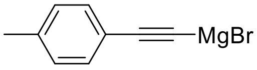	B
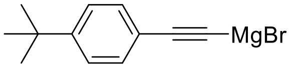	B
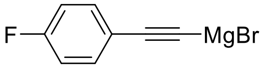	B
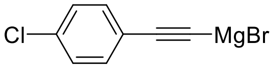	B
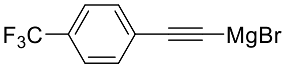	B
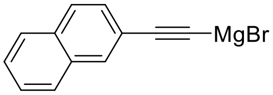	B
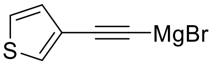	A
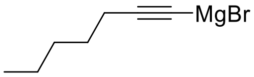	A
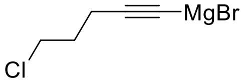	A

2.4.3. Preparation of Bis(phenylethynyl)phosphinic acid (**9b**)

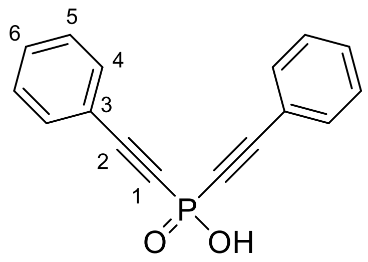

Bis(phenylethynyl)phosphinic acid (**9b**) was obtained as a colorless powder from synthesis according to the general procedure (case A) with $(DMAP)_2PO_2[OTf]$ (**1b**[OTf], 456 mg, 1 mmol, 1 eq.), phenylacetylene (330 µL, 3 mmol, 3 eq.) and ethylmagnesium bomide (3 M in Et_2O, 0.83 mL, 2.5 mmol, 2.5 eq.).

Single crystals of **9b** suitable for single crystal structure determination were obtained by slow evaporation of Et_2O from a concentrated solution (Fig. S 69).

Due to its hygroscopy, samples of **9b** for elemental analysis were dried *in vacuo* over P_2O_5 prior to measurement. The obtained values suggest an approximate residual water content of 10 mol%.

Yield: 217 mg (82 %); **Raman** ($\tilde{\nu}$ in cm^{-1}): 2183 (100), 1595 (54), 1230 (11), 999 (18), 96 (16), 78 (23); **IR** (ATR, $\tilde{\nu}$ in cm^{-1}): 3061 (vw), 2479 (vw), 2284 (vw), 2173 (s), 1621 (w), 1575 (w), 1488 (w), 1442 (w), 1283 (vw), 1228 (m), 1208 (m), 1161 (m), 1093 (w), 1070 (w), 1002 (s), 975 (vs), 929 (m), 864 (vs), 841 (s), 753 (vs), 684 (vs), 670 (vs), 625 (s), 538 (vs), 511 (m), 482 (vs), 465 (vs); **m.p.**: 148 – 151 °C (decomp.); **^{1}H NMR** (CD_2Cl_2, δ in ppm): 7.35 (t, 4H, $^{3}J_{HH}$ = 7.7 Hz, H5), 7.46 (t, 2H, $^{3}J_{HH}$ = 7.6 Hz, H6), 7.58–7.61 (m, 4H, H4), 10.20 (s(br), 1H, P–OH); **^{13}C{^{1}H} NMR** (CD_2Cl_2, δ in ppm): 83.0 (d, $^{1}J_{CP}$ = 276.3 Hz, C1), 100.8 (d, $^{2}J_{CP}$ = 53.0 Hz, C2), 119.7 (d, $^{3}J_{CP}$ = 5.4 Hz, C3), 129.0 (s, C5), 131.4 (s, C6), 133.2 (d, $^{4}J_{CP}$ = 2.3 Hz, C4); **^{31}P NMR** (CD_2Cl_2, δ in ppm): – 18.0 (s); **elemental analysis:** calc. for $C_{16}H_{11}O_2P \cdot 0.1\ H_2O$: C 71.7, H 4.2, N 0.0, S 0.0; found: C 71.4, H 4.1, N 0.0, S 0.0.

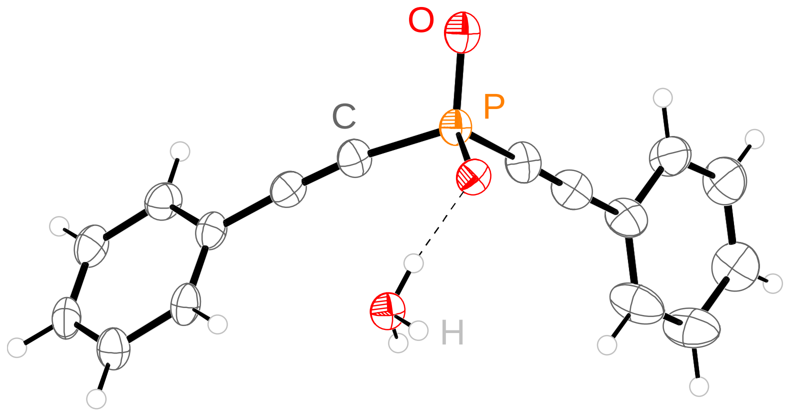

Fig. S 69. Molecular structure of bis(phenylethynyl)phosphinic acid (**9b**) · H_2O; thermal ellipsoids are displayed at 50 % probability level; CCDC 2442204.

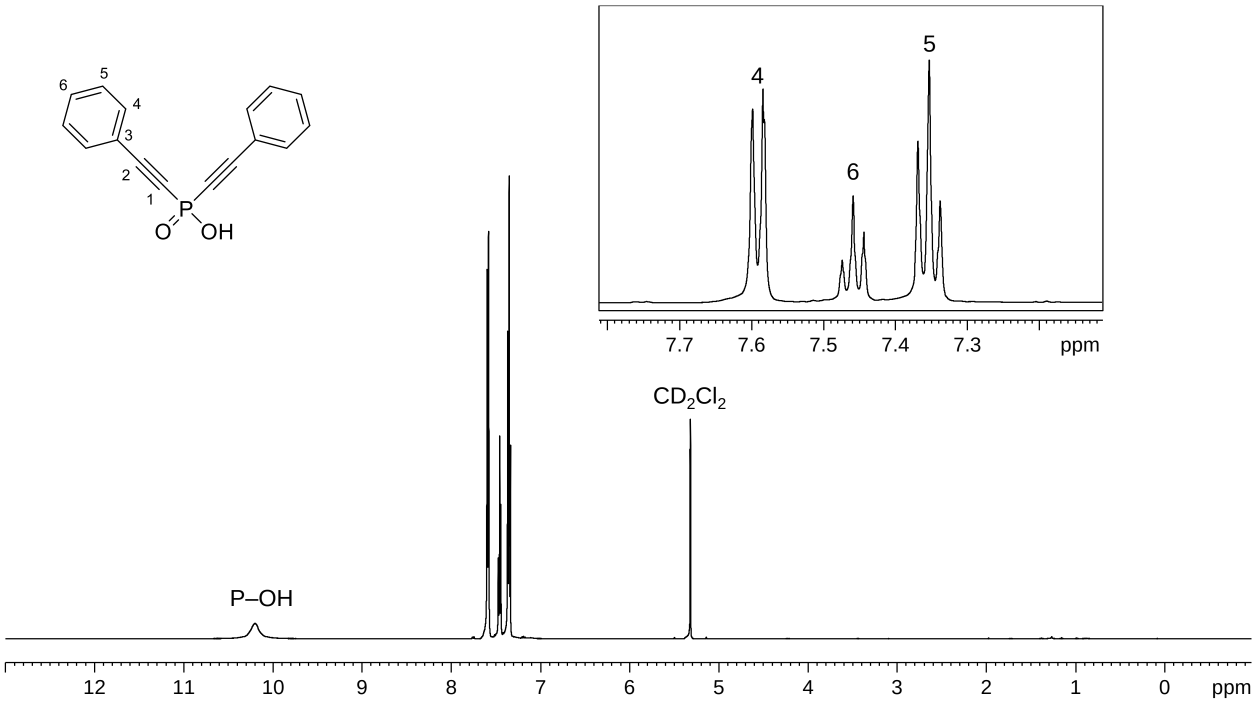

Fig. S 70. ^{1}H NMR spectrum (CD_2Cl_2, 300 K) of bis(phenylethynyl)phosphinic acid (**9b**) synthesized from **1b**[OTf].

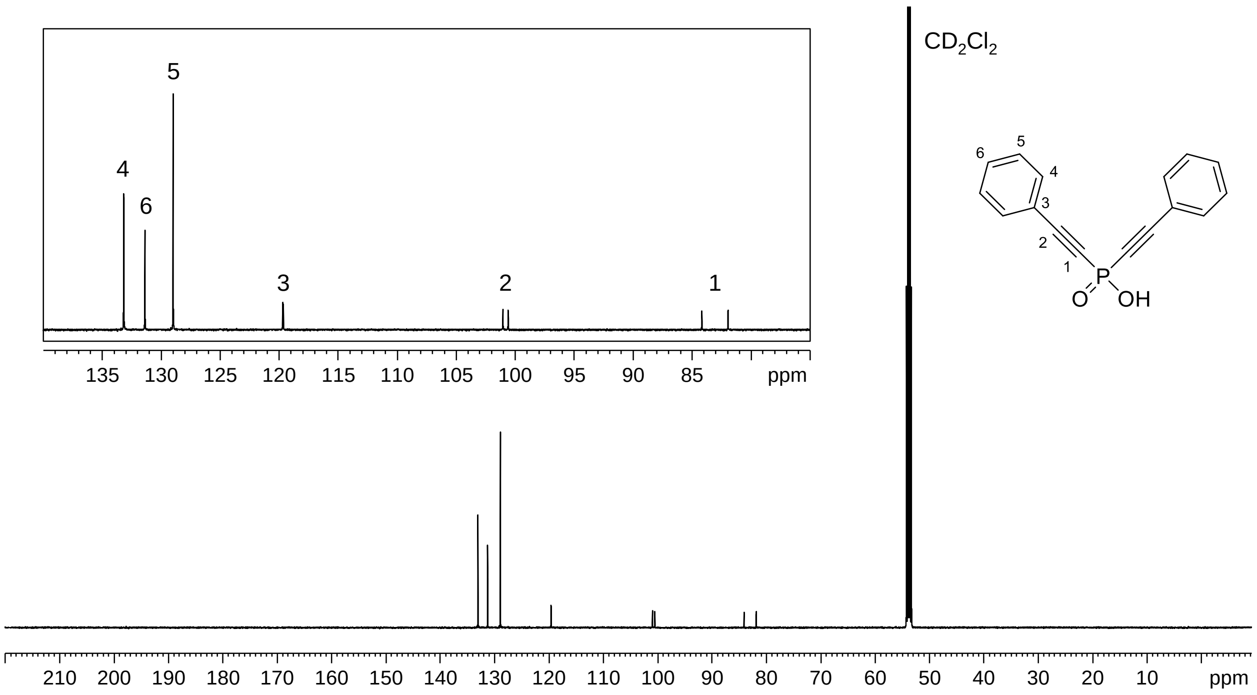

Fig. S 71. $^{13}C\{^{1}H\}$ NMR spectrum (CD_2Cl_2, 300 K) of bis(phenylethynyl)phosphinic acid (**9b**) synthesized from **1b**[OTf].

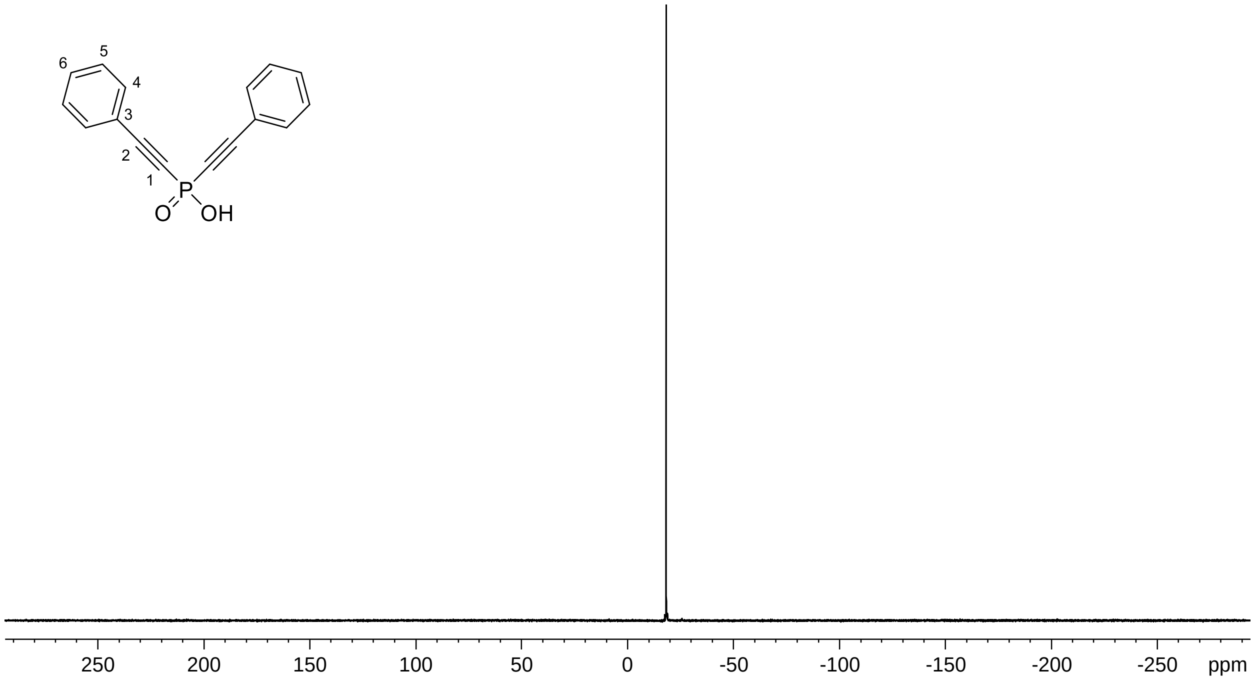

Fig. S 72. ^{31}P NMR spectrum (CD_2Cl_2, 300 K) of bis(phenylethynyl)phosphinic acid (**9b**) synthesized from **1b**[OTf].

2.4.4. Preparation of Bis(*p*-tolylethynyl)phosphinic acid (**9c**)

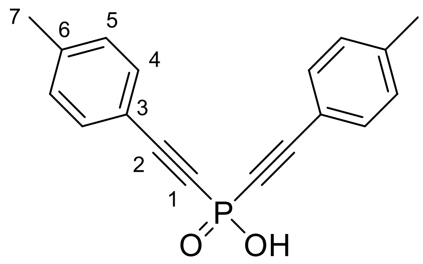

Bis(*p*-tolylethynyl)phosphinic acid (**9c**) was obtained as a colorless powder from synthesis according to the general procedure (case B) with $(DMAP)_2PO_2[OTf]$ (**1b**[OTf], 456 mg, 1 mmol, 1 eq.), *p*-tolylacetylene (380 µL, 3 mmol, 3 eq.) and ethylmagnesium bomide (3 M in Et_2O, 0.83 mL, 2.5 mmol, 2.5 eq.).

Single crystals of **9c** suitable for single crystal structure determination were obtained by slowly cooling a saturated benzene solution to 8 °C (Fig. S 73). Due to its hygroscopy, samples of **9c** for elemental analysis were dried *in vacuo* over P_2O_5 prior to measurement.

Yield: 242 mg (82 %); **Raman** ($\tilde{\nu}$ in cm^{-1}): 2179 (100), 1605 (37), 1233 (8), 1211 (5), 1181 (9), 856 (6), 80 (15); **IR** (ATR, $\tilde{\nu}$ in cm^{-1}): 3034 (vw), 2918 (vw), 2859 (vw), 2175 (m), 2101 (w), 1922 (vw), 1603 (w), 1506 (w), 1444 (w), 1409 (vw), 1379 (vw), 1251 (w), 1232 (w), 1193 (w), 1179 (w), 1104 (m), 1003 (s), 978 (s), 868 (vs), 815 (vs), 782 (s), 770 (m), 707 (w), 646 (w), 611 (vs), 586 (s), 537 (vs), 469 (vs), 459 (vs), 428 (s), 412 (vs); **m.p.**: 147 – 149 °C (decomp.); **^{1}H NMR** (CD_2Cl_2, δ in ppm): 2.36 (s, 6H, H7), 7.15 (d, 4H, $^{3}J_{HH}$ = 8.1 Hz, H5), 7.47 (d, 4H, $^{3}J_{HH}$ = 8.1 Hz, H4), 13.67 (s(br), 1H, P–OH); **^{13}C{^{1}H} NMR** (CD_2Cl_2, δ in ppm): 21.9 (s, C7), 82.7 (d, $^{1}J_{CP}$ = 276.9 Hz, C1), 101.2 (d, $^{2}J_{CP}$ = 53.5 Hz, C2), 116.6 (d, $^{3}J_{CP}$ = 5.5 Hz, C3), 129.7 (s, C5), 133.1 (d, $^{4}J_{CP}$ = 2.3 Hz, C4), 142.2 (s, C6); **^{31}P NMR** (CD_2Cl_2, δ in ppm): – 17.8 (s); **elemental analysis:** calc. for $C_{18}H_{15}O_2P$: C 73.5, H 5.1, N 0.0, S 0.0; found: C 73.3, H 5.0, N 0.0, S 0.0.

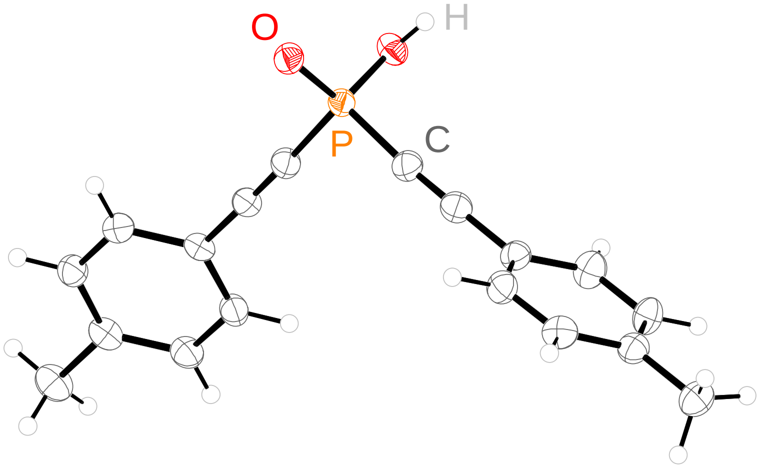

Fig. S 73. Molecular structure of bis(*p*-tolylethynyl)phosphinic acid (**9c**) in **9c** · H_2O · 0.5 C_6H_6; thermal ellipsoids are displayed at 50 % probability level; solvent molecules are omitted for clarity; CCDC 2442196.

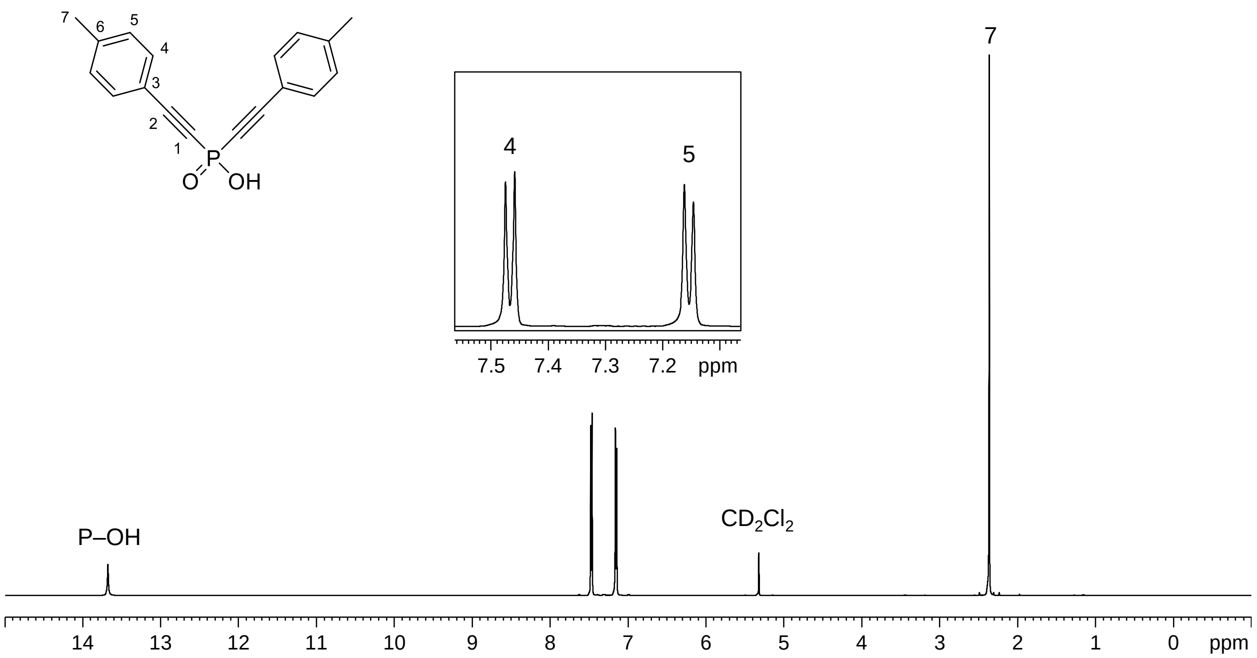

Fig. S 74. ^{1}H NMR spectrum (CD_2Cl_2, 300 K) of bis(*p*-tolylethynyl)phosphinic acid (**9c**) synthesized from **1b**[OTf].

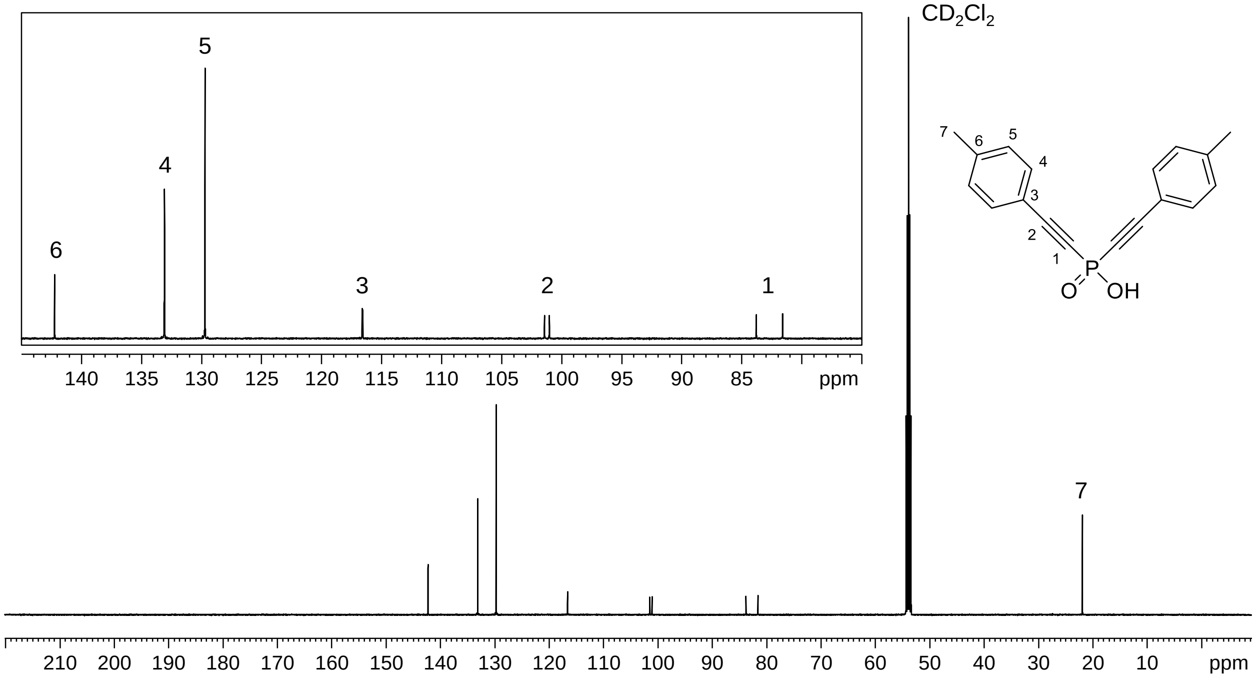

Fig. S 75. $^{13}C\{^{1}H\}$ NMR spectrum (CD_2Cl_2, 300 K) of bis(*p*-tolylethynyl)phosphinic acid (**9c**) synthesized from **1b**[OTf].

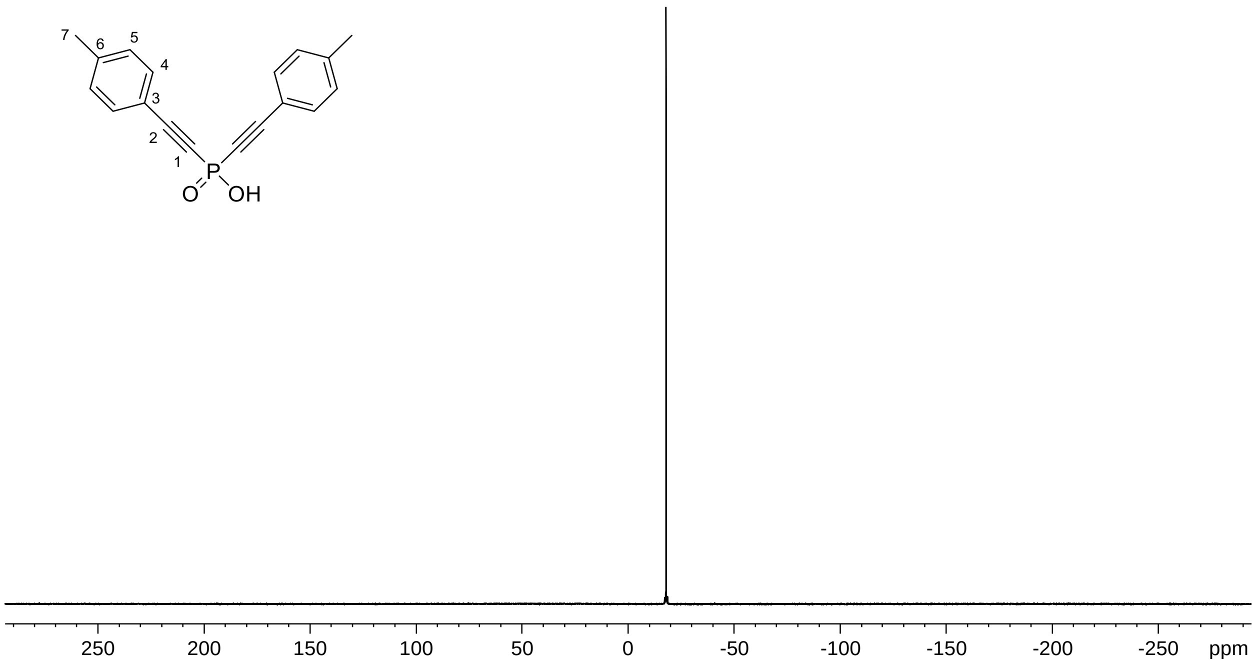

Fig. S 76. ^{31}P NMR spectrum (CD_2Cl_2, 300 K) of bis(*p*-tolylethynyl)phosphinic acid (**9c**) synthesized from **1b**[OTf].

2.4.5. Preparation of Bis((4-*tert*-butylphenyl)ethynyl)phosphinic acid (**9d**)

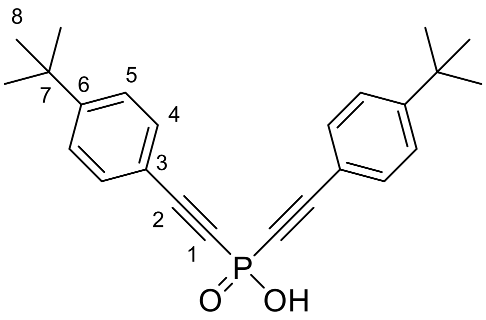

Bis(4-*tert*-butylphenyl)phosphinic acid (**9d**) was obtained as a colorless powder from an adjusted version of the general procedure (case B) with $(DMAP)_2PO_2[OTf]$ (**1b**[OTf], 456 mg, 1 mmol, 1 eq.), *p-tert*-butylphenyl-acetylene (542 µL, 3 mmol, 3 eq.) and ethylmagnesium bomide (3 M in Et_2O, 0.83 mL, 2.5 mmol, 2.5 eq.). Due to the compound's sodium salt being soluble in $CHCl_3$ as determined by ^{31}P NMR spectroscopy, the crude organic extract was instead redissolved in *n*-pentane after solvent removal. All other steps were performed according to the general procedure.

Single crystals of **9d** suitable for single crystal structure determination were obtained by slow vapor diffusion of *n*-pentane into a concentrated Et_2O solution (Fig. S 77).

Due to its hygroscopy, samples of **9d** for elemental analysis were dried *in vacuo* over P_2O_5 prior to measurement.

Yield: 255 mg (67 %); **Raman** ($\tilde{\nu}$ in cm^{-1}): 2185 (100), 1604 (40), 1249 (8), 1190 (11), 1108 (7), 74 (14); **IR** (ATR, $\tilde{\nu}$ in cm^{-1}): 2963 (w), 2905 (vw), 2869 (vw), 2361 (vw), 2180 (vs), 1914 (vw), 1603 (w), 1504 (m), 1462 (w), 1408 (w), 1395 (w), 1365 (w), 1267 (w), 1246 (w), 1199 (m), 1117 (w), 1108 (w), 1015 (w), 980 (vs), 923 (w), 872 (vs), 835 (vs), 721 (s), 699 (w), 653 (vw), 589 (s), 565 (vs), 499 (vs), 479 (vs); **m.p.**: 180 – 182 °C (decomp.); **^{1}H NMR** (CD_2Cl_2, δ in ppm): 1.29 (s, 18H, H8), 7.37 (d, 4H, $^{3}J_{HH}$ = 8.2 Hz, H5), 7.53 (d, 4H, $^{3}J_{HH}$ = 8.2 Hz, H4), 13.55 (s(br), 1H, P–OH); **^{13}C{^{1}H} NMR** (CD_2Cl_2, δ in ppm): 31.2 (s, C8), 35.4 (s, C7), 82.7 (d, $^{1}J_{CP}$ = 276.1 Hz, C1), 101.1 (d, $^{2}J_{CP}$ = 52.7 Hz, C2), 116.7 (d, $^{3}J_{CP}$ = 5.3 Hz, C3), 126.0 (s, C5), 133.0 (d, $^{4}J_{CC}$ = 2.3 Hz, C4), 155.0 (s, C6); **^{31}P NMR** (CD_2Cl_2, δ in ppm): – 18.0 (s); **elemental analysis:** calc. for $C_{24}H_{27}O_2P \cdot 0.4\ H_2O$: C 74.8, H 7.3, N 0.0, S 0.0; found: C 74.4, H 7.0, N 0.0, S 0.0.

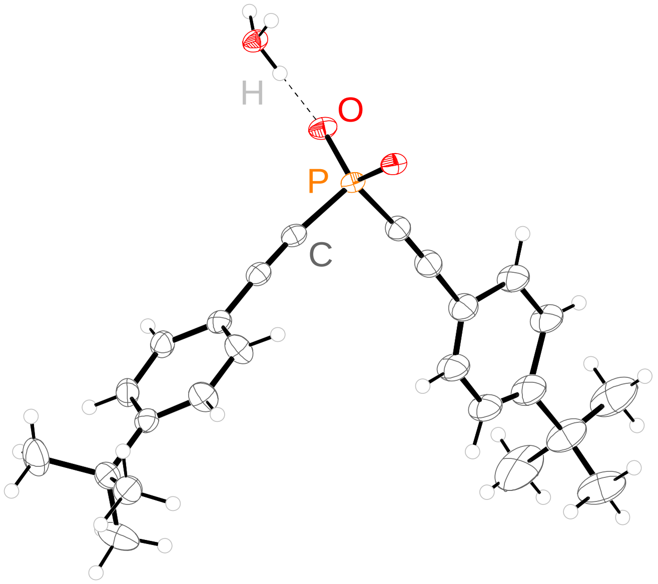

Fig. S 77. Molecular structure of bis((4-*tert*-butylphenyl)ethynyl)phosphinic acid (**9d**) · H_2O in **9d** · H_2O · 0.5 Et_2O; thermal ellipsoids are displayed at 50 % probability level; solvent molecules are omitted for clarity; CCDC 2442193.

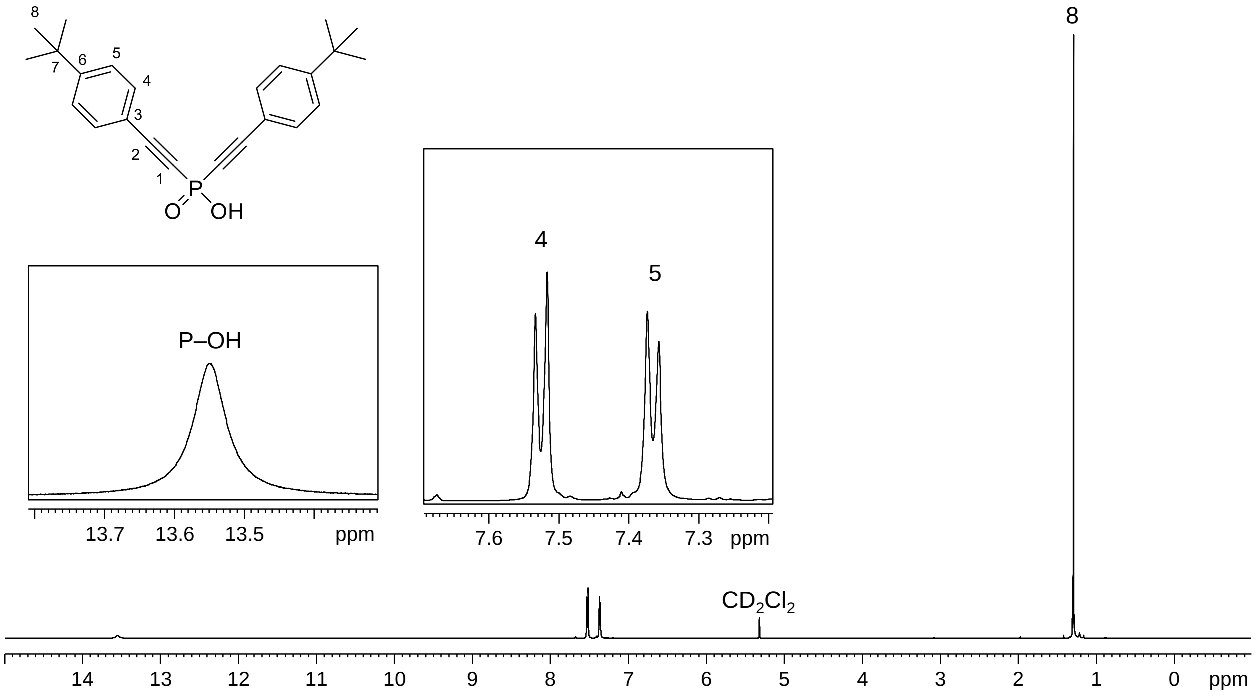

Fig. S 78. ^{1}H NMR spectrum (CD_2Cl_2, 300 K) of bis((4-*tert*-butylphenyl)ethynyl)phosphinic acid (**9d**) synthesized from **1b**[OTf].

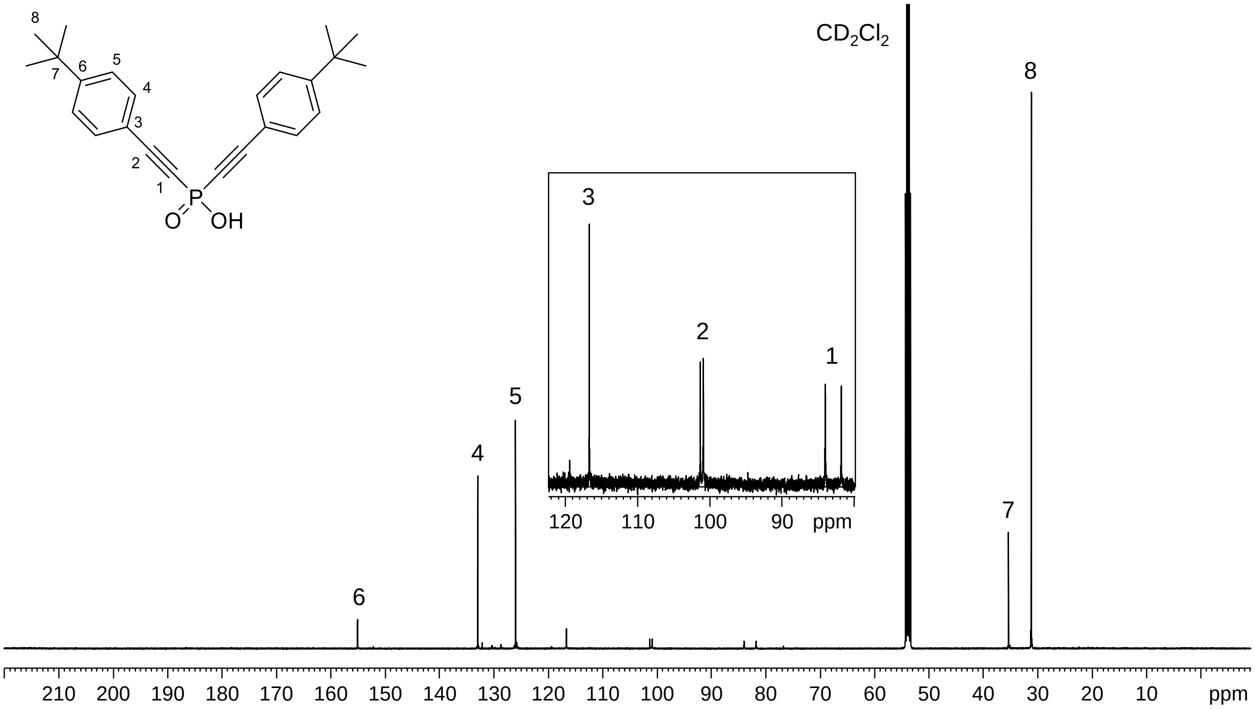

Fig. S 79. $^{13}C\{^{1}H\}$ NMR spectrum (CD_2Cl_2, 300 K) of bis((4-*tert*-butylphenyl)ethynyl)phosphinic acid (**9d**) synthesized from **1b**[OTf].

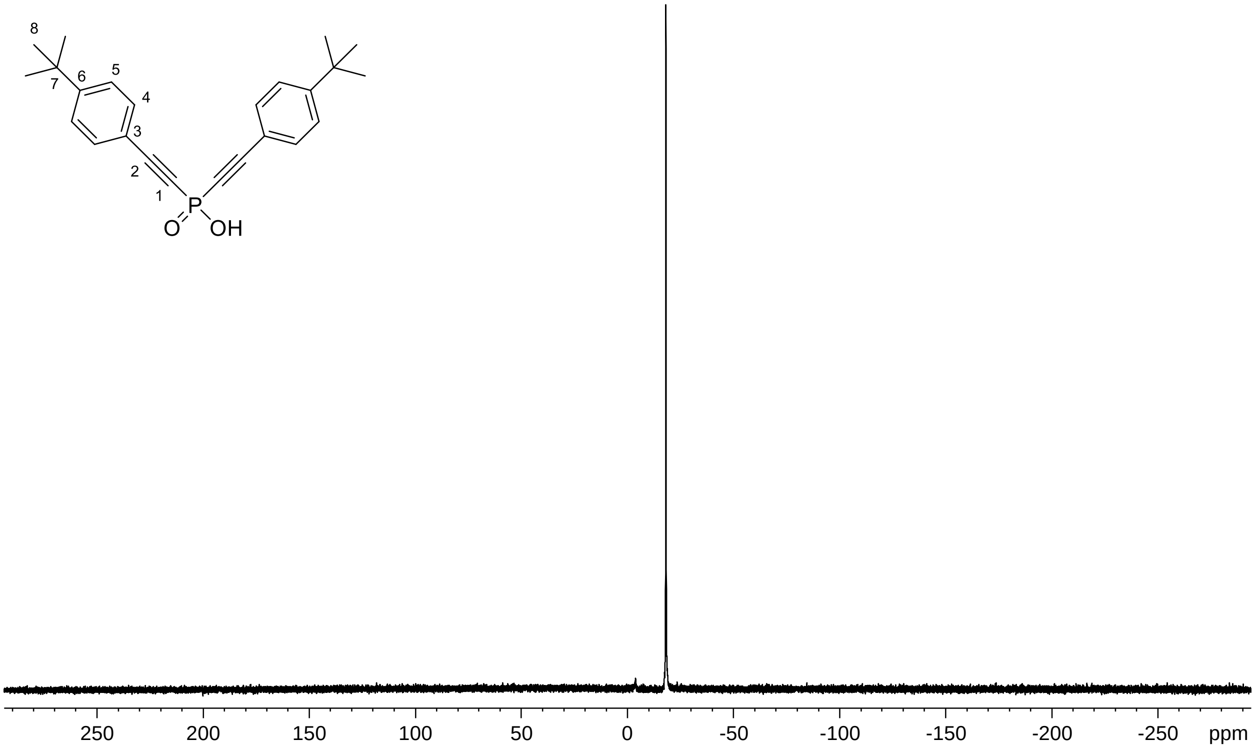

Fig. S 80. ^{31}P NMR spectrum (CD_2Cl_2, 300 K) of bis((4-*tert*-butylphenyl)ethynyl)phosphinic acid (**9d**) synthesized from **1b**[OTf].

2.4.6. Preparation of Bis((4-fluorophenyl)ethynyl)phosphinic acid (**9e**)

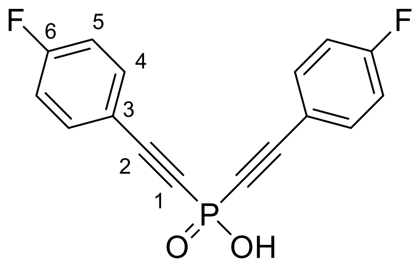

Bis((4-fluorophenyl)ethynyl)phosphinic acid (**9e**) was obtained as a colorless powder from synthesis according to the general procedure (case B) with $(DMAP)_2PO_2[OTf]$ (**1b**[OTf], 456 mg, 1 mmol, 1 eq.), *p*-fluorophenylacetylene (360.4 mg, 3 mmol, 3 eq.) and ethylmagnesium bomide (3 M in Et_2O, 0.83 mL, 2.5 mmol, 2.5 eq.).

Single crystals of **9e** suitable for single crystal structure determination were obtained by slow vapor diffusion of *n*-pentane into a concentrated CH_2Cl_2 solution (Fig. S 81).

Due to its hygroscopy, samples of **9e** for elemental analysis were dried *in vacuo* over P_2O_5 prior to measurement. The obtained values suggest an approximate residual water content of 10 mol% and 25 mol% residual NaCl.

Yield: 186 mg (62 %); **Raman** ($\tilde{\nu}$ in cm^{-1}): 2191 (100), 1598 (42), 1506 (5), 1239 (22), 1160 (5), 864 (10), 789 (5), 96 (26); **IR** (ATR, $\tilde{\nu}$ in cm^{-1}): 3104 (vw), 3071 (vw), 3050 (vw), 2189 (m), 2112 (vw), 2031 (vw), 1905 (vw), 1656 (vw), 1597 (w), 1504 (s), 1484 (w), 1297 (vw), 1254 (vw), 1232 (w), 1215 (m), 1157 (m), 1096 (w), 980 (s), 877 (s), 857 (m), 836 (vs), 799 (vs), 779 (vs), 629 (m), 563 (w), 540 (vs), 470 (s), 451 (vs); **m.p.**: 178 – 180 °C (decomp.); **^{1}H NMR** (CD_2Cl_2, δ in ppm): 7.05 (t, 4H, $^{3}J_{HH}$ = $^{3}J_{HF}$ = 8.4 Hz, H5), 7.58 (dd, 4H, $^{3}J_{HH}$ = 8.2 Hz, $^{4}J_{HH}$ = 5.5 Hz, H4), 13.78 (s(br), 1H, P–OH); **^{13}C{^{1}H} NMR** (CD_2Cl_2, δ in ppm): 82.8 (d, $^{1}J_{CP}$ = 276.3 Hz, C1), 99.8 (d, $^{2}J_{CP}$ = 53.3 Hz, C2), 115.8 (dd, $^{3}J_{CP}$ = 5.4 Hz, $^{4}J_{CF}$ = 3.6 Hz, C3), 116.5 (d, $^{2}J_{CF}$ = 22.6 Hz, C5), 135.5 (dd, $^{3}J_{CF}$ = 9.0 Hz, $^{4}J_{CP}$ = 2.1 Hz, C4), 164.5 (d, $^{1}J_{CF}$ = 253.4 Hz, C6); **^{19}F NMR** (CD_2Cl_2, δ in ppm): – 106.4 (m); **^{31}P NMR** (CD_2Cl_2, δ in ppm): – 19.1 (s); **elemental analysis:** calc. for $C_{16}H_9F_2O_2P \cdot 0.1\ H_2O \cdot 0.25\ NaCl$: C 60.3, H 2.9, N 0.0, S 0.0; found: C 60.3, H 2.8, N 0.0, S 0.1.

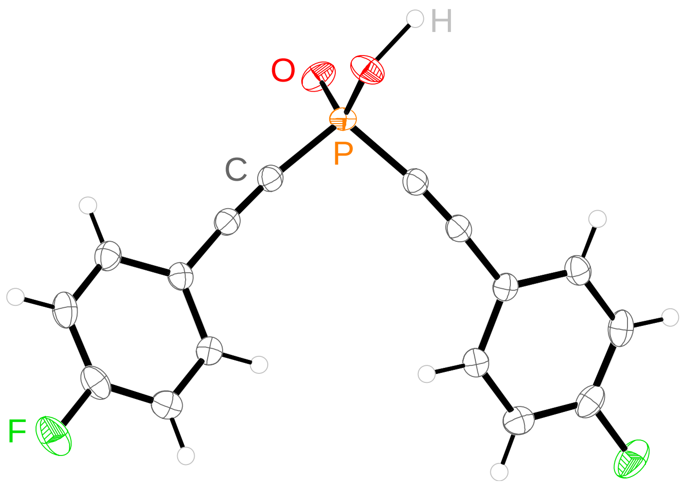

Fig. S 81. Molecular structure of bis((4-fluorophenyl)ethynyl)phosphinic acid (**9e**); thermal ellipsoids are displayed at 50 % probability level; CCDC 2442194.

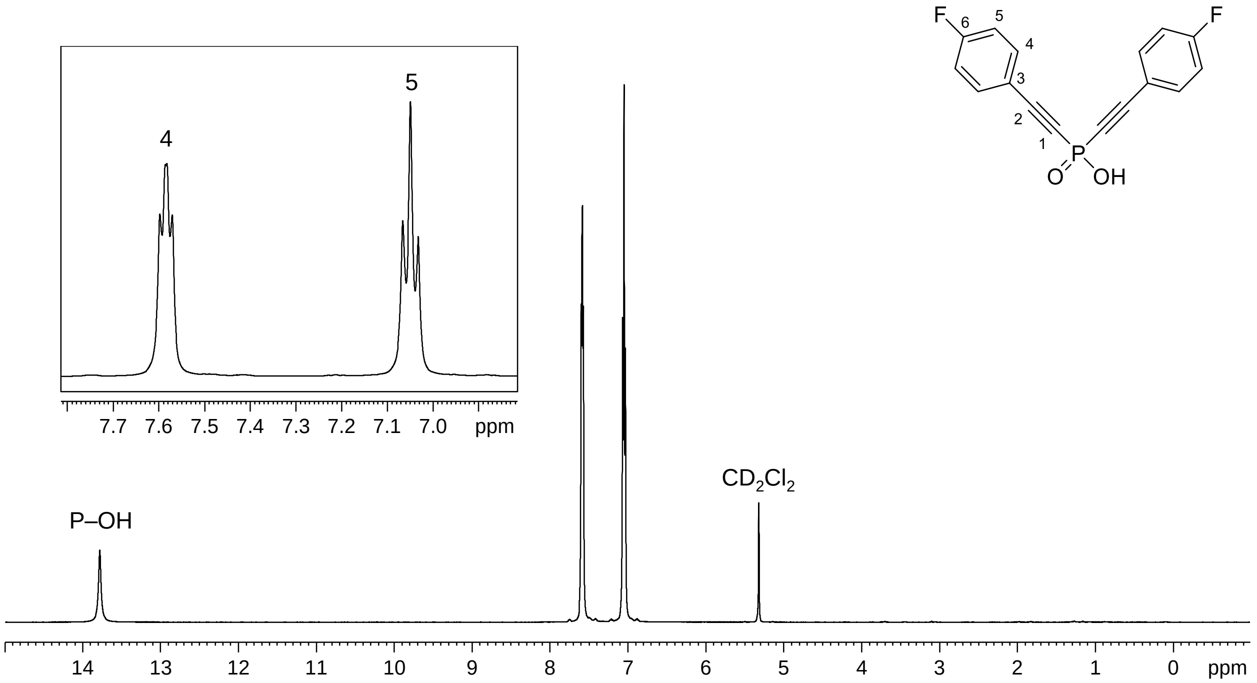

Fig. S 82. ^{1}H NMR spectrum (CD_2Cl_2, 300 K) of bis((4-fluorophenyl)ethynyl)phosphinic acid (**9e**) synthesized from **1b**[OTf].

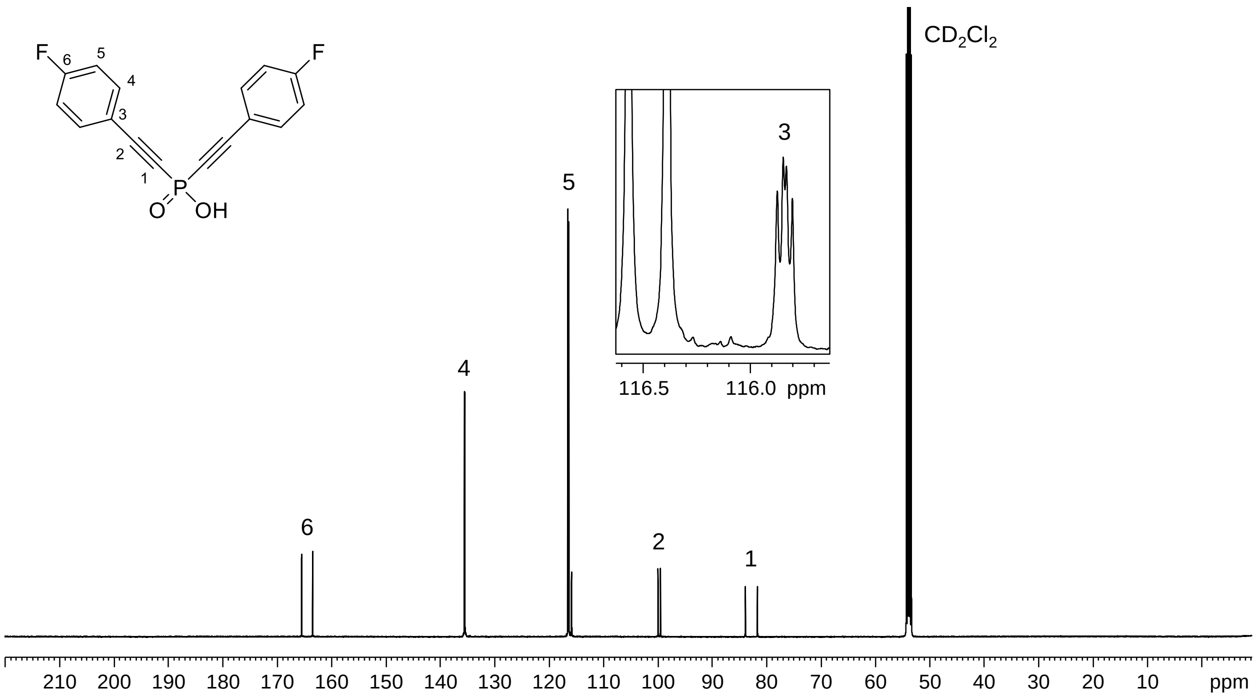

Fig. S 83. $^{13}C\{^{1}H\}$ NMR spectrum (CD_2Cl_2, 300 K) of bis((4-fluorophenyl)ethynyl)phosphinic acid (**9e**) synthesized from **1b**[OTf].

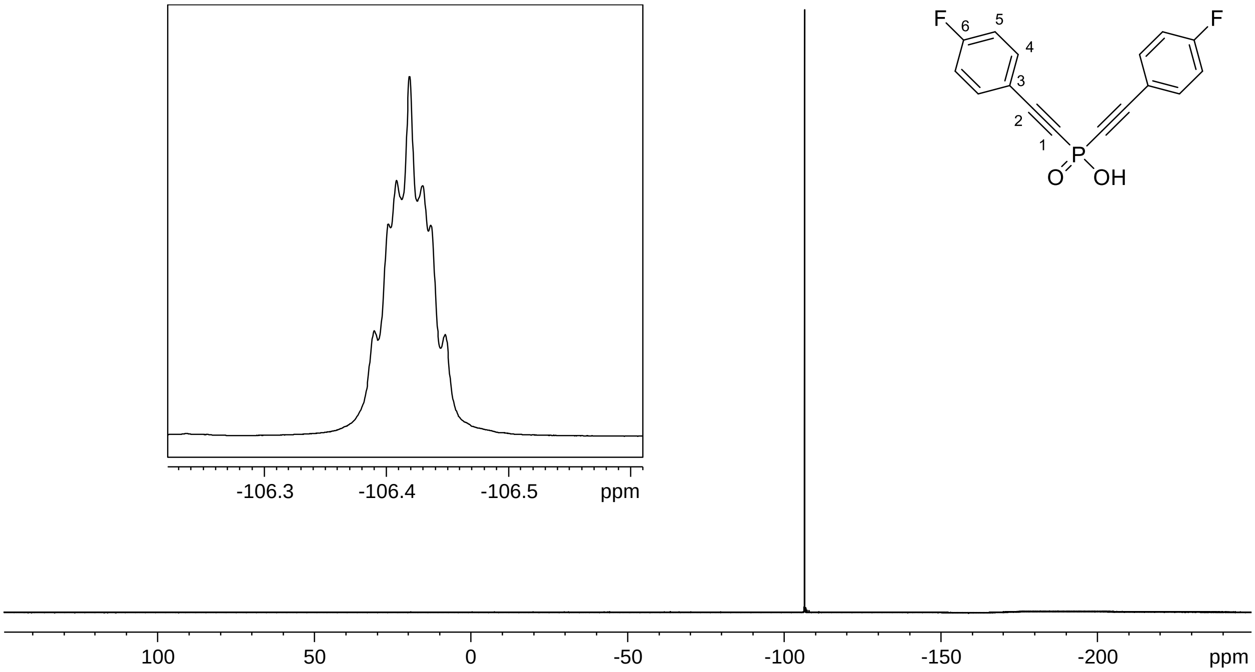

Fig. S 84. ^{19}F NMR spectrum (CD_2Cl_2, 300 K) of bis((4-fluorophenyl)ethynyl)phosphinic acid (**9e**) synthesized from **1b**[OTf].

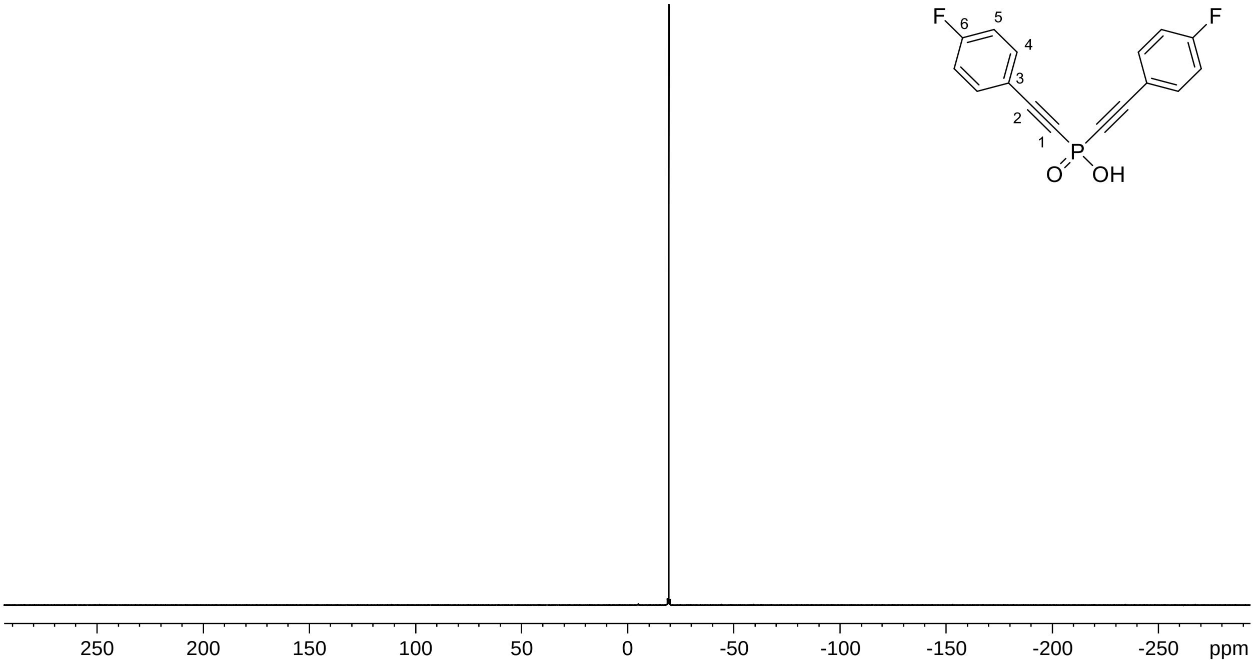

Fig. S 85. ^{31}P NMR spectrum (CD_2Cl_2, 300 K) of bis((4-fluorophenyl)ethynyl)phosphinic acid (**9e**) synthesized from **1b**[OTf].

2.4.7. Preparation of Bis((4-chlorophenyl)ethynyl)phosphinic acid (**9f**)

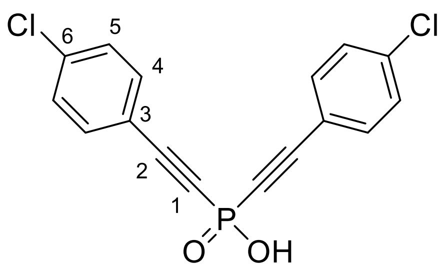

Bis((4-chlorophenyl)ethynyl)phosphinic acid (**9f**) was obtained as an off-white powder from synthesis according to the general procedure (case B) with $(DMAP)_2PO_2[OTf]$ (**1b**[OTf], 456 mg, 1 mmol, 1 eq.), *p*-chlorophenylacetylene (409.7 mg, 3 mmol, 3 eq.) and ethylmagnesium bomide (3 M in Et_2O, 0.83 mL, 2.5 mmol, 2.5 eq.).

Single crystals of **9f** suitable for single crystal structure determination were obtained by slow vapor diffusion of THF into a concentrated MeOH solution (Fig. S 86).

Due to its hygroscopy, samples of **9f** for elemental analysis were dried *in vacuo* over P_2O_5 prior to measurement. The obtained values suggest an approximate residual water content of 40 mol%.

Yield: 291 mg (87 %); **Raman** ($\tilde{\nu}$ in cm^{-1}): 2191 (100), 1589 (48), 1248 (6), 1232 (9), 1178 (6), 1089 (8), 98 (25), 79 (15); **IR** (ATR, $\tilde{\nu}$ in cm^{-1}): 3086 (vw), 2189 (m), 1588 (w), 1479 (m), 1398 (w), 1270 (vw), 1246 (w), 1221 (w), 1168 (w), 1101 (vw), 1086 (m), 980 (s), 873 (vs), 845 (s), 825 (vs), 763 (m), 721 (vs), 689 (vs), 639 (m), 566 (m), 533 (vs), 490 (s), 425 (vs); **m.p.**: 223 – 225 °C (decomp.); **^{1}H NMR** (DMSO-d$_6$, δ in ppm): 7.53 (d, 4H, $^3J_{HH}$ = 8.5 Hz, H5), 7.65 (d, 4H, $^3J_{HH}$ = 8.5 Hz, H4), 14.38 (s(br), 1H, P–OH); **^{13}C{^{1}H} NMR** (DMSO-d$_6$, δ in ppm): 87.2 (d, $^1J_{CP}$ = 244.3 Hz, C1), 95.3 (d, $^2J_{CP}$ = 46.0 Hz, C2), 118.1 (d, $^3J_{CP}$ = 5.0 Hz, C3), 129.2 (s, C5), 134.0 (d, $^4J_{CP}$ = 1.9 Hz, C4), 135.7 (s, C6); **^{31}P NMR** (DMSO-d$_6$, δ in ppm): – 28.7 (s); **elemental analysis:** calc. for $C_{16}H_9Cl_2O_2P \cdot 0.4\ H_2O$: C 56.1, H 2.9, N 0.0, S 0.0; found: C 56.1, H 2.6, N 0.0, S 0.2.

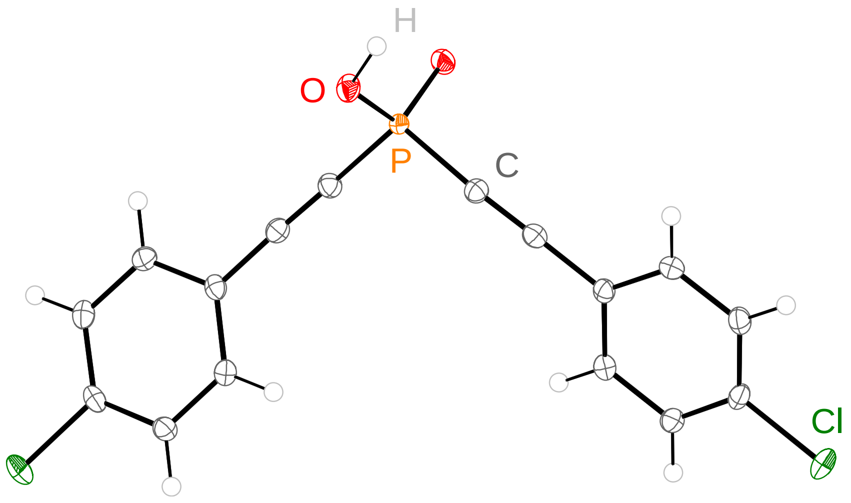

Fig. S 86. Molecular structure of bis((4-chlorophenyl)ethynyl)phosphinic acid (**9f**); thermal ellipsoids are displayed at 50 % probability level; CCDC 2442186.

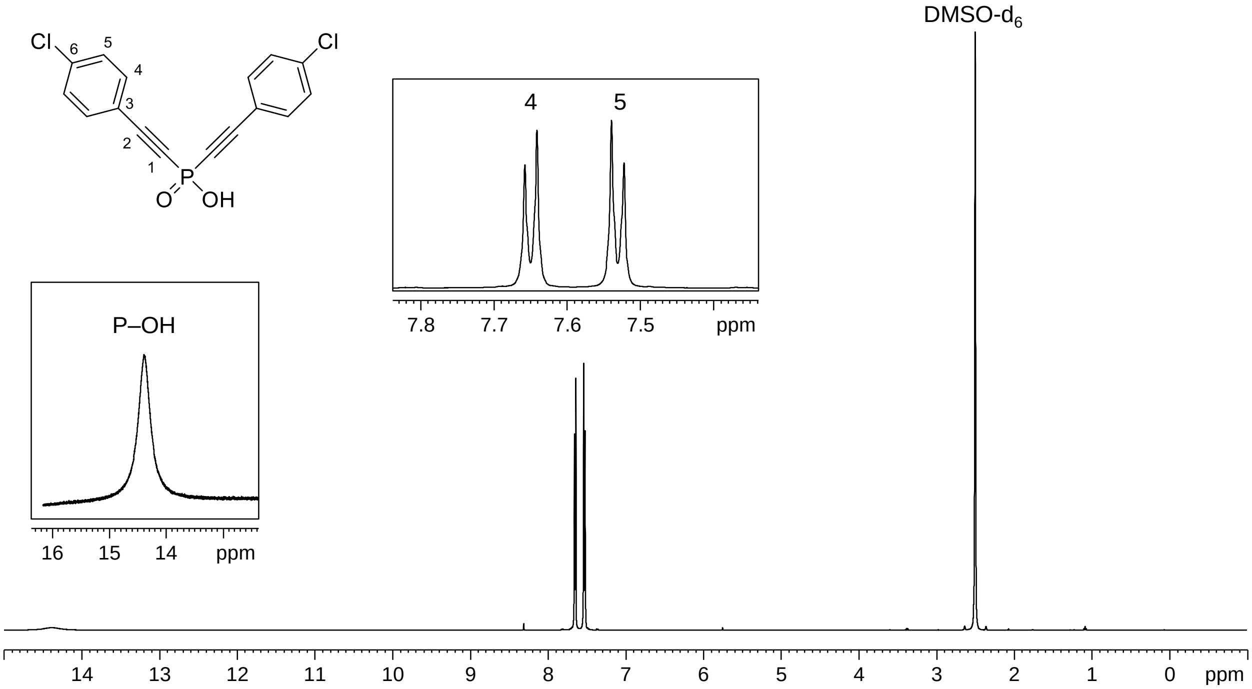

Fig. S 87. ^{1}H NMR spectrum (DMSO-d6, 300 K) of bis((4-chlorophenyl)ethynyl)phosphinic acid (**9f**) synthesized from **1b**[OTf].

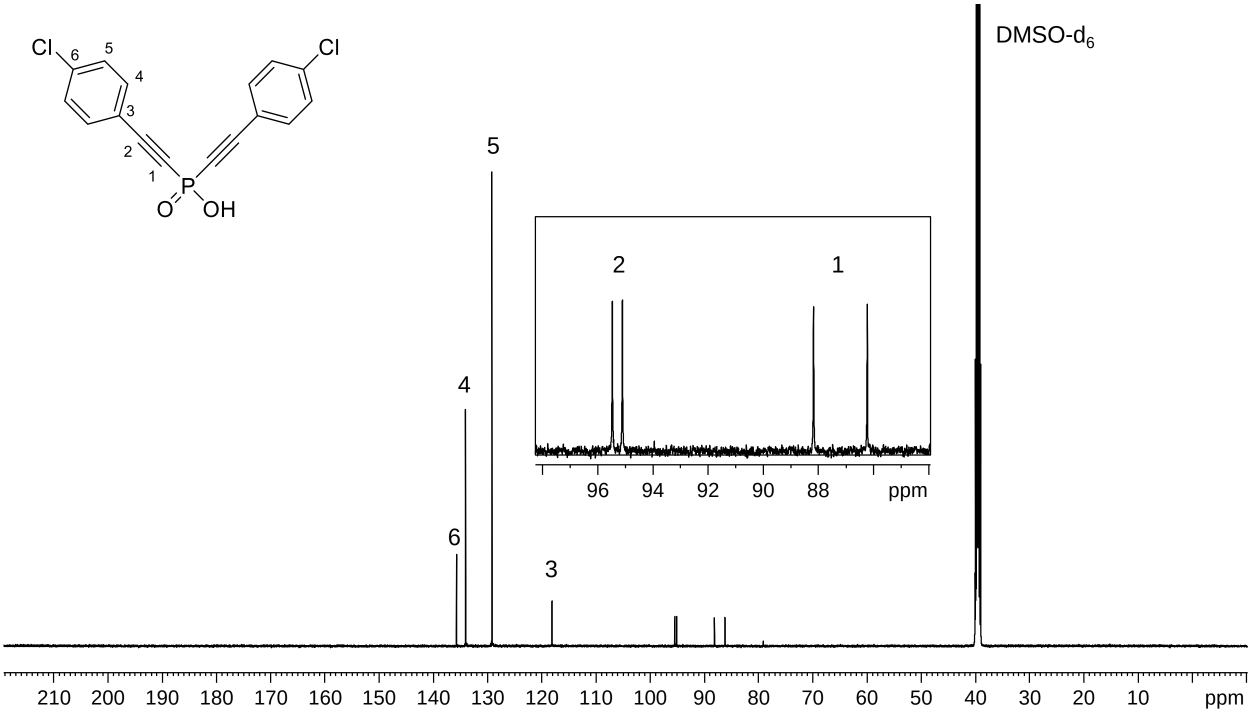

Fig. S 88. $^{13}C\{^{1}H\}$ NMR spectrum (DMSO-d6, 300 K) of bis((4-chlorophenyl)ethynyl)phosphinic acid (**9f**) synthesized from **1b**[OTf].

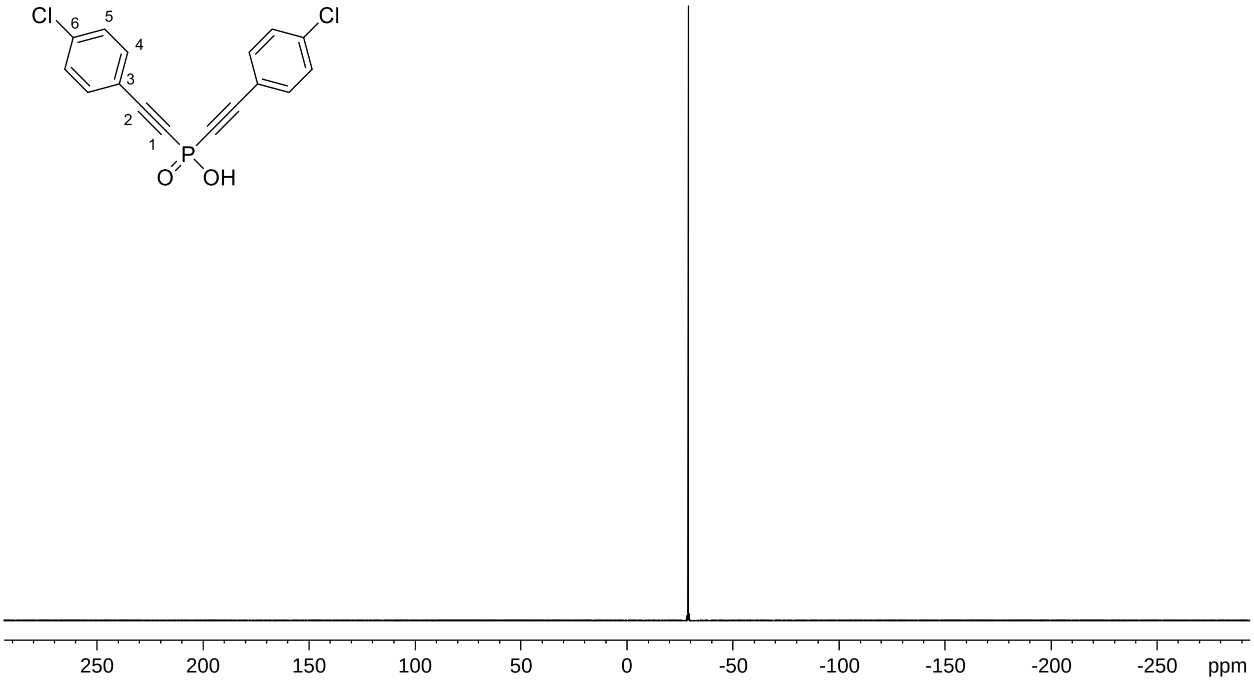

Fig. S 89. ^{31}P NMR spectrum (DMSO-d$_6$, 300 K) of bis((4-chlorophenyl)ethynyl)phosphinic acid (**9f**) synthesized from **1b**[OTf].

2.4.8. Preparation of Bis((4-(trifluoromethyl)phenyl)ethynyl)phosphinic acid (**9g**)

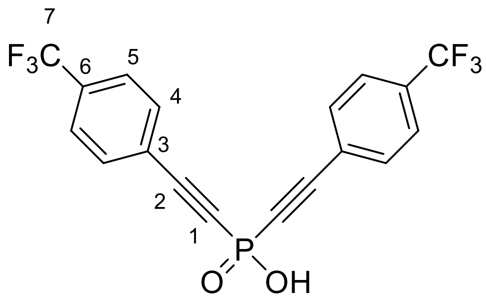

Bis(4-(trifluoromethyl)phenyl)phosphinic acid (**9g**) was obtained as a colorless powder from an adjusted version of the general procedure (case B) with $(DMAP)_2PO_2$[OTf] (**1b**[OTf], 456 mg, 1 mmol, 1 eq.), *p*-(trifluoromethyl)-phenylacetylene (489 µL, 3 mmol, 3 eq.) and ethyl-magnesium bomide (3 M in Et_2O, 0.83 mL, 2.5 mmol, 2.5 eq.). After receiving a crude sodium salt from precipitation with 1 M NaOH with strong brown discoloration, the crude product was first washed with THF to yield a colorless powder before acidification with 1 M HCl. All other steps were performed according to the general procedure.

All attempts to grow crystals of **9g** as the free acid sufficient for single crystal structure determination failed. Single crystals of **9g** as its pyridinium salt were obtained by slow vapor diffusion of Et_2O into a concentrated pyridine solution at – 30 °C (Fig. S 90).

Due to its hygroscopy, samples of **9g** for elemental analysis were dried *in vacuo* over P_2O_5 prior to measurement.

Yield: 297 mg (74 %); **Raman** (ṽ in cm^{-1}): 2192 (100), 1615 (29), 1512 (6), 1241 (8), 1229 (7), 1188 (5), 766 (6), 76 (16); **IR** (ATR, ṽ in cm^{-1}): 2190 (m), 2094 (vw), 1931 (vw), 1614 (vw), 1573 (vw), 1405 (w), 1368 (vw), 1322 (vs), 1240 (w), 1178 (m), 1168 (m), 1121 (vs), 1108 (s), 1067 (vs), 1016 (m), 961 (w), 874 (s), 842 (vs), 792 (w), 775 (m), 737 (w), 690 (s), 665 (m), 642 (w), 597 (m), 551 (m), 516 (m), 456 (m); **m.p.**: 196 – 199 °C (decomp.); **^{1}H NMR** (DMSO-d_6, δ in ppm): 7.80 (d, 4H, $^{3}J_{HH}$ = 8.4 Hz, H5), 7.83 (d, 4H, $^{3}J_{HH}$ = 8.4 Hz, H4), 14.96 (s(br), 1H, P–OH); **^{13}C{^{1}H} NMR** (DMSO-d_6, δ in ppm): 89.2 (d, $^{1}J_{CP}$ = 237.7 Hz, C1), 94.0 (d, $^{2}J_{CP}$ = 44.6 Hz, C2), 123.7 (q, $^{1}J_{CF}$ = 272.5 Hz, C7), 123.8 (dq, $^{3}J_{CP}$ = 4.7 Hz, $^{5}J_{CF}$ = 1.4 Hz, C3), 125.8 (q, $^{3}J_{CF}$ = 3.8 Hz, C5), 130.2 (q, $^{2}J_{CF}$ = 32.2 Hz, C6), 133.0 (d, $^{4}J_{CP}$ = 1.9 Hz, C4); **^{19}F NMR** (DMSO-d_6, δ in ppm): – 61.7 (s); **^{31}P NMR** (DMSO-d_6, δ in ppm): – 30.1 (s); **elemental analysis:** calc. for $C_{18}H_9F_6O_2P$: C 53.8, H 2.3, N 0.0, S 0.0; found: C 53.6, H 2.2, N 0.0, S 0.2.

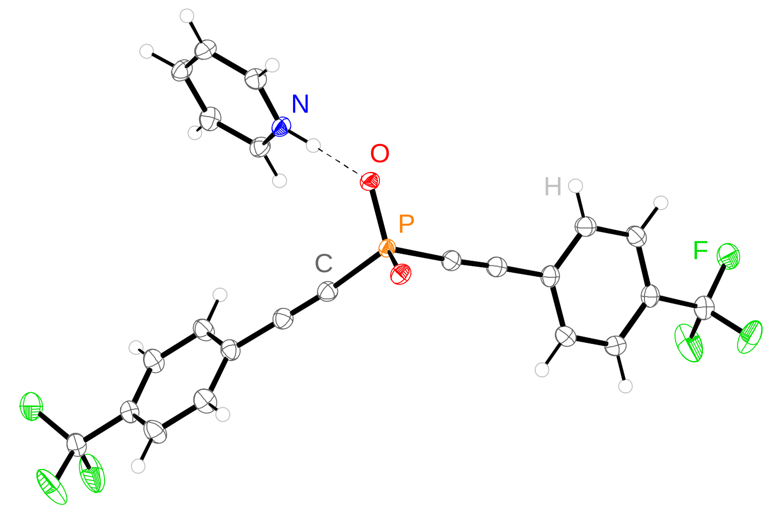

Fig. S 90. Molecular structure of pyridinium bis((4-(trifluoromethyl)phenyl)ethynyl)phosphinate (**9h** · Py); thermal ellipsoids are displayed at 50 % probability level; CCDC 2442187.

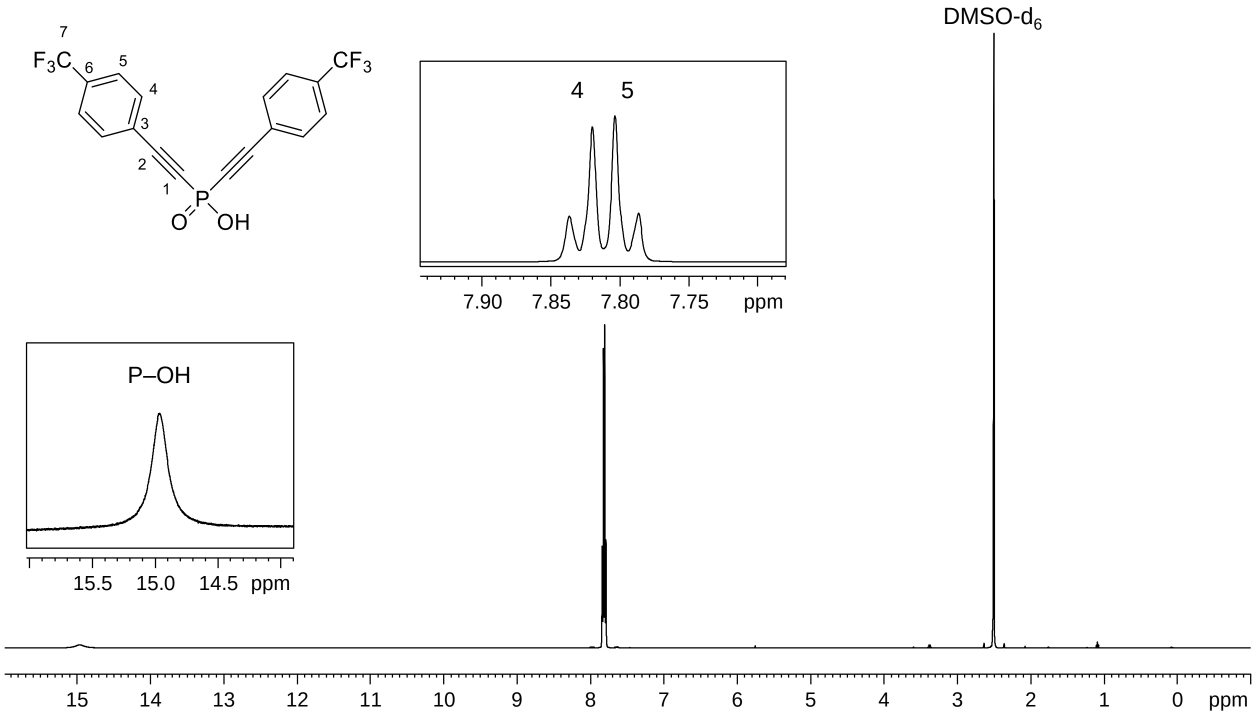

Fig. S 91. ^{1}H NMR spectrum (DMSO-d6, 300 K) of bis((4-(trifluoromethyl)phenyl)ethynyl)phosphinic acid (**9g**) synthesized from **1b**[OTf].

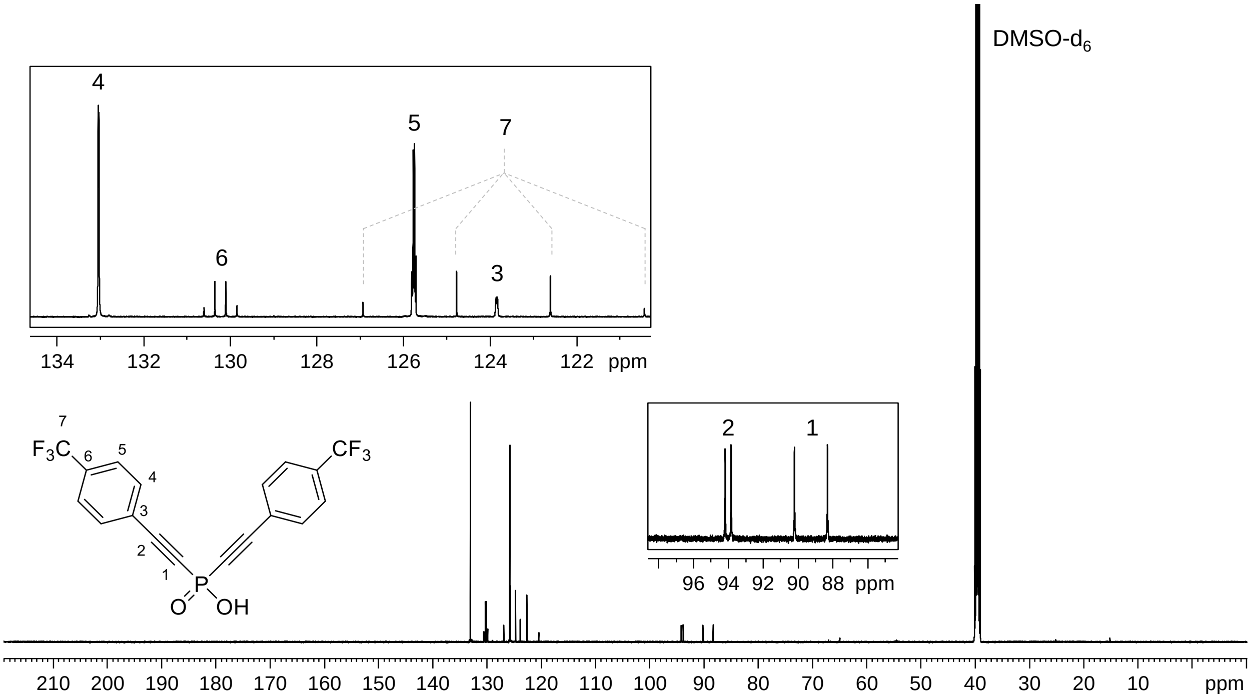

Fig. S 92. $^{13}C\{^{1}H\}$ NMR spectrum (DMSO-d$_6$, 300 K) of bis((4-(trifluoromethyl)phenyl)ethynyl)phosphinic acid (**9g**) synthesized from **1b**[OTf].

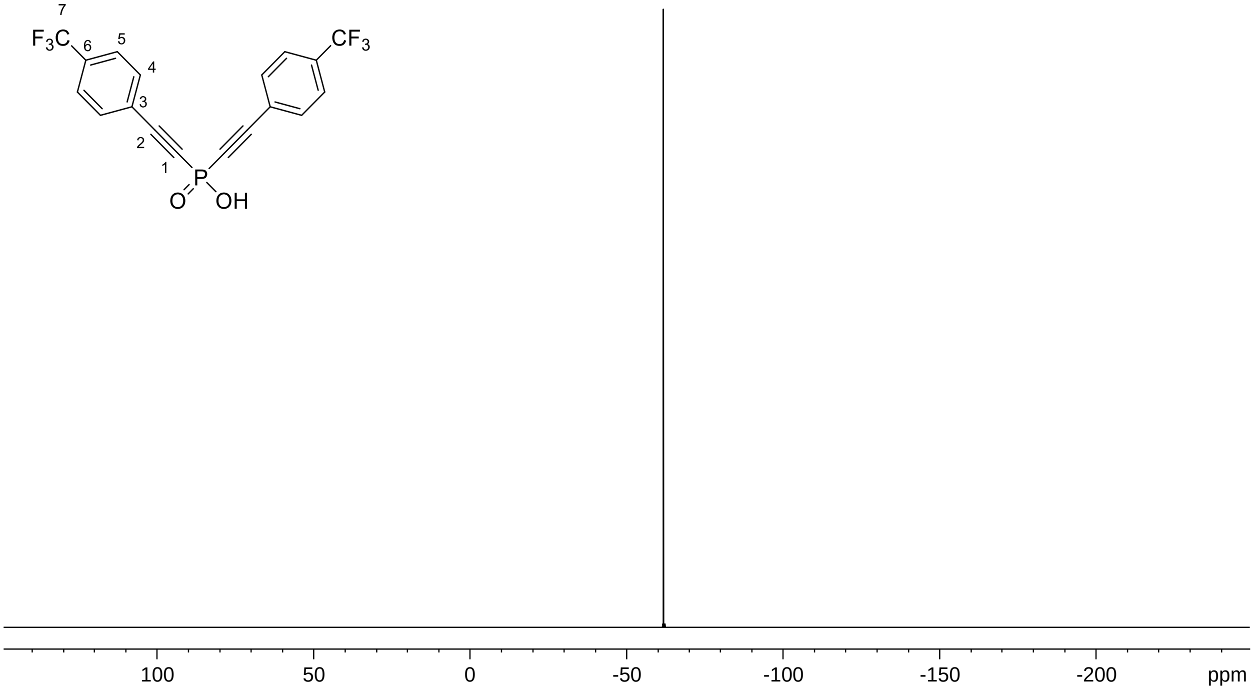

Fig. S 93. ^{19}F NMR spectrum (DMSO-d$_6$, 300 K) of bis((4-(trifluoromethyl)phenyl)ethynyl)phosphinic acid (**9g**) synthesized from **1b**[OTf].

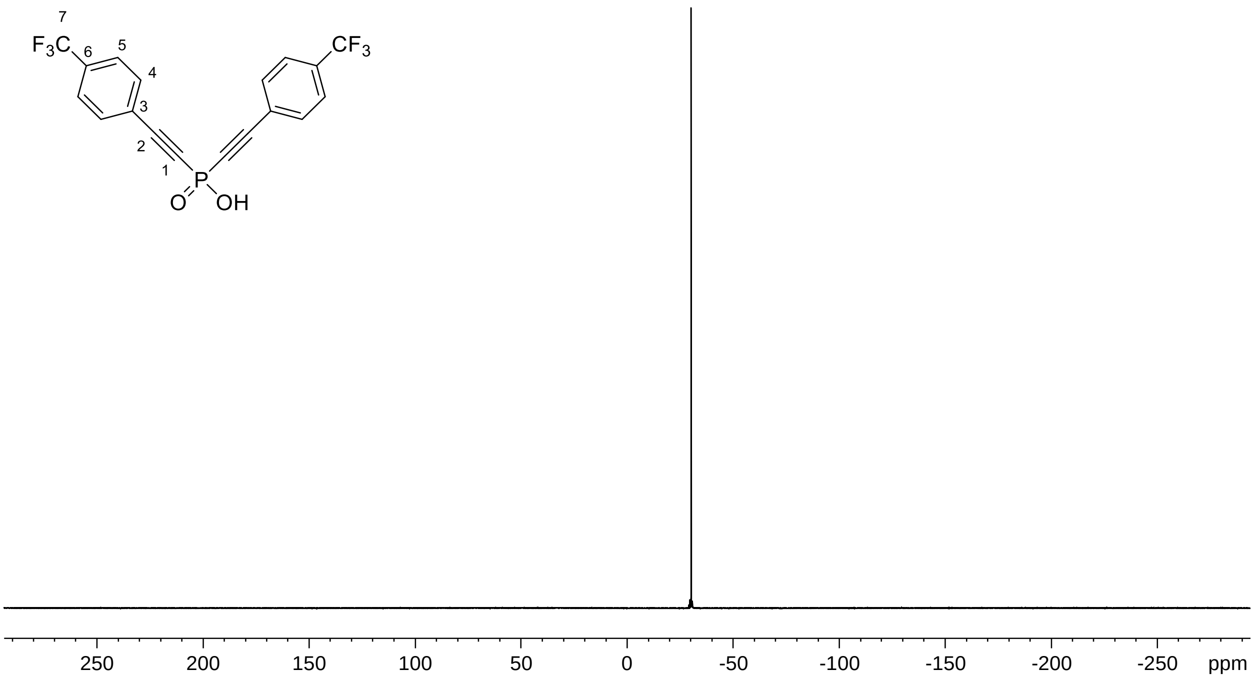

Fig. S 94. ^{31}P NMR spectrum (DMSO-d6, 300 K) of bis((4-(trifluoromethyl)phenyl)ethynyl)phosphinic acid (**9g**) synthesized from **1b**[OTf].

2.4.9. Preparation of Bis(2-naphtylethynyl)phosphinic acid (**9h**)

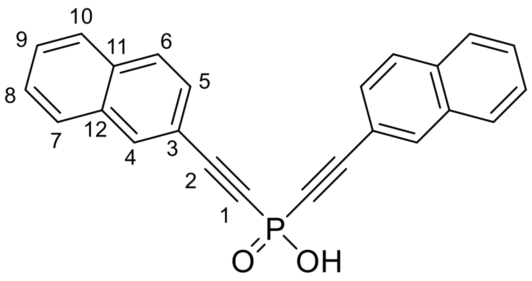

Bis(2-naphtylethynyl)phosphinic acid (**9h**) was obtained as a colorless powder from synthesis according to the general procedure (case B) with $(DMAP)_2PO_2[OTf]$ (**1b**[OTf], 456 mg, 1 mmol, 1 eq.), 2-ethynylnaphtalene (456 mg, 3 mmol, 3 eq.) and ethylmagnesium bomide (3 M in Et_2O, 0.83 mL, 2.5 mmol, 2.5 eq.).

All attempts to crystallize **9h** as the free acid failed. Single crystals of **9h** as its ammonium salt were obtained by suspending the free acid in 1M NH_3 solution, subsequently drying the suspension *in vacuo* and dissolving the obtained sample in MeOH. Slow vapor diffusion of THF into this MeOH solution then gave single crystals suitable for single crystal structure determination (Fig. S 95).

Due to its hygroscopy, samples of **9h** for elemental analysis were dried *in vacuo* over P_2O_5 prior to measurement. The obtained values suggest an approximate residual water content of 20 mol%.

Yield: 346 mg (94 %); **Raman** ($\tilde{\nu}$ in cm^{-1}): 3056 (7), 2186 (100), 1626 (58), 1596 (22), 1466 (26), 1433 (9), 1384 (62), 1179 (14), 1020 (8), 769 (18), 509 (8), 79 (48); **IR** (ATR, $\tilde{\nu}$ in cm^{-1}): 3055 (vw), 2221 (vw), 2180 (m), 1625 (w), 1594 (w), 1571 (w), 1499 (w), 1432 (vw), 1383 (vw), 1366 (vw), 1345 (vw), 1258 (w), 1214 (m), 1178 (w), 1154 (w), 1127 (w), 983 (s), 959 (s), 909 (s), 862 (m), 816 (s), 787 (vs), 747 (s), 634 (vs), 597 (s), 576 (m), 540 (m), 508 (w), 471 (vs), 454 (vs), 422 (s); **m.p.**: 68 – 70 °C (decomp.); **^{1}H NMR** (DMSO-d_6, δ in ppm): 7.58–7.67 (m, 6H, H5/8/9), 7.96–8.02 (m, 6H, H6/7/10), 8.35 (s, 2H, H4), 14.34 (s(br), 1H, P–OH); **^{13}C{^{1}H} NMR** (DMSO-d_6, δ in ppm): 86.3 (d, $^1J_{CP}$ = 246.5 Hz, C1), 97.1 (d, $^2J_{CP}$ = 46.2 Hz, C2), 116.4 (d, $^3J_{CP}$ = 5.0 Hz, C3), 127.2 (s, C9), 127.6 (d, $^4J_{CP}$ = 1.2 Hz, C5), 127.8 (s, C8), 128.1–128.2 (m, C7/10), 128.7 (s, C6), 132.3 (s, C12), 133.3 (s, C11), 133.4 (d, $^4J_{CP}$ = 1.9 Hz, C4); **^{31}P NMR** (DMSO-d_6, δ in ppm): – 27.9 (s); **elemental analysis:** calc. for $C_{24}H_{15}O_2P \cdot 0.2\ H_2O$: C 77.9, H 4.2, N 0.0, S 0.0; found: C 77.7, H 4.0, N 0.0, S 0.0.

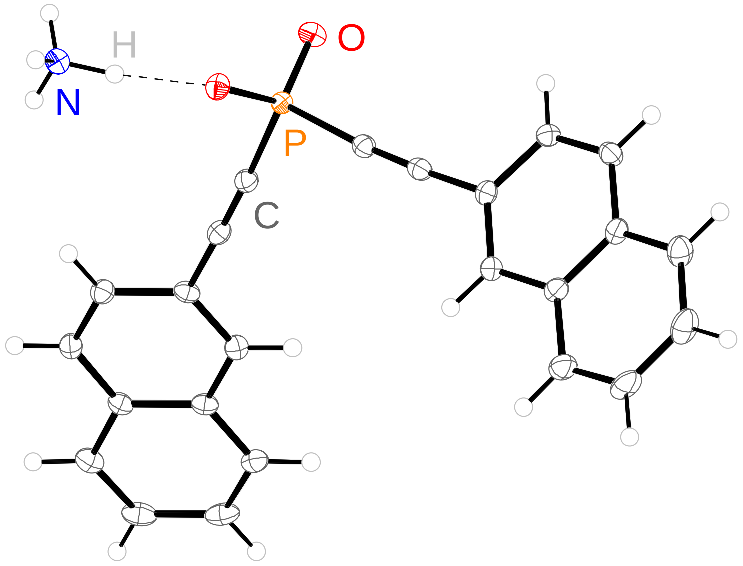

Fig. S 95. Molecular structure of ammonium bis(2-naphtylethynyl)phosphinate (**9h** · NH_3) in **9h** · NH_3 · THF; thermal ellipsoids are displayed at 50 % probability level; solvent molecules are omitted for clarity; CCDC 2442188.

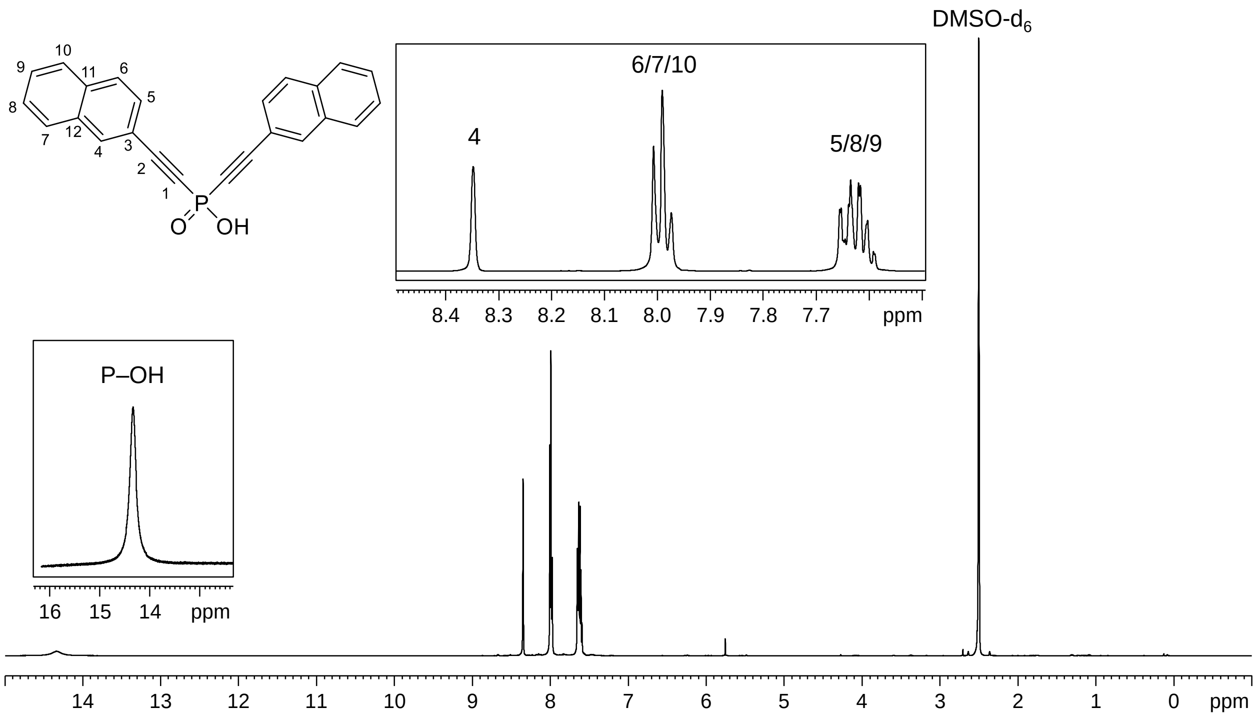

Fig. S 96. ^{1}H NMR spectrum (DMSO-d$_6$, 300 K) of bis(2-naphtylethynyl)phosphinic acid (**9h**) synthesized from **1b**[OTf].

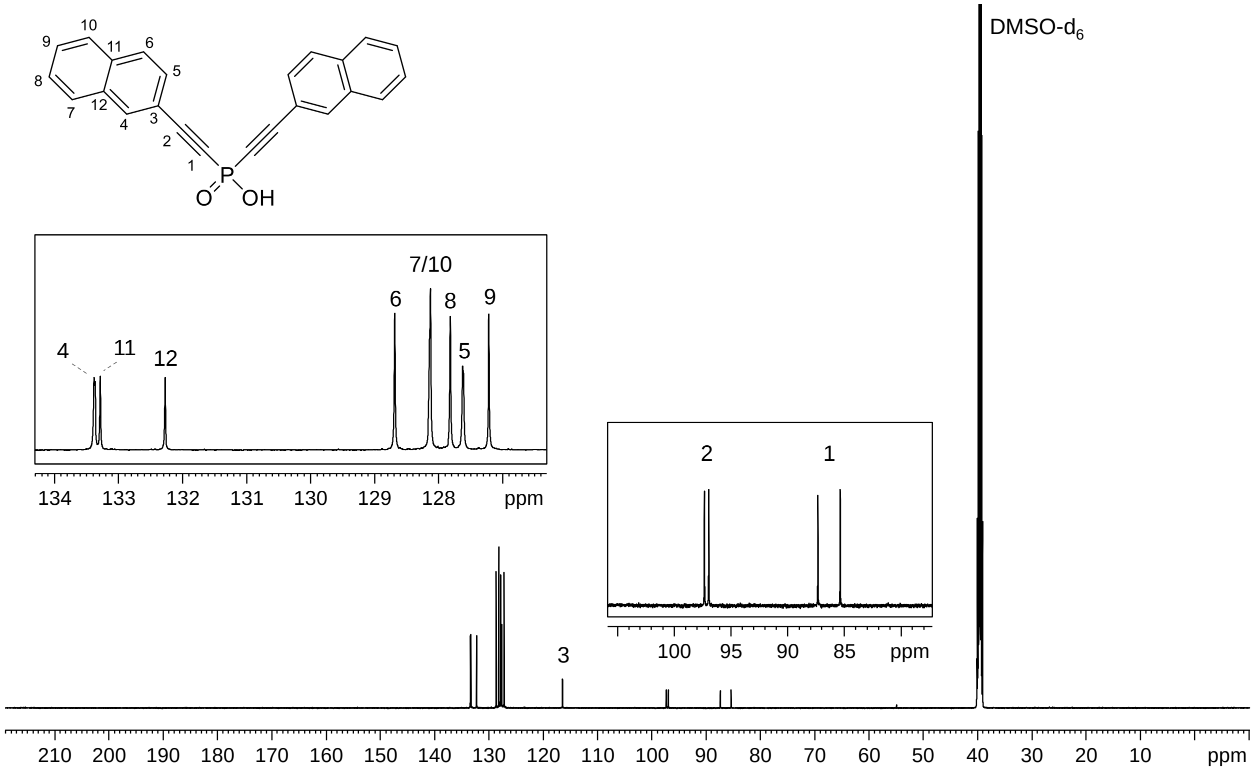

Fig. S 97. $^{13}C\{^{1}H\}$ NMR spectrum (DMSO-d6, 300 K) of bis(2-naphtylethynyl)phosphinic acid (**9h**) synthesized from **1b**[OTf].

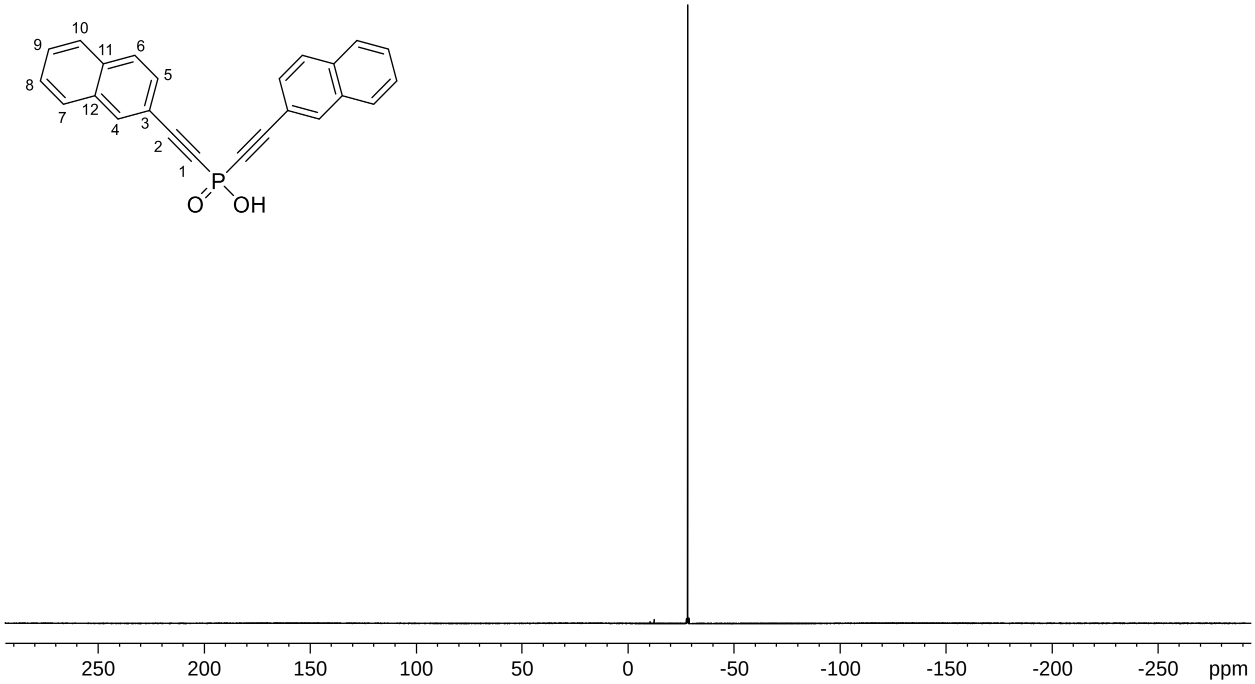

Fig. S 98. ^{31}P NMR spectrum (DMSO-d6, 300 K) of bis(2-naphtylethynyl)phosphinic acid (**9h**) synthesized from **1b**[OTf].

2.4.10. Preparation of Bis(thiophen-3-ylethynyl)phosphinic acid (**9i**)

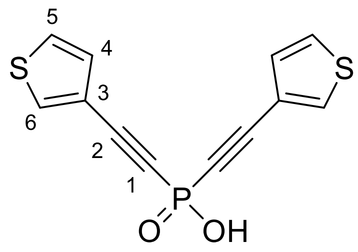

Bis(thiophen-3-ylethynyl)phosphinic acid (**9i**) was obtained as a colorless powder from synthesis according to the general procedure (case A) with $(DMAP)_2PO_2[OTf]$ (**1b**[OTf], 456 mg, 1 mmol, 1 eq.), 3-ethynylthiophene (296 µL, 3 mmol, 3 eq.) and ethyl-magnesium bomide (3 M in Et_2O, 0.83 mL, 2.5 mmol, 2.5 eq.). Single crystals of **9i** suitable for single crystal structure determination were obtained by slow evaporation of *n*-pentane into a concentrated $CHCl_3$ solution (Fig. S 99).

Yield: 215 mg (77 %); **Raman** ($\tilde{\nu}$ in cm^{-1}): 3112 (5), 2187 (99), 2173 (100), 2146 (5), 2138 (6), 2129 (6), 1511 (64), 1402 (25), 1229 (9), 1176 (10), 954 (5), 836 (13), 620 (6), 477 (9), 368 (11), 85 (92); **IR** (ATR, $\tilde{\nu}$ in cm^{-1}): 3108 (w), 2185 (m), 2168 (s), 1582 (w), 1487 (w), 1415 (w), 1358 (w), 1197 (w), 1168 (w), 1047 (m), 979 (m), 953 (vs), 873 (vs), 840 (s), 784 (vs), 763 (vs), 727 (s), 707 (s), 622 (vs), 539 (s), 504 (vs), 468 (s), 451 (s), 426 (vs); **m.p.**: 157 – 159 °C (decomp.); **^{1}H NMR** (CD_2Cl_2, δ in ppm): 7.23 (dd, 2H, $^{3}J_{HH}$ = 5.1 Hz, $^{4}J_{HH}$ = 1.0 Hz, H4), 7.33 (dd, 2H, $^{3}J_{HH}$ = 5.1 Hz, $^{4}J_{HH}$ = 3.0 Hz, H5), 7.79 (d, 2H, $^{4}J_{HH}$ = 2.8 Hz, H6), 9.63 (s(br), 1H, P–OH); **^{13}C{^{1}H} NMR** (CD_2Cl_2, δ in ppm): 82.9 (d, $^{1}J_{CP}$ = 277.3 Hz, C1), 96.2 (d, $^{2}J_{CP}$ = 54.2 Hz, C2), 118.9 (d, $^{3}J_{CP}$ = 5.7 Hz, C3), 126.7 (s, C5), 130.3 (d, $^{4}J_{CP}$ = 1.6 Hz, C4), 134.6 (d, $^{4}J_{CP}$ = 2.6 Hz, C6); **^{31}P NMR** (CD_2Cl_2, δ in ppm): – 18.1 (s); **elemental analysis:** calc. for $C_{12}H_7O_2PS_2$: C 51.8, H 2.5, N 0.0, S 23.0; found: C 51.6, H 2.2, N 0.0, S 23.1.

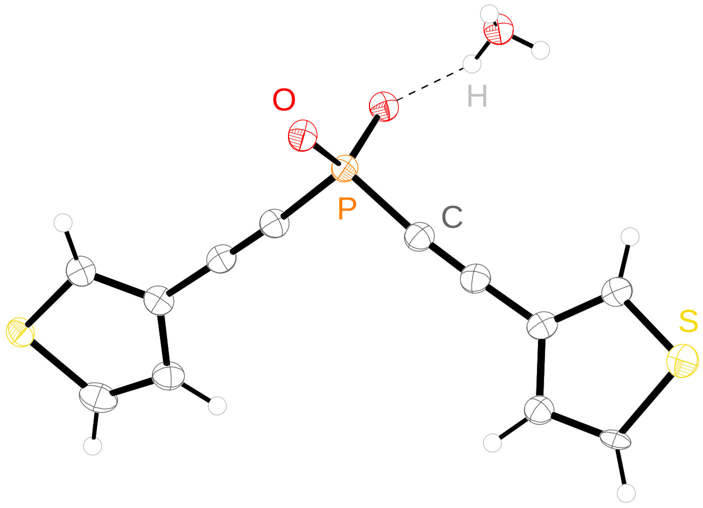

Fig. S 99. Molecular structure of bis(thiophen-3-ylethynyl)phosphinic acid (**9i**) · H_2O; thermal ellipsoids are displayed at 50 % probability level; CCDC 2442197.

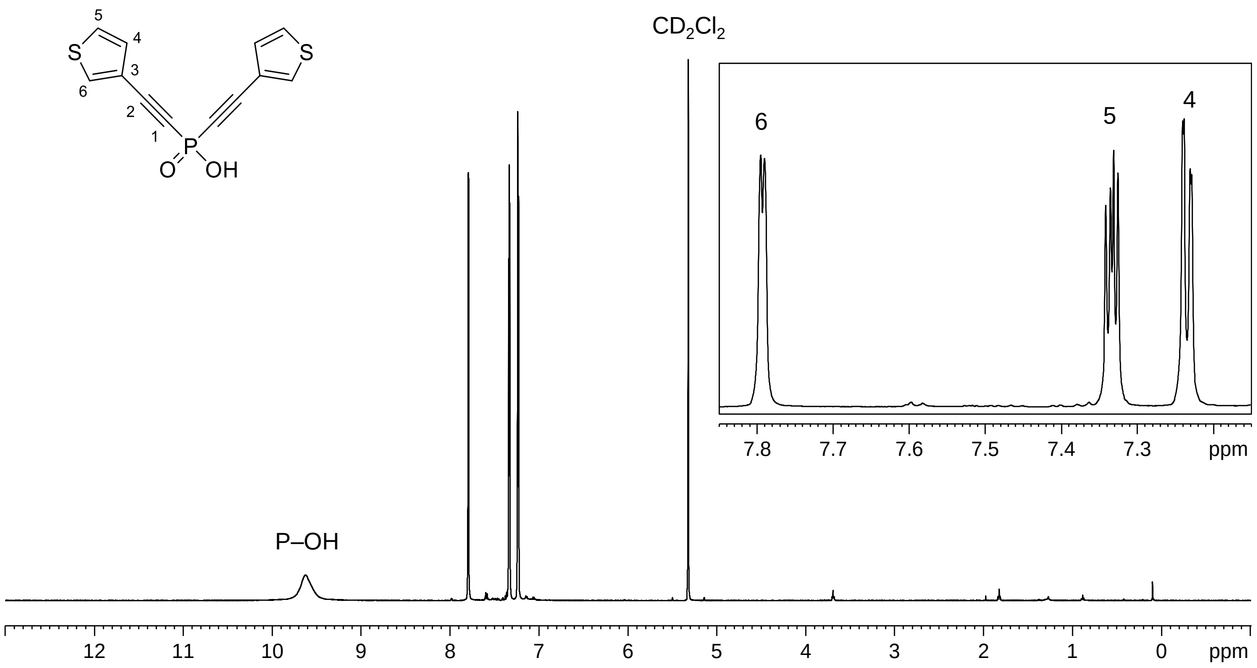

Fig. S 100. ^{1}H NMR spectrum (CD_2Cl_2, 300 K) of bis(thiophen-3-ylethynyl)phosphinic acid (**9i**) synthesized from **1b**[OTf].

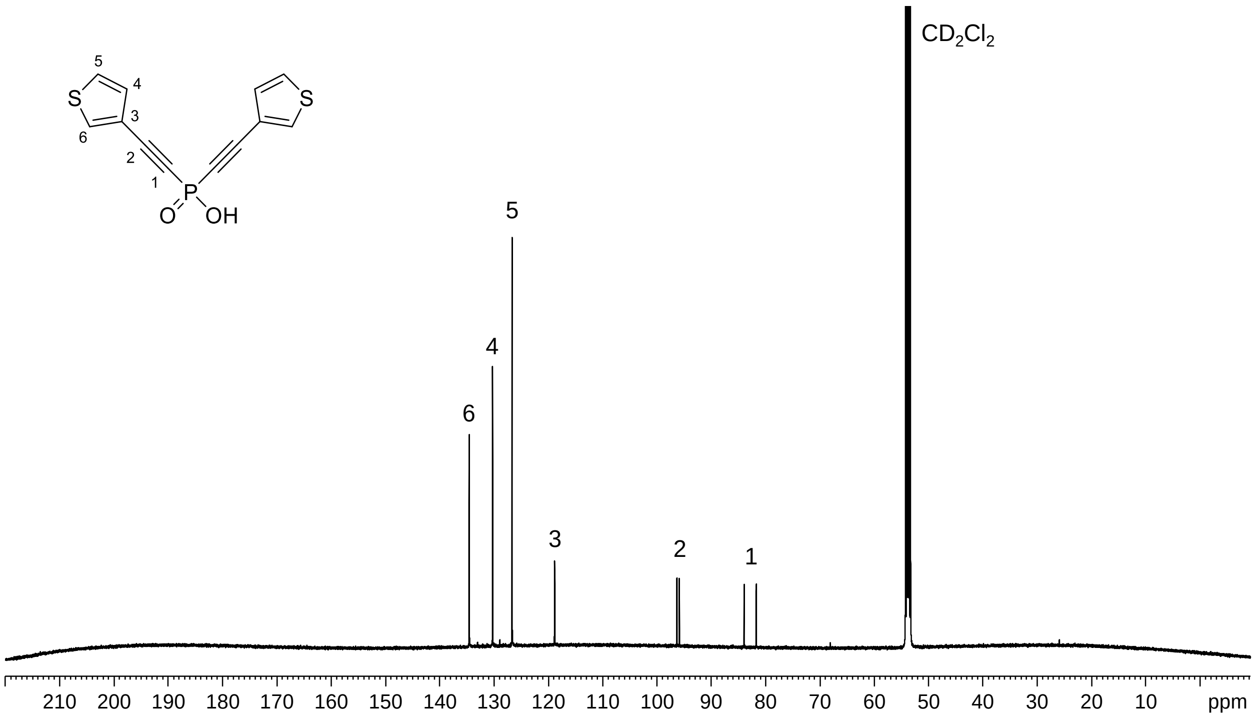

Fig. S 101. $^{13}C\{^{1}H\}$ NMR spectrum (CD_2Cl_2, 300 K) of bis(thiophen-3-ylethynyl)phosphinic acid (**9i**) synthesized from **1b**[OTf].

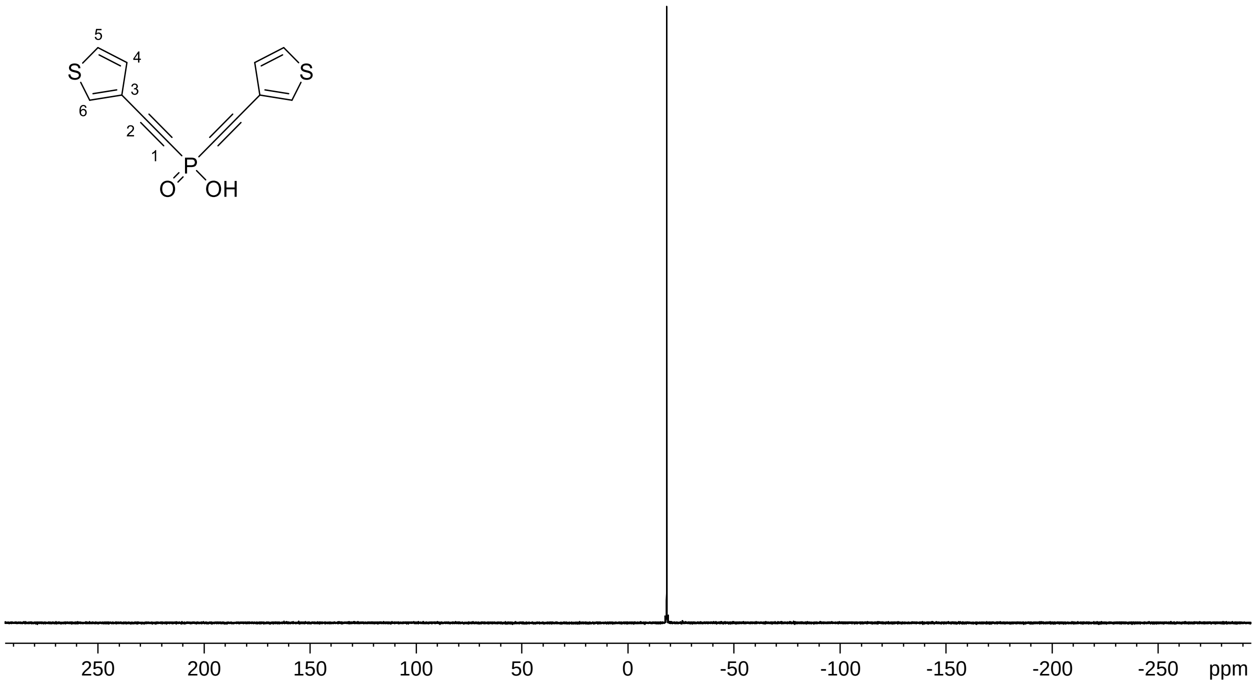

Fig. S 102. ^{31}P NMR spectrum (CD_2Cl_2, 300 K) of bis(thiophen-3-ylethynyl)phosphinic acid (**9i**) synthesized from **1b**[OTf].

2.4.11. Preparation of Ammonium bis(1-hexynyl)phosphinate (**9j** · NH_3)

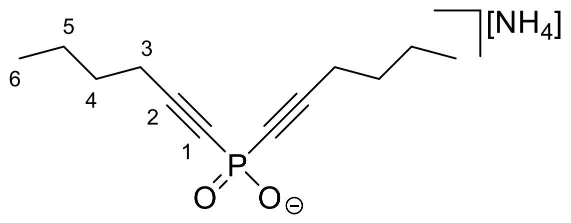

Ammonium bis(1-hexynyl)phosphinate (**9j** · NH_3) was obtained as a colorless powder from synthesis according to the general procedure (case A) with $(DMAP)_2PO_2[OTf]$ (**1b**[OTf], 456 mg, 1 mmol, 1 eq.), 1-hexyne (334 µL, 3 mmol, 3 eq.) and ethylmagnesium bomide (3 M in Et_2O, 0.83 mL, 2.5 mmol, 2.5 eq.).

Single crystals of **9j** · NH_3 suitable for single crystal structure determination were obtained by slow vapor diffusion of *n*-hexane into a concentrated $CHCl_3$ solution (Fig. S 103).

Yield: 173 mg (71 %); **Raman** ($\tilde{\nu}$ in cm^{-1}): (9), 2906 (46), 2870 (21), 2199 (100), 1448 (9), 1421 (7), 1324 (13), 1190 (7), 1108 (5), 1083 (9), 1059 (16), 812 (12), 419 (6), 76 (31); **IR** (ATR, $\tilde{\nu}$ in cm^{-1}): 3152 (vw), 2954 (m), 2930 (m), 2868 (m), 2197 (m), 1844 (vw), 1466 (m), 1445 (m), 1376 (vw), 1363 (vw), 1323 (vw), 1222 (w), 1188 (vs), 1107 (w), 1076 (vs), 1054 (vs), 1026 (m), 986 (vw), 957 (w), 929 (vw), 863 (w), 749 (vw), 629 (vs), 598 (s), 554 (vs), 494 (m), 424 (w); **m.p.**: 179 – 182 °C (decomp.); **^{1}H NMR** ($CDCl_3$, δ in ppm): 0.91 (t, 6H, $^3J_{HH}$ = 7.3 Hz, H6), 1.42 (*pseudo*-sxt, 4H, $^3J_{HH}$ = 7.3 Hz, H5), 1.54 (*pseudo*-quin, 4H, $^3J_{HH}$ = 7.3 Hz, H4), 2.32 (td, 4H, $^3J_{HH}$ = 7.3 Hz, $^4J_{HP}$ = 3.6 Hz, H3), 7.59 (s(br), 4H, NH_4^+); **^{13}C{^{1}H} NMR** ($CDCl_3$, δ in ppm): 13.7 (s, C6), 19.2 (d, $^3J_{CP}$ = 3.7 Hz, C3), 22.3 (s, C5), 30.1 (d, $^4J_{CP}$ = 1.5 Hz, C4), 80.6 (d, $^1J_{CP}$ = 237.7 Hz, C1), 98.2 (d, $^2J_{CP}$ = 44.4 Hz, C2); **^{31}P NMR** ($CDCl_3$, δ in ppm): – 27.5 (quin, $^4J_{PH}$ = 3.6 Hz); **elemental analysis:** calc. for $C_{12}H_{22}NO_2P$: C 59.2, H 9.1, N 5.8, S 0.0; found: C 59.2, H 9.1, N 5.6, S 0.0.

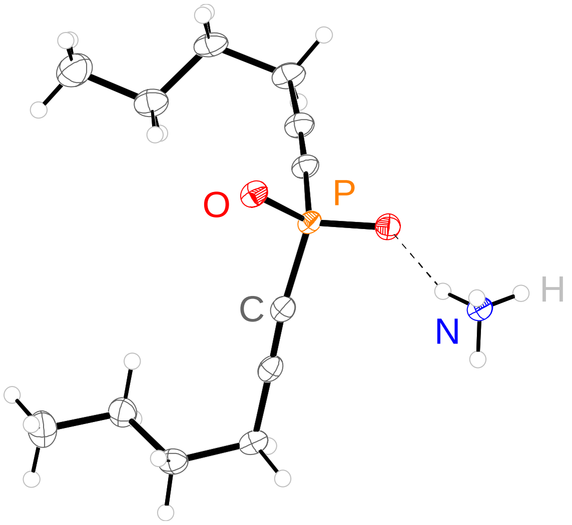

Fig. S 103. Molecular structure of ammonium bis(1-hexynyl)phosphinate (**9j** · NH_3); thermal ellipsoids are displayed at 50 % probability level; CCDC 2442200.

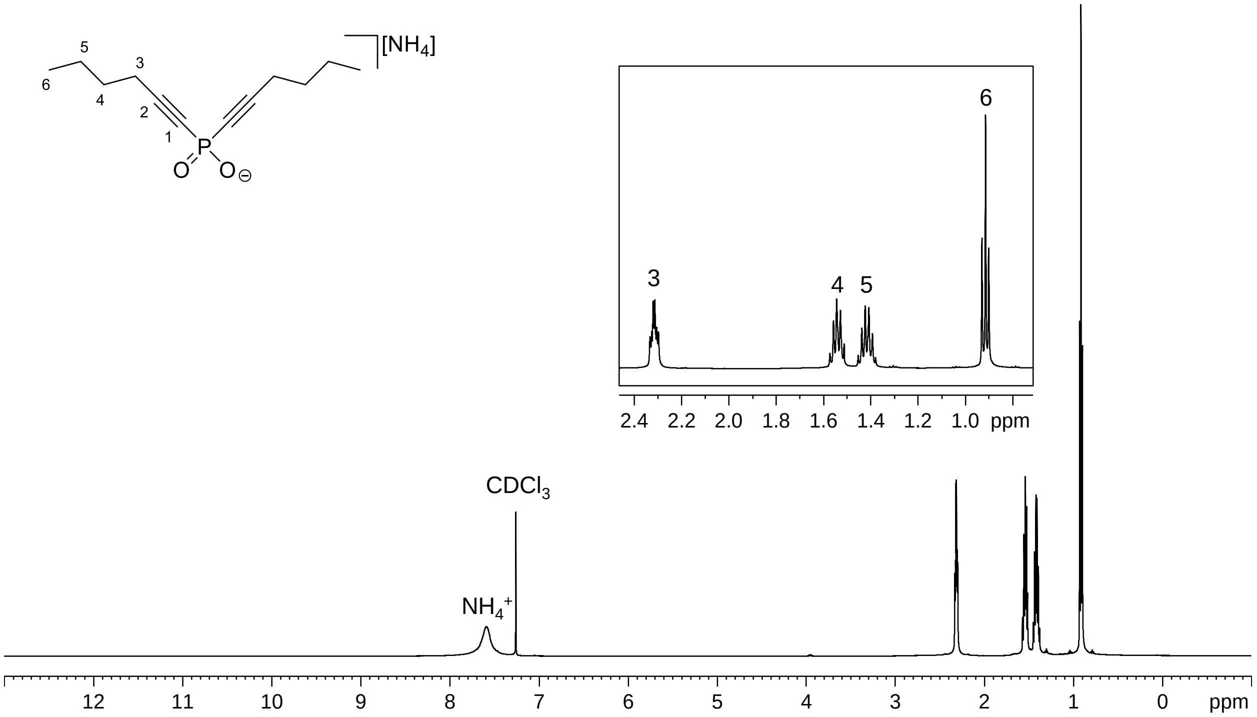

Fig. S 104. ^{1}H NMR spectrum ($CDCl_3$, 300 K) of ammonium bis(1-hexynyl)phosphinate (**9j** · NH_3) synthesized from **1b**[OTf].

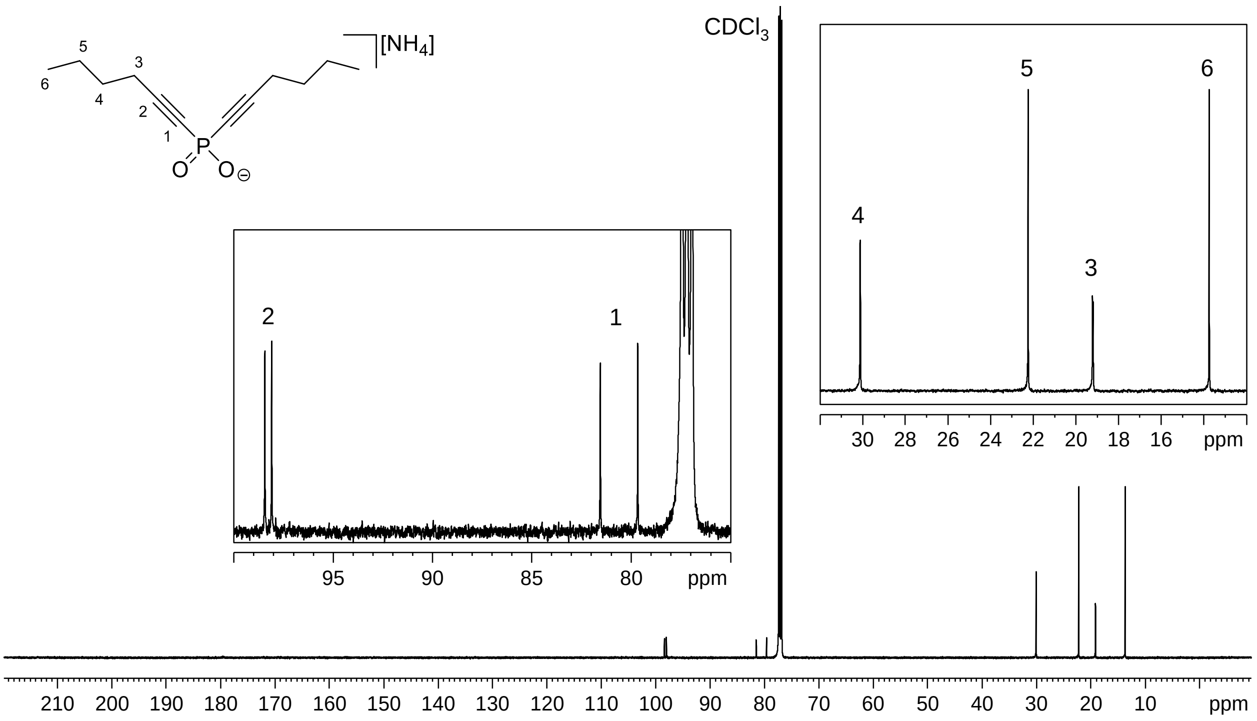

Fig. S 105. $^{13}C\{^{1}H\}$ NMR spectrum ($CDCl_3$, 300 K) of ammonium bis(1-hexynyl)phosphinate (**9j** · NH_3) synthesized from **1b**[OTf].

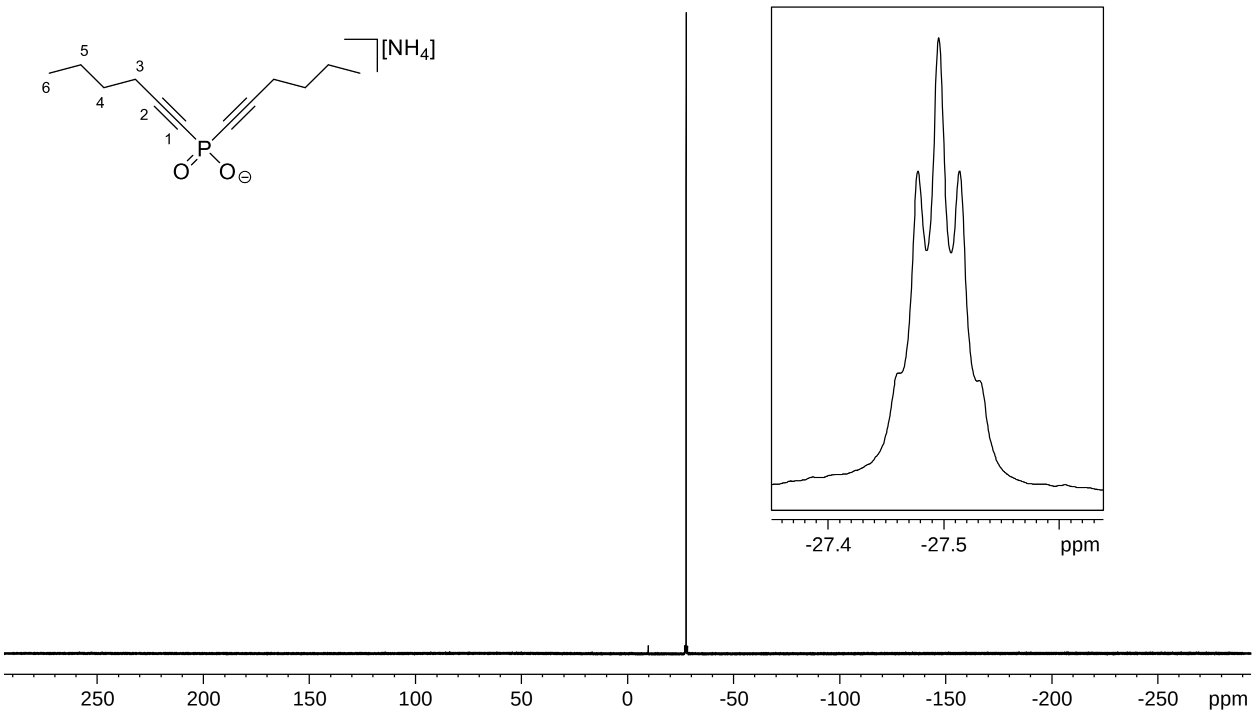

Fig. S 106. ^{31}P NMR spectrum ($CDCl_3$, 300 K) of ammonium bis(1-hexynyl)phosphinate (**9j** · NH_3) synthesized from **1b**[OTf].

2.4.12. Preparation of Ammonium bis(5-chloro-1-pentynyl)phosphinate (**9k** · NH_3)

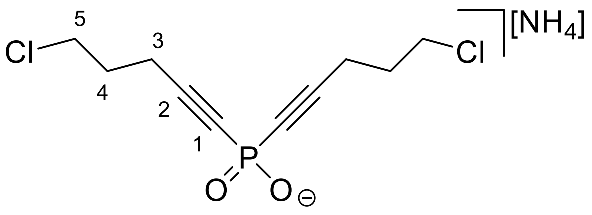

Ammonium bis(5-chloro-1-pentynyl)phosphinate (**9k** · NH_3) was obtained as a colorless powder from synthesis according to the general procedure (case A) with $(DMAP)_2PO_2[OTf]$ (**1b**[OTf], 456 mg, 1 mmol, 1 eq.), 5-chloro-1-pentyne (318 µL, 3 mmol, 3 eq.) and ethylmagnesium bomide (3 M in Et_2O, 0.83 mL, 2.5 mmol, 2.5 eq.).

Single crystals of **9k** · NH_3 suitable for confirmation of the compound's structural connectivity were obtained by slow vapor diffusion of Et_2O into a concentrated pyridine solution. Prolonged X-ray irradiation of the crystals was reproducibly observed to induce sample decay, preventing measurement of a sufficient data set for structure refinement.

Yield: 198 mg (70 %); **Raman** ($\tilde{\nu}$ in cm^{-1}): 2954 (29), 2917 (28), 2199 (100), 1443 (7), 1427 (11), 1327 (7), 1262 (5), 1069 (9), 1039 (10), 856 (6), 662 (15), 548 (6), 423 (8), 282 (5), 185 (6), 77 (27); **IR** (ATR, $\tilde{\nu}$ in cm^{-1}): 2959 (w), 2914 (w), 2857 (w), 2196 (m), 1864 (vw), 1682 (vw), 1467 (m), 1426 (w), 1354 (vw), 1326 (vw), 1288 (w), 1184 (vs), 1153 (w), 1061 (vs), 1048 (s), 1036 (s), 986 (w), 953 (w), 920 (w), 854 (w), 782 (w), 727 (w), 661 (s), 633 (vs), 592 (m), 543 (vs), 497 (m); **m.p.**: 186 – 189 °C (decomp.); **^{1}H NMR** (DMSO-d$_6$, δ in ppm): 1.88 (*pseudo*-quin, 4H, $^3J_{HH}$ = 6.7 Hz, H4), 2.37 (td, 4H, $^3J_{HH}$ = 6.9 Hz, $^4J_{HP}$ = 3.4 Hz, H3), 3.68 (t, 4H, $^3J_{HH}$ = 6.5 Hz, H5), 7.33 (s(br), 4H, NH_4^+); **^{13}C{^{1}H} NMR** (DMSO-d$_6$, δ in ppm): 16.0 (d, $^3J_{CP}$ = 3.4 Hz, C3), 30.7 (d, $^4J_{CP}$ = 1.6 Hz, C4), 44.1 (s, C5), 85.5 (d, $^1J_{CP}$ = 212.9 Hz, C1), 91.4 (d, $^2J_{CP}$ = 38.6 Hz, C2); **^{31}P NMR** (DMSO-d$_6$, δ in ppm): – 33.6 (quin, $^4J_{PH}$ = 3.4 Hz); **elemental analysis:** calc. for $C_{10}H_{16}Cl_2NO_2P$: C 42.3, H 5.7, N 4.9, S 0.0; found: C 41.9, H 5.9, N 4.8, S 0.2.

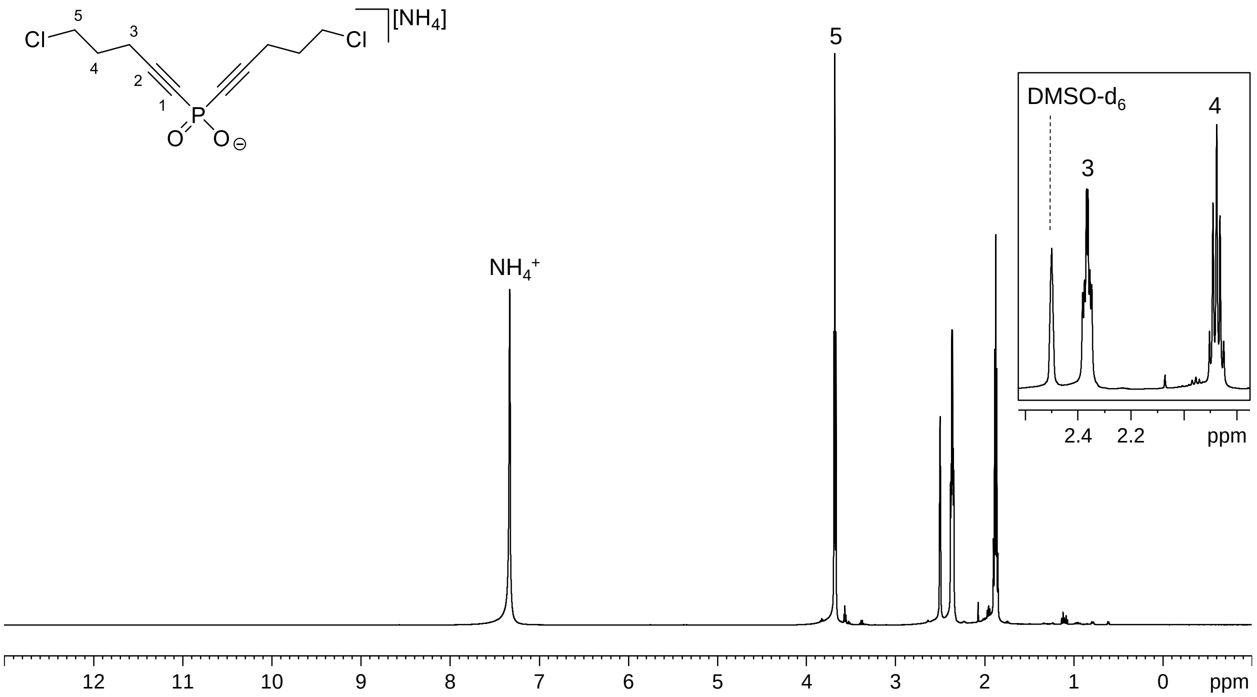

Fig. S 107. ^{1}H NMR spectrum (DMSO-d$_6$, 300 K) of ammonium bis(5-chloro-1-pentynyl)phosphinate (**9k** · NH_3) synthesized from **1b**[OTf].

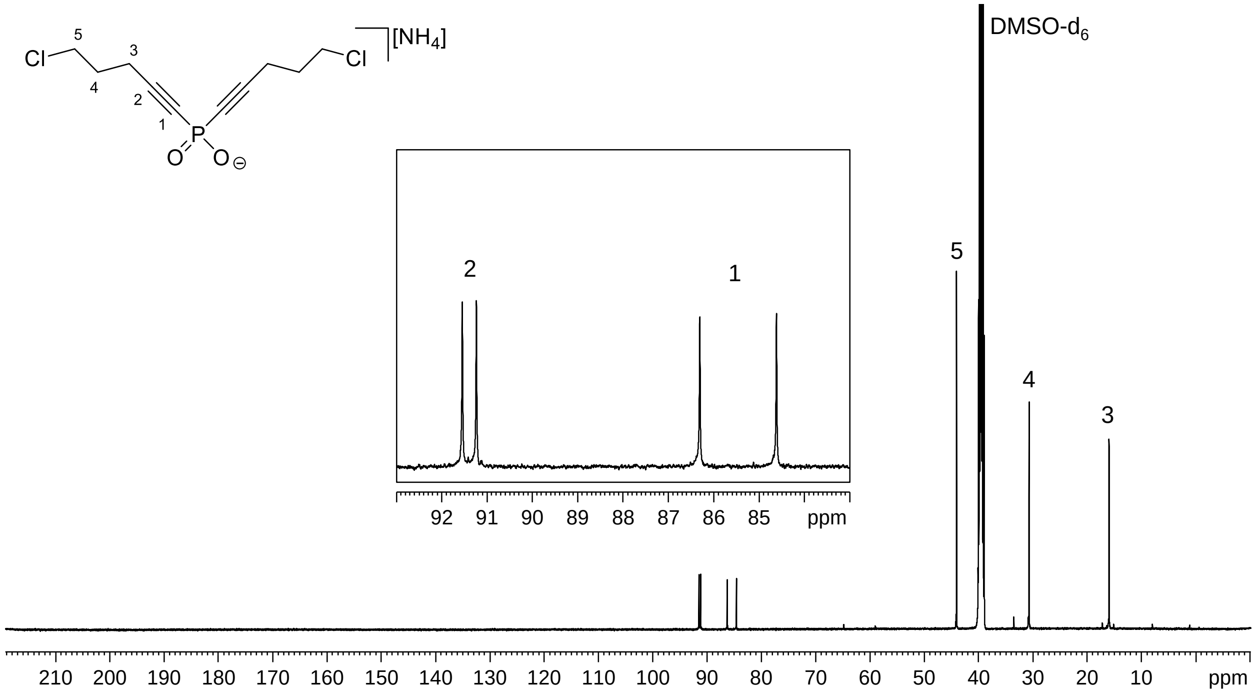

Fig. S 108. $^{13}C\{^{1}H\}$ NMR spectrum (DMSO-d$_6$, 300 K) of ammonium bis(5-chloro-1-pentynyl)phosphinate (**9k** · NH_3) synthesized from **1b**[OTf].

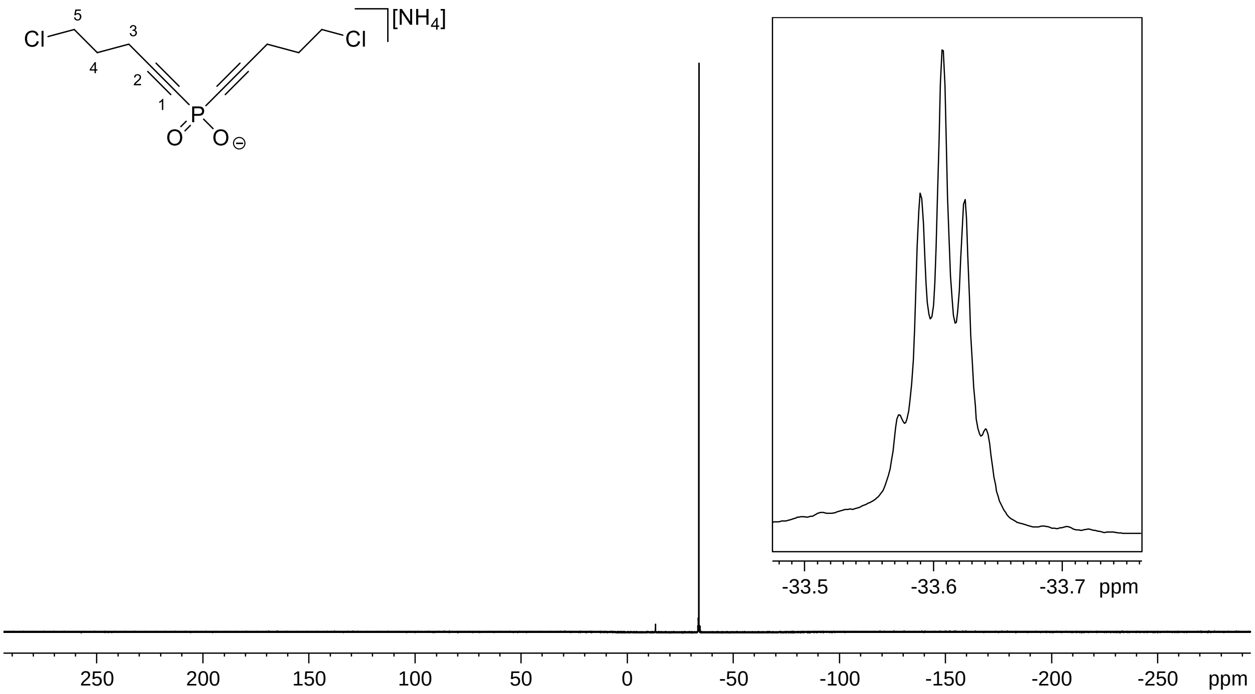

Fig. S 109. ^{31}P NMR spectrum (DMSO-d_6, 300 K) of ammonium bis(5-chloro-1-pentynyl)phosphinate (**9k** · NH_3) synthesized from **1b**[OTf].

2.5. Reduction of Dialkynylphosphinates

2.5.1. Preparation of Bis(phenylethyl)phosphinic acid (**10a**)

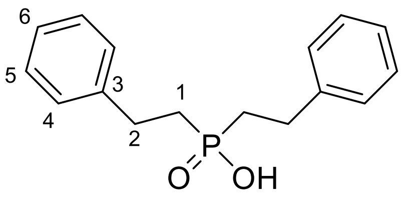

A thick-walled pressure reactor was charged with bis(phenylethynyl)phosphinic acid (**9b**, 532 mg, 2 mmol, 1 eq.) and palladium on carbon (53 mg, 10 % by weight). The solids were then purged with nitrogen before addition of 100 mL of EtOH. After passing nitrogen and hydrogen through the stirred suspension for 30 min each, the pressure release valve was closed and an atmosphere of 1.5 bar hydrogen was adjusted. The suspension was then further stirred for 16 hours and subsequently purged with nitrogen. After filtration, drying the filtrate *in vacuo* gave **10a** as a colorless powder. Single crystals of **10a** suitable for single crystal structure determination were obtained by slow evaporation of *n*-pentane into a concentrated CH_2Cl_2 solution (Fig. S 110).

Yield: 503 mg (92 %); **Raman** ($\tilde{\nu}$ in cm^{-1}): 3063 (75), 3055 (100), 3040 (41), 3030 (24), 3003 (11), 2944 (33), 2935 (36), 2913 (71), 2869 (17), 1602 (39), 1584 (23), 1458 (5), 1418 (15), 1206 (21), 1183 (17), 1162 (7), 1153 (7), 1031 (21), 1002 (81), 989 (8), 866 (9), 837 (13), 789 (14), 673 (11), 621 (18), 505 (7), 494 (9), 264 (5), 239 (10), 191 (15); **IR** (ATR, $\tilde{\nu}$ in cm^{-1}): 3085 (vw), 3061 (vw), 3028 (vw), 3004 (vw), 2944 (vw), 2913 (vw), 2864 (vw), 2631 (vw), 2285 (vw), 2135 (vw), 1601 (w), 1584 (w), 1494 (w), 1454 (w), 1414 (w), 1395 (w), 1273 (w), 1218 (w), 1178 (w), 1135 (m), 1072 (w), 1030 (m), 1013 (m), 970 (vs), 947 (s), 930 (s), 856 (s), 834 (m), 785 (s), 749 (s), 737 (vs), 725 (s), 698 (vs), 667 (m), 621 (w), 587 (w), 572 (m), 562 (m), 501 (m), 465 (vs), 422 (s); **m.p.**: 94 – 96 °C; **^{1}H NMR** (CD_2Cl_2, δ in ppm): 1.99–2.07 (m, 4H, H1), 2.90–2.97 (m, 4H, H2), 7.18–7.22 (m, 6H, H4/6), 7.26–7.31 (m, 4H, H5), 13.25 (s(br), 1H, P–OH); **^{13}C{^{1}H} NMR** (CD_2Cl_2, δ in ppm): 28.1 (d, $^2J_{CP}$ = 3.4 Hz, C2), 31.3 (d, $^1J_{CP}$ = 90.2 Hz, C1), 126.7 (s, C6), 128.5 (s, C4), 128.9 (s, C5), 141.7 (d, $^3J_{CP}$ = 15.1 Hz, C3); **^{31}P NMR** (CD_2Cl_2, δ in ppm): 56.1 (m); **elemental analysis:** calc. for $C_{16}H_{19}O_2P$: C 70.1, H 7.0, N 0.0, S 0.0; found: C 69.7, H 7.3, N 0.0, S 0.0.

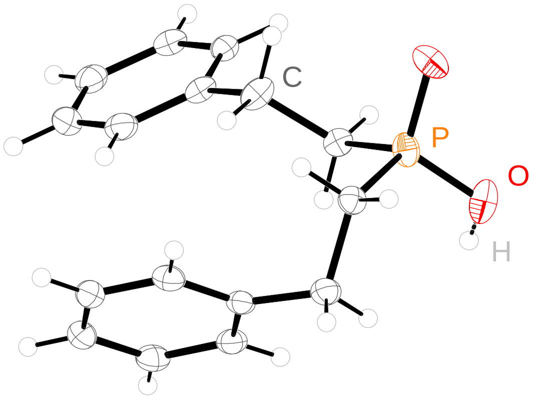

Fig. S 110. Molecular structure of bis(phenylethyl)phosphinic acid (**10a**); thermal ellipsoids are displayed at 50 % probability level; CCDC 2442199.

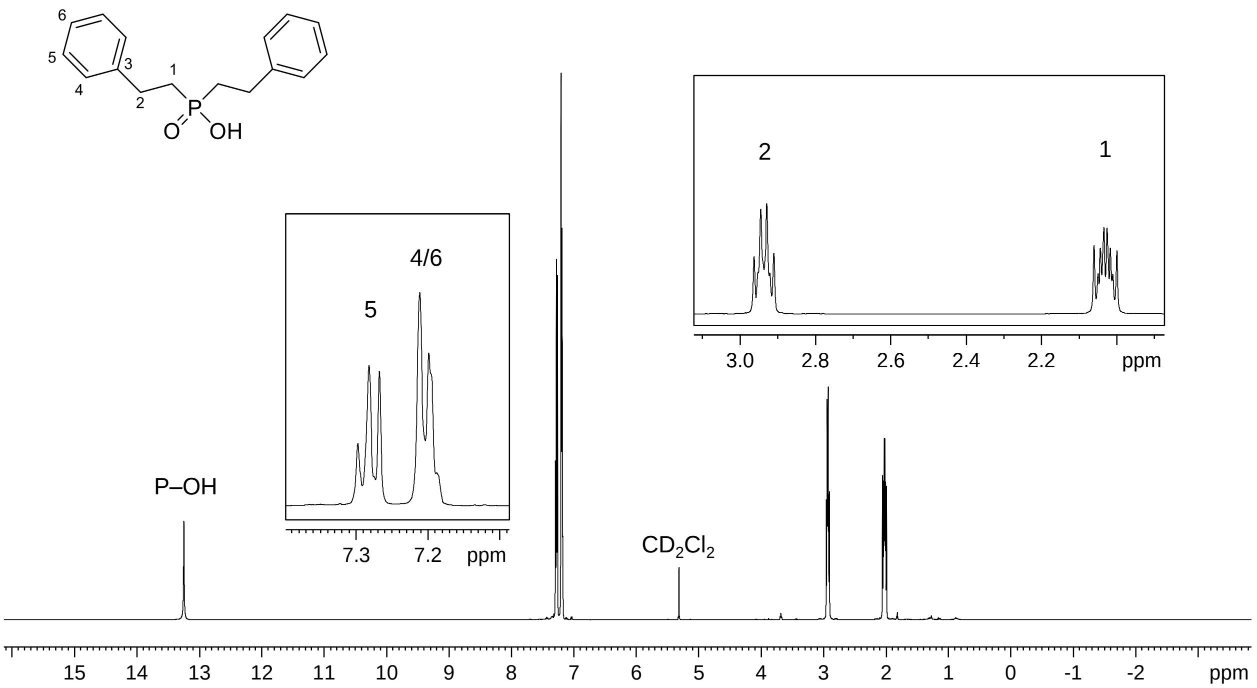

Fig. S 111. ^{1}H NMR spectrum (CD_2Cl_2, 300 K) of bis(phenylethyl)phosphinic acid (**10a**) synthesized from **9b**.

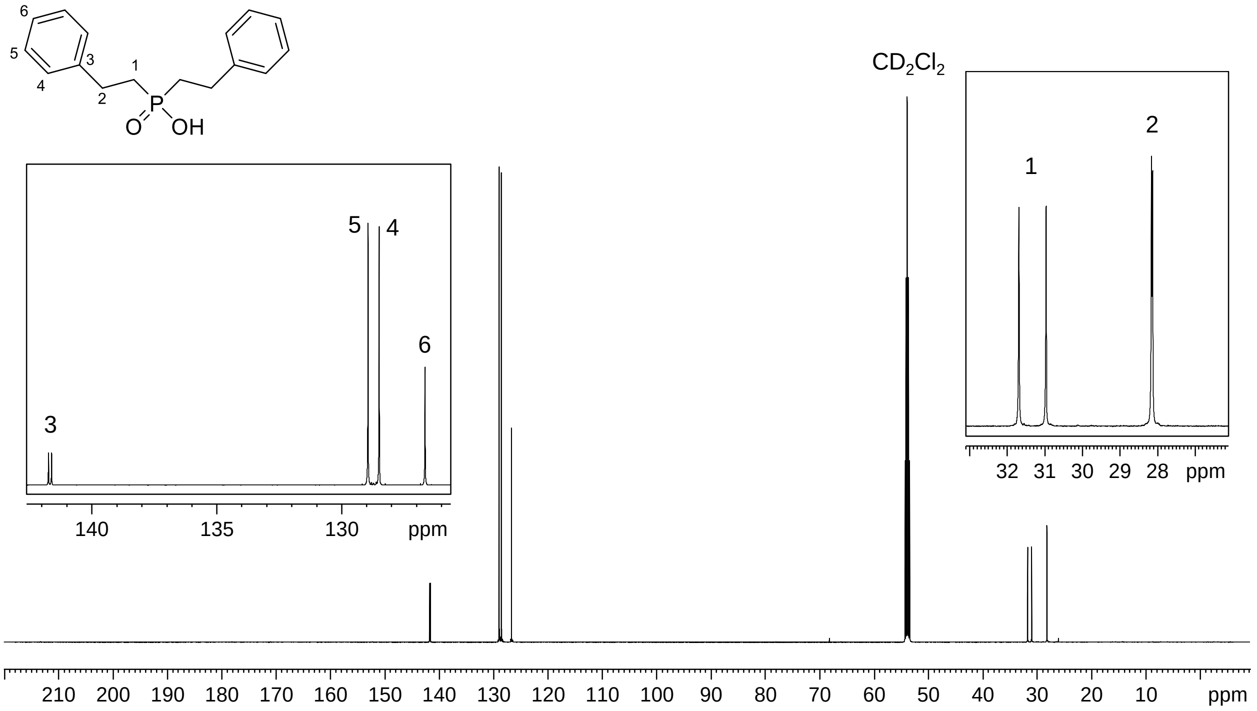

Fig. S 112. $^{13}C\{^{1}H\}$ NMR spectrum (CD_2Cl_2, 300 K) of bis(phenylethyl)phosphinic acid (**10a**) synthesized from **9b**.

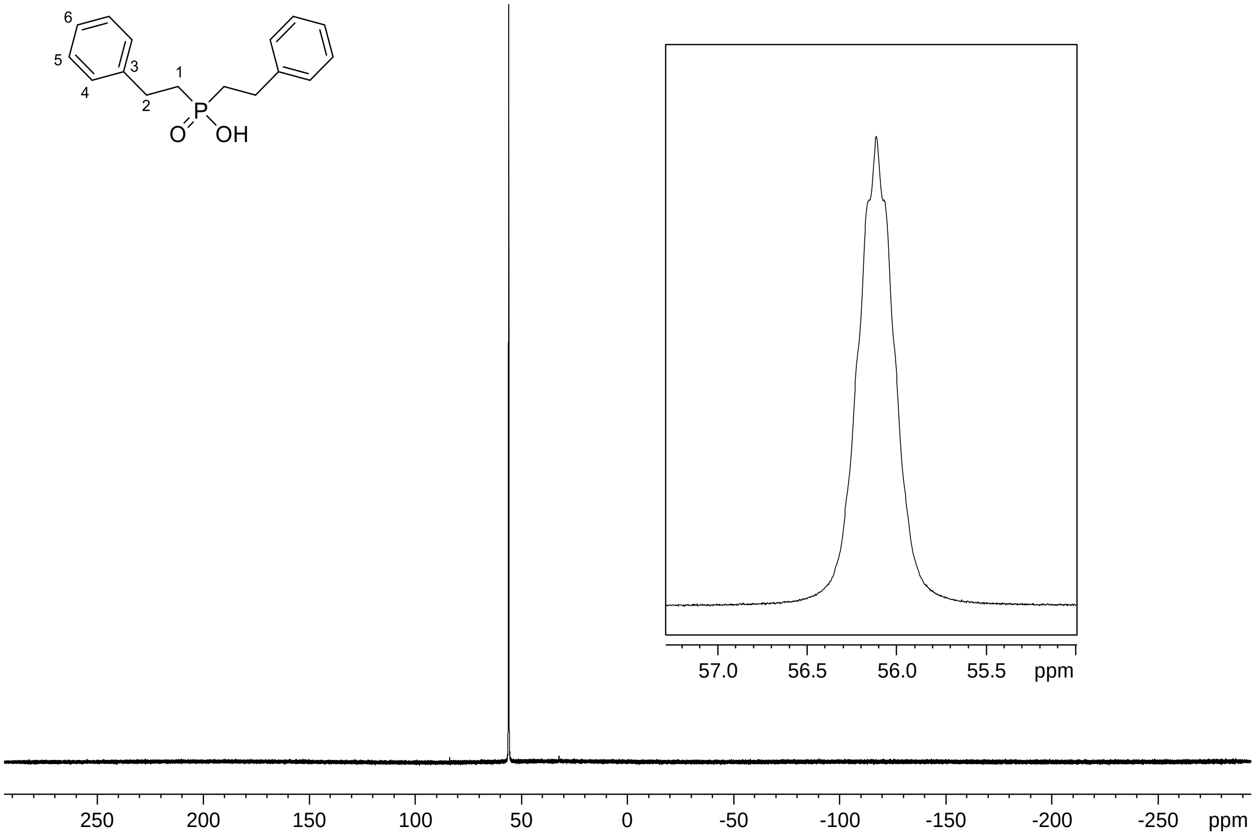

Fig. S 113. ^{31}P NMR spectrum (CD_2Cl_2, 300 K) of bis(phenylethyl)phosphinic acid (**10a**) synthesized from **9b**.

2.5.2. Preparation of Dihexylphosphinic acid (**10b**)

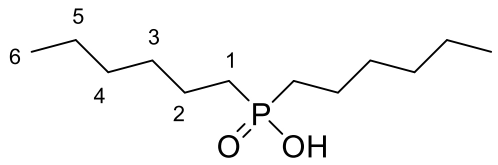

A thick-walled pressure reactor was charged with ammonium bis(1-hexynyl)phosphinate (**9j** · NH_3, 487 mg, 2 mmol, 1 eq.) and palladium on carbon (49 mg, 10 % by weight). The solids were then purged with nitrogen before addition of 100 mL of EtOH. After passing nitrogen and hydrogen through the stirred suspension for 30 min each, the pressure release valve was closed and an atmosphere of 1.5 bar hydrogen was adjusted. The suspension was then further stirred for 16 hours and subsequently purged with nitrogen. After filtration, drying the filtrate *in vacuo* gave the crude product as an orange-colored wax, which was converted into the free acid by washing with 1 M HCl under ultra-sonification. Subsequent filtration and drying *in vacuo* then afforded dihexylphosphinic acid (**10b**) as a colorless solid.

Single crystals of **10b** suitable for single crystal structure determination were obtained by cooling a saturated solution in toluene to – 30 °C (Fig. S 114).

Yield: 441 mg (94 %); **Raman** ($\tilde{\nu}$ in cm^{-1}): 2961 (31), 2914 (66), 2881 (100), 2860 (52), 2731 (7), 2703 (5), 1458 (21), 1442 (13), 1424 (7), 1300 (13), 1115 (13), 1061 (13), 1014 (5), 892 (10), 773 (5), 762 (7), 171 (26), 108 (13), 85 (18); **IR** (ATR, $\tilde{\nu}$ in cm^{-1}): 2953 (m), 2921 (s), 2871 (w), 2857 (w), 2847 (w), 1604 (w), 1466 (s), 1407 (w), 1378 (w), 1357 (w), 1316 (w), 1260 (m), 1240 (w), 1207 (w), 1146 (s), 1110 (m), 1063 (w), 1046 (m), 996 (s), 957 (vs), 891 (m), 850 (w), 774 (s), 755 (s), 720 (s), 557 (vs), 513 (w), 481 (m), 465 (m), 429 (m); **m.p.**: 75 – 77 °C; **^{1}H NMR** (CD_2Cl_2, δ in ppm): 0.89 (t, 6H, $^3J_{HH}$ = 6.6 Hz, H6), 1.25–1.35 (m, 8H, H4/5), 1.38 (quin, 4H, $^3J_{HH}$ = 6.9 Hz, H3), 1.51–1.68 (m, 8H, H1/2), 12.87 (s(br), 1H, P–OH); **^{13}C{^{1}H} NMR** (CD_2Cl_2, δ in ppm): 14.2 (s, C6), 22.0 (d, $^2J_{CP}$ = 4.2 Hz, C2), 22.9 (s, C5), 29.4 (d, $^1J_{CP}$ = 92.1 Hz, C1), 31.0 (d, $^3J_{CP}$ = 15.2 Hz, C3), 31.8 (s, C4); **^{31}P NMR** (CD_2Cl_2, δ in ppm): 59.4 (m); **elemental analysis:** calc. for $C_{12}H_{27}O_2P$: C 61.5, H 11.6, N 0.0, S 0.0; found: C 61.5, H 11.4, N 0.0, S 0.1.

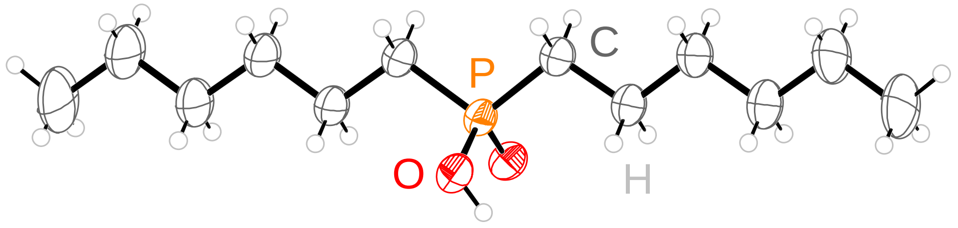

Fig. S 114. Molecular structure of dihexylphosphinic acid (**10b**); thermal ellipsoids are displayed at 50 % probability level; CCDC 2442195.

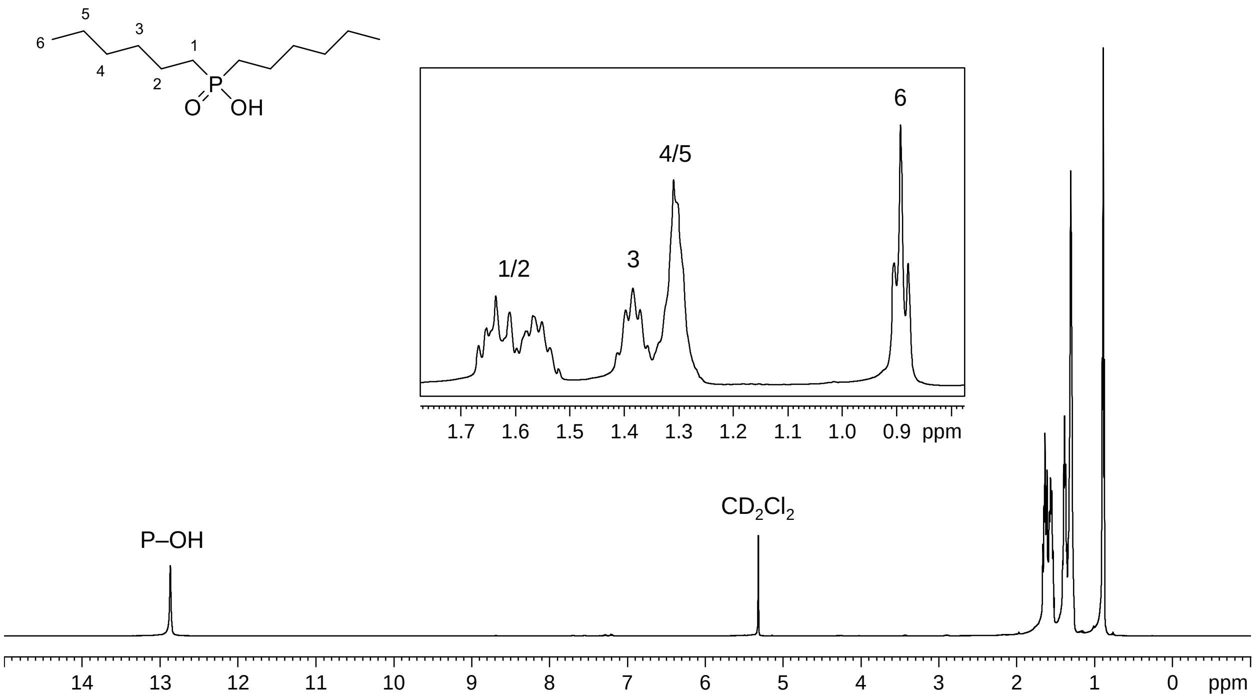

Fig. S 115. ^{1}H NMR spectrum (CD_2Cl_2, 300 K) of dihexylphosphinic acid (**10b**) synthesized from **9j** · NH_3.

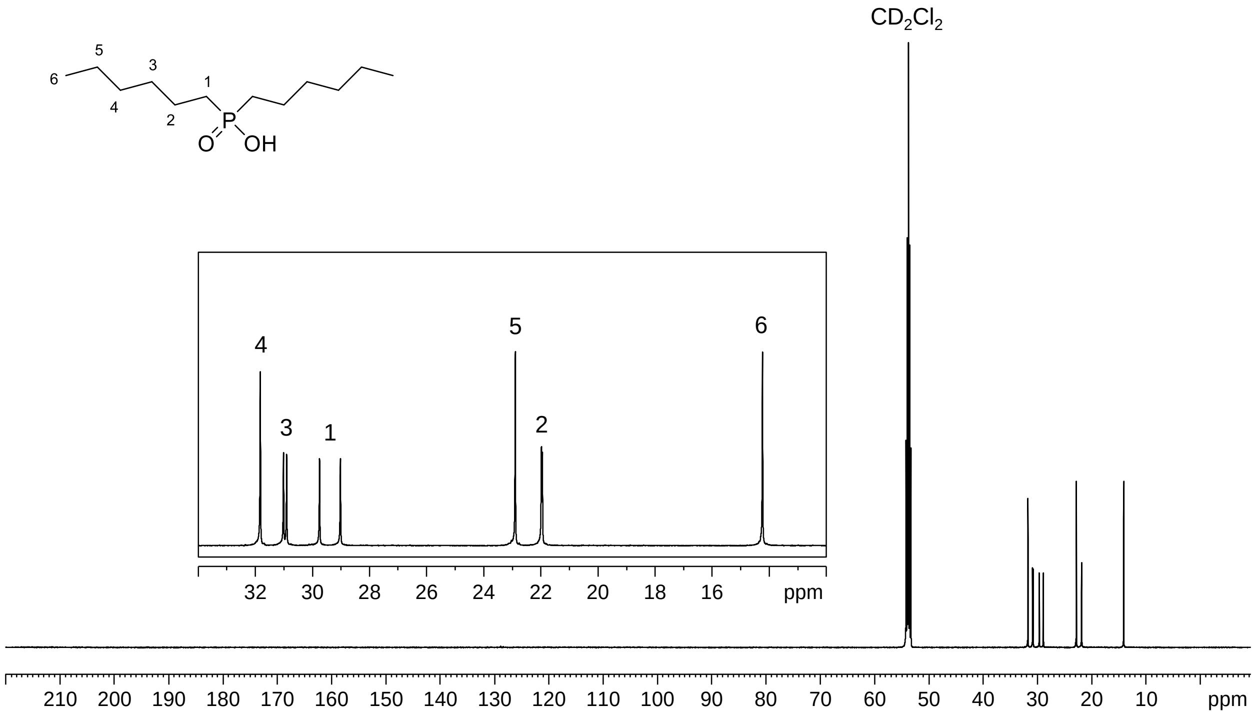

Fig. S 116. ^{13}C{^{1}H} NMR spectrum (CD_2Cl_2, 300 K) of dihexylphosphinic acid (**10b**) synthesized from **9j** · NH_3.

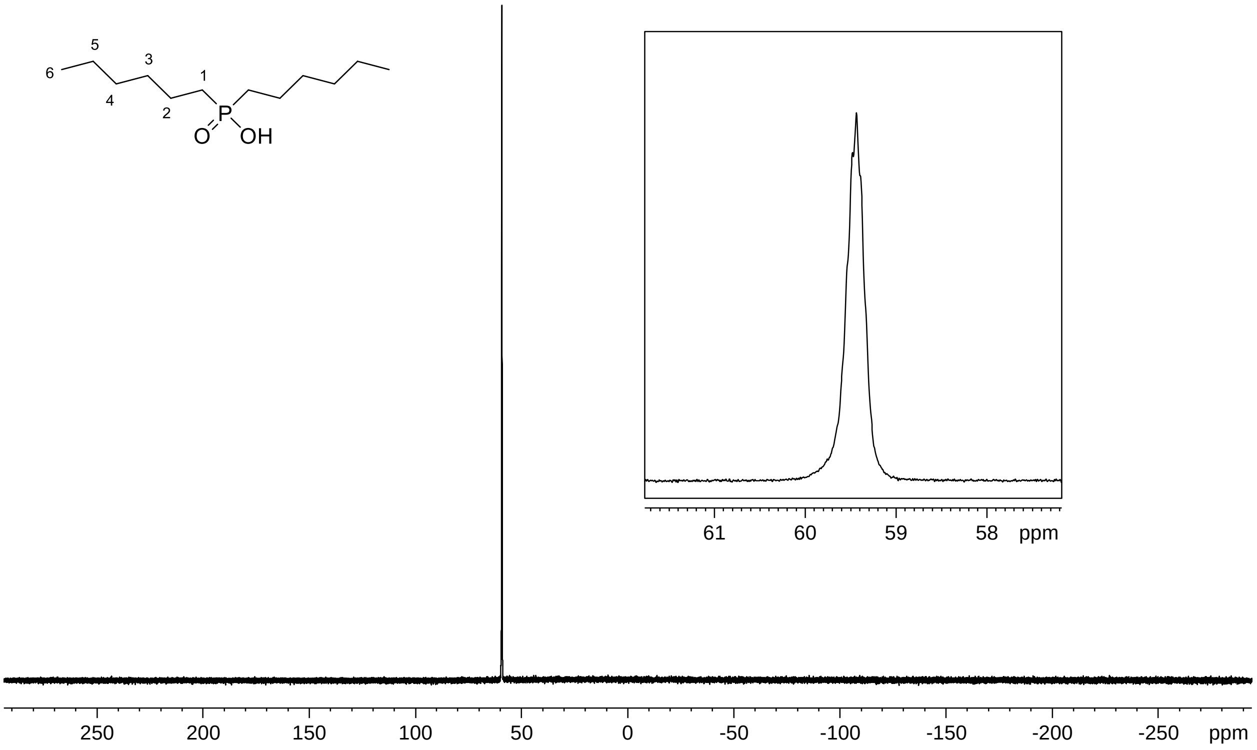

Fig. S 117. ^{31}P NMR spectrum (CD_2Cl_2, 300 K) of dihexylphosphinic acid (**10b**) synthesized from **9j** · NH_3.

3. X-ray Diffraction Refinements

3.1. General remarks

Suitable single crystals were coated with Paratone-N oil, mounted using a nylon loop, and frozen in a cold nitrogen stream. Unless otherwise stated, all crystals were measured at 100 K on a Rigaku Oxford Diffraction SuperNova diffractometer using Cu K_α radiation (λ = 1.54184 Å) or Mo K_α radiation (λ = 0.71073 Å), generated by a Nova or Mova micro-focus X-ray source. Reflections were collected with an Atlas S2 detector. Data reduction and absorption correction was performed with CrysaAlisPro[11] software. Using Olex2[12], the structures were solved with SHELXS/T[13] by direct methods and refined with SHELXL[14] by least-square minimization against F^2 using first isotropic and later anisotropic thermal parameters for all non-hydrogen atoms. Hydrogen atoms bonded to carbon atoms were added to the structure models on calculated positions using the riding model. All other hydrogen atoms were localized in the difference Fourier map. The position of the hydrogen atoms at oxygen or nitrogen atoms of the phosphinic acids, counter cations or co-crystallized solvent molecules could not be identified unambiguously due to hydrogen bonding on several instances. Images of the structures were produced with the Olex2 software.[12] All structures of this work have been deposited with the Cambridge Crystallographic Data Centre (CCDC) and can be accessed free of charge under the numbers CCDC 2242184 – 2242204.

In the figures containing molecular structures, each element is represented by a different color as follows: H (light grey), C (grey), N (blue), O (red), F (light green), Si (brown), P (orange), S (yellow), Cl (green).

In the structure refinement of **2g** and **9i** · H_2O, SADI, SIMU, RIGU, and EADP commands were used to model occurring disorders of the CF_3 and thiophenyl groups, respectively. Disorders of phenyl groups in the structural refinement of **9b** · H_2O and **9d** · H_2O · 0.5 Et_2O were refined without restraints or constraints. **9c** · H_2O · 0.5 C_6H_6 was modelled as an inversion twin with a ratio of 50:50. The crystal of **10b** was obtained, measured and refined as a twin (BASF = 0.435(2)) using the twin law [-0.0058 -0.9934 -0.0001 -1.0071 0.0057 0.0001 -0.4429 0.4421 -0.9998] at 200 K. In the refinement of the structure of **9d** · H_2O · 0.5 Et_2O, the PLATON/SQUEEZE[15] extension in Olex2 was used for a solvent mask of 0.5 Et_2O per asymmetric unit.

3.2. Crystallographic data

Table S 5. Crystallographic data of **2a**, **2b**, **2c** and **2d**.

	2a	2b	2c	2d
Empirical formula	$C_{12}H_{11}O_2P$	$C_{14}H_{15}O_2P$	$C_{16}H_{19}O_2P$	$C_{14}H_{15}O_4P$
Formula weight in (g mol^{-1})	218.18	246.23	274.28	278.23
Temperature in K	100.00(10)	99.98(17)	100.0(3)	100.0(3)
Crystal system	monoclinic	orthorhombic	triclinic	trigonal
Space group	$P2_1/c$	Pbca	P-1	R-3
a in Å	11.4391(2)	16.7209(5)	8.2961(3)	27.1618(9)
b in Å	5.94580(10)	5.8806(2)	8.4636(5)	27.1618(9)
c in Å	15.6097(4)	26.3519(10)	12.2467(6)	9.3344(5)
α in °	90	90	90.104(4)	90
β in °	100.167(2)	90	106.852(4)	90
γ in °	90	90	118.200(5)	120
Volume in Å^3	1045.02(4)	2591.15(15)	715.40(7)	5963.9(5)
Z	4	8	2	18
ρ_c in (g cm^{-3})	1.387	1.262	1.273	1.394
μ in mm^{-1}	2.134	1.777	1.660	1.921
F(000)	456.0	1040.0	292.0	2628.0
Crystal size in mm^3	0.19 x 0.07 x 0.05	0.33 x 0.11 x 0.05	0.25 x 0.1 x 0.02	0.43 x 0.03 x 0.02
Diffractometer	Rigaku OD Supernova	Rigaku OD Supernova	Rigaku OD Supernova	Rigaku OD Supernova
$\lambda_{XK\alpha}$ in Å	1.54184 (X = Cu)	1.54184 (X = Cu)	1.54184 (X = Cu)	1.54184 (X = Cu)
θ_{min} in °	7.852	6.708	7.646	6.508
θ_{max} in °	153.408	154.47	153.39	153.482
Index ranges	$-14 \le h \le 12$<br>$-7 \le k \le 6$<br>$-18 \le l \le 19$	$-18 \le h \le 21$<br>$-5 \le k \le 7$<br>$-29 \le l \le 32$	$-10 \le h \le 8$<br>$-10 \le k \le 10$<br>$-12 \le l \le 15$	$-34 \le h \le 24$<br>$-34 \le k \le 33$<br>$-11 \le l \le 11$
Reflections collected	6085	18416	7103	11856
Independent reflections	2167	2719	2971	2747
R_{int} / R_{sigma}	0.0338 / 0.0354	0.0584 / 0.0327	0.0414 / 0.0522	0.0566 / 0.0391
Data / restraints / parameters	2167 / 0 / 137	2719 / 0 / 157	2971 / 0 / 177	2747 / 0 / 175
GooF on F^2	1.111	1.078	1.032	1.079
Final R indices [I≥2σ(I)]	R_1 = 0.0560, wR_2 = 0.1471	R_1 = 0.0418, wR_2 = 0.1154	R_1 = 0.0497, wR_2 = 0.1289	R_1 = 0.0475, wR_2 = 0.1272
Final R indices (all data)	R_1 = 0.0612, wR_2 = 0.1504	R_1 = 0.0453, wR_2 = 0.1206	R_1 = 0.0610, wR_2 = 0.1393	R_1 = 0.0579, wR_2 = 0.1356
Largest diff. peak and hole in (e Å^3)	0.63 and – 0.47	0.32 and – 0.49	0.60 and – 0.56	0.50 and – 0.33
CCDC	2442184	2442190	2442203	2442191

Table S 6. Crystallographic data of **2e, 2f**, **2g** and **2h**.

	**2e**	**2f**	**2g**	**2h**
Empirical formula	$C_{14}H_{15}O_4P$	$C_{12}H_9F_2O_2P$	$C_{16}H_7F_{12}O_2P$	$C_{16}H_{15}O_2P$
Formula weight in (g mol^{-1})	278.23	254.16	490.19	270.25
Temperature in K	100.0(3)	100.0(3)	100.01(10)	100.00(10)
Crystal system	monoclinic	monoclinic	monoclinic	trigonal
Space group	C2/c	Pn	$P2_1/n$	R-3
a in Å	17.9131(3)	10.1089(2)	16.0174(3)	27.7974(6)
b in Å	10.3569(2)	14.7735(2)	6.86980(10)	27.7974(6)
c in Å	7.10510(10)	15.5714(2)	16.2295(3)	9.3292(4)
α in °	90	90	90	90
β in °	96.541(2)	90.383(2)	91.720(2)	90
γ in °	90	90	90	120
Volume in Å^3	1309.59(4)	2325.44(6)	1785.03(5)	6242.9(4)
Z	4	8	4	18
ρ_c in (g cm^{-3})	1.411	1.452	1.824	1.294
μ in mm^{-1}	1.944	2.260	0.287	1.712
F(000)	584.0	1040.0	968.0	2556.0
Crystal size in mm^3	0.18 x 0.14 x 0.12	0.29 x 0.04 x 0.03	0.24 x 0.121 x 0.088	0.17 x 0.06 x 0.04
Diffractometer	Rigaku OD Supernova	Rigaku OD Supernova	Rigaku OD Supernova	Rigaku OD Supernova
$\lambda_{XK\alpha}$ in Å	1.54184 (X = Cu)	1.54184 (X = Cu)	1.54184 (X = Cu)	1.54184 (X = Cu)
θ_{min} in °	9.882	5.982	5.022	6.36
θ_{max} in °	144.228	153.63	65.82	136.44
Index ranges	$-22 \leq h \leq 20$<br>$-12 \leq k \leq 12$<br>$-8 \leq l \leq 8$	$-12 \leq h \leq 12$<br>$-18 \leq k \leq 18$<br>$-12 \leq l \leq 19$	$-23 \leq h \leq 22$<br>$-9 \leq k \leq 10$<br>$-24 \leq l \leq 24$	$-24 \leq h \leq 33$<br>$-33 \leq k \leq 28$<br>$-11 \leq l \leq 11$
Reflections collected	6046	22349	19169	13984
Independent reflections	1300	7106	6030	2550
R_{int} / R_{sigma}	0.0193 / 0.0140	0.0593 / 0.0628	0.0241 / 0.0317	0.0648 / 0.0389
Data / restraints / parameters	1300 / 0 / 89	7106 / 2 / 618	6030 / 366 / 365	2550 / 0 / 173
GooF on F^2	1.219	1.054	1.032	1.041
Final R indices [I≥2σ(I)]	R_1 = 0.0322, wR_2 = 0.0829	R_1 = 0.0474, wR_2 = 0.1192	R_1 = 0.0592, wR_2 = 0.1500	R_1 = 0.0499, wR_2 = 0.1333
Final R indices (all data)	R_1 = 0.0323, wR_2 = 0.0830	R_1 = 0.0528, wR_2 = 0.1237	R_1 = 0.0769, wR_2 = 0.1639	R_1 = 0.0597, wR_2 = 0.1433
Largest diff. peak and hole in (e Å^3)	0.24 and – 0.47	0.39 and – 0.40	0.92 and – 0.59	0.44 and – 0.34
CCDC	2442185	2442192	2442202	2442201

Table S 7. Crystallographic data of **7b**, **9a**, **9b** and **9c**.

	**7b**	**9a**	**9b** · H_2O	**9c** · H_2O · 0.5 C_6H_6
Empirical formula	$C_{11}H_9N_2O_5P$	$C_{10}H_{19}O_2PSi_2$	$C_{16}H_{13}O_3P$	$C_{21}H_{20}O_3P$
Formula weight in (g mol^{-1})	280.17	258.40	284.23	351.34
Temperature in K	100.00(10)	100.01(10)	101(1)	100.0(3)
Crystal system	orthorhombic	monoclinic	monoclinic	monoclinic
Space group	Pbca	I2/m	P2$_1$/c	Ia
a in Å	12.3972(3)	13.4443(2)	13.8597(13)	22.3980(2)
b in Å	10.9873(2)	7.51900(10)	5.8984(6)	6.08080(10)
c in Å	17.6571(4)	15.7509(2)	18.1497(17)	27.5888(3)
α in °	90	90	90	90
β in °	90	102.4460(10)	101.500(9)	91.0070(10)
γ in °	90	90	90	90
Volume in Å^3	2405.10(9)	1554.80(4)	1454.0(2)	3756.95(8)
Z	8	4	4	8
ρ_c in (g cm^{-3})	1.547	1.104	1.298	1.242
μ in mm^{-1}	2.242	2.917	1.716	1.425
F(000)	1152.0	552.0	592.0	1480.0
Crystal size in mm^3	0.33 x 0.09 x 0.03	0.4 x 0.26 x 0.06	0.187 x 0.029 x 0.01	0.37 x 0.1 x 0.05
Diffractometer	Rigaku OD Supernova	Rigaku OD Supernova	Rigaku OD Supernova	Rigaku OD Supernova
$\lambda_{XK\alpha}$ in Å	1.54184 (X = Cu)	1.54184 (X = Cu)	1.54184 (X = Cu)	1.54184 (X = Cu)
θ_{min} in °	10.02	7.856	6.508	6.408
θ_{max} in °	153.172	153.124	140.04	153.036
Index ranges	$-15 \le h \le 15$<br>$-12 \le k \le 13$<br>$-22 \le l \le 22$	$-13 \le h \le 16$<br>$-9 \le k \le 9$<br>$-19 \le l \le 19$	$-16 \le h \le 16$<br>$-7 \le k \le 7$<br>$-22 \le l \le 22$	$-28 \le h \le 28$<br>$-7 \le k \le 6$<br>$-34 \le l \le 34$
Reflections collected	26659	7561	8003	22024
Independent reflections	2528	1757	2744	6849
R_{int} / R_{sigma}	0.0569 / 0.0240	0.0424 / 0.0308	0.0579 / 0.0650	0.0297 / 0.0296
Data / restraints / parameters	2528 / 0 / 172	1757 / 0 / 85	2744 / 0 / 216	6849 / 2 / 475
GooF on F^2	1.077	1.051	1.038	1.024
Final R indices [I≥2σ(I)]	R_1 = 0.0389, wR_2 = 0.1064	R_1 = 0.0296, wR_2 = 0.0792	R_1 = 0.0586, wR_2 = 0.1434	R_1 = 0.0343, wR_2 = 0.0882
Final R indices (all data)	R_1 = 0.0442, wR_2 = 0.1114	R_1 = 0.0324, wR_2 = 0.0821	R_1 = 0.0769, wR_2 = 0.1569	R_1 = 0.0371, wR_2 = 0.0911
Largest diff. peak and hole in (e Å^3)	0.42 and – 0.37	0.26 and – 0.35	0.53 and – 0.39	0.25 and – 0.29
Flack parameter	-	-	-	0.50(2)
CCDC	2442189	2442198	2442204	2442196

Table S 8 Crystallographic data of **9d**, **9e**, **9f** and **9g**.

	**9d** · H_2O · 0.5 Et_2O	**9e**	**9f**	**9g** · Py
Empirical formula	$C_{26}H_{34}O_{3.5}P$	$C_{16}H_9O_2F_2P$	$C_{16}H_9O_2PCl_2$	$C_{23}H_{14}F_6NO_2P$
Formula weight in (g mol^{-1})	433.50	302.20	335.10	481.32
Temperature in K	99.99(10)	100.01(10)	100.01(10)	100.00(10)
Crystal system	monoclinic	monoclinic	monoclinic	triclinic
Space group	I2/a	I2/a	C2/c	P-1
a in Å	24.9323(5)	7.0675(2)	30.5771(5)	7.1542(2)
b in Å	6.17430(10)	6.9360(2)	6.59710(10)	7.7424(3)
c in Å	33.2509(6)	28.1161(9)	7.27900(10)	19.1720(5)
α in °	90	90	90	87.878(2)
β in °	99.364(2)	92.929(3)	93.963(2)	86.632(2)
γ in °	90	90	90	85.010(2)
Volume in Å^3	5050.42(16)	1376.46(7)	1464.81(4)	1055.57(6)
Z	8	4	4	2
ρ_c in (g cm^{-3})	1.140	1.458	1.520	1.514
μ in mm^{-1}	0.134	0.223	5.028	1.843
F(000)	1864.0	616.0	680.0	488.0
Crystal size in mm^3	0.67 x 0.13 x 0.03	0.35 x 0.15 x 0.02	0.23 x 0.12 x 0.01	0.15 x 0.11 x 0.04
Diffractometer	Rigaku OD Supernova	Rigaku OD Supernova	Rigaku OD Supernova	Rigaku OD Supernova
$\lambda_{XK\alpha}$ in Å	0.71073 (X = Mo)	0.71073 (X = Mo)	1.54184 (X = Cu)	1.54184 (X = Cu)
θ_{min} in °	6.404	5.804	5.794	4.62
θ_{max} in °	50.69	65.258	153.364	153.608
Index ranges	$-25 \leq h \leq 30$<br>$-7 \leq k \leq 7$<br>$-40 \leq l \leq 32$	$-9 \leq h \leq 10$<br>$-7 \leq k \leq 10$<br>$-31 \leq l \leq 39$	$-37 \leq h \leq 38$<br>$-6 \leq k \leq 8$<br>$-7 \leq l \leq 9$	$-8 \leq h \leq 9$<br>$-6 \leq k \leq 9$<br>$-23 \leq l \leq 24$
Reflections collected	18649	7843	6457	10342
Independent reflections	4563	2257	1530	4374
R_{int} / R_{sigma}	0.0237 / 0.0234	0.0255 / 0.0315	0.0316 / 0.0224	0.0259 / 0.0319
Data / restraints / parameters	4563 / 348 / 326	2257 / 0 / 97	1530 / 0 / 97	4374 / 0 / 298
GooF on F^2	1.042	1.051	1.046	1.038
Final R indices [I≥2σ(I)]	R_1 = 0.0522, wR_2 = 0.1267	R_1 = 0.0407, wR_2 = 0.1009	R_1 = 0.0273, wR_2 = 0.0722	R_1 = 0.0370, wR_2 = 0.0900
Final R indices (all data)	R_1 = 0.0599, wR_2 = 0.1314	R_1 = 0.0530, wR_2 = 0.1078	R_1 = 0.0291, wR_2 = 0.0736	R_1 = 0.0423, wR_2 = 0.0939
Largest diff. peak and hole in (e Å^3)	0.36 and – 0.45	0.35 and – 0.32	0.29 and – 0.38	0.41 and – 0.38
CCDC	2442193	2442194	2442186	2442187

Table S 9. Crystallographic data of **9h**, **9i**, **9j** and **10a**.

	**9h** · NH_3 · THF	**9i** · H_2O	**9j** · NH_3	**10a**
Empirical formula	$C_{28}H_{26}NO_3P$	$C_{12}H_9O_3PS_2$	$C_{12}H_{22}NO_2P$	$C_{16}H_{19}O_2P$
Formula weight in (g mol^{-1})	455.47	296.28	243.27	274.28
Temperature in K	100.00(10)	100.01(10)	100.01(10)	100.00(10)
Crystal system	orthorhombic	monoclinic	orthorhombic	triclinic
Space group	$P2_12_12_1$	$P2_1/n$	Pbca	P-1
a in Å	6.15620(10)	12.5423(3)	10.3632(3)	6.0955(2)
b in Å	16.7127(3)	5.77350(10)	8.4028(2)	7.4859(3)
c in Å	23.8964(5)	19.0950(6)	32.7880(11)	15.7245(5)
α in °	90	90	90	81.063(3)
β in °	90	108.789(3)	90	79.306(3)
γ in °	90	90	90	86.907(3)
Volume in $Å^3$	2458.62(8)	1309.04(6)	2855.18(14)	696.26(4)
Z	4	4	8	2
ρ_c in (g cm^{-3})	1.230	1.503	1.132	1.308
μ in mm^{-1}	0.141	4.833	0.181	1.706
F(000)	960.0	608.0	1056.0	292.0
Crystal size in mm^3	0.18 x 0.08 x 0.01	0.31 x 0.043 x 0.007	0.31 x 0.18 x 0.02	0.217 x 0.122 x 0.101
Diffractometer	Rigaku OD Supernova	Rigaku OD Supernova	Rigaku OD Supernova	Rigaku OD Supernova
$\lambda_{XK\alpha}$ in Å	0.71073 (X = Mo)	1.54184 (X = Cu)	0.71073 (X = Mo)	1.54184 (X = Cu)
θ_{min} in °	4.874	7.476	4.65	5.784
θ_{max} in °	61.818	144.204	64.942	153.326
Index ranges	$-8 \le h \le 8$<br>$-23 \le k \le 22$<br>$-34 \le l \le 31$	$-10 \le h \le 15$<br>$-7 \le k \le 7$<br>$-23 \le l \le 23$	$-13 \le h \le 15$<br>$-12 \le k \le 8$<br>$-32 \le l \le 46$	$-7 \le h \le 7$<br>$-9 \le k \le 9$<br>$-18 \le l \le 19$
Reflections collected	28962	10740	19230	6581
Independent reflections	6994	2571	4667	2882
R_{int} / R_{sigma}	0.0386 / 0.0468	0.0549 / 0.0467	0.0666 / 0.0715	0.0225 / 0.0281
Data / restraints / parameters	6994 / 0 / 314	2571 / 181 / 199	4667 / 0 / 163	2882 / 0 / 174
GooF on F^2	1.066	1.067	1.038	1.117
Final R indices [I≥2σ(I)]	R_1 = 0.0435, wR_2 = 0.0865	R_1 = 0.0539, wR_2 = 0.1416	R_1 = 0.0630, wR_2 = 0.1347	R_1 = 0.0412, wR_2 = 0.1040
Final R indices (all data)	R_1 = 0.0563, wR_2 = 0.0912	R_1 = 0.0620, wR_2 = 0.1500	R_1 = 0.1008, wR_2 = 0.1553	R_1 = 0.0439, wR_2 = 0.1064
Largest diff. peak and hole in (e $Å^3$)	0.43 and – 0.32	0.97 and – 0.52	0.51 and – 0.50	0.37 and – 0.44
Flack parameter	0.00(3)	-	-	-
CCDC	2442188	2442197	2442200	2442199

Table S 10. Crystallographic data of **10b**.

	**10b**
Empirical formula	$C_{12}H_{27}O_2P$
Formula weight in (g mol^{-1})	234.30
Temperature in K	200.01(10)
Crystal system	triclinic
Space group	P-1
a in Å	6.5470(5)
b in Å	6.5949(6)
c in Å	17.8182(12)
α in °	81.994(6)
β in °	89.619(6)
γ in °	79.077(7)
Volume in Å^3	747.88(10)
Z	2
ρ_c in (g cm^{-3})	1.040
μ in mm^{-1}	1.493
F(000)	260.0
Crystal size in mm^3	0.217 x 0.071 x 0.066
Diffractometer	Rigaku OD Supernova
λ_{XK}[illegible] in Å	1.54184 (X = Cu)
θ_{min} in °	5.01
θ_{max} in °	153.198
Index ranges	$-8 \le h \le 8$<br>$-8 \le k \le 8$<br>$-22 \le l \le 22$
Reflections collected	5260
Independent reflections	5260
R_{int} / R_{sigma}	? / 0.0207
Data / restraints / parameters	5260 / 0 / 140
GooF on F^2	1.065
Final R indices [I≥2σ(I)]	R_1 = 0.0587,<br>wR_2 = 0.1741
Final R indices (all data)	R_1 = 0.0673,<br>wR_2 = 0.1793
Largest diff. peak and hole in (e Å^3)	0.51 and – 0.42
CCDC	2442195

4. Computational Investigations

4.1. Theoretical methods

The geometries and energies of all systems included in this study were fully optimized at the RI-BP86-D4/def2-TZVP level of theory without symmetry constrains. The calculations have been performed by using the program TURBOMOLE version 7.7.[16] For the calculations we have used the BP86[17,18] functional with the D4 correction for dispersion.[19] The triple-zeta quality def2-TZVP basis set[20] was used as a good compromise between the accuracy of the results and the size of the systems. In order to reproduce solvent effects, we have used the conductor-like screening model COSMO,[21] which is a variant of the dielectric continuum solvation models. The minimum nature of the complexes and compounds have been confirmed by doing frequency calculations. The Gibbs free energy values have been calculated at 298.15 K and 0.1 Mpa.

4.2. Cartesian Coordinates

7a⁺

P	9.6995797	-1.1811616	2.0774392
C	10.0478593	-3.9352892	2.1611345
H	10.0253057	-3.7136811	3.2269071
C	12.0356894	-0.0133198	1.1321497
H	11.3712139	0.1832527	0.2923331
C	9.8703106	-3.0101544	-0.0056267
H	9.7157222	-2.0920670	-0.5701608
C	14.0921795	0.1620141	2.3568386
C	9.9615358	-4.2685152	-0.5782368
H	9.9054439	-4.3679169	-1.6608829
C	13.3479869	0.4251689	1.2038644
H	13.7725970	0.9781539	0.3678454
C	12.1833958	-0.9477432	3.2970465
H	11.6302399	-1.4543226	4.0863610
C	10.1051145	-5.3849152	0.2497149
C	13.4987242	-0.5293150	3.4162025
H	14.0430154	-0.7338112	4.3366129
C	10.1445108	-5.2135849	1.6359483
H	10.2341880	-6.0629308	2.3109307
O	9.3251113	-1.4448980	3.4784495
O	9.1521196	-0.3690047	0.9758628
H	10.1714879	-6.3838049	-0.1819359
N	11.4828321	-0.6975044	2.1637184
H	15.1236973	0.5061309	2.4350550
N	9.9276910	-2.8624745	1.3406764

8a^{2+}

O	-0.6345113	3.3816214	-0.7093960
O	-1.6881761	1.8861169	3.9723902
P	-0.9193338	3.0717454	3.5318196
P	-1.3062272	2.9404938	0.5109100
C	-2.4674910	1.1109062	-1.0835441
C	-2.4448509	0.5286606	1.2305714
C	-3.1013016	-0.0764188	-1.3970792
H	-2.1624280	1.8491384	-1.8238424
C	-3.0817586	-0.6663633	0.9481063
H	-2.1453308	0.8233459	2.2422646
C	-3.4163388	-0.9774448	-0.3740162
H	-3.3345928	-0.2932215	-2.4386517
H	-3.3044525	-1.3505324	1.7658996
O	-0.5582117	2.6901021	1.8317957
C	-2.6811184	5.2218751	0.1835750
C	-3.5780696	3.7890407	1.8587043
C	-3.6380220	6.1786604	0.4666072
H	-1.9099914	5.3379697	-0.5763110
C	-4.5552851	4.7171755	2.1594283
H	-3.4917076	2.8454002	2.3953741
C	-4.5871006	5.9295259	1.4627051
H	-3.6320849	7.1123400	-0.0939522
H	-5.2740139	4.4929667	2.9458907
H	-5.3457631	6.6768544	1.6974134
C	1.1829954	1.6891574	4.6061111
C	1.5912746	3.9726214	4.0919218
C	2.4672944	1.5491768	5.1007139
H	0.4399337	0.8942061	4.6052352
C	2.8835953	3.8721782	4.5747328
H	1.1546514	4.8915798	3.7055220
C	3.3309832	2.6490617	5.0842368
H	2.7807537	0.5875644	5.5038498
H	3.5269540	4.7504080	4.5590932
H	4.3445975	2.5560776	5.4752991
O	-1.2527672	4.5062335	3.5501557
N	-2.1562983	1.3996727	0.2169701
H	-3.9120193	-1.9204013	-0.6077059
N	-2.6639258	4.0433538	0.8764396
N	0.7690851	2.8865172	4.1071475

Dioxophosphorane-a^{+}

P	9.4369440	-1.2455676	2.0870378
C	9.9503793	-3.9590823	2.2054976
H	9.9187566	-3.8256286	3.2863573
C	9.7457675	-2.8503701	1.3675795
C	9.7803648	-2.9862165	-0.0301912
H	9.6190521	-2.1095674	-0.6567540
C	10.0192848	-4.2406853	-0.5865569
H	10.0469696	-4.3576186	-1.6701549
C	10.2232282	-5.3470625	0.2446236

C	10.1893646	-5.2077328	1.6359407
H	10.3496667	-6.0748631	2.2768866
O	9.4181072	-1.2338493	3.5638529
O	9.2451871	-0.1667807	1.0965967
H	10.4099784	-6.3272249	-0.1962218

7b

P	9.7430097	-2.6290047	2.1502482
C	11.0704601	-0.4984808	0.8839207
H	10.4894739	-0.8975009	0.0535982
C	12.6238021	1.0024547	1.9275685
C	11.9027053	0.6095732	0.7992377
H	11.9836787	1.1484601	-0.1431365
C	11.6410973	-0.8186296	3.1430718
H	11.4762772	-1.4544036	4.0131197
C	12.4940875	0.2751151	3.1132996
H	13.0494585	0.5471738	4.0092336
O	10.2130830	-3.3798346	3.3316662
O	8.4742558	-1.6870459	2.6487596
N	10.9511489	-1.1795850	2.0420029
H	13.2877244	1.8660059	1.8822860
O	9.6198018	-3.0378370	0.7304267
C	7.8589532	-0.7696814	1.8278814
C	7.7213176	0.5319909	2.3302582
C	7.3597108	-1.1092781	0.5620747
C	7.0962464	1.5075823	1.5630917
H	8.1049036	0.7569938	3.3250610
C	6.7391730	-0.1309566	-0.2072515
H	7.4845000	-2.1256391	0.1949347
C	6.6184111	1.1646853	0.2977512
H	6.9736054	2.5273172	1.9222566
H	6.3408386	-0.3565283	-1.1946484
N	5.9688160	2.1996878	-0.5262635
O	5.8703135	3.3354899	-0.0480783
O	5.5655494	1.8720251	-1.6471051

8b

O	-1.7529502	-1.5594002	2.2493210
O	-1.6416424	2.4589991	-0.0032131
P	-0.4489115	1.6146323	-0.2296287
P	-1.3109301	-0.9308720	0.9985085
C	-3.8266009	-0.0062861	0.8048935
C	-2.7823813	-0.3497310	-1.2911568
C	-4.9164150	0.5843145	0.1961823
H	-3.7252532	-0.1402943	1.8803430
C	-3.8604873	0.2278094	-1.9419334
H	-1.8854231	-0.6878791	-1.7982319
C	-4.9328901	0.7097618	-1.1952051
H	-5.7346767	0.9522271	0.8115701

H	-3.8344321	0.3166929	-3.0258444
O	-0.5138511	0.3915141	1.0761523
O	-0.1702052	0.8169310	-1.4619471
N	-2.7867372	-0.4619497	0.0604293
H	-5.7769701	1.1881035	-1.6919221
O	0.8717749	2.3606969	0.3553363
O	-0.6579460	-1.8910718	-0.1073672
C	0.7218908	-1.9530374	-0.3342603
C	1.1344840	-2.0959241	-1.6565144
C	1.6274329	-1.9130259	0.7254833
C	2.4918378	-2.2064972	-1.9290530
H	0.3971924	-2.1023046	-2.4562968
C	2.9870268	-2.0168316	0.4513405
H	1.2764713	-1.8122694	1.7523321
C	3.3980560	-2.1661787	-0.8705641
H	2.8628116	-2.3150304	-2.9457277
H	3.7309994	-1.9913133	1.2442555
C	2.1535662	1.8523063	0.2541316
C	2.7286800	1.5340721	-0.9815565
C	2.8865361	1.7524742	1.4414609
C	4.0653697	1.1509893	-1.0276100
H	2.1253738	1.5950169	-1.8845248
C	4.2201624	1.3627847	1.3955949
H	2.4015103	2.0040271	2.3836749
C	4.7976431	1.0800477	0.1578994
H	4.5579245	0.9226761	-1.9711029
H	4.8264836	1.2891838	2.2962288
N	6.2312889	0.7340286	0.0994460
O	6.8399000	0.6369690	1.1694970
O	6.7344735	0.5782104	-1.0165395
N	4.8409139	-2.2965566	-1.1597538
O	5.1788598	-2.3869866	-2.3425982
O	5.6128604	-2.3099635	-0.1974481

Dioxophosphorane-b

P	9.5569429	-2.8113442	2.2106768
O	9.9722815	-3.5802413	3.3892405
O	8.5600449	-1.6317401	2.6397525
O	9.8533036	-2.9078941	0.7721870
C	7.9338455	-0.7131506	1.7823158
C	7.7944323	0.5769809	2.2945228
C	7.4221191	-1.0797094	0.5378951
C	7.1208040	1.5324415	1.5410112
H	8.2067903	0.8143633	3.2741572
C	6.7508816	-0.1201574	-0.2145255
H	7.5593202	-2.0885528	0.1548053
C	6.6080949	1.1680243	0.2969973
H	6.9852244	2.5504333	1.9001502
H	6.3339415	-0.3576481	-1.1910277
N	5.8839620	2.1803822	-0.5044300
O	5.7604822	3.3073334	-0.0181548
O	5.4500182	1.8304457	-1.6045104

7c

P	9.7769104	-2.6735160	2.0990141
C	10.9803213	-0.4154789	0.9320491
H	10.4033962	-0.7968554	0.0907586
C	12.4630124	1.1127311	2.0369755
C	11.7431419	0.7445828	0.9002332
H	11.7695627	1.3437479	-0.0081667
C	11.6164267	-0.8336798	3.1562939
H	11.5018772	-1.5275433	3.9895358
C	12.4021688	0.3095729	3.1787152
H	12.9583280	0.5619209	4.0799891
O	10.2762040	-3.4439277	3.2581050
O	8.4737528	-1.8020421	2.6043589
N	10.9288785	-1.1710502	2.0472792
H	13.0725535	2.0167801	2.0330816
O	9.6959177	-3.0464776	0.6654991
C	7.8849012	-0.8454459	1.7877845
C	7.8501139	0.4680853	2.2649142
C	7.3253604	-1.1740141	0.5496512
C	7.2545757	1.4660135	1.4914282
H	8.2817441	0.6870502	3.2421209
C	6.7368413	-0.1650735	-0.2147572
H	7.3792920	-2.2019483	0.1966010
C	6.7005283	1.1538246	0.2476242
H	7.2213956	2.4902479	1.8656018
H	6.2979272	-0.4163492	-1.1813488
H	6.2341334	1.9331800	-0.3556140

8c

O	-1.4469732	3.4498989	0.0866163
O	1.4183873	-0.0539085	2.5479014
P	0.4106955	0.8732271	3.0872053
P	-1.4222818	2.5223164	1.2301119
C	-3.1380951	1.2151982	-0.4668578
C	-2.7596674	0.1174220	1.5997694
C	-3.9930424	0.2073989	-0.8787812
H	-2.8838780	2.0937530	-1.0574961
C	-3.6089893	-0.9130187	1.2221891
H	-2.1943464	0.1501834	2.5507838
C	-4.2326898	-0.8724796	-0.0246969
H	-4.4590954	0.2717074	-1.8602188
H	-3.7652096	-1.7415427	1.9106415
O	-0.1232084	1.8149678	1.6250216
O	-0.8732392	0.5015143	3.7635982
N	-2.5413797	1.1504262	0.7475807
H	-4.8993437	-1.6779409	-0.3339523
O	-2.1127897	2.9654497	2.5905678
O	1.1485960	2.1193199	3.8350182
C	-3.4353274	3.3936058	2.7303135
C	-3.9934415	4.3541305	1.8897487
C	-4.1438152	2.8305157	3.7893493

C	-5.3138623	4.7485682	2.1214620
H	-3.3988423	4.7830066	1.0843228
C	-5.4585073	3.2419614	4.0089276
H	-3.6470891	2.0981933	4.4249097
C	-6.0466929	4.1977433	3.1753457
H	-5.7667688	5.5020216	1.4760950
H	-6.0235859	2.8151403	4.8382258
H	-7.0732692	4.5190239	3.3527691
C	0.4112012	3.2164504	4.2709533
C	0.5883835	4.4286656	3.6031041
C	-0.4751588	3.1059642	5.3429934
C	-0.1581062	5.5408891	3.9972462
H	1.2938940	4.4795728	2.7740361
C	-1.2151018	4.2245339	5.7266626
H	-0.5971611	2.1417631	5.8329070
C	-1.0675426	5.4401913	5.0524535
H	-0.0306353	6.4883276	3.4719369
H	-1.9173596	4.1438545	6.5576069
H	-1.6556562	6.3076714	5.3533422

Dioxophosphorane-c

P	9.6406689	-2.8221703	2.1344185
O	10.0594996	-3.6643338	3.2628503
O	8.5394041	-1.7624431	2.6002517
O	10.0181337	-2.7811110	0.7116700
C	7.9339966	-0.8040618	1.7534154
C	7.9051292	0.5022044	2.2308121
C	7.3372817	-1.1693520	0.5512754
C	7.2551178	1.4769779	1.4718822
H	8.3797392	0.7394772	3.1823625
C	6.6932326	-0.1815515	-0.1963616
H	7.3871425	-2.1979918	0.1974897
C	6.6501030	1.1380326	0.2594384
H	7.2234865	2.5047017	1.8341686
H	6.2225297	-0.4509106	-1.1420688
H	6.1441331	1.9021391	-0.3306211

7d^{-}

P	9.4868142	-1.3184448	2.0511782
C	12.0393473	-0.0370289	1.1462287
H	11.3560982	0.1377767	0.3116709
C	14.1299789	0.1026225	2.3220662
C	13.3739832	0.3623623	1.1752738
H	13.8095016	0.8742513	0.3158573
C	12.1828062	-0.9173123	3.2926153
H	11.6066775	-1.4092974	4.0802555
C	13.5229282	-0.5494692	3.3989319
H	14.0773489	-0.7649615	4.3134641
O	9.2098816	-1.2408023	3.5366387

O	9.0279844	-0.1980773	1.1437709
N	11.4744253	-0.6637203	2.1855260
H	15.1757918	0.4107823	2.3782480
O	9.8348766	-2.6575855	1.4406362

8d^{2-}

O	-0.4558140	2.3432939	-0.7575185
O	0.2320736	-0.6548057	3.1233419
P	-0.3584625	0.7219961	3.3403385
P	-1.0539707	2.2936917	0.6281734
C	-2.8172420	0.7509524	-0.9947851
C	-3.3197688	0.6770207	1.3091036
C	-3.9221411	-0.0265178	-1.3116537
H	-2.0924879	1.1420781	-1.7129621
C	-4.4257086	-0.1307997	1.0424048
H	-2.9198083	0.9036552	2.3364657
C	-4.7553227	-0.4739939	-0.2706720
H	-4.1225125	-0.2841893	-2.3532401
H	-5.0202230	-0.4905098	1.8843351
O	-0.2656384	1.3634917	1.5905072
O	-1.8776175	0.7448270	3.6256805
N	-2.5466026	1.1054486	0.2767760
H	-5.6357855	-1.0851091	-0.4872366
O	-1.7869634	3.4691400	1.2006435
O	0.4442184	1.7516015	4.1071517

Dioxophosphorane-d

P	9.5138036	-2.5623093	2.0400107
O	10.1910460	-2.8405711	3.3545010
O	8.7055602	-1.3055738	1.8616268
O	9.6451443	-3.5407770	0.9044281

7e

P	9.5780831	-1.1841069	2.1029108
C	9.9968961	-3.9450579	2.1969498
H	9.9564020	-3.8143872	3.2789342
C	12.0233751	-0.0355115	1.1372454
H	11.3464152	0.1544853	0.3040300
C	9.8223386	-2.8249056	1.3715235
C	9.8299085	-2.9804231	-0.0221810
H	9.6595972	-2.1063284	-0.6518771
C	14.1064183	0.0925493	2.3197573
C	10.0221909	-4.2431007	-0.5842845
H	10.0203142	-4.3635618	-1.6687499
C	13.3547175	0.3599547	1.1737477

H	13.7886502	0.8754757	0.3183856
C	12.1679956	-0.9248822	3.3001290
H	11.5984423	-1.4062796	4.0957758
C	10.2045613	-5.3555048	0.2427347
C	13.5032680	-0.5541595	3.4002228
H	14.0550951	-0.7649180	4.3150011
C	10.1884436	-5.2068860	1.6327161
H	10.3157936	-6.0784651	2.2765175
O	9.2757930	-1.2774294	3.5585752
O	9.0848519	-0.2083936	1.0914927
H	10.3495870	-6.3432694	-0.1975446
N	11.4659485	-0.6717809	2.1820987
H	15.1524650	0.3951921	2.3741373

8e

O	-0.3377064	2.0119769	-0.7321779
O	0.4504511	-0.2427268	3.7990106
P	-0.4128496	0.9166647	3.4804809
P	-0.9794992	1.8785921	0.5949249
C	-3.0160261	0.9709637	-1.0153526
C	-3.2070902	0.2888203	1.2427298
C	-4.1598386	0.2690281	-1.3574017
H	-2.4000540	1.5300759	-1.7192223
C	-4.3588670	-0.4269412	0.9427764
H	-2.7652844	0.3574025	2.2558497
C	-4.8405714	-0.4422014	-0.3657566
H	-4.5024189	0.2752654	-2.3903383
H	-4.8600164	-0.9723241	1.7405092
O	-0.3499342	0.9405358	1.6453134
C	-1.5460964	3.4356229	1.2830545
C	-0.7533418	4.5541104	0.9810033
C	-2.6706460	3.5721813	2.1085112
C	-1.0787413	5.7982907	1.5159280
H	0.1150815	4.4376270	0.3331753
C	-3.0033064	4.8266921	2.6161166
H	-3.2611905	2.7046848	2.3943086
C	-2.2071923	5.9366394	2.3261461
H	-0.4494595	6.6609401	1.2972970
H	-3.8785933	4.9329536	3.2576163
H	-2.4638633	6.9127727	2.7397082
C	0.4016322	2.5100462	3.7576813
C	1.6926664	2.7360109	3.2586039
C	-0.2579111	3.5265246	4.4582348
C	2.3037411	3.9760435	3.4379423
H	2.2095773	1.9367714	2.7261555
C	0.3625226	4.7614913	4.6509173
H	-1.2620514	3.3390912	4.8375698
C	1.6392863	4.9904970	4.1342917
H	3.3046488	4.1515324	3.0404960
H	-0.1551285	5.5501686	5.1988657
H	2.1213444	5.9590150	4.2776403
O	-1.8883109	1.0223237	3.7585701
N	-2.5679047	0.9688209	0.2611701

H	-5.7381121	-1.0086175	-0.6159638

Dioxophosphorane-e

P	9.4369440	-1.2455676	2.0870378
C	9.9503793	-3.9590823	2.2054976
H	9.9187566	-3.8256286	3.2863573
C	9.7457675	-2.8503701	1.3675795
C	9.7803648	-2.9862165	-0.0301912
H	9.6190521	-2.1095674	-0.6567540
C	10.0192848	-4.2406853	-0.5865569
H	10.0469696	-4.3576186	-1.6701549
C	10.2232282	-5.3470625	0.2446236
C	10.1893646	-5.2077328	1.6359407
H	10.3496667	-6.0748631	2.2768866
O	9.4181072	-1.2338493	3.5638529
O	9.2451871	-0.1667807	1.0965967
H	10.4099784	-6.3272249	-0.1962218

7f

P	9.6053452	-1.1749848	2.1049860
C	9.9998387	-3.9448932	2.1954975
H	9.9600808	-3.8130924	3.2775346
C	12.0245320	-0.0363322	1.1429005
H	11.3574035	0.1611436	0.3038966
C	9.8296599	-2.8231297	1.3718762
C	9.8332485	-2.9811263	-0.0212272
H	9.6638068	-2.1062136	-0.6502859
C	14.1369910	0.1000775	2.3208226
C	10.0214588	-4.2443642	-0.5841918
H	10.0165228	-4.3646841	-1.6688766
C	13.3435078	0.3591941	1.1701533
H	13.7494899	0.8782593	0.3061087
C	12.1684256	-0.9211251	3.2955969
H	11.6097035	-1.4007745	4.0994957
C	10.2037116	-5.3574111	0.2418943
C	13.4921397	-0.5569548	3.4035705
H	14.0174435	-0.7727262	4.3300458
C	10.1875735	-5.2078274	1.6317180
H	10.3123689	-6.0799258	2.2756270
O	9.2814126	-1.2789696	3.5573006
O	9.0908199	-0.2100564	1.0909955
H	10.3472028	-6.3455711	-0.1983543
N	11.4476887	-0.6753636	2.1818765
N	15.4438111	0.4713592	2.3869509
C	16.2185102	0.2139536	3.5944175
H	16.2559784	-0.8621151	3.8253688
H	17.2433485	0.5650171	3.4413866
H	15.7993087	0.7429580	4.4654156
C	16.0669894	1.1651188	1.2669398

H	15.5763215	2.1307214	1.0637633
H	17.1164285	1.3592178	1.5077991
H	16.0331644	0.5570439	0.3493598

8f

O	0.2200788	1.5034585	-2.6827343
O	-0.4182490	-2.0394233	-0.2781423
P	0.7672787	-1.3682404	0.3336829
P	0.4009679	1.1942244	-1.2466058
C	-2.1266238	0.2378322	-1.4688195
C	-1.5498981	0.8413957	0.7274865
C	-3.2885726	-0.3463998	-1.0433098
H	-1.8141970	0.2573719	-2.5115227
C	-2.6967336	0.2744661	1.2184821
H	-0.8135200	1.3010323	1.3788770
C	-3.5964679	-0.3896657	0.3443014
H	-3.9298289	-0.8145021	-1.7838393
H	-2.8589293	0.3024687	2.2915497
O	1.2087637	-0.0680546	-0.8335359
C	0.9443055	2.5646656	-0.2277554
C	1.5766009	2.3444266	1.0059892
C	0.7389731	3.8696777	-0.7011964
C	1.9887152	3.4396296	1.7662273
H	1.7151724	1.3215282	1.3665901
C	1.1605458	4.9557363	0.0642034
H	0.2688493	4.0170987	-1.6739907
C	1.7825924	4.7401640	1.2978607
H	2.4786157	3.2755799	2.7267950
H	1.0105866	5.9713683	-0.3036227
H	2.1142161	5.5916266	1.8942446
C	2.2800023	-2.3355447	0.1372219
C	2.3796185	-3.2786743	-0.8943130
C	3.3466404	-2.1649433	1.0288975
C	3.5421322	-4.0361122	-1.0384962
H	1.5347808	-3.4135983	-1.5705290
C	4.5070840	-2.9271137	0.8858544
H	3.2468138	-1.4403897	1.8376814
C	4.6066690	-3.8603145	-0.1494824
H	3.6184978	-4.7692868	-1.8430422
H	5.3349820	-2.7960020	1.5846409
H	5.5146744	-4.4552105	-0.2617901
O	0.7400674	-0.6524054	1.6541613
N	-1.2590225	0.8236050	-0.6008762
N	-4.6895824	-1.0405577	0.8105402
C	-4.9109876	-1.1609355	2.2482174
H	-5.0467165	-0.1733063	2.7151061
H	-5.8189740	-1.7465206	2.4196817
H	-4.0688680	-1.6713010	2.7408842
C	-5.5647205	-1.7585880	-0.1098816
H	-5.9711973	-1.0828045	-0.8773788
H	-5.0346718	-2.5843175	-0.6104958
H	-6.4052736	-2.1761740	0.4521731

5. References

[1] T. Schneider, K. Schwedtmann, J. Fidelius, J. J. Weigand, *Nat. Synth.* **2023**, *2*, 972–979.

[2] H. J. Jessen, T. Dürr-Mayer, T. M. Haas, A. Ripp, C. C. Cummins, *Acc. Chem. Res.* **2021**, *54*, 4036–4050.

[3] M. I. Arz, V. T. Annibale, N. L. Kelly, J. V. Hanna, I. Manners, *J. Am. Chem. Soc.* **2019**, *141*, 2894–2899.

[4] K. Moedritzer, L. Maier, L. C. D. Groenweghe, *J. Chem. Eng. Data* **1962**, *7*, 307–310.

[5] M. S. Markoulides, A. C. Regan, *Org. Biomol. Chem.* **2013**, *11*, 119–129.

[6] G. Aksnes, P. Majewski, *Phosphorous Sulfur Relat. Elem.* **1986**, *26*, 261–274.

[7] S. K. Davidowski, C. E. Lisowski, J. L. Yarger, *Magn. Reson. Chem.* **2016**, *54*, 234–238.

[8] L. Botez, G. B. de Jong, J. C. Slootweg, B. Deelman, *European J. Org. Chem.* **2017**, *2017*, 434–437.

[9] D. A. Watson, M. Chiu, R. G. Bergman, *Organometallics* **2006**, *25*, 4731–4733.

[10] Y. S. Vygodskii, T. V. Volkova, A. A. Sakharova, D. A. Sapozhnikov, G. G. Nikiforova, A. M. Matieva, *Polym. Sci. Ser. A* **2006**, *48*, 683–688.

[11] Oxford Diffraction / Agilent Technologies UK Ltd, **2016**.

[12] O. V. Dolomanov, L. J. Bourhis, R. J. Gildea, J. A. K. Howard, H. Puschmann, *J. Appl. Crystallogr.* **2009**, *42*, 339–341.

[13] G. M. Sheldrick, *Acta Crystallogr. Sect. A Found. Crystallogr.* **2015**, *71*, 3–8.

[14] G. M. Sheldrick, *Acta Crystallogr. Sect. C, Struct. Chem.* **2015**, *71*, 3–8.

[15] A. L. Spek, *J. Appl. Crystallogr.* **2003**, *36*, 7–13.

[16] R. Ahlrichs, M. Bär, M. Häser, H. Horn, C. Kölmel, *Chem. Phys. Lett.* **1989**, *162*, 165–169.

[17] J. P. Perdew, *Phys. Rev. B* **1986**, *33*, 8822–8824.

[18] A. D. Becke, *J. Chem. Phys.* **1996**, *104*, 1040–1046.

[19] E. Caldeweyher, C. Bannwarth, S. Grimme, *J. Chem. Phys.* **2017**, *147*, DOI 10.1063/1.4993215.

[20] F. Weigend, R. Ahlrichs, *Phys. Chem. Chem. Phys.* **2005**, *7*, 3297.

[21] A. Klamt, *WIREs Comput. Mol. Sci.* **2011**, *1*, 699–709.
