# Supplementary material for: Synthesis of Diaryl‐ and Dialkynylphosphinates From Ubiquitous PV Sources via a Redox‐Neutral Approach
Source: Adv Sci (Weinh). 2025 Jul 2;12(36):e09922. doi: 10.1002/advs.202509922 (PMC12463002; doi:10.1002/advs.202509922)

checkCIF/PLATON report

Structure factors have been supplied for datablock(s) jjw1848

THIS REPORT IS FOR GUIDANCE ONLY. IF USED AS PART OF A REVIEW PROCEDURE FOR PUBLICATION, IT SHOULD NOT REPLACE THE EXPERTISE OF AN EXPERIENCED CRYSTALLOGRAPHIC REFEREE.

No syntax errors found. CIF dictionary Interpreting this report

Datablock: jjw1848

Bond precision: C-C = 0.0027 A Wavelength=1.54184

Cell:	a=6.0955(2)	b=7.4859(3)	c=15.7245(5)
	alpha=81.063(3)	beta=79.306(3)	gamma=86.907(3)
Temperature:	100 K		

	Calculated	Reported
Volume	696.26(4)	696.26(4)
Space group	P -1	P -1
Hall group	-P 1	-P 1
Moiety formula	C16 H19 O2 P	C16 H19 O2 P
Sum formula	C16 H19 O2 P	C16 H19 O2 P
Mr	274.28	274.28
Dx,g cm-3	1.308	1.308
Z	2	2
Mu (mm-1)	1.706	1.706
F000	292.0	292.0
F000'	293.34	
h,k,lmax	7,9,19	7,9,19
Nref	2941	2882
Tmin,Tmax	0.779,0.842	0.794,0.884
Tmin'	0.691	

Correction method= # Reported T Limits: Tmin=0.794 Tmax=0.884
AbsCorr = ANALYTICAL

Data completeness= 0.980 Theta(max)= 76.663

R(reflections)= 0.0412(2687) wR2(reflections)= 0.1064(2882)

S = 1.117 Npar= 174

The following ALERTS were generated. Each ALERT has the format
test-name_ALERT_alert-type_alert-level.
Click on the hyperlinks for more details of the test.

Alert level C

```
PLAT230_ALERT_2_C Hirshfeld Test Diff for    P1       --O1       .        6.3 s.u.
PLAT906_ALERT_3_C Large K Value in the Analysis of Variance ......      2.045 Check
PLAT911_ALERT_3_C Missing FCF Refl Between Thmin & STh/L=    0.600         4 Report
                6 -2  3,  -5 -4  7,  -3  4  9,  -5  1 10,
```

Alert level G

```
PLAT007_ALERT_5_G Number of Unrefined Donor-H Atoms .............          2 Report
              H1    H2
PLAT154_ALERT_1_G The s.u.'s on the Cell Angles are Equal ..(Note)      0.003 Degree
PLAT299_ALERT_4_G Atom Site Occupancy Constrained at ............        0.5 Check
              H1      H2
PLAT789_ALERT_4_G Atoms with Negative _atom_site_disorder_group  #          2 Check
PLAT822_ALERT_4_G CIF-embedded .res Contains Negative PART Numbers          2 Check
PLAT912_ALERT_4_G Missing # of FCF Reflections Above STh/L=  0.600         55 Note
PLAT933_ALERT_2_G Number of HKL-OMIT Records in Embedded .res File          1 Note
               -5  1 10,
PLAT941_ALERT_3_G Average HKL Measurement Multiplicity ..........        2.3 Low
PLAT969_ALERT_5_G The 'Henn et al.' R-Factor-gap value ..........      3.646 Note
              Predicted wR2: Based on SigI**2  2.92 or SHELX Weight  9.53
PLAT978_ALERT_2_G Number C-C Bonds with Positive Residual Density.         15 Info
```

```
   0 ALERT level A = Most likely a serious problem - resolve or explain
   0 ALERT level B = A potentially serious problem, consider carefully
   3 ALERT level C = Check. Ensure it is not caused by an omission or oversight
  10 ALERT level G = General information/check it is not something unexpected

   1 ALERT type 1 CIF construction/syntax error, inconsistent or missing data
   3 ALERT type 2 Indicator that the structure model may be wrong or deficient
   3 ALERT type 3 Indicator that the structure quality may be low
   4 ALERT type 4 Improvement, methodology, query or suggestion
   2 ALERT type 5 Informative message, check
```

Validation response form

Please find below a validation response form (VRF) that can be filled in and pasted into your CIF.

```
# start Validation Reply Form
_vrf_PLAT230_jjw1848
;
PROBLEM: Hirshfeld Test Diff for    P1       --O1       .        6.3 s.u.
RESPONSE: ...
;
_vrf_PLAT906_jjw1848
;
PROBLEM: Large K Value in the Analysis of Variance ......      2.045 Check
RESPONSE: ...
```

```
;
_vrf_PLAT911_jjw1848
;
PROBLEM: Missing FCF Refl Between Thmin & STh/L=    0.600          4 Report
RESPONSE: ...
;
# end Validation Reply Form
```

It is advisable to attempt to resolve as many as possible of the alerts in all categories. Often the minor alerts point to easily fixed oversights, errors and omissions in your CIF or refinement strategy, so attention to these fine details can be worthwhile. In order to resolve some of the more serious problems it may be necessary to carry out additional measurements or structure refinements. However, the purpose of your study may justify the reported deviations and the more serious of these should normally be commented upon in the discussion or experimental section of a paper or in the "special_details" fields of the CIF. checkCIF was carefully designed to identify outliers and unusual parameters, but every test has its limitations and alerts that are not important in a particular case may appear. Conversely, the absence of alerts does not guarantee there are no aspects of the results needing attention. It is up to the individual to critically assess their own results and, if necessary, seek expert advice.

Publication of your CIF in IUCr journals

A basic structural check has been run on your CIF. These basic checks will be run on all CIFs submitted for publication in IUCr journals (*Acta Crystallographica*, *Journal of Applied Crystallography*, *Journal of Synchrotron Radiation*); however, if you intend to submit to *Acta Crystallographica Section C* or *E* or *IUCrData*, you should make sure that full publication checks are run on the final version of your CIF prior to submission.

Publication of your CIF in other journals

Please refer to the *Notes for Authors* of the relevant journal for any special instructions relating to CIF submission.

PLATON version of 02/02/2025; check.def file version of 02/02/2025

Datablock jjw1848 - ellipsoid plot

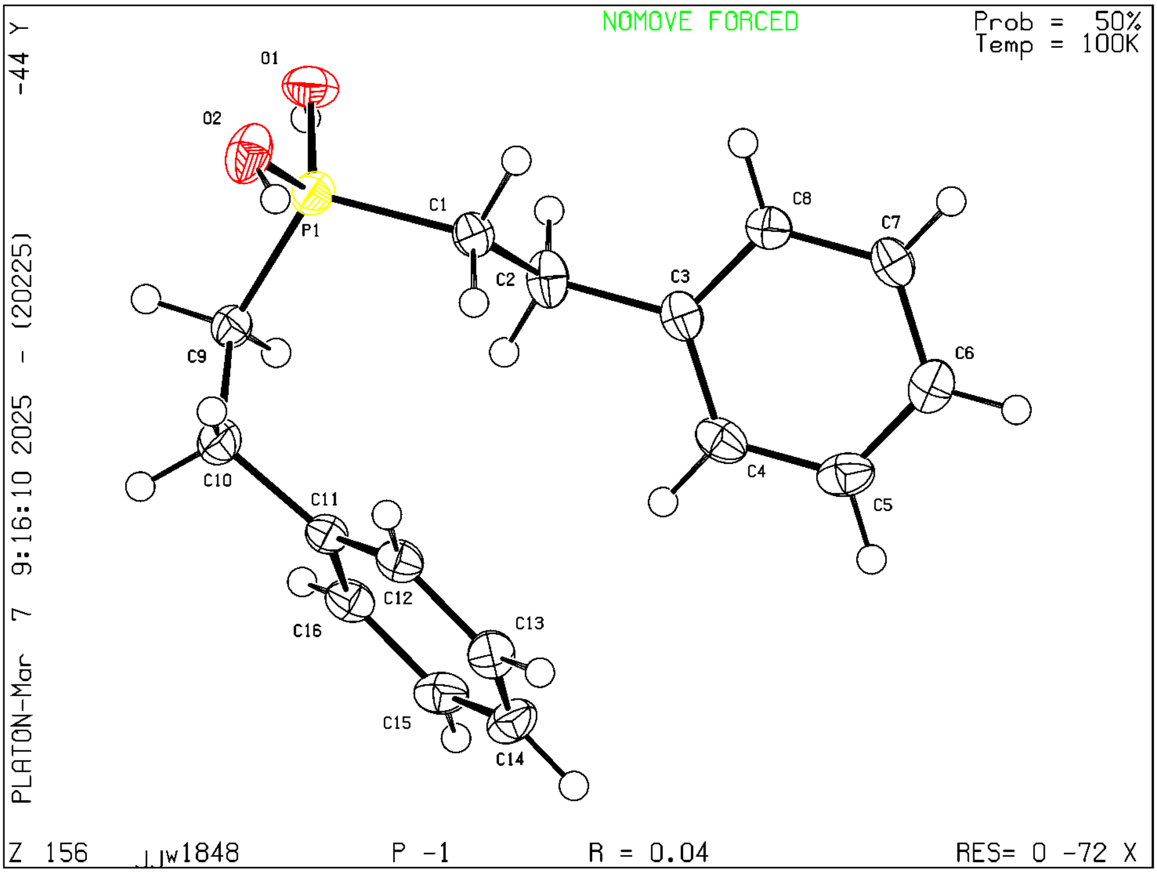

Supplement: Supplementary file 2 — Supporting Information [file ADVS-12-e09922-s001.zip › 10a_jjw1848.pdf]
