# Supplementary material for: Synthesis of Diaryl‐ and Dialkynylphosphinates From Ubiquitous PV Sources via a Redox‐Neutral Approach
Source: Adv Sci (Weinh). 2025 Jul 2;12(36):e09922. doi: 10.1002/advs.202509922 (PMC12463002; doi:10.1002/advs.202509922)

checkCIF/PLATON report

Structure factors have been supplied for datablock(s) jjw1773

THIS REPORT IS FOR GUIDANCE ONLY. IF USED AS PART OF A REVIEW PROCEDURE FOR PUBLICATION, IT SHOULD NOT REPLACE THE EXPERTISE OF AN EXPERIENCED CRYSTALLOGRAPHIC REFEREE.

No syntax errors found.   CIF dictionary   Interpreting this report

Datablock: jjw1773

Bond precision:	C-C = 0.0030 A		Wavelength=0.71073
Cell:	a=16.0174(3)	b=6.8698(1)	c=16.2295(3)
	alpha=90	beta=91.720(2)	gamma=90
Temperature:	100 K		

	Calculated	Reported
Volume	1785.03(5)	1785.03(5)
Space group	P 21/n	P 1 21/n 1
Hall group	-P 2yn	-P 2yn
Moiety formula	C16 H7 F12 O2 P	C16 H7 F12 O2 P
Sum formula	C16 H7 F12 O2 P	C16 H7 F12 O2 P
Mr	490.19	490.19
Dx,g cm-3	1.824	1.824
Z	4	4
Mu (mm-1)	0.287	0.287
F000	968.0	968.0
F000'	969.43	
h,k,lmax	24,10,24	23,10,24
Nref	6692	6030
Tmin,Tmax	0.959,0.975	0.953,0.979
Tmin'	0.933	

Correction method= # Reported T Limits: Tmin=0.953 Tmax=0.979
AbsCorr = ANALYTICAL

Data completeness= 0.901   Theta(max)= 32.910

R(reflections)= 0.0592(4721)   wR2(reflections)= 0.1639(6030)

S = 1.032   Npar= 365

The following ALERTS were generated. Each ALERT has the format
test-name_ALERT_alert-type_alert-level.
Click on the hyperlinks for more details of the test.

Alert level C

```
PLAT213_ALERT_2_C Atom F1              has ADP max/min Ratio .....         3.2 prolat
PLAT213_ALERT_2_C Atom F3              has ADP max/min Ratio .....         3.1 prolat
PLAT213_ALERT_2_C Atom F11B            has ADP max/min Ratio .....         3.3 prolat
PLAT906_ALERT_3_C Large K Value in the Analysis of Variance ......       4.014 Check
```

Alert level G

```
PLAT002_ALERT_2_G Number of Distance or Angle Restraints on AtSite          14 Note
PLAT003_ALERT_2_G Number of Uiso or U(i,j) Restrained non-H Atoms          25 Report
PLAT007_ALERT_5_G Number of Unrefined Donor-H Atoms .............           1 Report
              H1
PLAT066_ALERT_1_G Predicted and Reported Tmin&Tmax Range Identical          ? Check
PLAT176_ALERT_4_G The CIF-Embedded .res File Contains SADI Records          6 Report
PLAT178_ALERT_4_G The CIF-Embedded .res File Contains SIMU Records          4 Report
PLAT187_ALERT_4_G The CIF-Embedded .res File Contains RIGU Records          3 Report
PLAT188_ALERT_3_G A Non-default SIMU Restraint Value has been used     0.0100 Report
PLAT188_ALERT_3_G A Non-default SIMU Restraint Value has been used     0.0100 Report
PLAT188_ALERT_3_G A Non-default SIMU Restraint Value has been used     0.0100 Report
PLAT230_ALERT_2_G Hirshfeld Test Diff for    F9A      --C7       .         8.5 s.u.
PLAT230_ALERT_2_G Hirshfeld Test Diff for    F9B      --C7       .        14.0 s.u.
PLAT242_ALERT_2_G Low    'MainMol' Ueq as Compared to Neighbors of          C4 Check
PLAT242_ALERT_2_G Low    'MainMol' Ueq as Compared to Neighbors of          C7 Check
PLAT242_ALERT_2_G Low    'MainMol' Ueq as Compared to Neighbors of         C12 Check
PLAT242_ALERT_2_G Low    'MainMol' Ueq as Compared to Neighbors of         C16 Check
PLAT301_ALERT_3_G Main Residue  Disorder .............(Resd    1)          29% Note
PLAT434_ALERT_2_G Short Inter HL..HL Contact F2        ..F5B      .       2.66 Ang.
                                                   x,-1+y,z  =      1_545 Check
PLAT811_ALERT_5_G No ADDSYM Analysis: Too Many Excluded Atoms ....           ! Info
PLAT860_ALERT_3_G Number of Least-Squares Restraints ............         366 Note
PLAT910_ALERT_3_G Missing # of FCF Reflection(s) Below Theta(Min).           2 Note
               -1  0  1,   1  0  1,
PLAT912_ALERT_4_G Missing # of FCF Reflections Above STh/L=  0.600         643 Note
PLAT941_ALERT_3_G Average HKL Measurement Multiplicity ..........         3.2 Low
PLAT969_ALERT_5_G The 'Henn et al.' R-Factor-gap value ..........       6.260 Note
              Predicted wR2: Based on SigI**2  2.62 or SHELX Weight 15.88
PLAT978_ALERT_2_G Number C-C Bonds with Positive Residual Density.           9 Info
```

   0 **ALERT level A** = Most likely a serious problem - resolve or explain
   0 **ALERT level B** = A potentially serious problem, consider carefully
   4 **ALERT level C** = Check. Ensure it is not caused by an omission or oversight
  25 **ALERT level G** = General information/check it is not something unexpected

   1 ALERT type 1 CIF construction/syntax error, inconsistent or missing data
  13 ALERT type 2 Indicator that the structure model may be wrong or deficient
   8 ALERT type 3 Indicator that the structure quality may be low
   4 ALERT type 4 Improvement, methodology, query or suggestion
   3 ALERT type 5 Informative message, check

It is advisable to attempt to resolve as many as possible of the alerts in all categories. Often the minor alerts point to easily fixed oversights, errors and omissions in your CIF or refinement strategy, so attention to these fine details can be worthwhile. In order to resolve some of the more serious problems it may be necessary to carry out additional measurements or structure refinements. However, the purpose of your study may justify the reported deviations and the more serious of these should normally be commented upon in the discussion or experimental section of a paper or in the "special_details" fields of the CIF. checkCIF was carefully designed to identify outliers and unusual parameters, but every test has its limitations and alerts that are not important in a particular case may appear. Conversely, the absence of alerts does not guarantee there are no aspects of the results needing attention. It is up to the individual to critically assess their own results and, if necessary, seek expert advice.

Publication of your CIF in IUCr journals

A basic structural check has been run on your CIF. These basic checks will be run on all CIFs submitted for publication in IUCr journals (*Acta Crystallographica*, *Journal of Applied Crystallography*, *Journal of Synchrotron Radiation*); however, if you intend to submit to *Acta Crystallographica Section C* or *E* or *IUCrData*, you should make sure that full publication checks are run on the final version of your CIF prior to submission.

Publication of your CIF in other journals

Please refer to the *Notes for Authors* of the relevant journal for any special instructions relating to CIF submission.

PLATON version of 15/07/2024; check.def file version of 15/07/2024

Datablock jjw1773 - ellipsoid plot

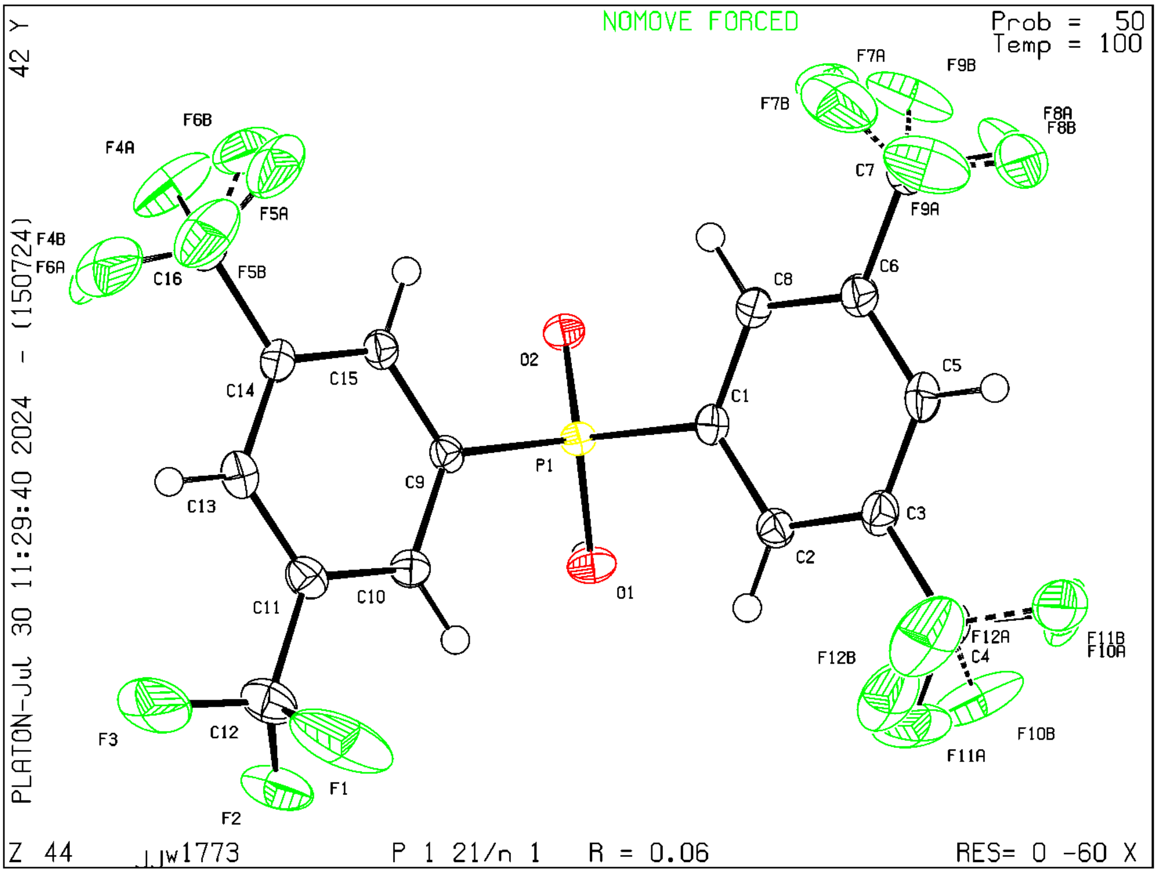

Supplement: Supplementary file 2 — Supporting Information [file ADVS-12-e09922-s001.zip › 2g_jjw1773.pdf]
