# Supplementary material for: Synthesis of Diaryl‐ and Dialkynylphosphinates From Ubiquitous PV Sources via a Redox‐Neutral Approach
Source: Adv Sci (Weinh). 2025 Jul 2;12(36):e09922. doi: 10.1002/advs.202509922 (PMC12463002; doi:10.1002/advs.202509922)

checkCIF/PLATON report

Structure factors have been supplied for datablock(s) jjw1788

THIS REPORT IS FOR GUIDANCE ONLY. IF USED AS PART OF A REVIEW PROCEDURE FOR PUBLICATION, IT SHOULD NOT REPLACE THE EXPERTISE OF AN EXPERIENCED CRYSTALLOGRAPHIC REFEREE.

No syntax errors found. CIF dictionary Interpreting this report

Datablock: jjw1788

Bond precision: C-C = 0.0025 A Wavelength=1.54184

Cell:	a=13.4443(2)	b=7.5190(1)	c=15.7509(2)
	alpha=90	beta=102.446(1)	gamma=90
Temperature:	100 K		

	Calculated	Reported
Volume	1554.80(4)	1554.80(4)
Space group	I 2/m	I 1 2/m 1
Hall group	-I 2y	-I 2y
Moiety formula	C20 H38 O4 P2 Si4	0.5(C20 H38 O4 P2 Si4)
Sum formula	C20 H38 O4 P2 Si4	C10 H19 O2 P Si2
Mr	516.80	258.40
Dx,g cm-3	1.104	1.104
Z	2	4
Mu (mm-1)	2.917	2.917
F000	552.0	552.0
F000'	556.29	
h,k,lmax	16,9,19	16,9,19
Nref	1772	1757
Tmin,Tmax	0.463,0.839	0.286,1.000
Tmin'	0.271	

Correction method= # Reported T Limits: Tmin=0.286 Tmax=1.000
AbsCorr = GAUSSIAN

Data completeness= 0.992 Theta(max)= 76.562

R(reflections)= 0.0296(1618) wR2(reflections)= 0.0821(1757)

S = 1.051 Npar= 85

```
The following ALERTS were generated. Each ALERT has the format
       test-name_ALERT_alert-type_alert-level.
Click on the hyperlinks for more details of the test.
```

Alert level C

```
PLAT042_ALERT_1_C Calc. and Reported MoietyFormula Strings  Differ     Please Check
              Calc: C20 H38 O4 P2 Si4
              Rep.: 0.5(C20 H38 O4 P2 Si4)
```

Alert level G

```
PLAT045_ALERT_1_G Calculated and Reported Z Differ by a Factor ...      0.500 Check
PLAT230_ALERT_2_G Hirshfeld Test Diff for    P1       --C1       .        7.8 s.u.
PLAT230_ALERT_2_G Hirshfeld Test Diff for    P1       --C5       .        5.4 s.u.
PLAT230_ALERT_2_G Hirshfeld Test Diff for    Si1      --C2       .        7.2 s.u.
PLAT230_ALERT_2_G Hirshfeld Test Diff for    Si2      --C6       .        6.8 s.u.
PLAT299_ALERT_4_G Atom Site Occupancy Constrained at ............         0.5 Check
              H4A     H4C     H7A     H7C
PLAT303_ALERT_2_G Full Occupancy Atom H1        with # Connections       2.00 Check
PLAT912_ALERT_4_G Missing # of FCF Reflections Above STh/L=  0.600         15 Note
PLAT941_ALERT_3_G Average HKL Measurement Multiplicity ..........         4.3 Low
PLAT969_ALERT_5_G The 'Henn et al.' R-Factor-gap value ..........       2.395 Note
              Predicted wR2: Based on SigI**2  3.43 or SHELX Weight  7.81
PLAT978_ALERT_2_G Number C-C Bonds with Positive Residual Density.          2 Info
```

```
   0 ALERT level A = Most likely a serious problem - resolve or explain
   0 ALERT level B = A potentially serious problem, consider carefully
   1 ALERT level C = Check. Ensure it is not caused by an omission or oversight
  11 ALERT level G = General information/check it is not something unexpected

   2 ALERT type 1 CIF construction/syntax error, inconsistent or missing data
   6 ALERT type 2 Indicator that the structure model may be wrong or deficient
   1 ALERT type 3 Indicator that the structure quality may be low
   2 ALERT type 4 Improvement, methodology, query or suggestion
   1 ALERT type 5 Informative message, check
```

It is advisable to attempt to resolve as many as possible of the alerts in all categories. Often the minor alerts point to easily fixed oversights, errors and omissions in your CIF or refinement strategy, so attention to these fine details can be worthwhile. In order to resolve some of the more serious problems it may be necessary to carry out additional measurements or structure refinements. However, the purpose of your study may justify the reported deviations and the more serious of these should normally be commented upon in the discussion or experimental section of a paper or in the "special_details" fields of the CIF. checkCIF was carefully designed to identify outliers and unusual parameters, but every test has its limitations and alerts that are not important in a particular case may appear. Conversely, the absence of alerts does not guarantee there are no aspects of the results needing attention. It is up to the individual to critically assess their own results and, if necessary, seek expert advice.

Publication of your CIF in IUCr journals

A basic structural check has been run on your CIF. These basic checks will be run on all CIFs submitted for publication in IUCr journals (*Acta Crystallographica*, *Journal of Applied Crystallography*, *Journal of Synchrotron Radiation*); however, if you intend to submit to *Acta Crystallographica Section C* or *E* or *IUCrData*, you should make sure that full publication checks are run on the final version of your CIF prior to submission.

Publication of your CIF in other journals

Please refer to the *Notes for Authors* of the relevant journal for any special instructions relating to CIF submission.

PLATON version of 15/07/2024; check.def file version of 15/07/2024

Datablock jjw1788 - ellipsoid plot

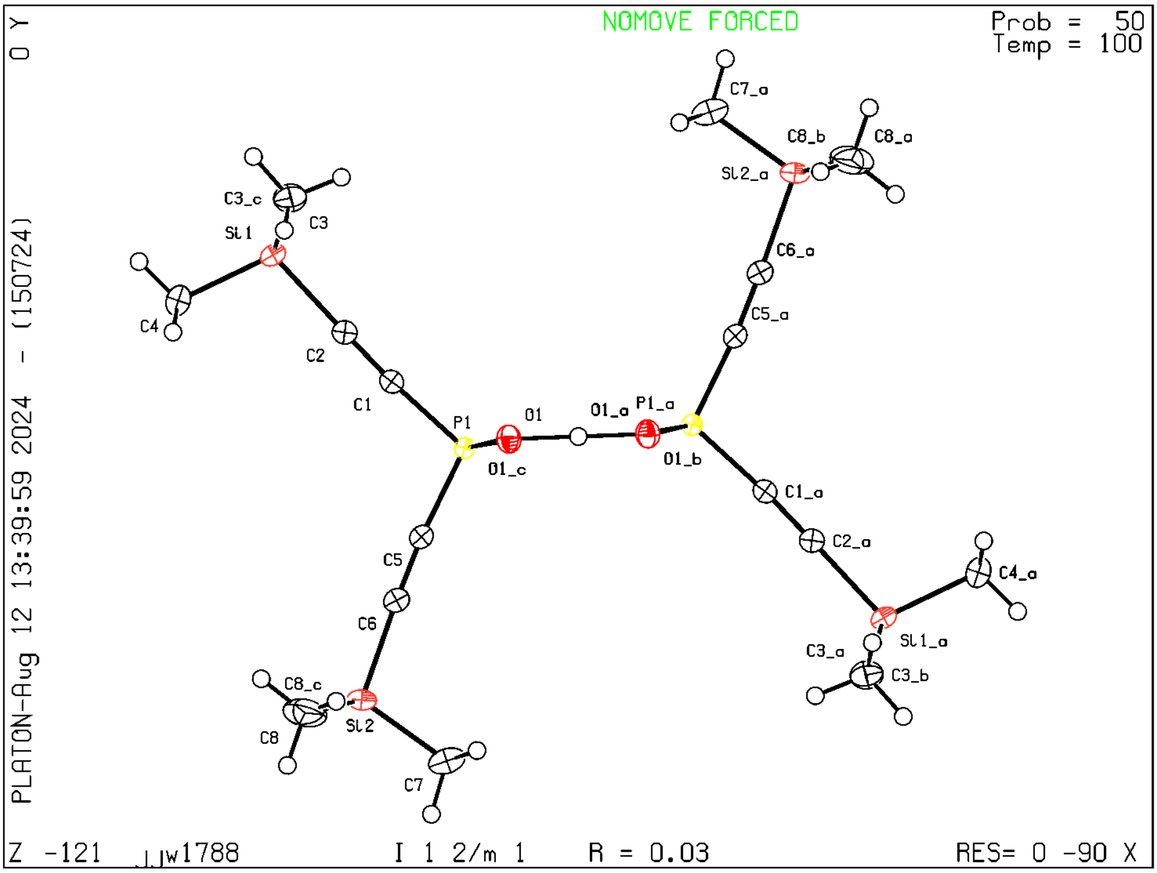

Supplement: Supplementary file 2 — Supporting Information [file ADVS-12-e09922-s001.zip › 9a_jjw1788.pdf]
