# Supplementary material for: Synthesis of Diaryl‐ and Dialkynylphosphinates From Ubiquitous PV Sources via a Redox‐Neutral Approach
Source: Adv Sci (Weinh). 2025 Jul 2;12(36):e09922. doi: 10.1002/advs.202509922 (PMC12463002; doi:10.1002/advs.202509922)

checkCIF/PLATON report

Structure factors have been supplied for datablock(s) jjw1738

THIS REPORT IS FOR GUIDANCE ONLY. IF USED AS PART OF A REVIEW PROCEDURE FOR PUBLICATION, IT SHOULD NOT REPLACE THE EXPERTISE OF AN EXPERIENCED CRYSTALLOGRAPHIC REFEREE.

No syntax errors found. CIF dictionary Interpreting this report

Datablock: jjw1738

Bond precision:	C-C = 0.0046 A	Wavelength=1.54184	
Cell:	a=22.3980(2)	b=6.0808(1)	c=27.5888(3)
	alpha=90	beta=91.007(1)	gamma=90
Temperature:	100 K		

	Calculated	Reported
Volume	3756.95(8)	3756.95(8)
Space group	I a	I 1 a 1
Hall group	I -2ya	I -2ya
Moiety formula	C18 H17 O3 P, C18 H15 O2 P, C6 H6, H2 O	0.5(C18 H17 O3 P), 0.5(C18 H15 O2 P), 0.5(H2 O), 0.5(C6 H6)
Sum formula	C42 H40 O6 P2	C21 H20 O3 P
Mr	702.68	351.34
Dx,g cm-3	1.242	1.242
Z	4	8
Mu (mm-1)	1.425	1.425
F000	1480.0	1480.0
F000'	1486.43	
h,k,lmax	28,7,34	28,7,34
Nref	7909[3956]	6849
Tmin,Tmax	0.843,0.931	0.733,0.938
Tmin'	0.590	

Correction method= # Reported T Limits: Tmin=0.733 Tmax=0.938
AbsCorr = ANALYTICAL

Data completeness= 1.73/0.87 Theta(max)= 76.518

R(reflections)= 0.0343(6483) wR2(reflections)= 0.0910(6849)

S = 1.023 Npar= 475

The following ALERTS were generated. Each ALERT has the format
test-name_ALERT_alert-type_alert-level.
Click on the hyperlinks for more details of the test.

Alert level C

```
STRVA01_ALERT_4_C           Flack test results are ambiguous.
           From the CIF: _refine_ls_abs_structure_Flack    0.500
           From the CIF: _refine_ls_abs_structure_Flack_su    0.020
PLAT042_ALERT_1_C Calc. and Reported MoietyFormula Strings  Differ     Please Check
              Calc: C18 H17 O3 P, C18 H15 O2 P, C6 H6, H2 O
              Rep.: 0.5(C18 H17 O3 P), 0.5(C18 H15 O2 P), 0.5(H2 O
                    ), 0.5(C6 H6)
PLAT313_ALERT_2_C Oxygen with Three Covalent Bonds (rare) ........          O5 Check
PLAT340_ALERT_3_C Low Bond Precision on  C-C Bonds ...............     0.0046 Ang.
```

Alert level G

```
PLAT007_ALERT_5_G Number of Unrefined Donor-H Atoms .............           2 Report
              H5A   H5B
PLAT045_ALERT_1_G Calculated and Reported Z Differ by a Factor ...      0.500 Check
PLAT111_ALERT_2_G ADDSYM Detects New (Pseudo) Centre of Symmetry .        84 %Fit
PLAT113_ALERT_2_G ADDSYM Suggests Possible Pseudo/New  Space Group       I2/a Check
              Check Model Parameter Symmetry for Reflection Data Support
PLAT303_ALERT_2_G Full Occupancy Atom H2        with # Connections       2.00 Check
PLAT371_ALERT_2_G Long   C(sp2)-C(sp1) Bond  C2       - C3       .       1.43 Ang.
PLAT371_ALERT_2_G Long   C(sp2)-C(sp1) Bond  C11      - C12      .       1.44 Ang.
PLAT371_ALERT_2_G Long   C(sp2)-C(sp1) Bond  C20      - C21      .       1.43 Ang.
PLAT371_ALERT_2_G Long   C(sp2)-C(sp1) Bond  C29      - C30      .       1.43 Ang.
PLAT912_ALERT_4_G Missing # of FCF Reflections Above STh/L=  0.600          13 Note
PLAT915_ALERT_3_G No Flack x Check Done: Low Friedel Pair Coverage          74 %
PLAT969_ALERT_5_G The 'Henn et al.' R-Factor-gap value ..........        2.82 Note
              Predicted wR2: Based on SigI**2  3.22 or SHELX Weight  9.22
PLAT978_ALERT_2_G Number C-C Bonds with Positive Residual Density.          11 Info
```

---

0 **ALERT level A** = Most likely a serious problem - resolve or explain
0 **ALERT level B** = A potentially serious problem, consider carefully
4 **ALERT level C** = Check. Ensure it is not caused by an omission or oversight
13 **ALERT level G** = General information/check it is not something unexpected

2 ALERT type 1 CIF construction/syntax error, inconsistent or missing data
9 ALERT type 2 Indicator that the structure model may be wrong or deficient
2 ALERT type 3 Indicator that the structure quality may be low
2 ALERT type 4 Improvement, methodology, query or suggestion
2 ALERT type 5 Informative message, check

It is advisable to attempt to resolve as many as possible of the alerts in all categories. Often the minor alerts point to easily fixed oversights, errors and omissions in your CIF or refinement strategy, so attention to these fine details can be worthwhile. In order to resolve some of the more serious problems it may be necessary to carry out additional measurements or structure refinements. However, the purpose of your study may justify the reported deviations and the more serious of these should normally be commented upon in the discussion or experimental section of a paper or in the "special_details" fields of the CIF. checkCIF was carefully designed to identify outliers and unusual parameters, but every test has its limitations and alerts that are not important in a particular case may appear. Conversely, the absence of alerts does not guarantee there are no aspects of the results needing attention. It is up to the individual to critically assess their own results and, if necessary, seek expert advice.

Publication of your CIF in IUCr journals

A basic structural check has been run on your CIF. These basic checks will be run on all CIFs submitted for publication in IUCr journals (*Acta Crystallographica*, *Journal of Applied Crystallography*, *Journal of Synchrotron Radiation*); however, if you intend to submit to *Acta Crystallographica Section C* or *E* or *IUCrData*, you should make sure that full publication checks are run on the final version of your CIF prior to submission.

Publication of your CIF in other journals

Please refer to the *Notes for Authors* of the relevant journal for any special instructions relating to CIF submission.

PLATON version of 06/01/2024; check.def file version of 05/01/2024

Datablock jjw1738 - ellipsoid plot

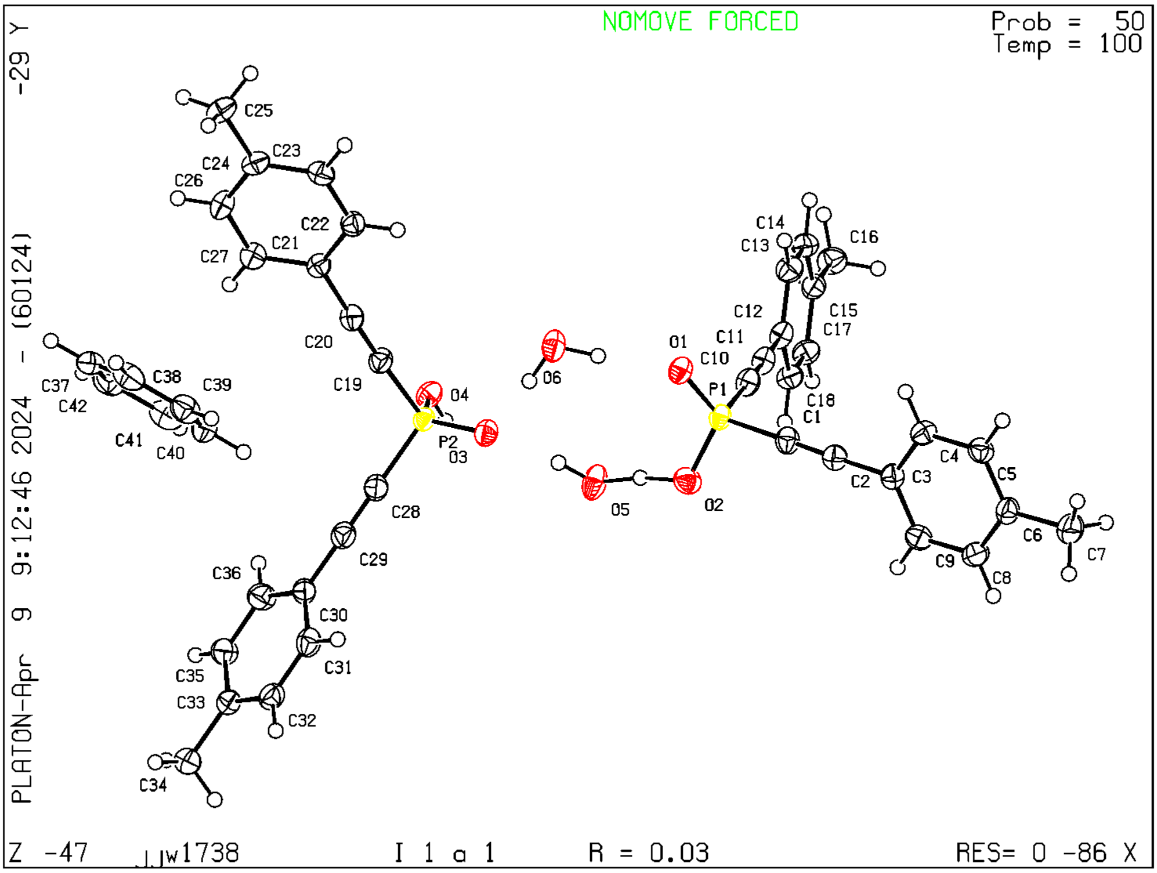

Supplement: Supplementary file 2 — Supporting Information [file ADVS-12-e09922-s001.zip › 9c_H2O_05C6H6_jjw1738.pdf]
