# Supplementary material for: Synthesis of Diaryl‐ and Dialkynylphosphinates From Ubiquitous PV Sources via a Redox‐Neutral Approach
Source: Adv Sci (Weinh). 2025 Jul 2;12(36):e09922. doi: 10.1002/advs.202509922 (PMC12463002; doi:10.1002/advs.202509922)

checkCIF/PLATON report

Structure factors have been supplied for datablock(s) jjw1756

THIS REPORT IS FOR GUIDANCE ONLY. IF USED AS PART OF A REVIEW PROCEDURE FOR PUBLICATION, IT SHOULD NOT REPLACE THE EXPERTISE OF AN EXPERIENCED CRYSTALLOGRAPHIC REFEREE.

No syntax errors found. CIF dictionary Interpreting this report

Datablock: jjw1756

Bond precision:	C-C = 0.0034 A		Wavelength=0.71073
Cell:	a=24.9323(5)	b=6.1743(1)	c=33.2509(6)
	alpha=90	beta=99.364(2)	gamma=90
Temperature:	100 K		

	Calculated	Reported
Volume	5050.42(16)	5050.42(16)
Space group	I 2/a	I 1 2/a 1
Hall group	-I 2ya	-I 2ya
Moiety formula	C24 H26 O2 P, H3 O [+ solvent]	C24 H26 O2 P, H3 O, 0.5[C4H10O]
Sum formula	C24 H29 O3 P [+ solvent]	C26 H34 O3.50 P
Mr	396.44	433.50
Dx,g cm-3	1.043	1.140
Z	8	8
Mu (mm-1)	0.127	0.134
F000	1696.0	1864.0
F000’	1697.52	
h,k,lmax	30,7,40	30,7,40
Nref	4642	4563
Tmin,Tmax	0.979,0.996	0.959,0.997
Tmin’	0.914	

Correction method= # Reported T Limits: Tmin=0.959 Tmax=0.997
AbsCorr = ANALYTICAL

Data completeness= 0.983 Theta(max)= 25.345

R(reflections)= 0.0522(3969) wR2(reflections)= 0.1314(4563)

S = 1.042 Npar= 326

The following ALERTS were generated. Each ALERT has the format
test-name_ALERT_alert-type_alert-level.
Click on the hyperlinks for more details of the test.

Alert level C

```
PLAT220_ALERT_2_C NonSolvent   Resd 1  C   Ueq(max)/Ueq(min) Range        4.0 Ratio
PLAT222_ALERT_3_C NonSolvent Resd 1  H   Uiso(max)/Uiso(min) Range        4.1 Ratio
PLAT242_ALERT_2_C Low    'MainMol' Ueq as Compared to Neighbors of        C21 Check
PLAT313_ALERT_2_C Oxygen with Three Covalent Bonds (rare) ........         O3 Check
PLAT906_ALERT_3_C Large K Value in the Analysis of Variance ......      2.245 Check
PLAT910_ALERT_3_C Missing # of FCF Reflection(s) Below Theta(Min).          6 Note
                2  0  0,  -2  0  2,   0  0  2,   2  0  2,  -2  0  4,   0  0  4,
PLAT911_ALERT_3_C Missing FCF Refl Between Thmin & STh/L=    0.600         70 Report
                0  0  6,   0  0  8,   0  0 10,   0  0 12, -20  0 24, -18  0 24,
              -16  0 24, -24  0 26, -22  0 26, -20  0 26, -18  0 26, -16  0 26,
              -24  0 28, -22  0 28, -20  0 28, -18  0 28, -16  0 28, -14  0 28,
              -10  0 28, -13  2 29, -22  0 30, -20  0 30, -18  0 30, -16  0 30,
              -14  2 30, -10  0 30, -15  2 31, -10  1 31, -21  1 32, -20  0 32,
              -18  0 32, -16  0 32, -16  2 32, -14  0 32, -13  1 32, -12  0 32,
              -11  1 32, -10  0 32, -20  1 33, -18  1 33, -17  2 33, -16  1 33,
              -15  2 33, -14  1 33, -12  1 33, -10  1 33, -19  1 34, -18  0 34,
              -17  1 34, -16  0 34, -16  2 34, -15  1 34, -14  0 34, -13  1 34,
              -12  0 34, -11  1 34, -10  0 34, -16  1 35, -14  1 35, -12  1 35,
              -16  0 36, -15  1 36, -14  0 36, -13  1 36, -12  0 36, -10  0 36,
              -14  1 37, -12  1 37, -12  0 38, -10  0 38,
```

Alert level G

```
FORMU01_ALERT_2_G  There is a discrepancy between the atom counts in the
            _chemical_formula_sum and the formula from the _atom_site* data.
            Atom count from _chemical_formula_sum:C26 H34 O3.5 P1
            Atom count from the _atom_site data:  C24 H29 O3 P1
CELLZ01_ALERT_1_G Difference between formula and atom_site contents detected.
CELLZ01_ALERT_1_G ALERT: Large difference may be due to a
            symmetry error - see SYMMG tests
           From the CIF: _cell_formula_units_Z    8
           From the CIF: _chemical_formula_sum  C26 H34 O3.50 P
           TEST: Compare cell contents of formula and atom_site data

           atom    Z*formula  cif sites diff
           C        208.00    192.00   16.00
           H        272.00    232.00   40.00
           O         28.00     24.00    4.00
           P          8.00      8.00    0.00
PLAT003_ALERT_2_G Number of Uiso or U(i,j) Restrained non-H Atoms          14 Report
PLAT007_ALERT_5_G Number of Unrefined Donor-H Atoms .............           2 Report
              H3A   H3B
PLAT041_ALERT_1_G Calc. and Reported SumFormula    Strings  Differ     Please Check
              Calc: C24 H29 O3 P
              Rep.: C26 H34 O3.50 P
PLAT042_ALERT_1_G Calc. and Reported MoietyFormula Strings  Differ     Please Check
              Calc: C24 H26 O2 P, H3 O
              Rep.: C24 H26 O2 P, H3 O, 0.5[C4H10O]
PLAT063_ALERT_4_G Crystal Size Possibly too Large for Beam Size ..       0.67 mm
PLAT083_ALERT_2_G SHELXL Second Parameter in WGHT  Unusually Large      11.31 Why ?
```

```
PLAT178_ALERT_4_G The CIF-Embedded .res File Contains SIMU Records          1 Report
PLAT187_ALERT_4_G The CIF-Embedded .res File Contains RIGU Records          1 Report
PLAT188_ALERT_3_G A Non-default SIMU Restraint Value has been used     0.0100 Report
PLAT230_ALERT_2_G Hirshfeld Test Diff for    P1       --C1       .        5.6 s.u.
PLAT300_ALERT_4_G Atom Site Occupancy of C16A       Constrained at        0.4 Check
PLAT300_ALERT_4_G Atom Site Occupancy of C16B       Constrained at        0.4 Check
PLAT300_ALERT_4_G Atom Site Occupancy of C16C       Constrained at        0.2 Check
PLAT300_ALERT_4_G Atom Site Occupancy of C17A       Constrained at        0.4 Check
PLAT300_ALERT_4_G Atom Site Occupancy of C17B       Constrained at        0.4 Check
PLAT300_ALERT_4_G Atom Site Occupancy of C17C       Constrained at        0.2 Check
PLAT300_ALERT_4_G Atom Site Occupancy of C19A       Constrained at        0.4 Check
PLAT300_ALERT_4_G Atom Site Occupancy of C19B       Constrained at        0.4 Check
PLAT300_ALERT_4_G Atom Site Occupancy of C19C       Constrained at        0.2 Check
PLAT300_ALERT_4_G Atom Site Occupancy of C20A       Constrained at        0.4 Check
PLAT300_ALERT_4_G Atom Site Occupancy of C20B       Constrained at        0.4 Check
PLAT300_ALERT_4_G Atom Site Occupancy of C20C       Constrained at        0.2 Check
PLAT300_ALERT_4_G Atom Site Occupancy of H16A       Constrained at        0.4 Check
PLAT300_ALERT_4_G Atom Site Occupancy of H16B       Constrained at        0.4 Check
PLAT300_ALERT_4_G Atom Site Occupancy of H16C       Constrained at        0.2 Check
PLAT300_ALERT_4_G Atom Site Occupancy of H17A       Constrained at        0.4 Check
PLAT300_ALERT_4_G Atom Site Occupancy of H17B       Constrained at        0.4 Check
PLAT300_ALERT_4_G Atom Site Occupancy of H17C       Constrained at        0.2 Check
PLAT300_ALERT_4_G Atom Site Occupancy of H19A       Constrained at        0.4 Check
PLAT300_ALERT_4_G Atom Site Occupancy of H19B       Constrained at        0.4 Check
PLAT300_ALERT_4_G Atom Site Occupancy of H19C       Constrained at        0.2 Check
PLAT300_ALERT_4_G Atom Site Occupancy of H20A       Constrained at        0.4 Check
PLAT300_ALERT_4_G Atom Site Occupancy of H20B       Constrained at        0.4 Check
PLAT300_ALERT_4_G Atom Site Occupancy of H20C       Constrained at        0.2 Check
PLAT301_ALERT_3_G Main Residue  Disorder ..............(Resd    1)        15% Note
PLAT335_ALERT_2_G Check Large C6 Ring C-C Range C15      -C20B           0.29 Ang.
PLAT335_ALERT_2_G Check Large C6 Ring C-C Range C15      -C20C           0.30 Ang.
PLAT371_ALERT_2_G Long   C(sp2)-C(sp1) Bond  C2       - C3       .       1.43 Ang.
PLAT371_ALERT_2_G Long   C(sp2)-C(sp1) Bond  C14      - C15      .       1.45 Ang.
PLAT412_ALERT_2_G Short Intra XH3 .. XHn     H22A     ..H17B     .       2.07 Ang.
                                                    x,y,z  =     1_555 Check
PLAT412_ALERT_2_G Short Intra XH3 .. XHn     H23A     ..H19C     .       2.11 Ang.
                                                    x,y,z  =     1_555 Check
PLAT412_ALERT_2_G Short Intra XH3 .. XHn     H23C     ..H19C     .       2.12 Ang.
                                                    x,y,z  =     1_555 Check
PLAT412_ALERT_2_G Short Intra XH3 .. XHn     H24C     ..H19A     .       2.05 Ang.
                                                    x,y,z  =     1_555 Check
PLAT412_ALERT_2_G Short Intra XH3 .. XHn     H24C     ..H19B     .       2.10 Ang.
                                                    x,y,z  =     1_555 Check
PLAT413_ALERT_2_G Short Inter XH3 .. XHn     H22C     ..H19B     .       2.13 Ang.
                                                  x,1+y,z  =     1_565 Check
PLAT413_ALERT_2_G Short Inter XH3 .. XHn     H22C     ..H16C     .       2.07 Ang.
                                              1-x,2-y,1-z  =     5_676 Check
PLAT413_ALERT_2_G Short Inter XH3 .. XHn     H24A     ..H16C     .       2.07 Ang.
                                              1-x,2-y,1-z  =     5_676 Check
PLAT605_ALERT_4_G Largest Solvent Accessible VOID in the Structure        124 A**3
PLAT860_ALERT_3_G Number of Least-Squares Restraints ............         348 Note
PLAT868_ALERT_4_G ALERTS Due to the Use of _smtbx_masks Suppressed          ! Info
PLAT912_ALERT_4_G Missing # of FCF Reflections Above STh/L=  0.600          3 Note
PLAT933_ALERT_2_G Number of HKL-OMIT Records in Embedded .res File          1 Note
               -2  0  4,
PLAT941_ALERT_3_G Average HKL Measurement Multiplicity ..........         4.1 Low
PLAT969_ALERT_5_G The 'Henn et al.' R-Factor-gap value ..........       6.632 Note
```

```
            Predicted wR2: Based on SigI**2  1.98 or SHELX Weight 12.61
PLAT978_ALERT_2_G Number C-C Bonds with Positive Residual Density.         6 Info
```

```
  0 ALERT level A = Most likely a serious problem - resolve or explain
  0 ALERT level B = A potentially serious problem, consider carefully
  7 ALERT level C = Check. Ensure it is not caused by an omission or oversight
 58 ALERT level G = General information/check it is not something unexpected

  4 ALERT type 1 CIF construction/syntax error, inconsistent or missing data
 21 ALERT type 2 Indicator that the structure model may be wrong or deficient
  8 ALERT type 3 Indicator that the structure quality may be low
 30 ALERT type 4 Improvement, methodology, query or suggestion
  2 ALERT type 5 Informative message, check
```

It is advisable to attempt to resolve as many as possible of the alerts in all categories. Often the minor alerts point to easily fixed oversights, errors and omissions in your CIF or refinement strategy, so attention to these fine details can be worthwhile. In order to resolve some of the more serious problems it may be necessary to carry out additional measurements or structure refinements. However, the purpose of your study may justify the reported deviations and the more serious of these should normally be commented upon in the discussion or experimental section of a paper or in the "special_details" fields of the CIF. checkCIF was carefully designed to identify outliers and unusual parameters, but every test has its limitations and alerts that are not important in a particular case may appear. Conversely, the absence of alerts does not guarantee there are no aspects of the results needing attention. It is up to the individual to critically assess their own results and, if necessary, seek expert advice.

Publication of your CIF in IUCr journals

A basic structural check has been run on your CIF. These basic checks will be run on all CIFs submitted for publication in IUCr journals (*Acta Crystallographica*, *Journal of Applied Crystallography*, *Journal of Synchrotron Radiation*); however, if you intend to submit to *Acta Crystallographica Section C* or *E* or *IUCrData*, you should make sure that full publication checks are run on the final version of your CIF prior to submission.

Publication of your CIF in other journals

Please refer to the *Notes for Authors* of the relevant journal for any special instructions relating to CIF submission.

PLATON version of 13/05/2024; check.def file version of 04/05/2024

Datablock jjw1756 - ellipsoid plot

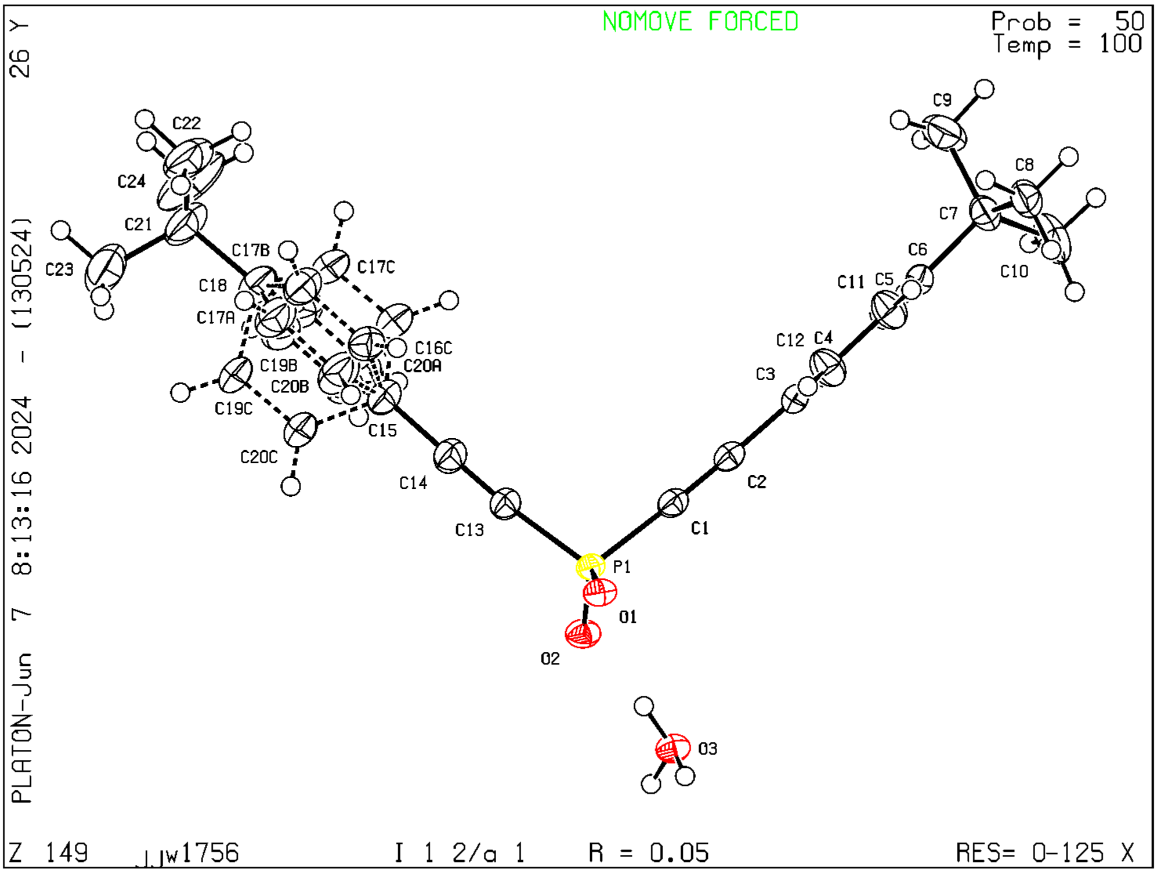

Supplement: Supplementary file 2 — Supporting Information [file ADVS-12-e09922-s001.zip › 9d_H2O_05Et2O_jjw1756.pdf]
