# Supplementary material for: Synthesis of Diaryl‐ and Dialkynylphosphinates From Ubiquitous PV Sources via a Redox‐Neutral Approach
Source: Adv Sci (Weinh). 2025 Jul 2;12(36):e09922. doi: 10.1002/advs.202509922 (PMC12463002; doi:10.1002/advs.202509922)

checkCIF/PLATON report

Structure factors have been supplied for datablock(s) jjw1820

THIS REPORT IS FOR GUIDANCE ONLY. IF USED AS PART OF A REVIEW PROCEDURE FOR PUBLICATION, IT SHOULD NOT REPLACE THE EXPERTISE OF AN EXPERIENCED CRYSTALLOGRAPHIC REFEREE.

No syntax errors found.   CIF dictionary   Interpreting this report

Datablock: jjw1820

```
Bond precision:   C-C = 0.0020 A          Wavelength=1.54184

Cell:            a=7.1542(2)        b=7.7424(3)       c=19.1720(5)
                 alpha=87.878(2)    beta=86.632(2)    gamma=85.020(2)
Temperature:     100 K

                  Calculated                  Reported
Volume            1055.57(6)                  1055.57(6)
Space group       P -1                        P -1
Hall group        -P 1                        -P 1
Moiety formula    C18 H8 F6 O2 P, C5 H6 N     C18 H8 F6 O2 P, C5 H6 N
Sum formula       C23 H14 F6 N O2 P           C23 H14 F6 N O2 P
Mr                481.32                      481.32
Dx,g cm-3         1.514                       1.514
Z                 2                           2
Mu (mm-1)         1.843                       1.843
F000              488.0                       488.0
F000'             490.50
h,k,lmax          9,9,24                      9,9,24
Nref              4447                        4374
Tmin,Tmax         0.784,0.929                 0.804,0.934
Tmin'             0.758

Correction method= # Reported T Limits: Tmin=0.804 Tmax=0.934
AbsCorr = ANALYTICAL

Data completeness= 0.984          Theta(max)= 76.804

                                                     wR2(reflections)=
R(reflections)= 0.0370( 3894)                        0.0939( 4374)

S = 1.038                  Npar= 298
```

The following ALERTS were generated. Each ALERT has the format
test-name_ALERT_alert-type_alert-level.
Click on the hyperlinks for more details of the test.

Alert level C

```
PLAT242_ALERT_2_C Low    'MainMol' Ueq as Compared to Neighbors of         C9 Check
```

Alert level G

```
PLAT007_ALERT_5_G Number of Unrefined Donor-H Atoms .............           1 Report
              H1
PLAT154_ALERT_1_G The s.u.'s on the Cell Angles are Equal ..(Note)      0.002 Degree
PLAT230_ALERT_2_G Hirshfeld Test Diff for    P1       --C1       .        7.4 s.u.
PLAT230_ALERT_2_G Hirshfeld Test Diff for    P1       --C10      .        7.3 s.u.
PLAT371_ALERT_2_G Long   C(sp2)-C(sp1) Bond  C2       - C3       .       1.44 Ang.
PLAT371_ALERT_2_G Long   C(sp2)-C(sp1) Bond  C11      - C12      .       1.43 Ang.
PLAT912_ALERT_4_G Missing # of FCF Reflections Above STh/L=  0.600         72 Note
PLAT941_ALERT_3_G Average HKL Measurement Multiplicity ..........         2.4 Low
PLAT969_ALERT_5_G The 'Henn et al.' R-Factor-gap value ..........       2.636 Note
              Predicted wR2: Based on SigI**2  3.56 or SHELX Weight  9.04
PLAT978_ALERT_2_G Number C-C Bonds with Positive Residual Density.         15 Info
```

0 **ALERT level A** = Most likely a serious problem - resolve or explain
0 **ALERT level B** = A potentially serious problem, consider carefully
1 **ALERT level C** = Check. Ensure it is not caused by an omission or oversight
10 **ALERT level G** = General information/check it is not something unexpected

1 ALERT type 1 CIF construction/syntax error, inconsistent or missing data
6 ALERT type 2 Indicator that the structure model may be wrong or deficient
1 ALERT type 3 Indicator that the structure quality may be low
1 ALERT type 4 Improvement, methodology, query or suggestion
2 ALERT type 5 Informative message, check

It is advisable to attempt to resolve as many as possible of the alerts in all categories. Often the minor alerts point to easily fixed oversights, errors and omissions in your CIF or refinement strategy, so attention to these fine details can be worthwhile. In order to resolve some of the more serious problems it may be necessary to carry out additional measurements or structure refinements. However, the purpose of your study may justify the reported deviations and the more serious of these should normally be commented upon in the discussion or experimental section of a paper or in the "special_details" fields of the CIF. checkCIF was carefully designed to identify outliers and unusual parameters, but every test has its limitations and alerts that are not important in a particular case may appear. Conversely, the absence of alerts does not guarantee there are no aspects of the results needing attention. It is up to the individual to critically assess their own results and, if necessary, seek expert advice.

Publication of your CIF in IUCr journals

A basic structural check has been run on your CIF. These basic checks will be run on all CIFs submitted for publication in IUCr journals (*Acta Crystallographica*, *Journal of Applied Crystallography*, *Journal of Synchrotron Radiation*); however, if you intend to submit to *Acta Crystallographica Section C* or *E* or *IUCrData*, you should make sure that full publication checks are run on the final version of your CIF prior to submission.

Publication of your CIF in other journals

Please refer to the *Notes for Authors* of the relevant journal for any special instructions relating to CIF submission.

PLATON version of 22/08/2024; check.def file version of 21/08/2024

Datablock jjw1820 - ellipsoid plot

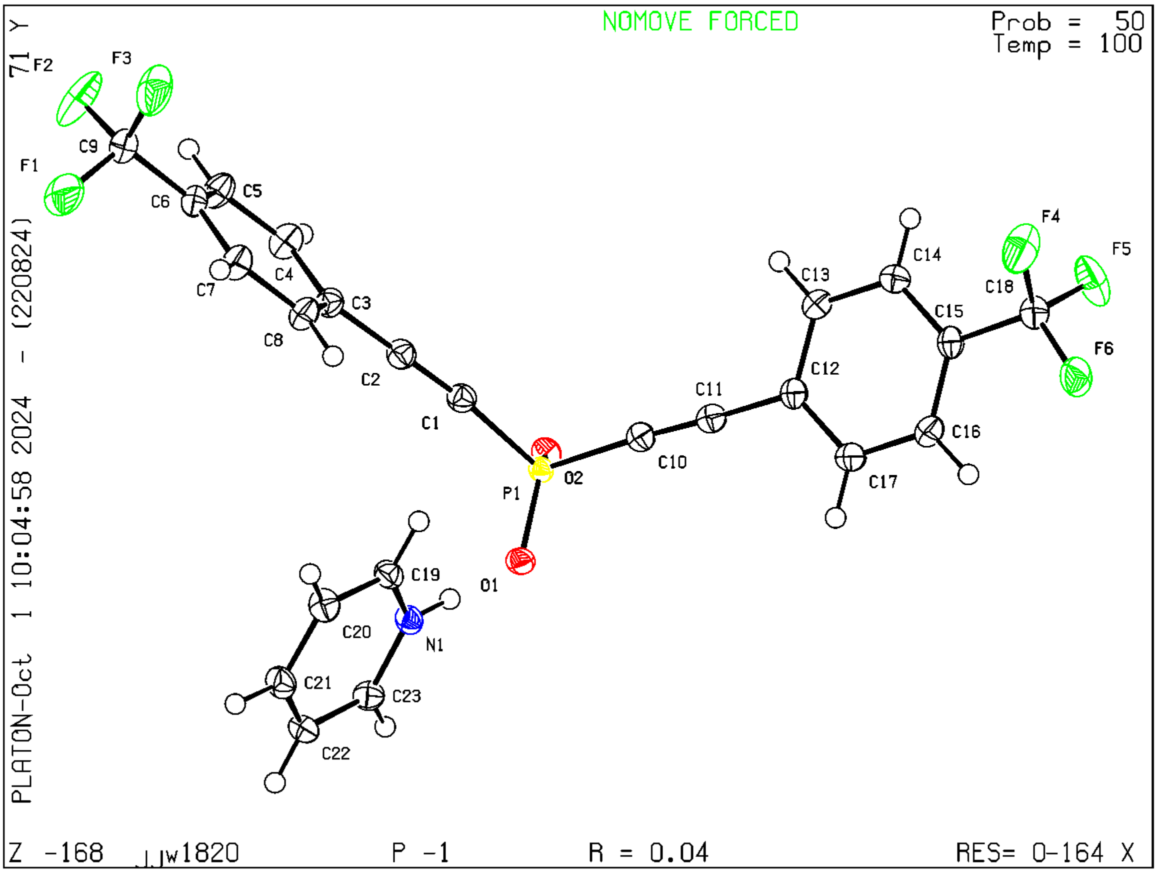

Supplement: Supplementary file 2 — Supporting Information [file ADVS-12-e09922-s001.zip › 9g_C5H5N_jjw1820.pdf]
