# Supplementary material for: Synthesis of Diaryl‐ and Dialkynylphosphinates From Ubiquitous PV Sources via a Redox‐Neutral Approach
Source: Adv Sci (Weinh). 2025 Jul 2;12(36):e09922. doi: 10.1002/advs.202509922 (PMC12463002; doi:10.1002/advs.202509922)

# checkCIF/PLATON report

Structure factors have been supplied for datablock(s) jjw1770

THIS REPORT IS FOR GUIDANCE ONLY. IF USED AS PART OF A REVIEW PROCEDURE FOR PUBLICATION, IT SHOULD NOT REPLACE THE EXPERTISE OF AN EXPERIENCED CRYSTALLOGRAPHIC REFEREE.

No syntax errors found. CIF dictionary Interpreting this report

# Datablock: jjw1770

---

```
Bond precision:   C-C = 0.0032 A          Wavelength=0.71073

Cell:              a=6.1562(1)     b=16.7127(3)      c=23.8964(5)
                   alpha=90        beta=90           gamma=90
Temperature:       100 K

                Calculated                  Reported
Volume          2458.62(8)                  2458.62(8)
Space group     P 21 21 21                  P 21 21 21
Hall group      P 2ac 2ab                   P 2ac 2ab
Moiety formula  C24 H14 O2 P, C4 H8 O, H4 N C24 H14 O2 P, C4 H8 O, H4 N
Sum formula     C28 H26 N O3 P              C28 H26 N O3 P
Mr              455.47                      455.47
Dx,g cm-3       1.230                       1.230
Z               4                           4
Mu (mm-1)       0.141                       0.141
F000            960.0                       960.0
F000'           960.80
h,k,lmax        8,24,34                     8,23,34
Nref            7756[ 4393]                 6994
Tmin,Tmax       0.987,0.999                 0.978,0.998
Tmin'           0.975

Correction method= # Reported T Limits: Tmin=0.978 Tmax=0.998
AbsCorr = ANALYTICAL

Data completeness= 1.59/0.90       Theta(max)= 30.909

                                                      wR2(reflections)=
R(reflections)= 0.0435( 6099)                         0.0912( 6994)

S = 1.066                  Npar= 314
```

The following ALERTS were generated. Each ALERT has the format

**test-name_ALERT_alert-type_alert-level.**

Click on the hyperlinks for more details of the test.

## Alert level G

```
PLAT066_ALERT_1_G Predicted and Reported Tmin&Tmax Range Identical          ? Check
PLAT371_ALERT_2_G Long   C(sp2)-C(sp1) Bond  C2       - C3       .       1.43 Ang.
PLAT371_ALERT_2_G Long   C(sp2)-C(sp1) Bond  C14      - C15      .       1.43 Ang.
PLAT398_ALERT_2_G Deviating  C-O-C   Angle From 120 for O3       .      108.4 Degree
PLAT850_ALERT_4_G Check Flack Parameter Exact Value 0.00 with s.u.       0.03 Check
PLAT910_ALERT_3_G Missing # of FCF Reflection(s) Below Theta(Min).          3 Note
                0  1  1,   0  0  2,   0  1  2,
PLAT912_ALERT_4_G Missing # of FCF Reflections Above STh/L=  0.600        269 Note
PLAT969_ALERT_5_G The 'Henn et al.' R-Factor-gap value ...........      3.623 Note
              Predicted wR2: Based on SigI**2  2.52 or SHELX Weight  8.56
PLAT978_ALERT_2_G Number C-C Bonds with Positive Residual Density.         11 Info
```

```
   0 ALERT level A = Most likely a serious problem - resolve or explain
   0 ALERT level B = A potentially serious problem, consider carefully
   0 ALERT level C = Check. Ensure it is not caused by an omission or oversight
   9 ALERT level G = General information/check it is not something unexpected

   1 ALERT type 1 CIF construction/syntax error, inconsistent or missing data
   4 ALERT type 2 Indicator that the structure model may be wrong or deficient
   1 ALERT type 3 Indicator that the structure quality may be low
   2 ALERT type 4 Improvement, methodology, query or suggestion
   1 ALERT type 5 Informative message, check
```

It is advisable to attempt to resolve as many as possible of the alerts in all categories. Often the minor alerts point to easily fixed oversights, errors and omissions in your CIF or refinement strategy, so attention to these fine details can be worthwhile. In order to resolve some of the more serious problems it may be necessary to carry out additional measurements or structure refinements. However, the purpose of your study may justify the reported deviations and the more serious of these should normally be commented upon in the discussion or experimental section of a paper or in the "special_details" fields of the CIF. checkCIF was carefully designed to identify outliers and unusual parameters, but every test has its limitations and alerts that are not important in a particular case may appear. Conversely, the absence of alerts does not guarantee there are no aspects of the results needing attention. It is up to the individual to critically assess their own results and, if necessary, seek expert advice.

**Publication of your CIF in IUCr journals**

A basic structural check has been run on your CIF. These basic checks will be run on all CIFs submitted for publication in IUCr journals (*Acta Crystallographica*, *Journal of Applied Crystallography*, *Journal of Synchrotron Radiation*); however, if you intend to submit to *Acta Crystallographica Section C* or *E* or *IUCrData*, you should make sure that full publication checks are run on the final version of your CIF prior to submission.

**Publication of your CIF in other journals**

Please refer to the *Notes for Authors* of the relevant journal for any special instructions relating to CIF submission.

---

**PLATON version of 15/07/2024; check.def file version of 15/07/2024**

**Datablock jjw1770** - ellipsoid plot

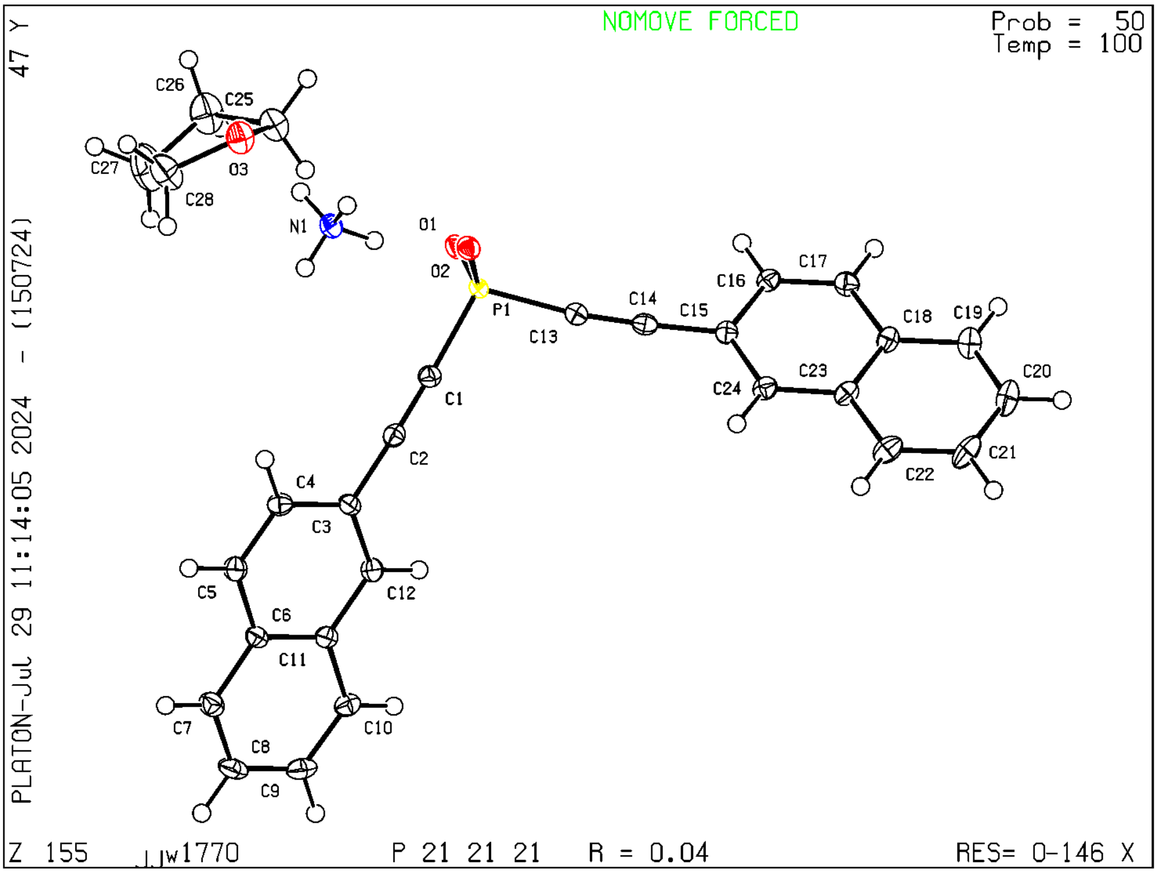

Supplement: Supplementary file 2 — Supporting Information [file ADVS-12-e09922-s001.zip › 9h_NH3_THF_jjw1770.pdf]
